# Supplementary material for: Reference Intervals and Percentiles for Hematologic and Serum Biochemical Values in Captive Bred Rhesus (Macaca mulatta) and Cynomolgus Macaques (Macaca fascicularis)
Source: Animals (Basel). 2023 Jan 28;13(3):445. doi: 10.3390/ani13030445 (PMC9913310; doi:10.3390/ani13030445)
Supplement: Supplementary file 1 [file animals-13-00445-s001.zip › Description of supplementary Figures.pdf]

# Supplementary Figure descriptions

## Figure S1:

A. Paired comparisons between years in adult rhesus and cynomolgus macaques, numbers indicate percentual differences (Delta) between Year2 – Year1.
B. Unpaired comparisons between years in adult rhesus and cynomolgus macaques, numbers indicate percentual differences (Delta) between Year2 – Year1.
C. Unpaired comparisons between years in juvenile rhesus and cynomolgus macaques, numbers indicate percentual differences (Delta) between Year2 – Year1.
D. Unpaired comparisons between years in rhesus and cynomolgus macaques, numbers indicate percentual differences (Delta) between 20-21 – 18-19.

## Figures S2 1 to 36:

Laboratory tests according to gender, age and collection period in rhesus and cynomolgus macaques. P1 = 2018 and 2019, P2 = 2020 and 2021. Boxes indicate median and quartile ranges, black point indicates arithmetic mean and error bars indicate the normal range. Round symbols indicate animals with outdoor access and triangles indicate indoor housed animals. Numbers refer to page and laboratory test, see table at end of this document.

## Figure S3:

Comparisons between gender and age in rhesus and cynomolgus macaques, numbers indicate percentual differences (Delta) between. YF-YM = Young Females and Males, YF-AF = Young and Adult Females, YM-AM = Young and Adult Males, AF-AM = Adult Females and Males.

## Figures S4 1 to 36:

Laboratory tests according to gender and age in rhesus and cynomolgus macaques. Boxes indicate median and quartile ranges, black point indicates arithmetic mean and error bars indicate the normal range. Round symbols indicate animals with outdoor access and triangles indicate indoor housed animals. Numbers refer to page and laboratory test, see table at end of this document.

## Figure S5:

Comparisons between adult rhesus macaques with or without outdoor access, numbers indicate percentual differences (Delta) between outdoor – indoor.

## Figures S6 1 to 36:

Laboratory tests according to outdoor access in adult rhesus and cynomolgus macaques. Boxes indicate median and quartile ranges, black point indicates arithmetic mean and error bars indicate the normal range. Numbers refer to page and laboratory test, see table at end of this document.

## Figure S7:

Comparisons between non-overweight and overweight adult rhesus and cynomolgus, numbers indicate percentual differences (Delta) between non-overweight and overweight.

## Figures S8 1 to 36:

Laboratory tests according to body weight in adult rhesus and cynomolgus macaques. Boxes indicate median and quartile ranges, black point indicates arithmetic mean and error bars indicate the normal range. Numbers refer to page and laboratory test, see table at end of this document.

## Figure S9:

Comparisons between non-pregnant and pregnant adult rhesus and cynomolgus females, numbers indicate percentual differences (Delta) between non-pregnant – pregnant.

## Figures S10 1 to 36:

Laboratory tests according to pregnancy in adult female rhesus and cynomolgus macaques. Boxes indicate median and quartile ranges, black point indicates arithmetic mean and error bars indicate the normal range.

| Nr | Test name | Description |
|---|---|---|
| **1** | ALB | Albumin |
| **2** | TP | Protein total |
| **3** | ALP | Alkaline phosphatase |
| **4** | ALT | ALAT |
| **5** | AST | ASAT |
| **6** | GGT | Gamma-GT |
| **7** | LDH | LDH |
| **8** | BILT | Bilirubin total |
| **9** | CHOL | Cholesterol |
| **10** | Cl | Chloride |
| **11** | CO2 | Bicarbonate |
| **12** | Fe | Iron |
| **13** | K | Potassium |
| **14** | Na | Sodium |
| **15** | Phos | Phosphate |
| **16** | Ca | Calcium |
| **17** | GLU | Glucose, split for 2018-2019 (A)and 2020-2021 (B) |
| **18** | URE | Urea |
| **19** | Cre | Creatinine |
| **20** | RBC | Red blood cell count |
| **21** | HGB | Hemoglobin |
| **22** | HCT | Hematocrit |
| **23** | MCV | Mean corpuscular volume |
| **24** | MCH | Mean corpuscular hemoglobin |
| **25** | MCHC | Mean corpuscular hemoglobin concentration |
| **26** | PLT | Platelet count |
| **27** | PDW | Platelet distribution width |
| **28** | MPV | Mean platelet volume |
| **29** | P-LCR | Platelet larger cell ratio |
| **30** | PCT | Plateletcrit |
| **31** | WBC | White blood cell count |
| **32** | Neut | Neutrophil count |
| **33** | Mono | Monocyte count |
| **34** | Lymph | Lymphocyte count |
| **35** | Eo | Eosinophil count |
| **36** | Baso | Basophil count |
