# Supplementary material for: Reference Intervals and Percentiles for Hematologic and Serum Biochemical Values in Captive Bred Rhesus (Macaca mulatta) and Cynomolgus Macaques (Macaca fascicularis)
Source: Animals (Basel). 2023 Jan 28;13(3):445. doi: 10.3390/ani13030445 (PMC9913310; doi:10.3390/ani13030445)
Supplement: Supplementary file 1 [file animals-13-00445-s001.zip › Figure S1.pdf]

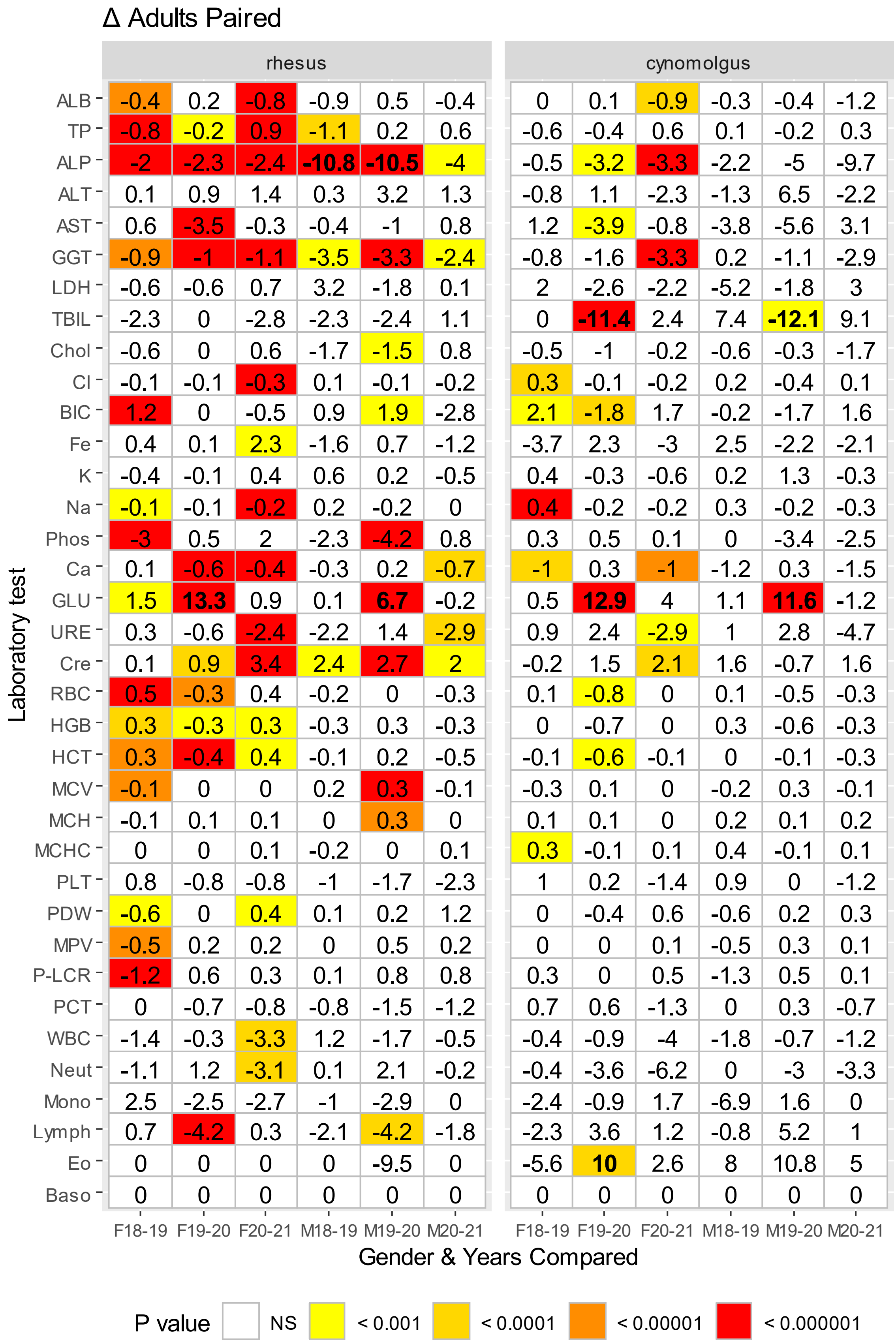

Figure S1 A. Paired comparisons between years in adult rhesus and cynomolgus macaques, numbers indicate percentual differences (Delta) between Year2 – Year1.

Δ Adults Unpaired

Laboratory test	rhesus						cynomolgus					
	F18-19	F19-20	F20-21	M18-19	M19-20	M20-21	F18-19	F19-20	F20-21	M18-19	M19-20	M20-21
ALB	-0.2	0	-0.2	-0.8	0.8	-0.5	0.7	0.3	-1	0.7	-0.2	-1
TP	-0.8	-0.3	0.9	-1.7	0.6	0.5	-0.9	-0.4	0.5	-0.6	-0.7	1
ALP	0.2	-1.8	-0.2	4.1	**-10.9**	-2.9	0	-2.2	-5	11.7	-3.6	-15.2
ALT	-1.5	0.6	0.3	-4.9	4.2	-0.2	-3.3	-0.1	0.1	-5.2	0.2	0.2
AST	0.3	-3.3	1	0	-0.3	-0.1	1.1	-3.7	-0.6	-1.2	-1.9	0.8
GGT	0	-1.5	0.1	-0.3	-1.8	-0.4	-2.2	-0.4	-3.2	-0.7	1.2	1.7
LDH	-1.3	-0.7	0.9	-1.5	-0.3	-0.3	2.6	-4.4	-1.5	-1.3	-1.3	5.8
TBIL	-2.4	0	-5.6	0	2.4	0	-1.9	**-9.1**	5	**10.7**	-13	4.5
Chol	-0.6	-0.7	0.1	-1.6	-0.3	0.7	0	-0.1	-0.9	-1.7	0.5	-1.8
Cl	0	-0.1	-0.3	0.1	-0.1	-0.2	0.4	-0.1	-0.2	0.2	-0.2	-0.3
BIC	1.1	-0.1	-0.8	0.3	1.7	-2.3	1.6	-1.3	1.4	1.2	-0.7	0.8
Fe	-0.5	-0.9	2.3	-2	0.2	-1.2	-2.2	2.5	-2.7	1.6	-1.3	-0.9
K	-0.3	-0.2	0.5	-0.5	0.4	-0.3	1.3	-0.4	-0.1	0.9	0.3	0
Na	0	-0.1	-0.2	0.1	-0.2	-0.1	0.5	-0.3	-0.1	0.1	-0.1	-0.2
Phos	-1.4	0.7	2.3	1.4	-2.4	1.2	0	0.6	-0.9	3.4	-3.4	1
Ca	0	-0.4	-0.1	-0.6	0	-0.5	-0.4	0.7	-0.9	-0.5	0	-0.4
GLU	0.8	**13**	0.7	-0.7	**9**	2.6	-0.8	**15.3**	2.4	-0.4	**13.6**	-2.1
URE	0.8	0.4	-1.6	0.2	1.9	-3.4	1.5	2.6	-2.3	-1.9	4.6	-1.3
Cre	0.1	0.5	3	0.3	1.1	1.7	-0.6	1.1	2.9	-0.6	0.9	3.2
RBC	0.3	-0.7	0.7	-0.1	-0.3	-0.1	-0.6	-0.7	-0.3	-0.4	-0.5	-1.1
HGB	0.3	-0.9	0.3	-0.6	0.4	-0.4	0	-0.3	0	0.3	0	-0.6
HCT	0.1	-0.6	0.1	-0.4	0.1	-0.3	-0.2	-0.4	-0.1	-0.3	-0.4	-0.9
MCV	-0.1	-0.1	-0.2	0	0.1	-0.2	0.3	0.4	0.2	-0.1	0.6	-0.5
MCH	-0.1	0	-0.1	0	0.3	-0.3	0.9	0.5	0.3	0.5	0.5	0.2
MCHC	0	0.1	0.1	-0.1	0.1	0.2	0.6	0	0.1	0.7	0	0.1
PLT	1.1	-0.8	-2	0.9	-1.5	-2.3	1	0.5	-1.8	2.3	-2.3	1.1
PDW	-0.6	0	1.4	0	0	1.4	0.6	-0.5	0.5	0	0.3	-0.2
MPV	-0.2	0.2	0.7	0	0.2	1.1	0.3	0	0.3	0	0.5	-0.1
P-LCR	-0.6	0.3	1.4	-0.2	1	1.8	1.6	-1.8	1.5	-0.5	1.7	-0.2
PCT	0.7	-0.7	-0.7	0.7	-0.7	-1.6	1.3	0	-1.3	2	-2	-0.3
WBC	0.6	-0.8	-1.8	-1.3	1.3	-0.5	0.2	-0.5	-4	0.6	-0.7	1.1
Neut	-0.8	-0.1	-2.1	-4.7	4.8	0.2	3.3	-2.4	-6.2	3.7	-4.3	4.3
Mono	3	-1.7	-2	-2.9	0.8	-2.3	-2.4	-0.8	0.4	-1.4	2.7	5.1
Lymph	0.9	-3	1.8	3.4	-2.2	-2.6	-3.8	5.9	4.4	-0.6	-0.4	5.7
Eo	16.7	-12.5	**20**	8.3	-13.6	13.6	-11.1	**16.7**	0	8.5	3.7	3.8
Baso	0	0	0	0	0	0	0	0	0	0	0	0

Gender & Years Compared

P value: NS; < 0.001; < 0.0001; < 0.00001; < 0.000001

Figure S1 B. Unpaired comparisons between years in adult rhesus and cynomolgus macaques, numbers indicate percentual differences (Delta) between Year2 – Year1.

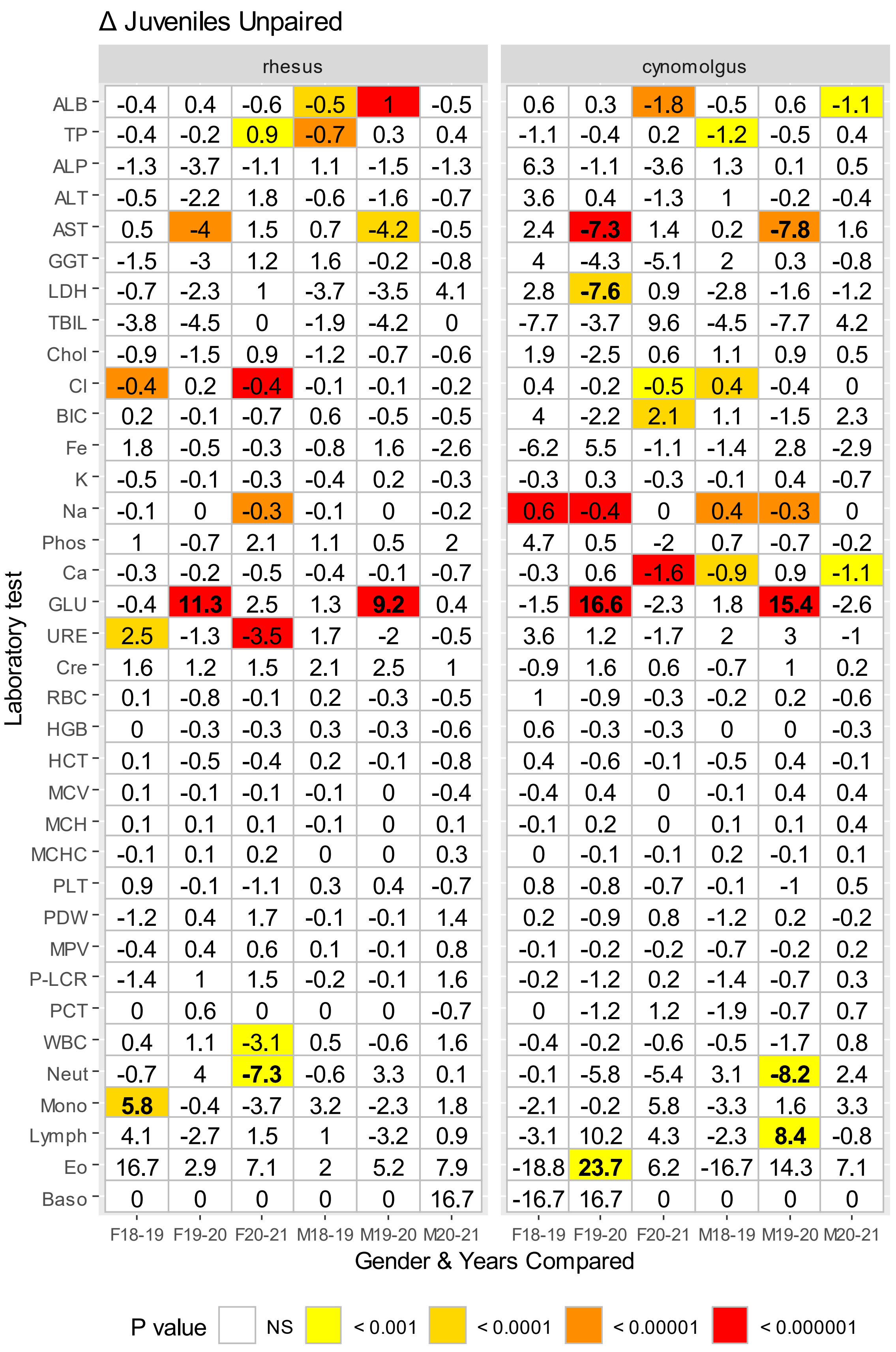

Figure S1 C. Unpaired comparisons between years in juvenile rhesus and cynomolgus macaques, numbers indicate percentual differences (Delta) between Year2 – Year1.

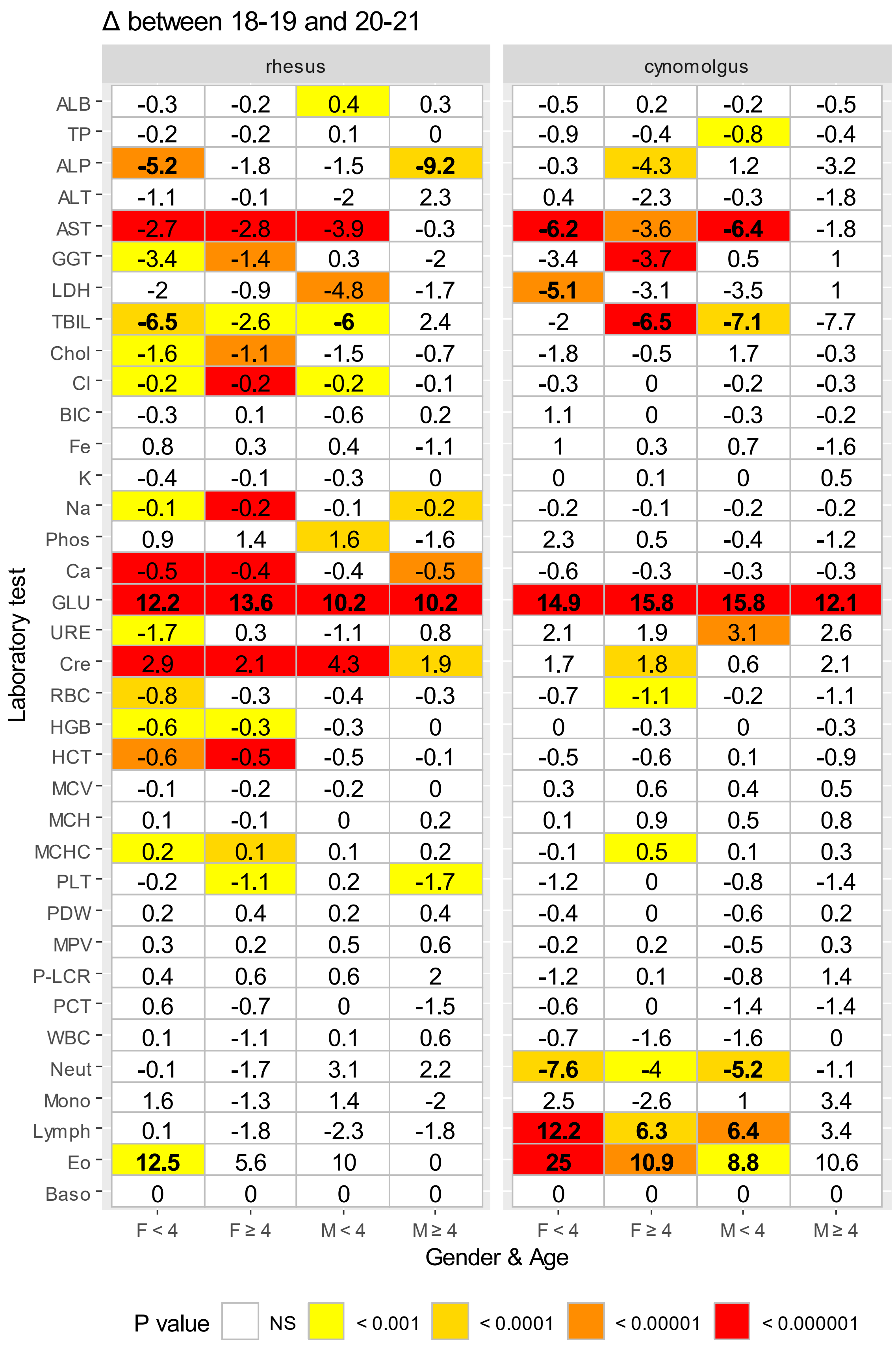

Figure S1 D. Unpaired comparisons between years in rhesus and cynomolgus macaques, numbers indicate percentual differences (Delta) between 20-21 – 18-19.
