# Supplementary material for: Reference Intervals and Percentiles for Hematologic and Serum Biochemical Values in Captive Bred Rhesus (Macaca mulatta) and Cynomolgus Macaques (Macaca fascicularis)
Source: Animals (Basel). 2023 Jan 28;13(3):445. doi: 10.3390/ani13030445 (PMC9913310; doi:10.3390/ani13030445)
Supplement: Supplementary file 1 [file animals-13-00445-s001.zip › Figure S10.pdf]

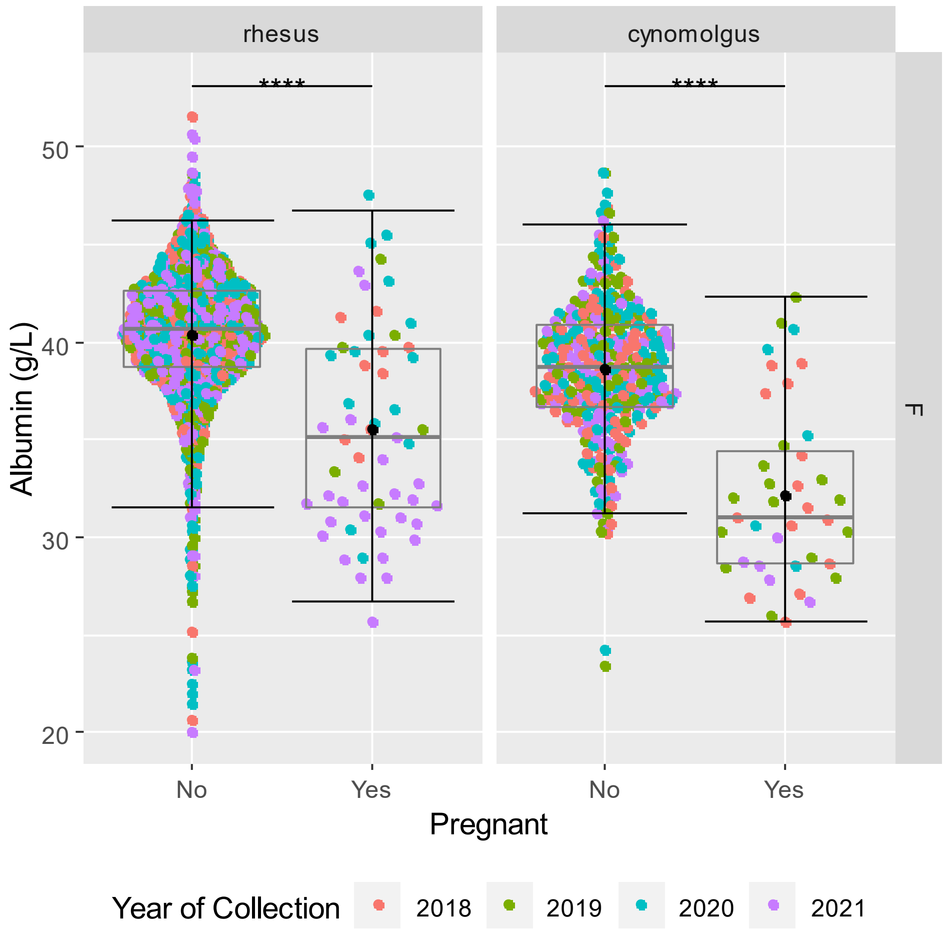

Figure S10 1. Albumin levels according to pregnancy in adult female rhesus and cynomolgus macaques. Boxes indicate median and quartile ranges, black point indicates arithmetic mean and error bars indicate the normal range.

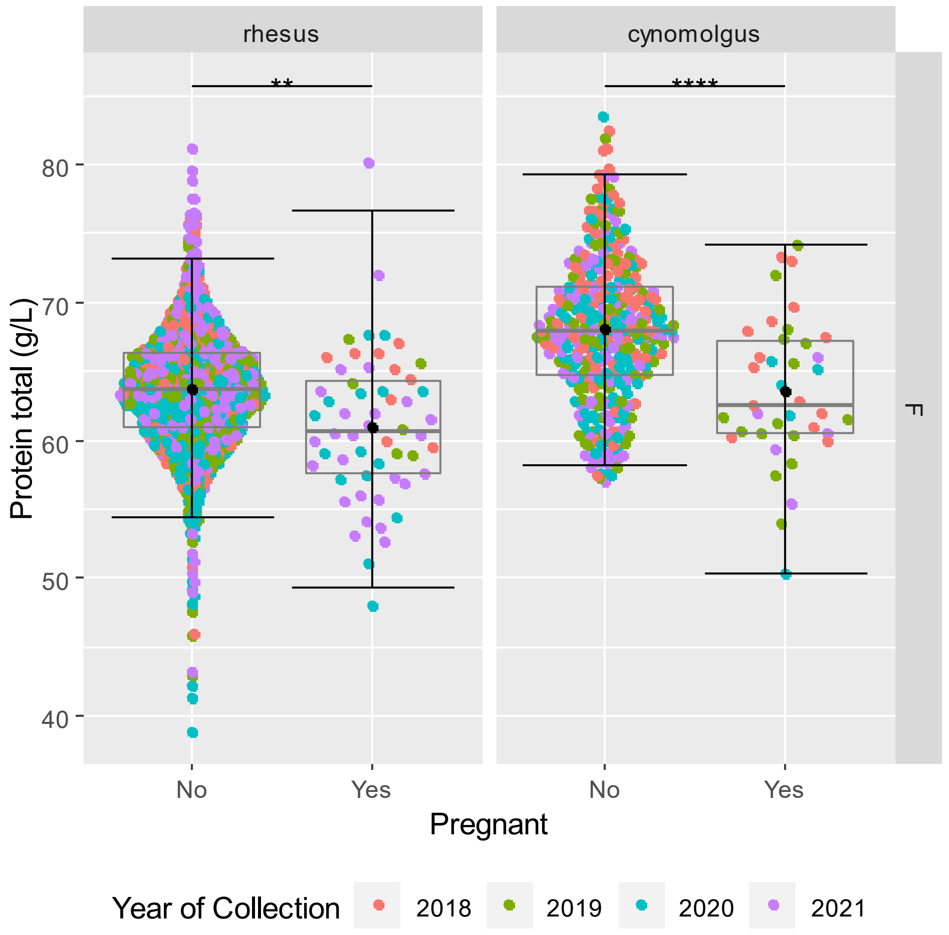

Figure S10 2. Total Protein levels according to pregnancy in adult female rhesus and cynomolgus macaques. Boxes indicate median and quartile ranges, black point indicates arithmetic mean and error bars indicate the normal range.

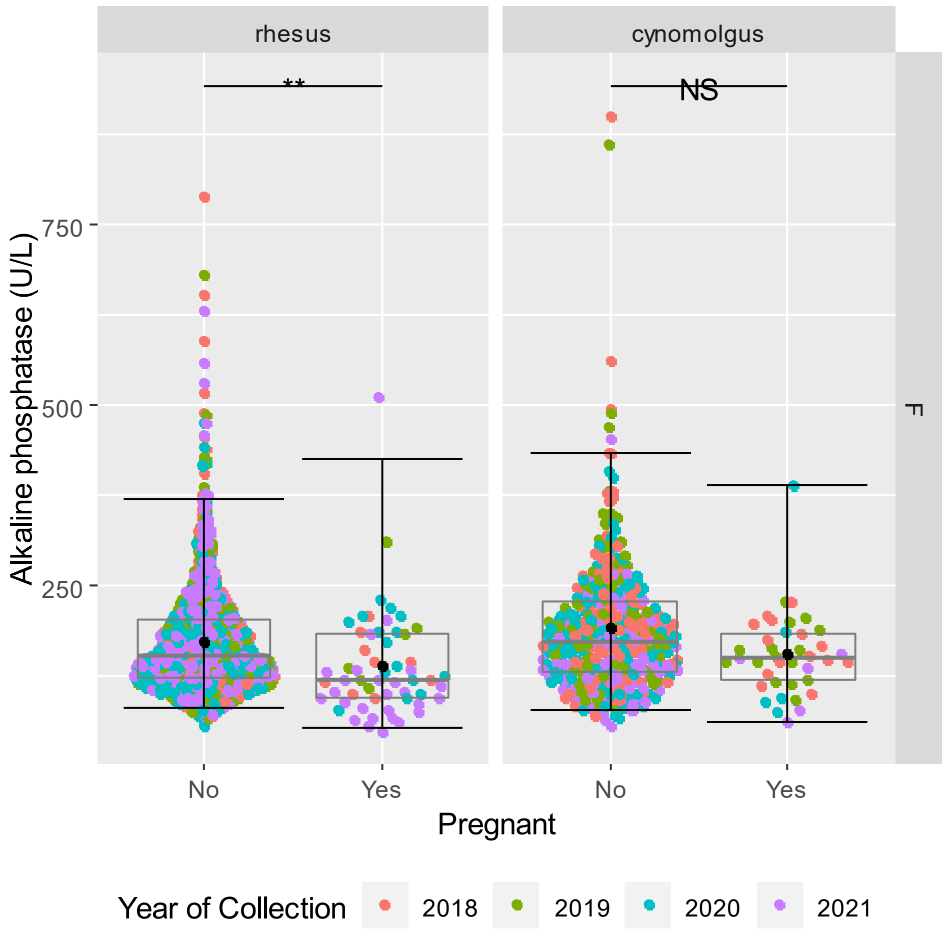

Figure S10 3. Alkaline Phosphatase levels according to period in adult female rhesus and cynomolgus macaques. Boxes indicate median and quartile ranges, black point indicates arithmetic mean and error bars indicate the normal range.

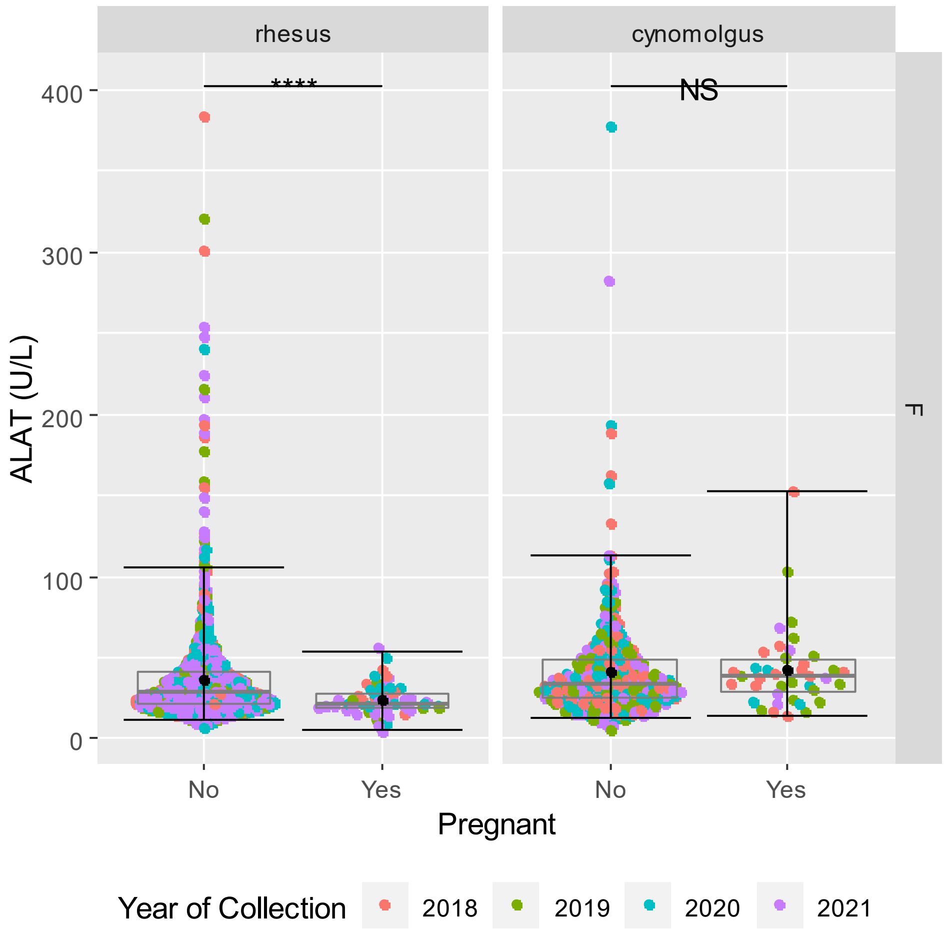

Figure S10 4. ALAT levels according to pregnancy in adult female rhesus and cynomolgus macaques. Boxes indicate median and quartile ranges, black point indicates arithmetic mean and error bars indicate the normal range.

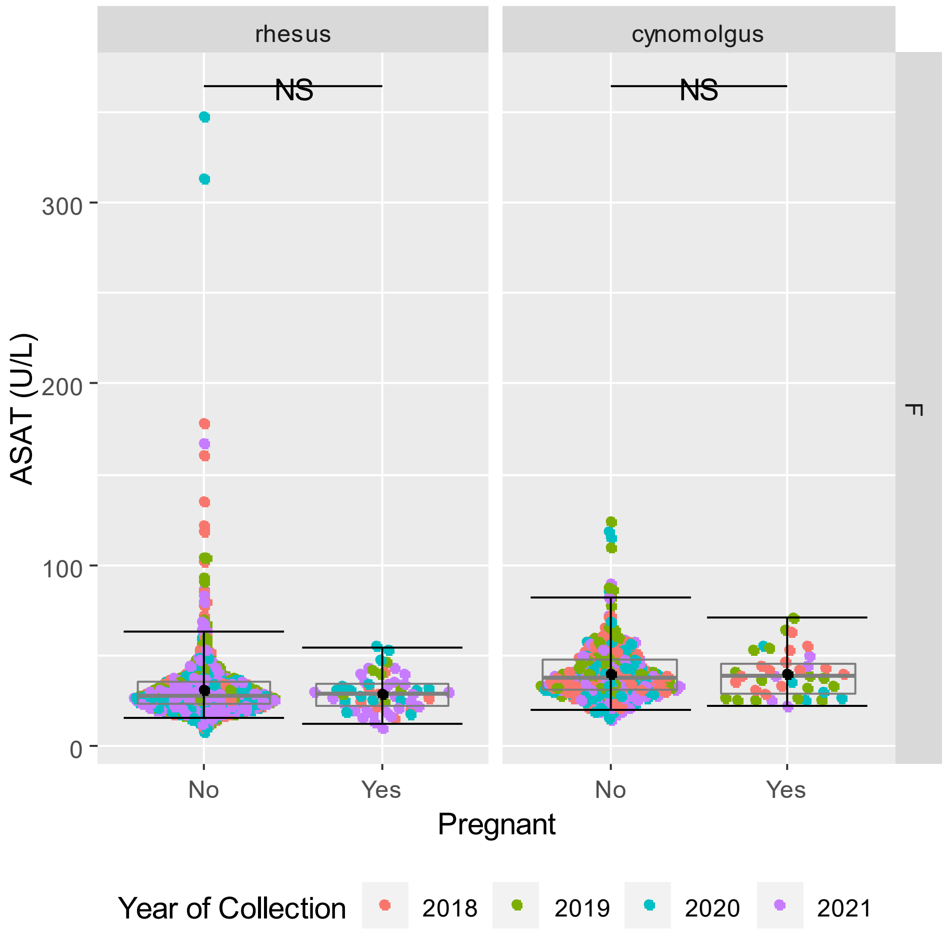

Figure S10 5. ASAT levels according to pregnancy in adult female rhesus and cynomolgus macaques. Boxes indicate median and quartile ranges, black point indicates arithmetic mean and error bars indicate the normal range.

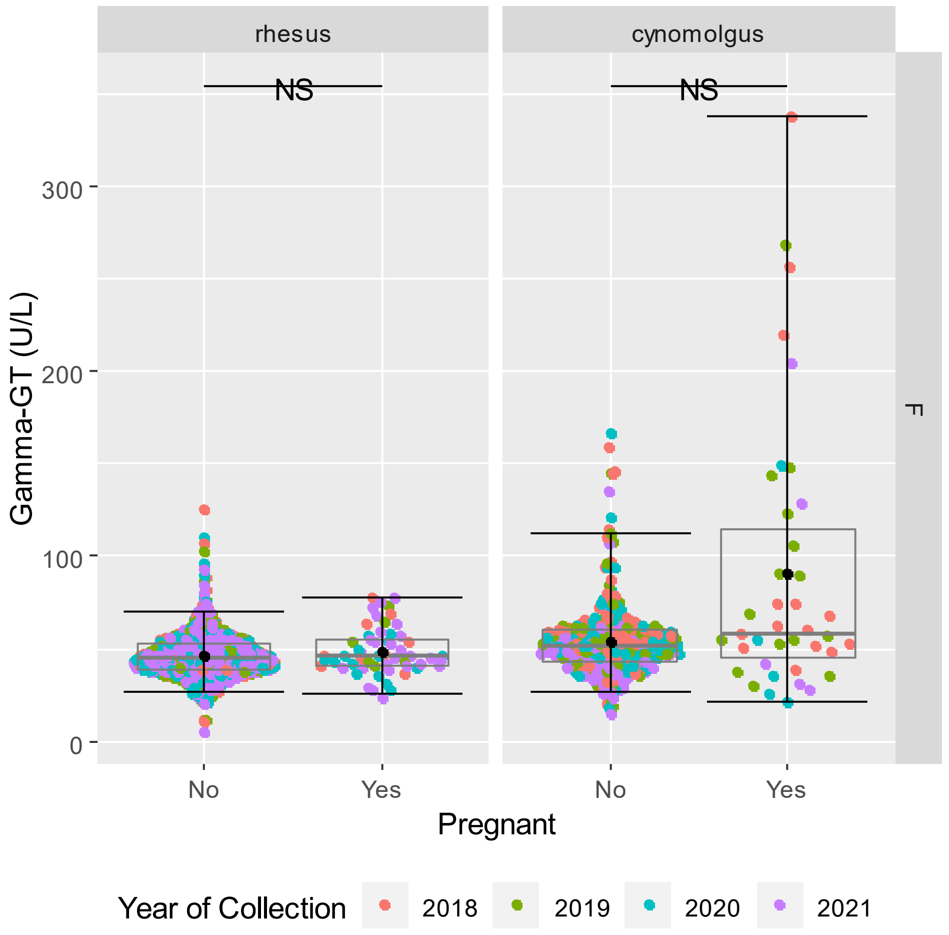

Figure S10 6. Gamma-GT levels according to pregnancy in adult female rhesus and cynomolgus macaques. Boxes indicate median and quartile ranges, black point indicates arithmetic mean and error bars indicate the normal range.

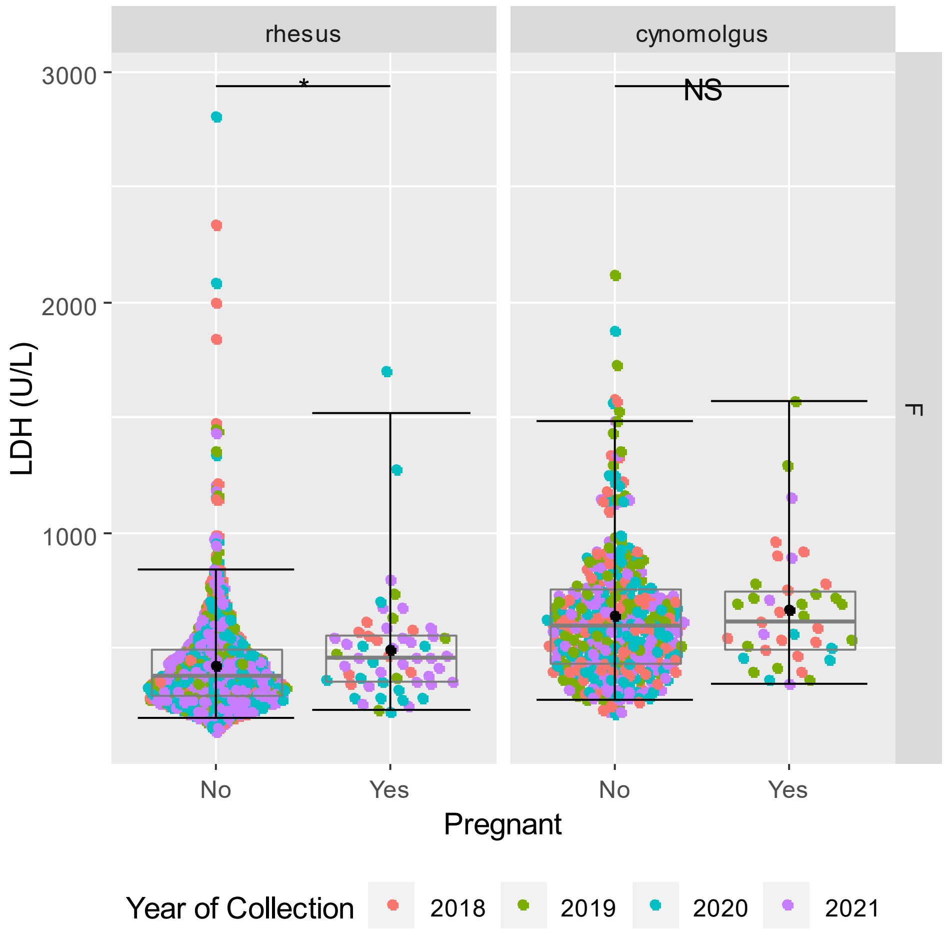

Figure S10 7. LDH levels according to pregnancy in adult female rhesus and cynomolgus macaques. Boxes indicate median and quartile ranges, black point indicates arithmetic mean and error bars indicate the normal range.

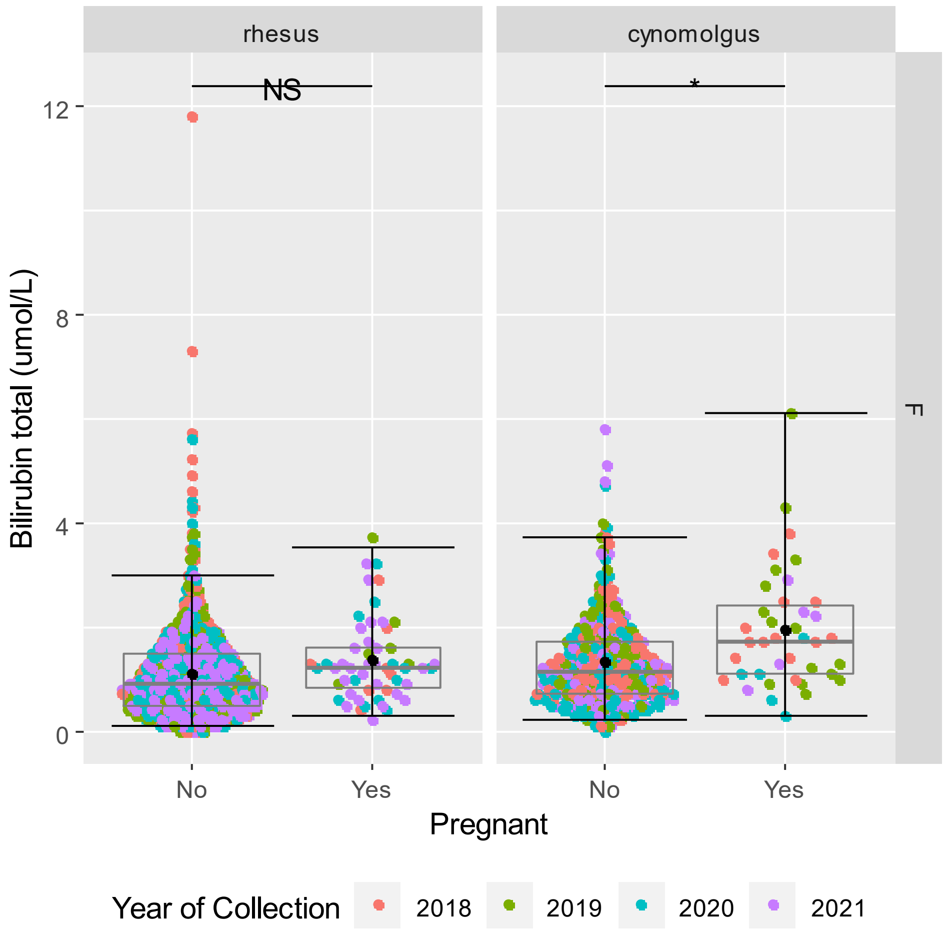

Figure S10 8. Total Bilirubin levels according to pregnancy in adult female rhesus and cynomolgus macaques. Boxes indicate median and quartile ranges, black point indicates arithmetic mean and error bars indicate the normal range.

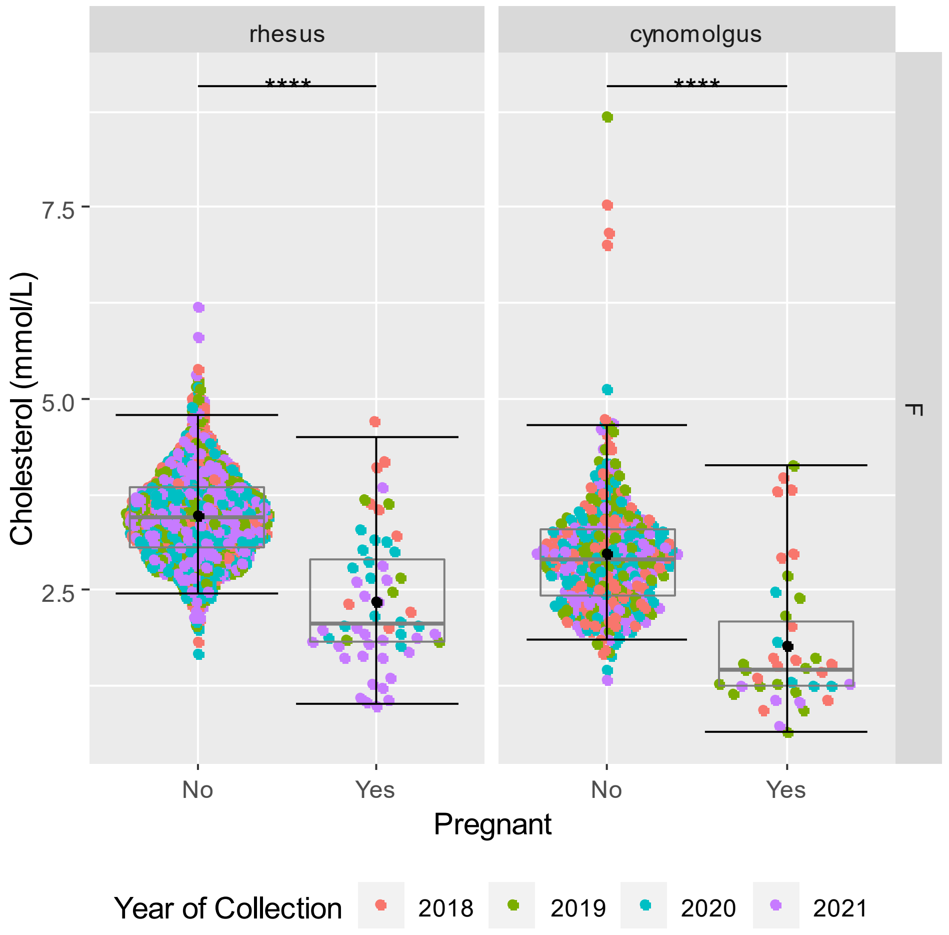

Figure S10 9. Cholesterol levels according to pregnancy in adult female rhesus and cynomolgus macaques. Boxes indicate median and quartile ranges, black point indicates arithmetic mean and error bars indicate the normal range.

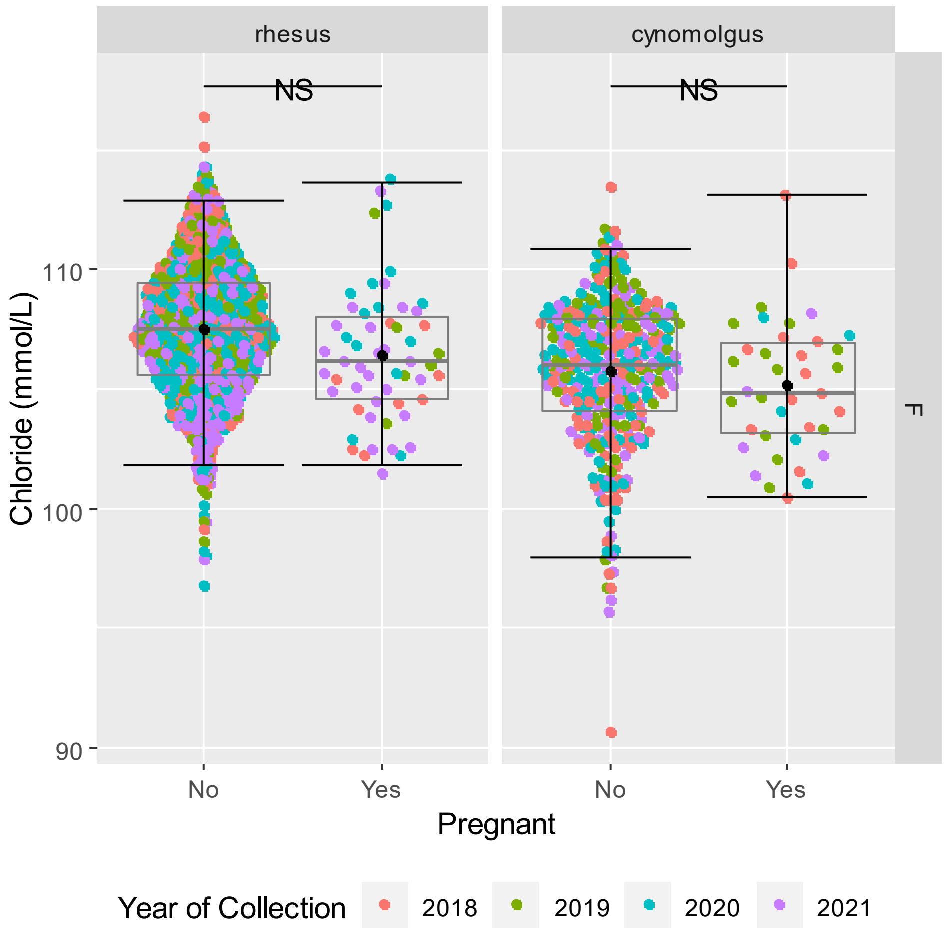

Figure S10 10. Chloride levels according to pregnancy in adult female rhesus and cynomolgus macaques. Boxes indicate median and quartile ranges, black point indicates arithmetic mean and error bars indicate the normal range.

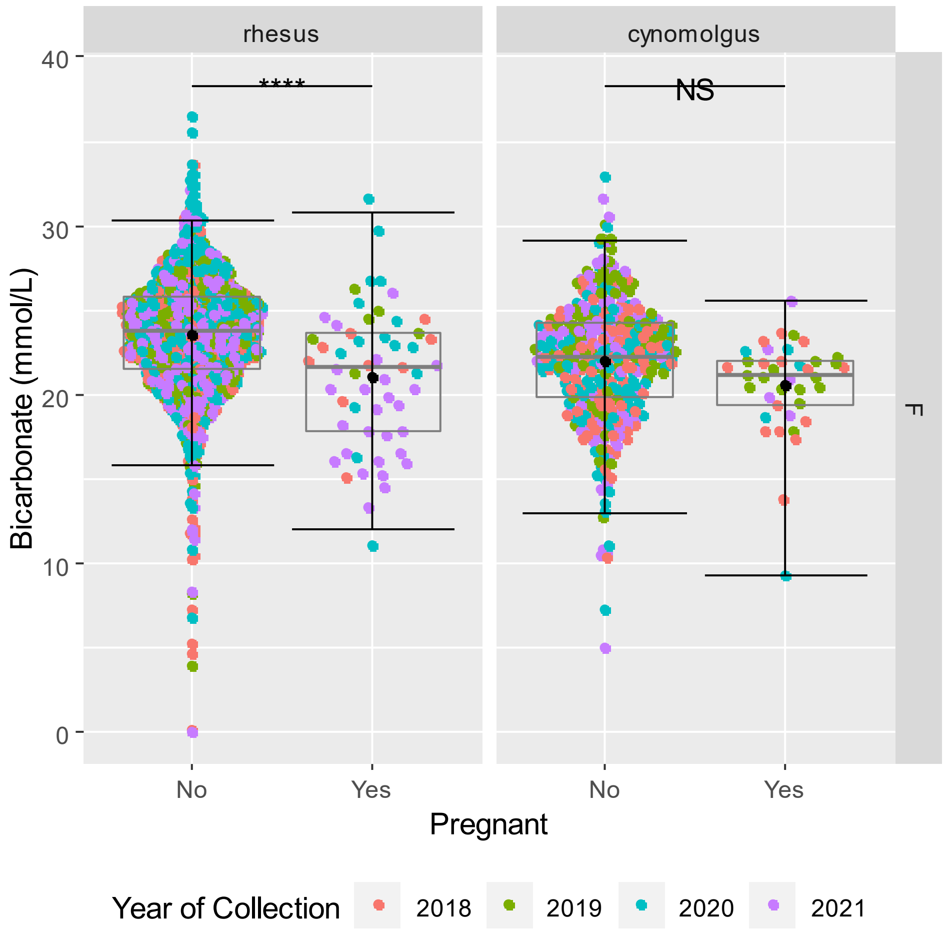

Figure S10 11. Bicarbonate levels according to pregnancy in adult female rhesus and cynomolgus macaques. Boxes indicate median and quartile ranges, black point indicates arithmetic mean and error bars indicate the normal range.

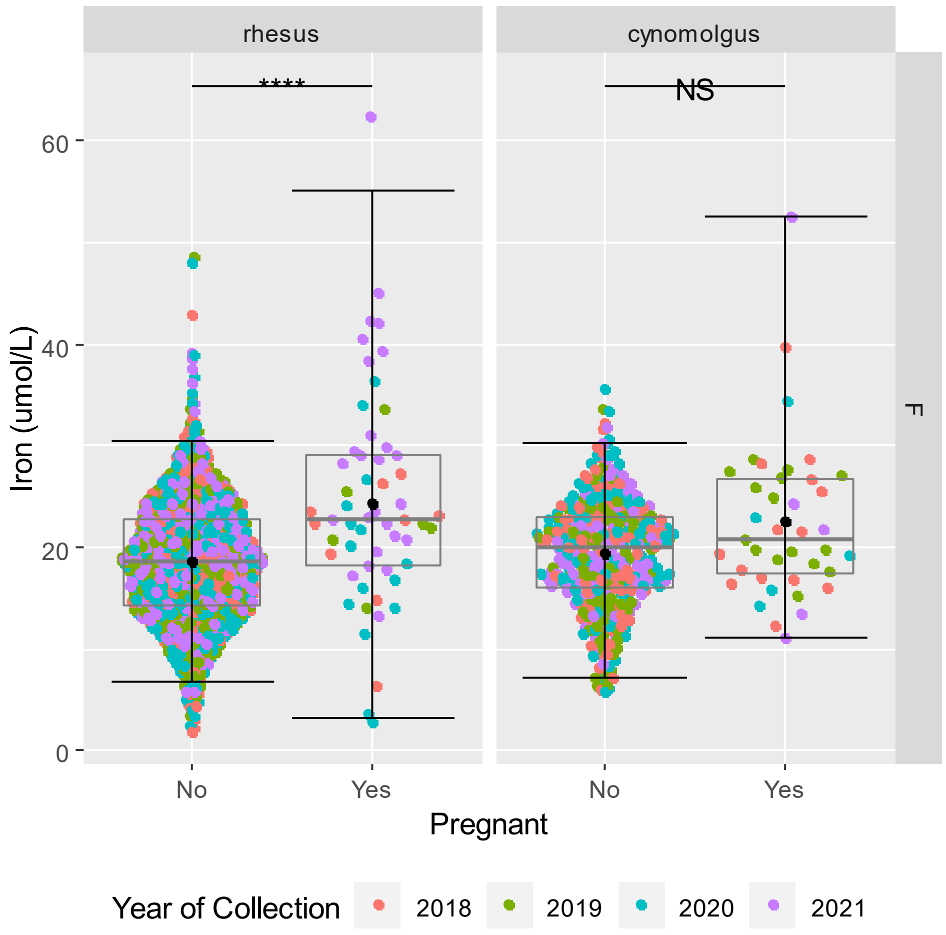

Figure S10 12. Iron levels according to pregnancy in adult female rhesus and cynomolgus macaques. Boxes indicate median and quartile ranges, black point indicates arithmetic mean and error bars indicate the normal range.

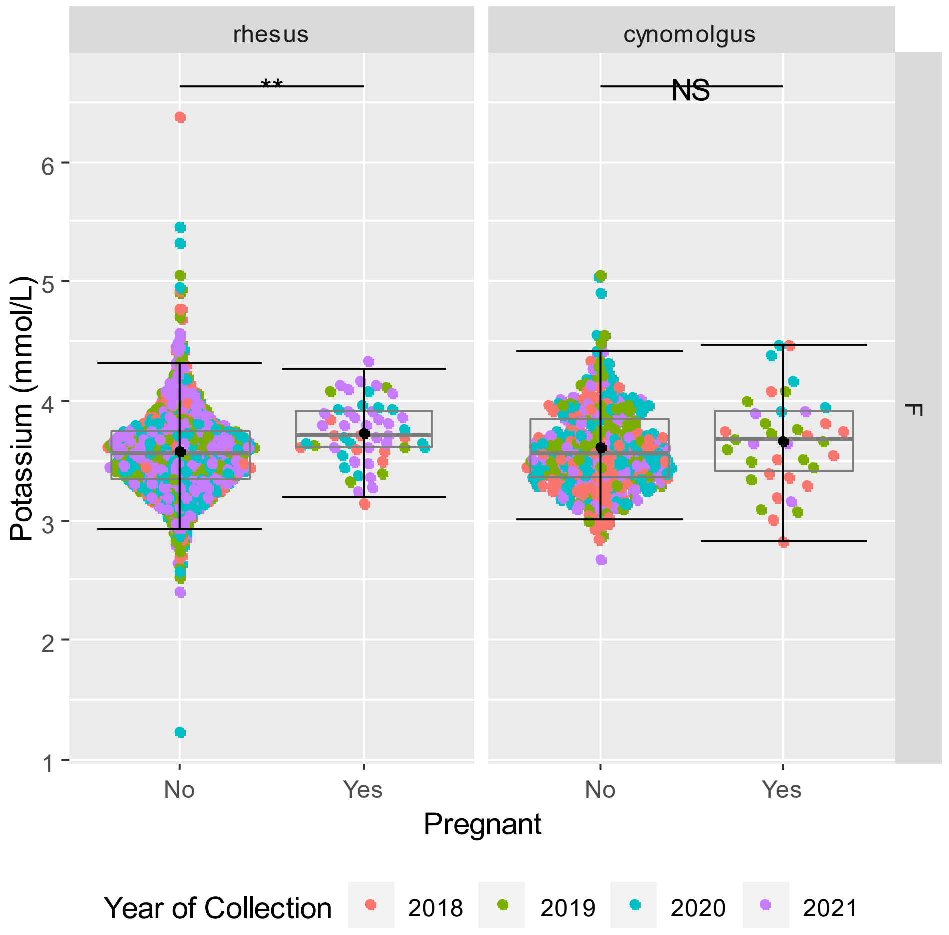

Figure S10 13. Potassium levels according to pregnancy in adult female rhesus and cynomolgus macaques. Boxes indicate median and quartile ranges, black point indicates arithmetic mean and error bars indicate the normal range.

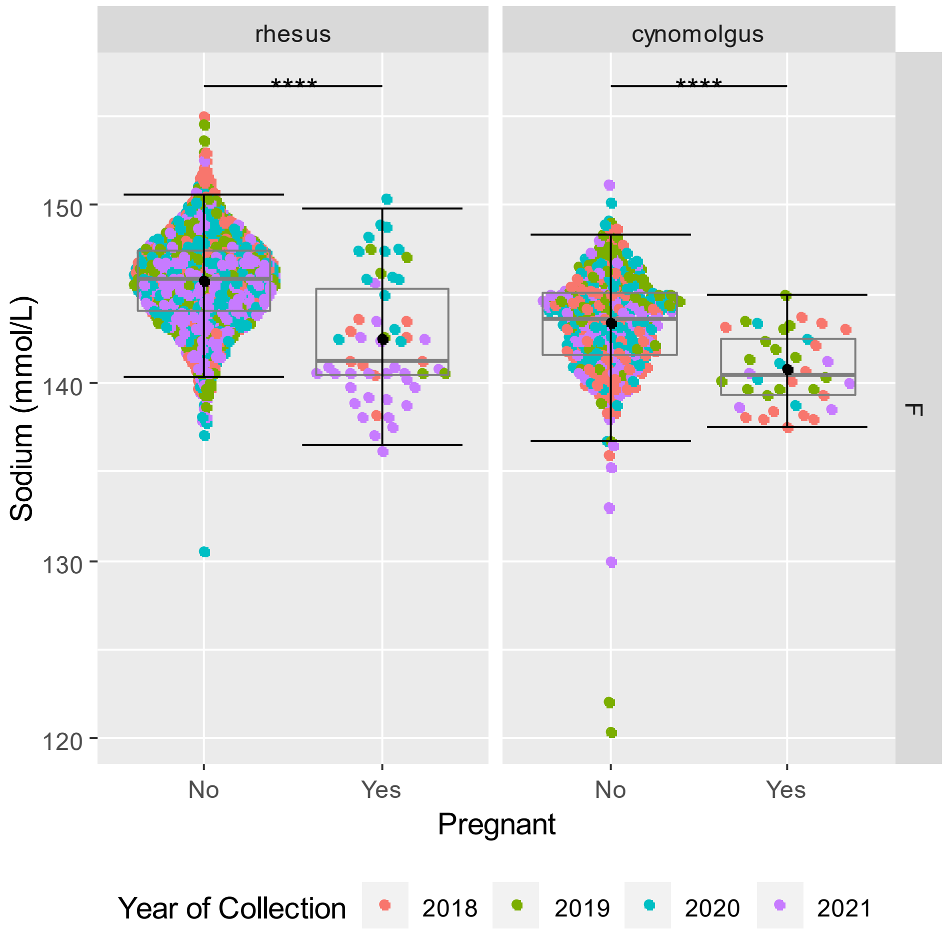

Figure S10 14. Sodium levels according to pregnancy in adult female rhesus and cynomolgus macaques. Boxes indicate median and quartile ranges, black point indicates arithmetic mean and error bars indicate the normal range.

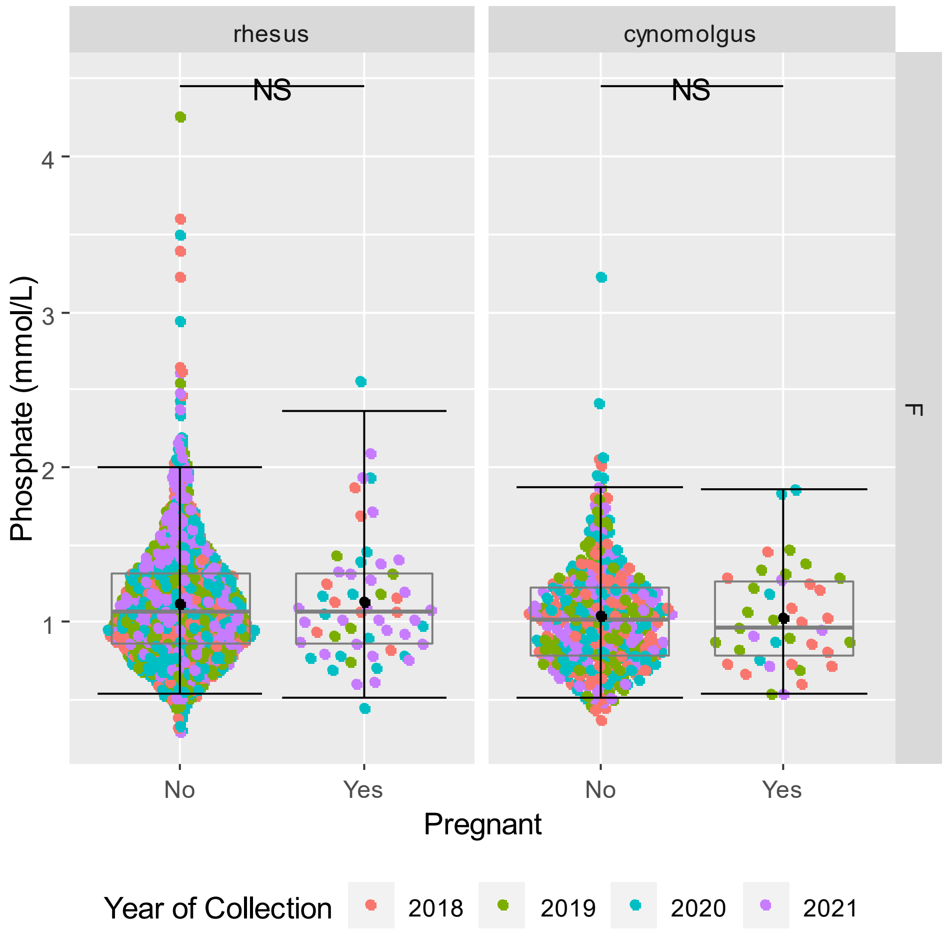

Figure S10 15. Phosphate levels according to pregnancy in adult female rhesus and cynomolgus macaques. Boxes indicate median and quartile ranges, black point indicates arithmetic mean and error bars indicate the normal range.

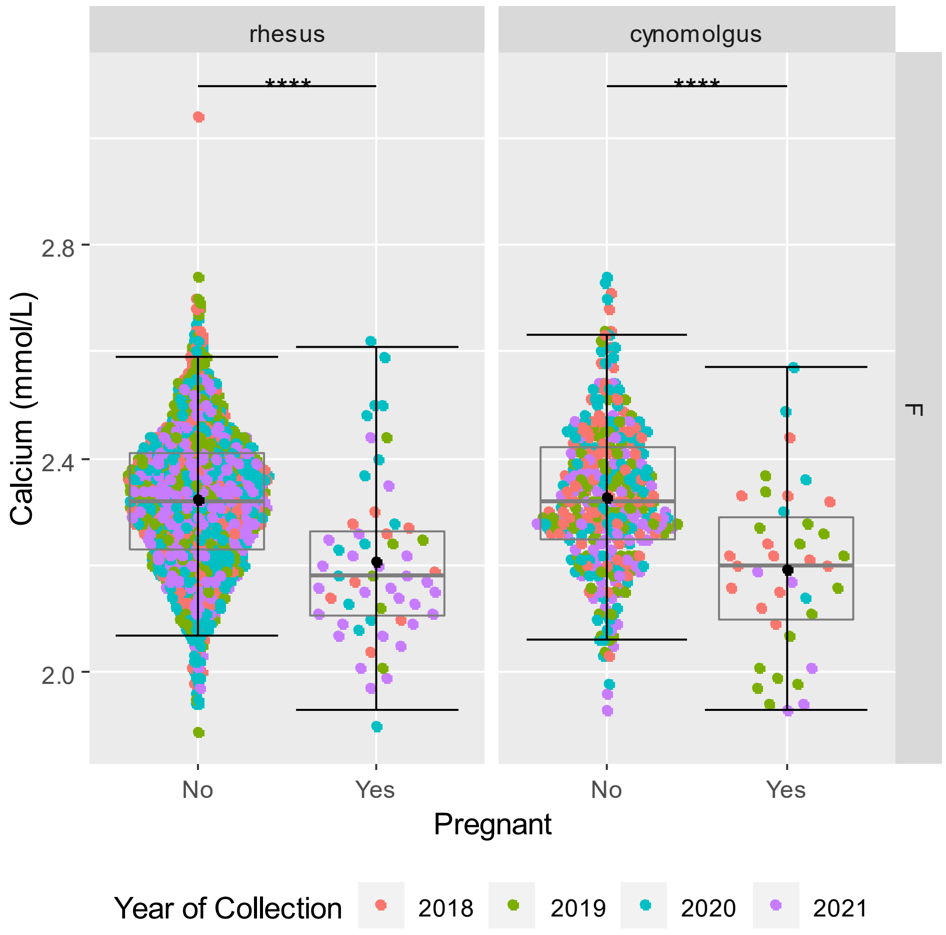

Figure S10 16. Calcium levels according to pregnancy in adult female rhesus and cynomolgus macaques. Boxes indicate median and quartile ranges, black point indicates arithmetic mean and error bars indicate the normal range.

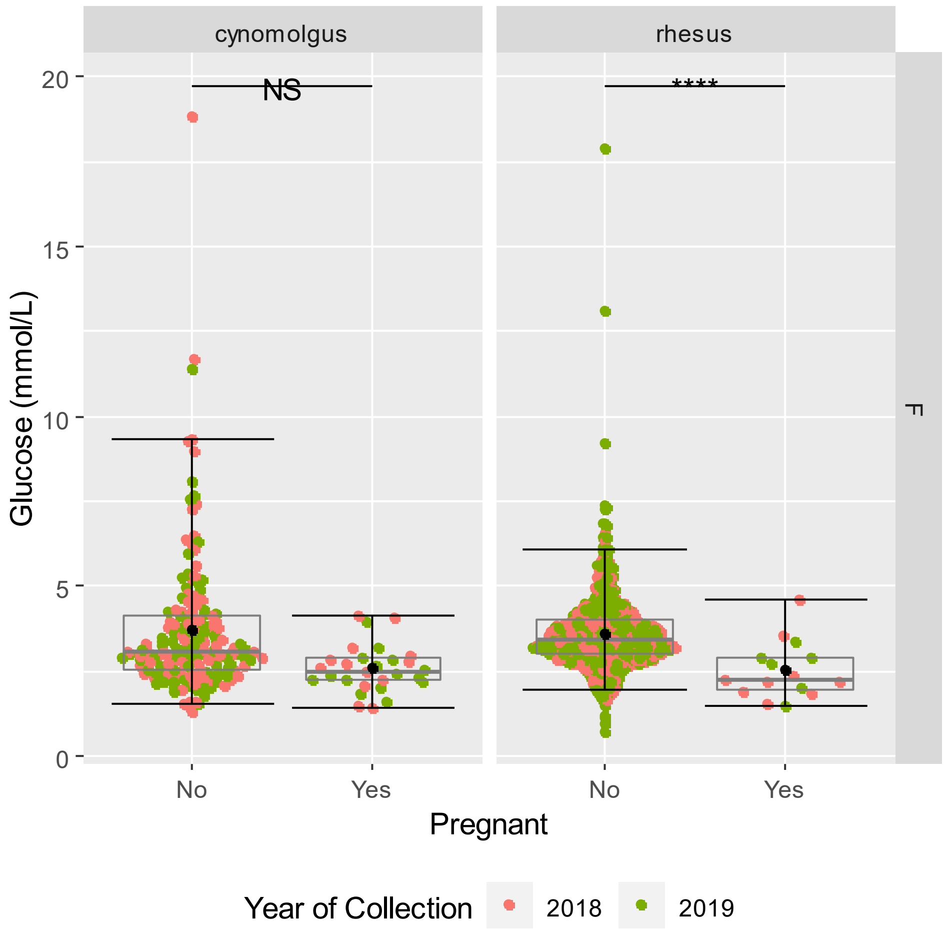

Figure S10 17A. Glucose levels in 2018-2019 according to pregnancy in adult female rhesus and cynomolgus macaques. Boxes indicate median and quartile ranges, black point indicates arithmetic mean and error bars indicate the normal range.

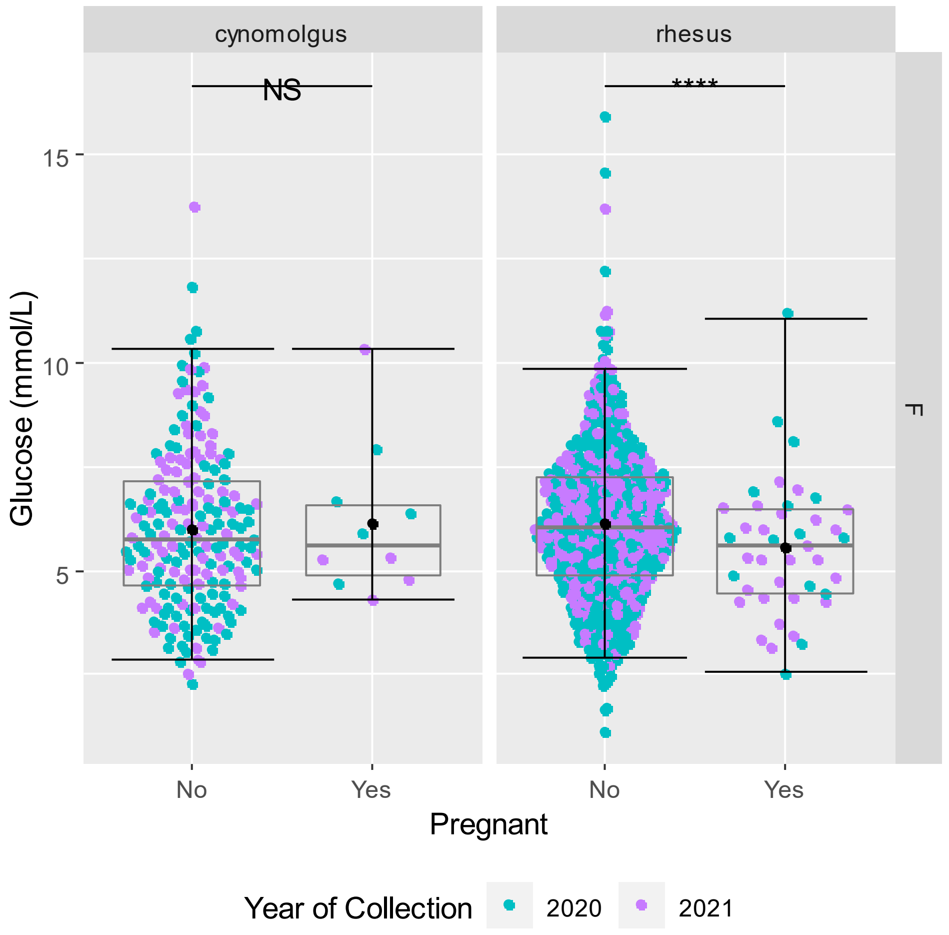

Figure S10 17B. Glucose levels in 2020-2021 according to pregnancy in adult female rhesus and cynomolgus macaques. Boxes indicate median and quartile ranges, black point indicates arithmetic mean and error bars indicate the normal range.

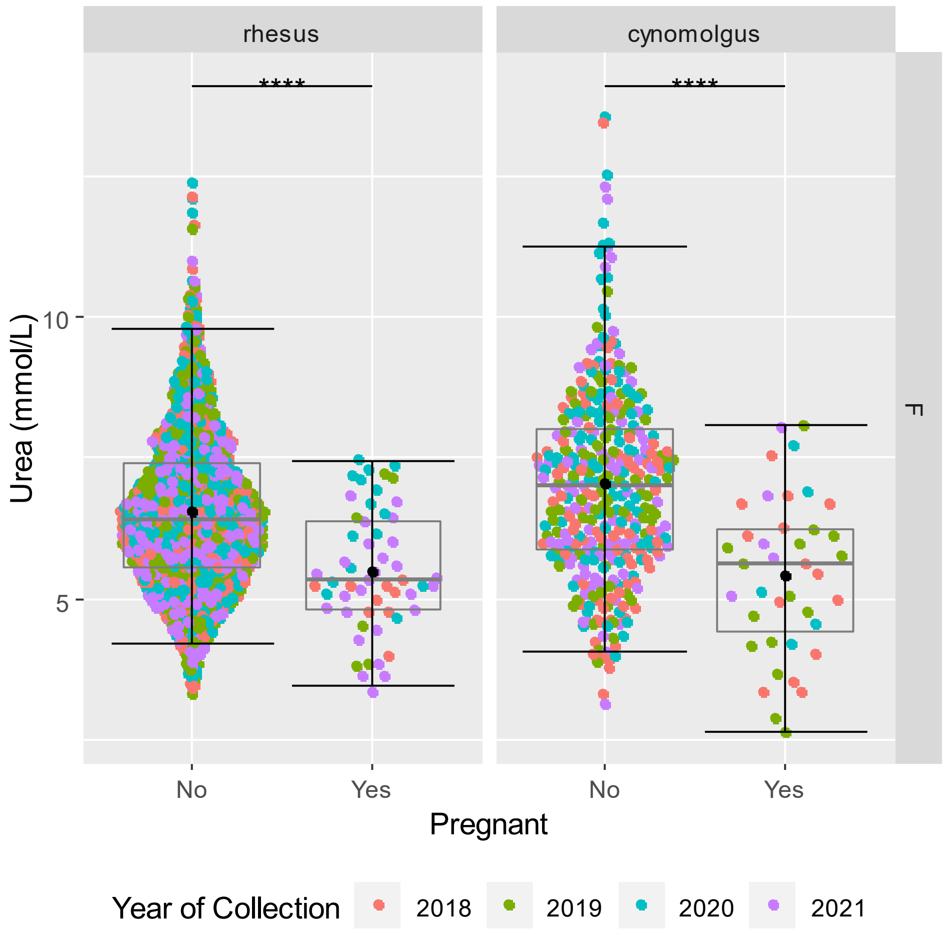

Figure S10 18. Urea levels according to pregnancy in adult female rhesus and cynomolgus macaques. Boxes indicate median and quartile ranges, black point indicates arithmetic mean and error bars indicate the normal range.

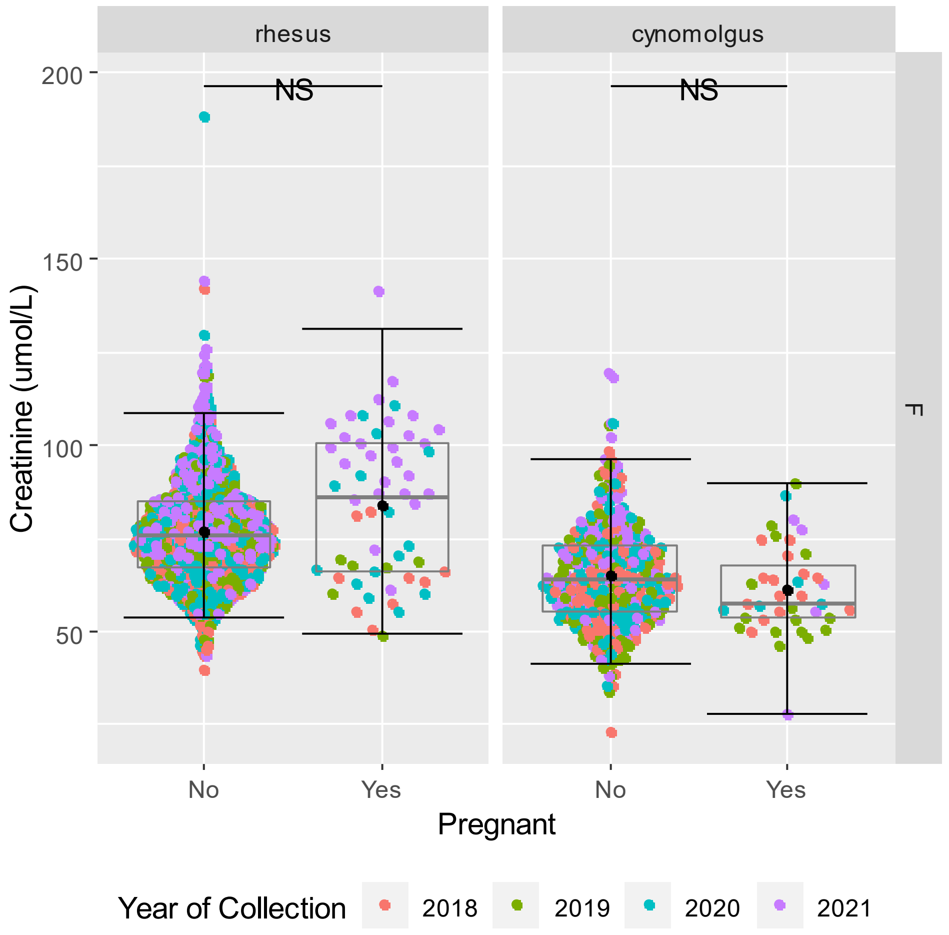

Figure S10 19. Creatinine levels according to pregnancy in adult female rhesus and cynomolgus macaques. Boxes indicate median and quartile ranges, black point indicates arithmetic mean and error bars indicate the normal range.

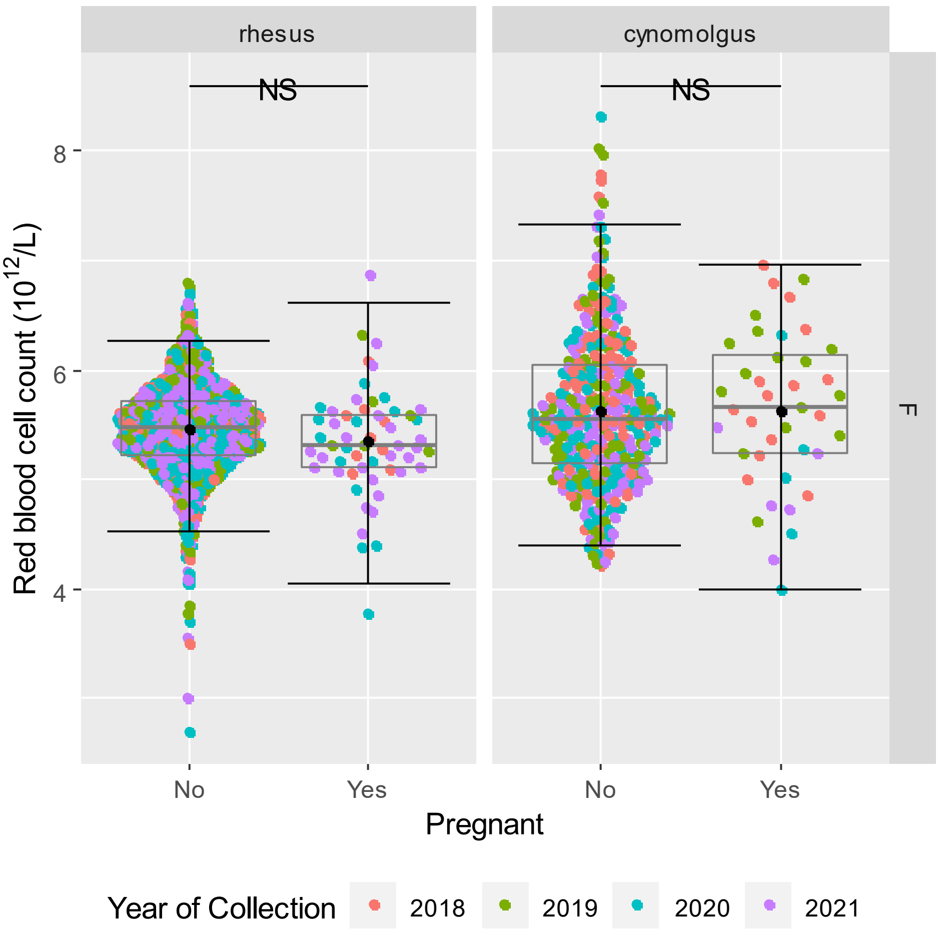

Figure S10 20. Red Blood Cell count according to pregnancy in adult female rhesus and cynomolgus macaques. Boxes indicate median and quartile ranges, black point indicates arithmetic mean and error bars indicate the normal range.

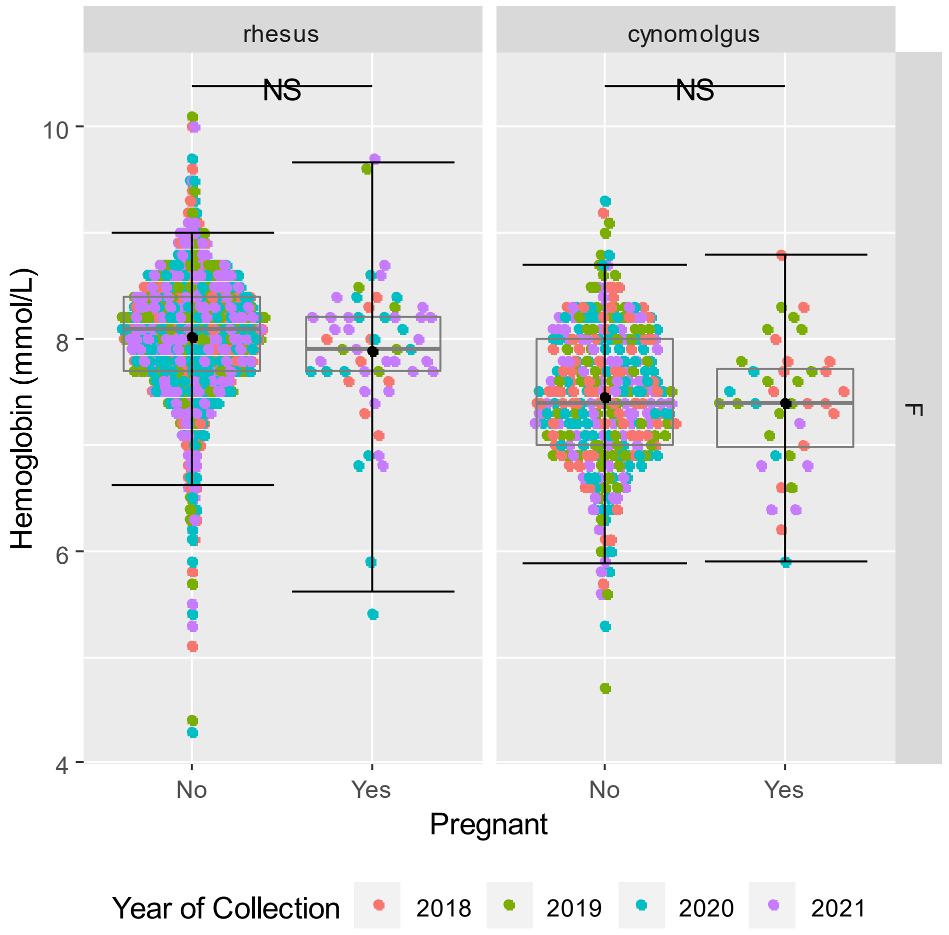

Figure S10 21. Hemoglobin levels according to pregnancy in adult female rhesus and cynomolgus macaques. Boxes indicate median and quartile ranges, black point indicates arithmetic mean and error bars indicate the normal range.

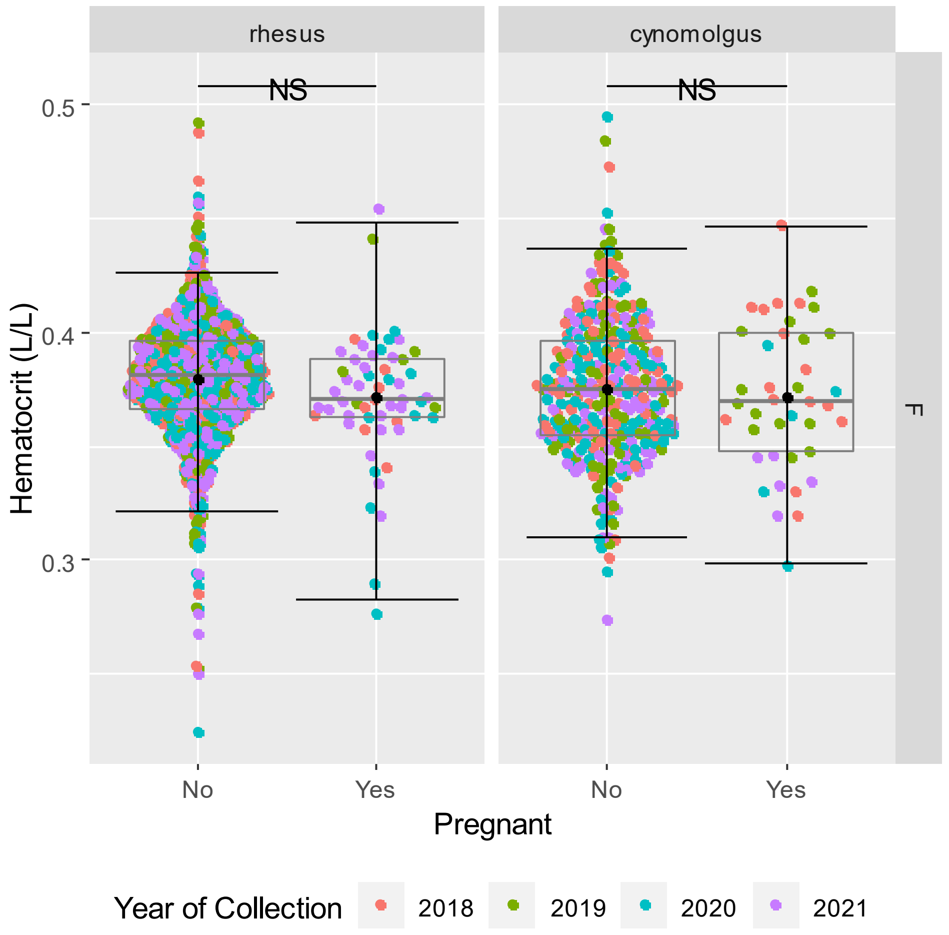

Figure S10 22. Hematocrit levels according to pregnancy in adult female rhesus and cynomolgus macaques. Boxes indicate median and quartile ranges, black point indicates arithmetic mean and error bars indicate the normal range.

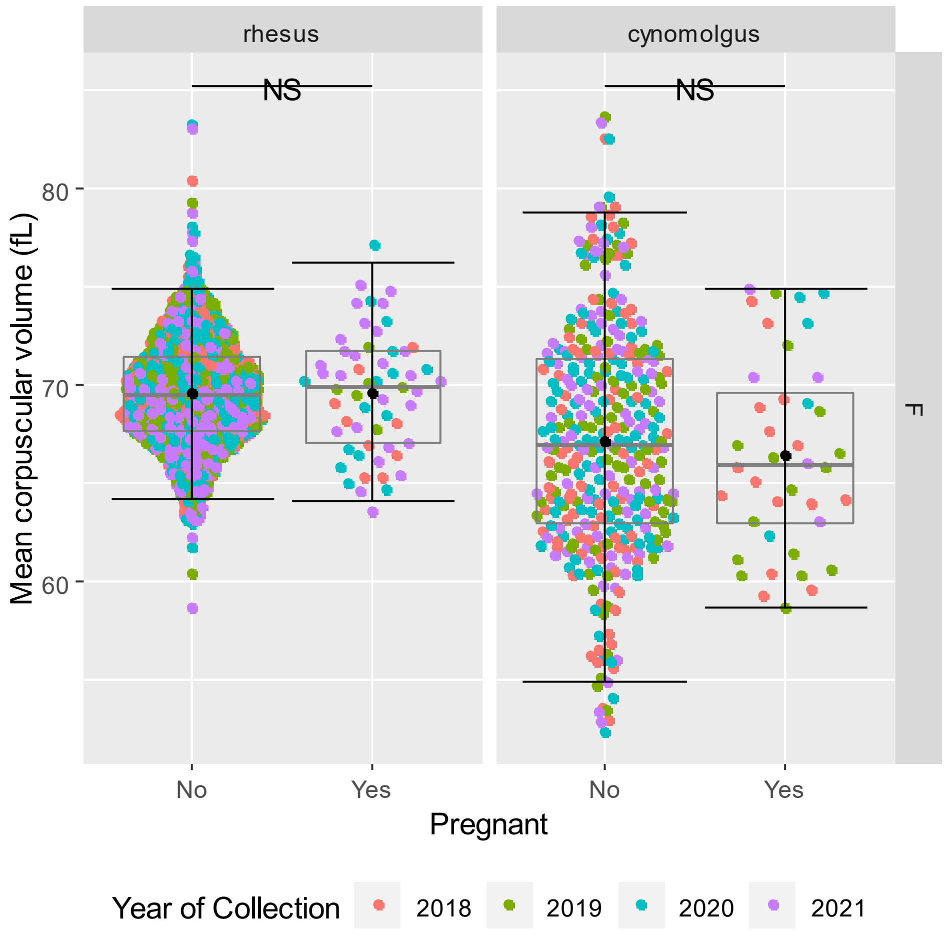

Figure S10 23. Mean Corpuscular Volume according to pregnancy in adult female rhesus and cynomolgus macaques. Boxes indicate median and quartile ranges, black point indicates arithmetic mean and error bars indicate the normal range.

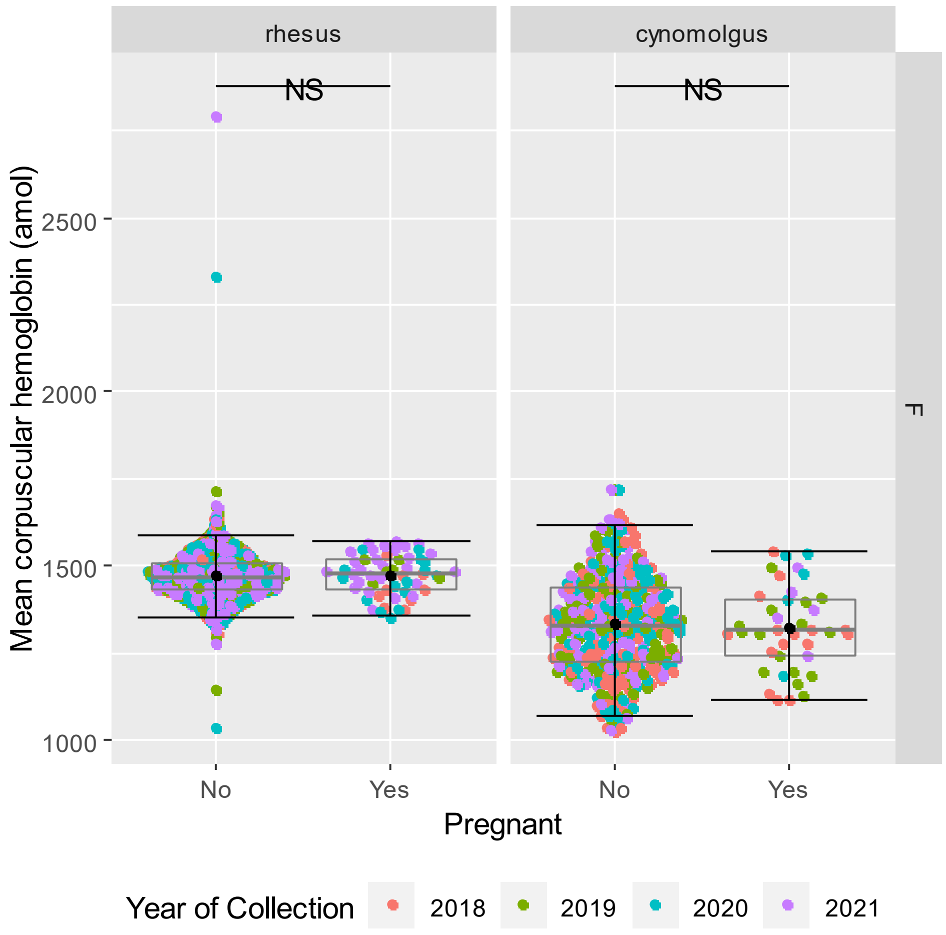

Figure S10 24. Mean Corpuscular Hemoglobin according to pregnancy in adult female rhesus and cynomolgus macaques. Boxes indicate median and quartile ranges, black point indicates arithmetic mean and error bars indicate the normal range.

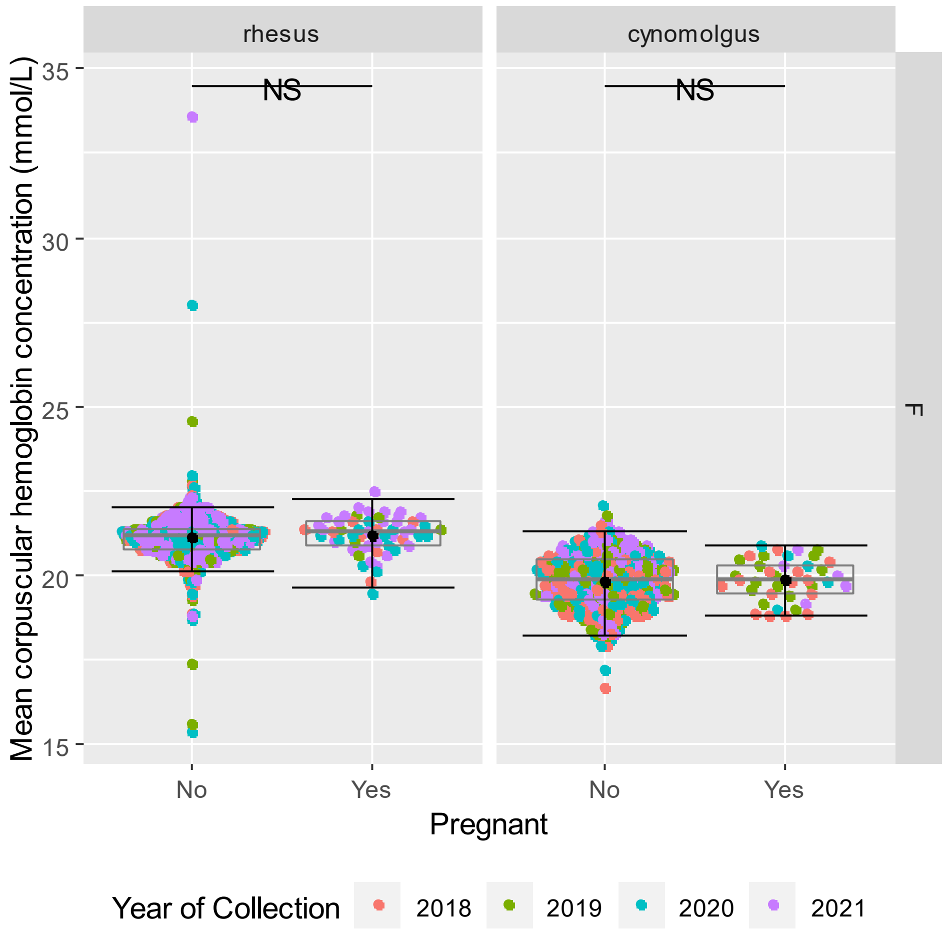

Figure S10 25. Mean Corpuscular Hemoglobin Concentration according to pregnancy in adult female rhesus and cynomolgus macaques. Boxes indicate median and quartile ranges, black point indicates arithmetic mean and error bars indicate the normal range.

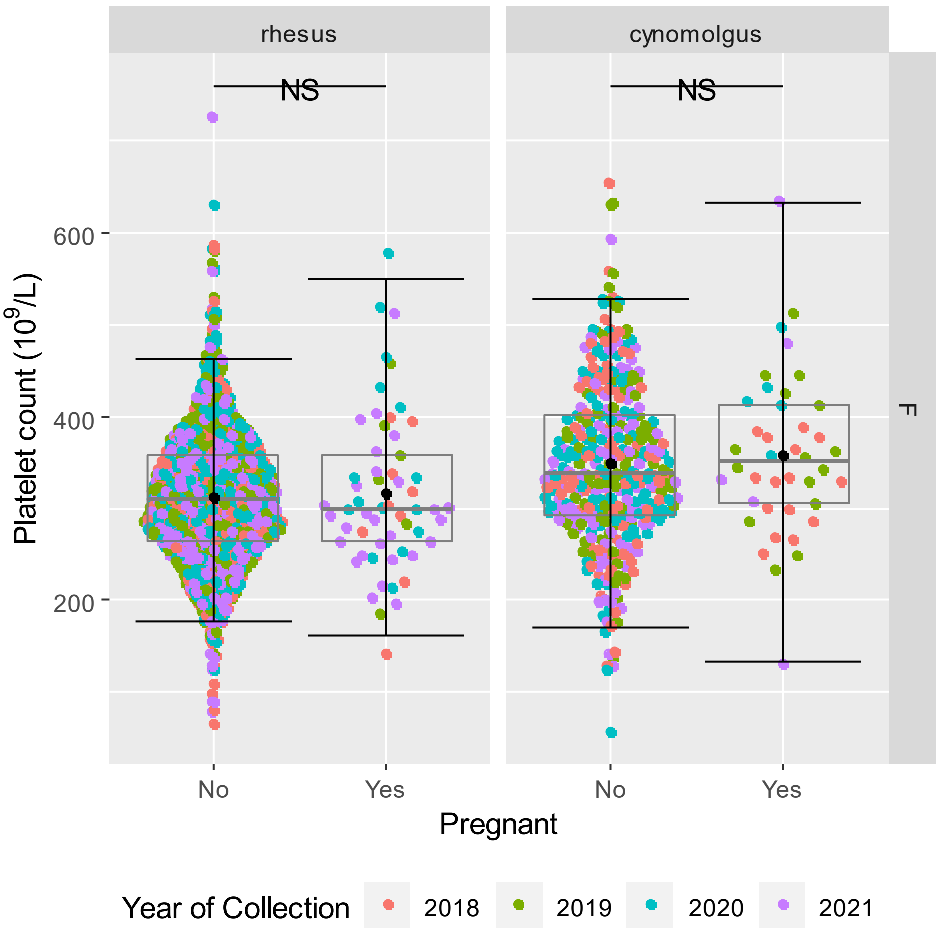

Figure S10 26. Platelet Count according to pregnancy in adult female rhesus and cynomolgus macaques. Boxes indicate median and quartile ranges, black point indicates arithmetic mean and error bars indicate the normal range.

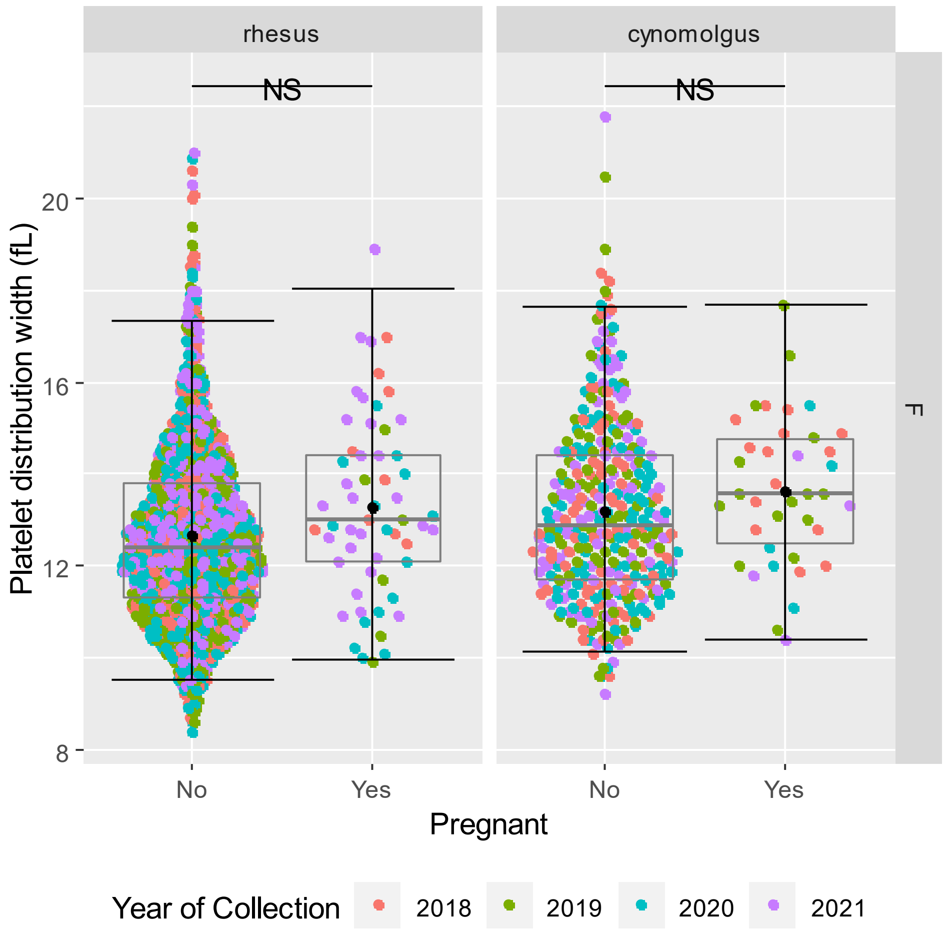

Figure S10 27. Platelet Distribution Width according to pregnancy in adult female rhesus and cynomolgus macaques. Boxes indicate median and quartile ranges, black point indicates arithmetic mean and error bars indicate the normal range.

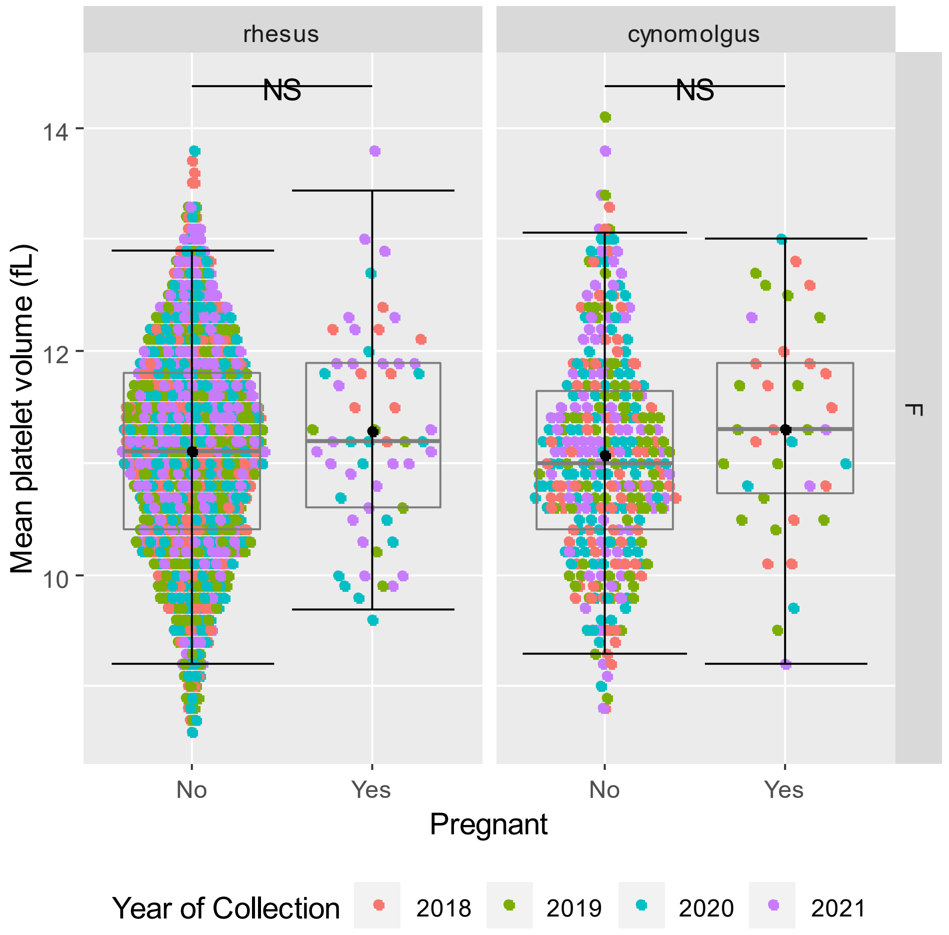

Figure S10 28. Mean Platelet Volume according to pregnancy in adult female rhesus and cynomolgus macaques. Boxes indicate median and quartile ranges, black point indicates arithmetic mean and error bars indicate the normal range.

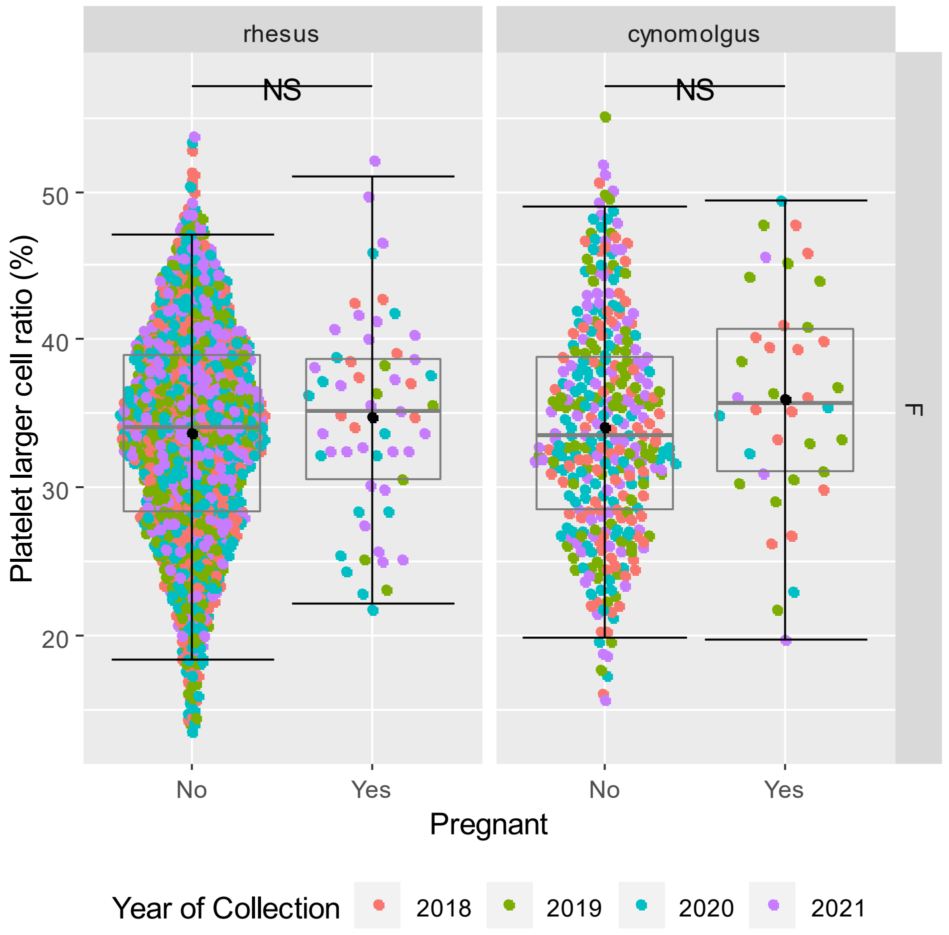

Figure S10 29. Platelet Larger Cell Ratio according to pregnancy in adult female rhesus and cynomolgus macaques. Boxes indicate median and quartile ranges, black point indicates arithmetic mean and error bars indicate the normal range.

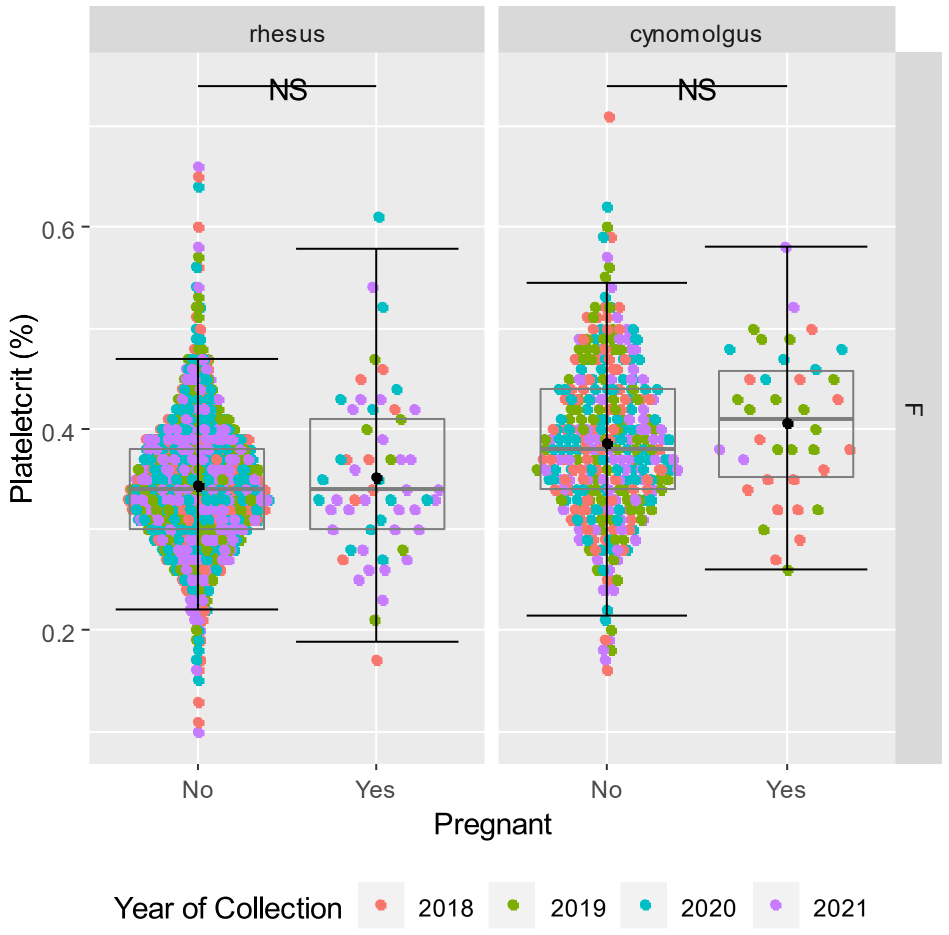

Figure S10 30. Plateletcrit according to pregnancy in adult female rhesus and cynomolgus macaques. Boxes indicate median and quartile ranges, black point indicates arithmetic mean and error bars indicate the normal range.

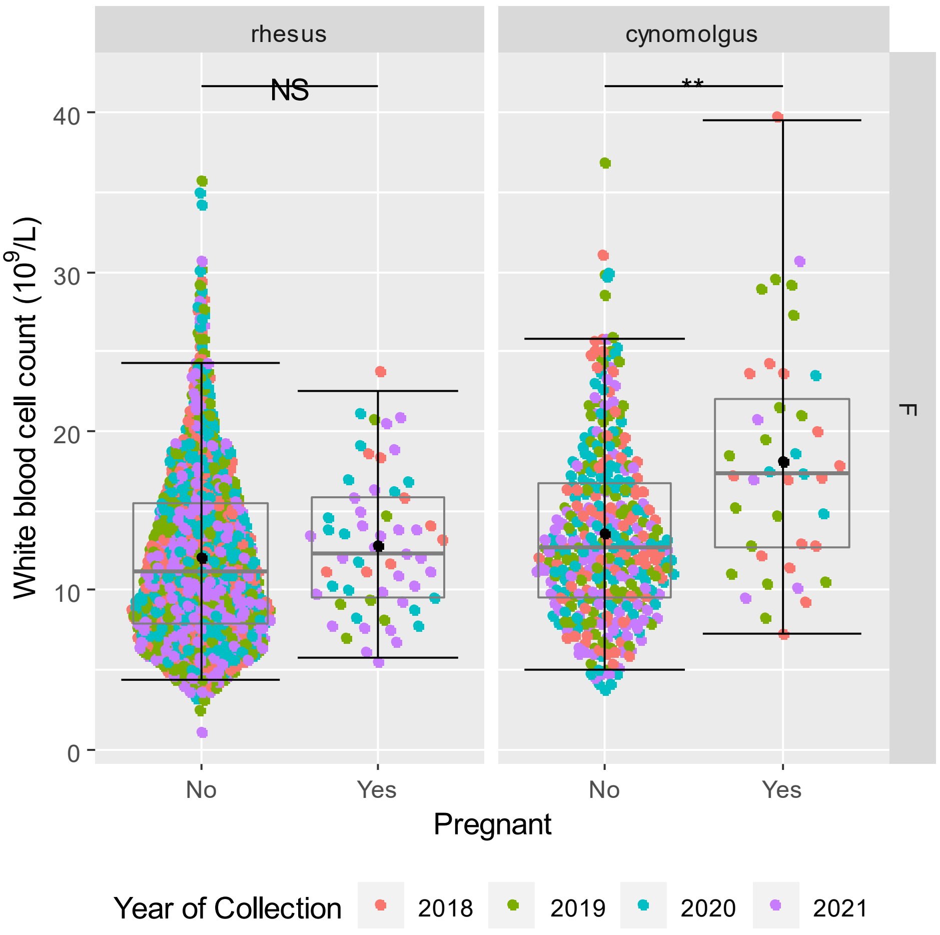

Figure S10 31. White Blood Cell Count according to pregnancy in adult female rhesus and cynomolgus macaques. Boxes indicate median and quartile ranges, black point indicates arithmetic mean and error bars indicate the normal range.

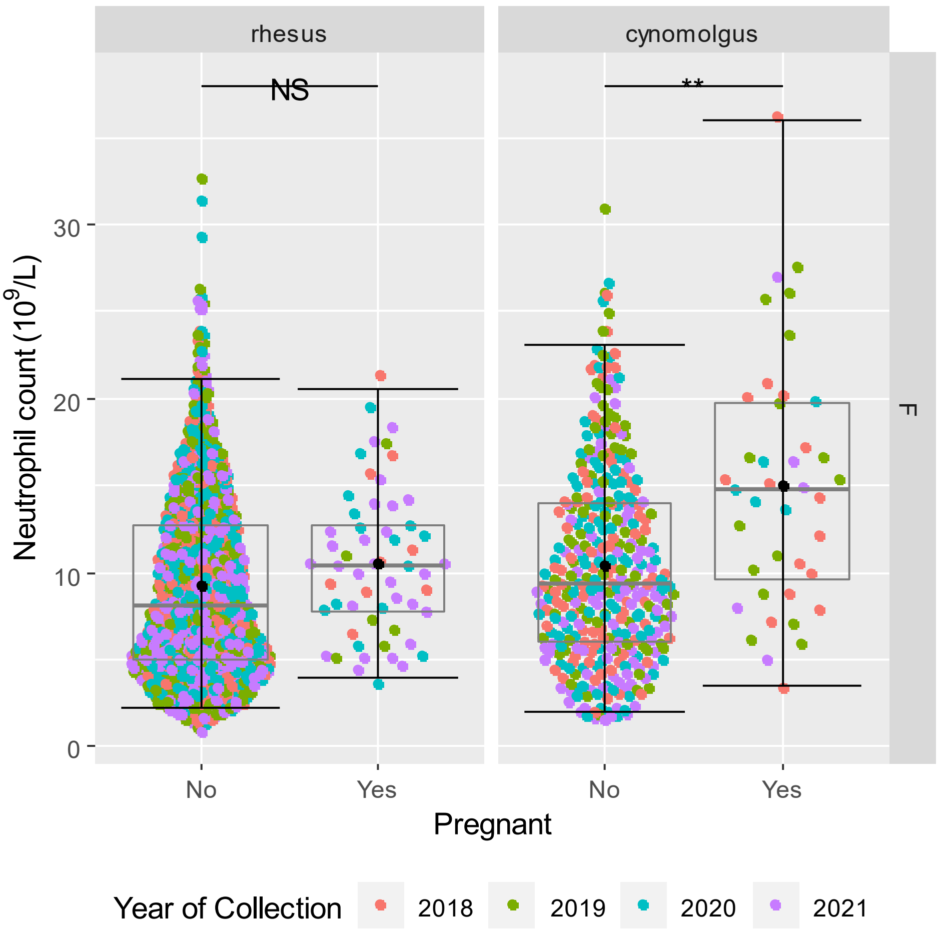

Figure S10 32. Neutrophil Count according to pregnancy in adult female rhesus and cynomolgus macaques. Boxes indicate median and quartile ranges, black point indicates arithmetic mean and error bars indicate the normal range.

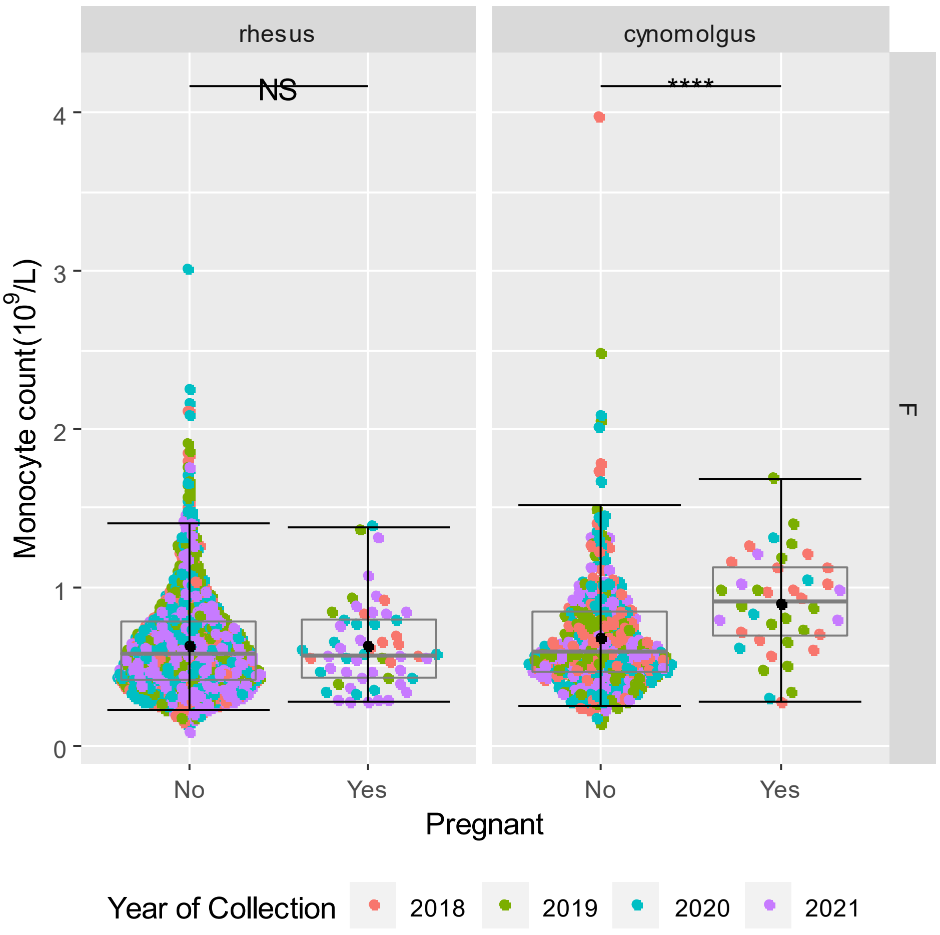

Figure S10 33. Monocyte Count according to pregnancy in adult female rhesus and cynomolgus macaques. Boxes indicate median and quartile ranges, black point indicates arithmetic mean and error bars indicate the normal range.

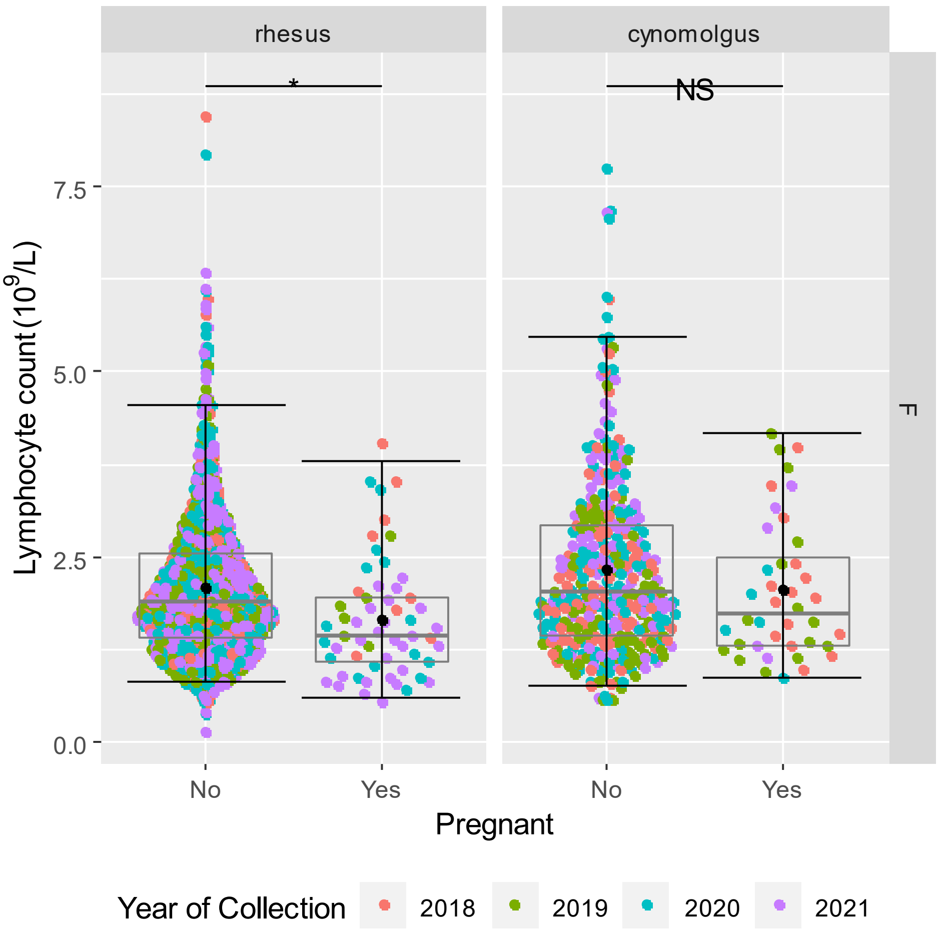

Figure S10 34. Lymphocyte Count according to pregnancy in adult female rhesus and cynomolgus macaques. Boxes indicate median and quartile ranges, black point indicates arithmetic mean and error bars indicate the normal range.

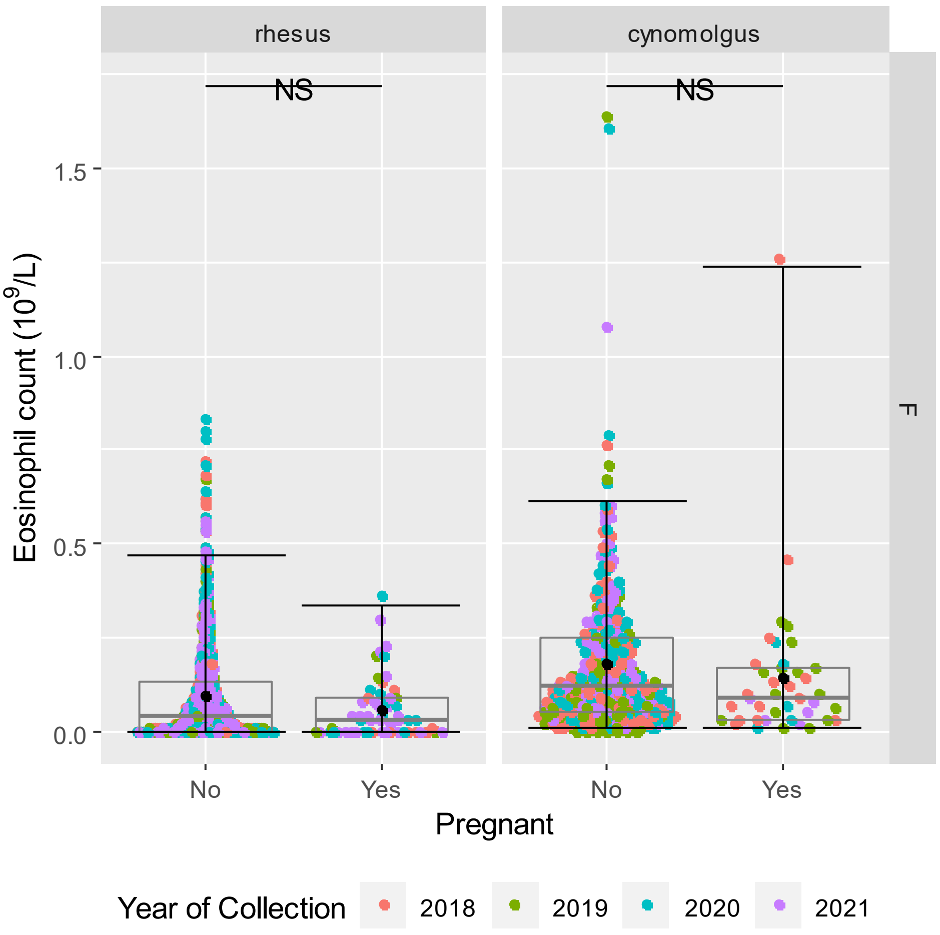

Figure S10 35. Eosinophil Count according to pregnancy in adult female rhesus and cynomolgus macaques. Boxes indicate median and quartile ranges, black point indicates arithmetic mean and error bars indicate the normal range.

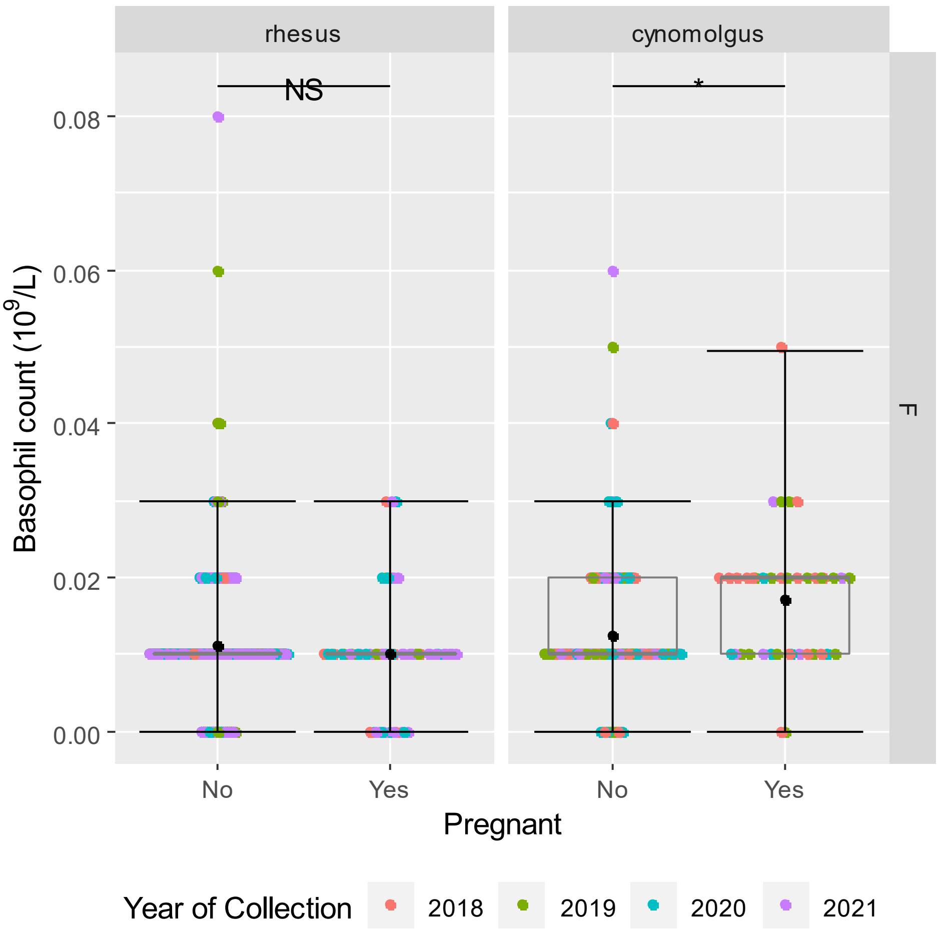

Figure S10 36. Basophil Count according to pregnancy in adult female rhesus and cynomolgus macaques. Boxes indicate median and quartile ranges, black point indicates arithmetic mean and error bars indicate the normal range.
