# Supplementary material for: Reference Intervals and Percentiles for Hematologic and Serum Biochemical Values in Captive Bred Rhesus (Macaca mulatta) and Cynomolgus Macaques (Macaca fascicularis)
Source: Animals (Basel). 2023 Jan 28;13(3):445. doi: 10.3390/ani13030445 (PMC9913310; doi:10.3390/ani13030445)
Supplement: Supplementary file 1 [file animals-13-00445-s001.zip › Figure S2.pdf]

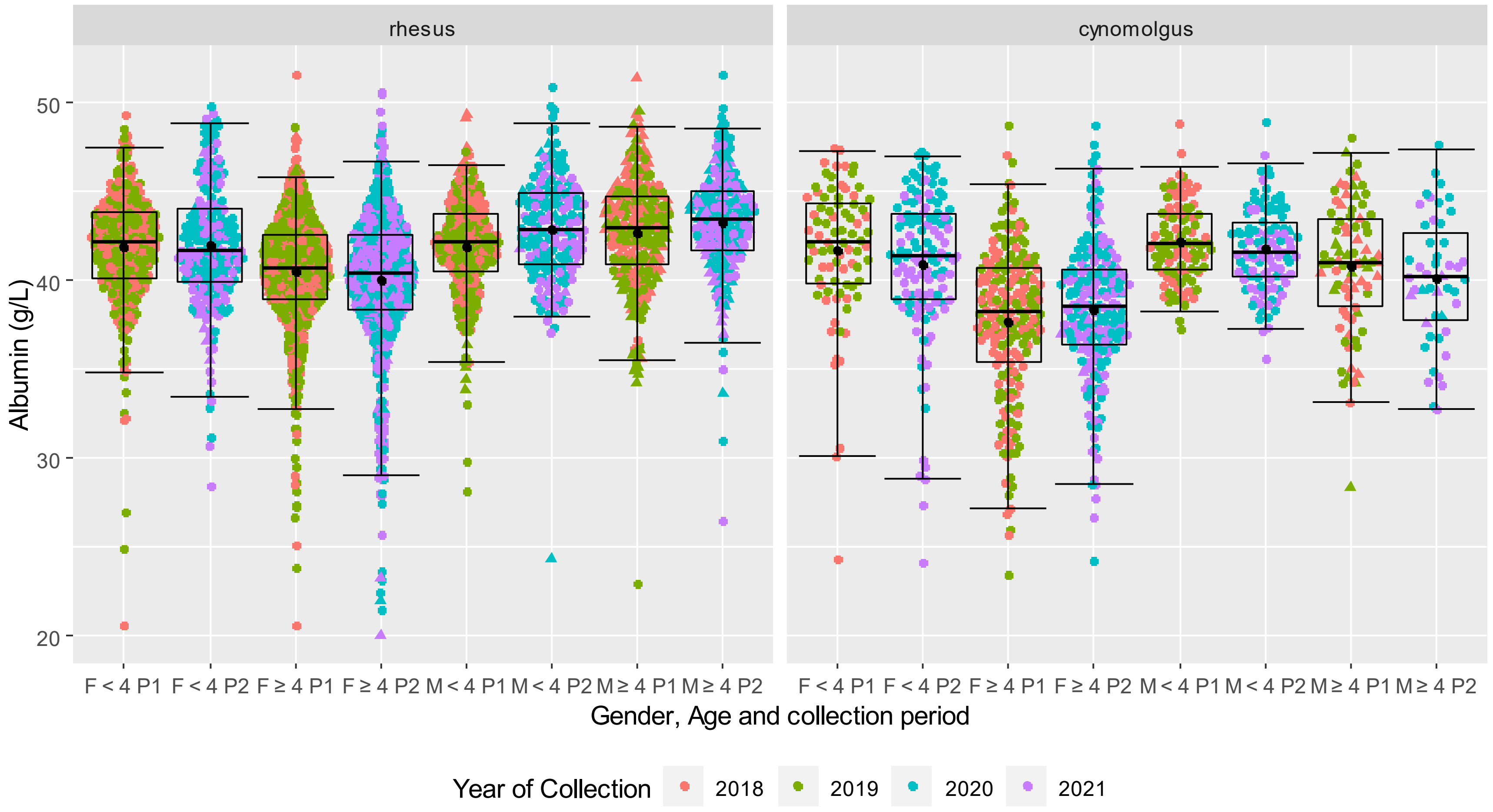

Figure S2 1. Albumin levels according to gender, age and collection period in rhesus and cynomolgus macaques. P1 = 2018 and 2019, P2 = 2020 and 2021. Boxes indicate median and quartile ranges, black point indicates arithmetic mean and error bars indicate the normal range. Round symbols indicate animals with outdoor access and triangles indicate indoor housed animals

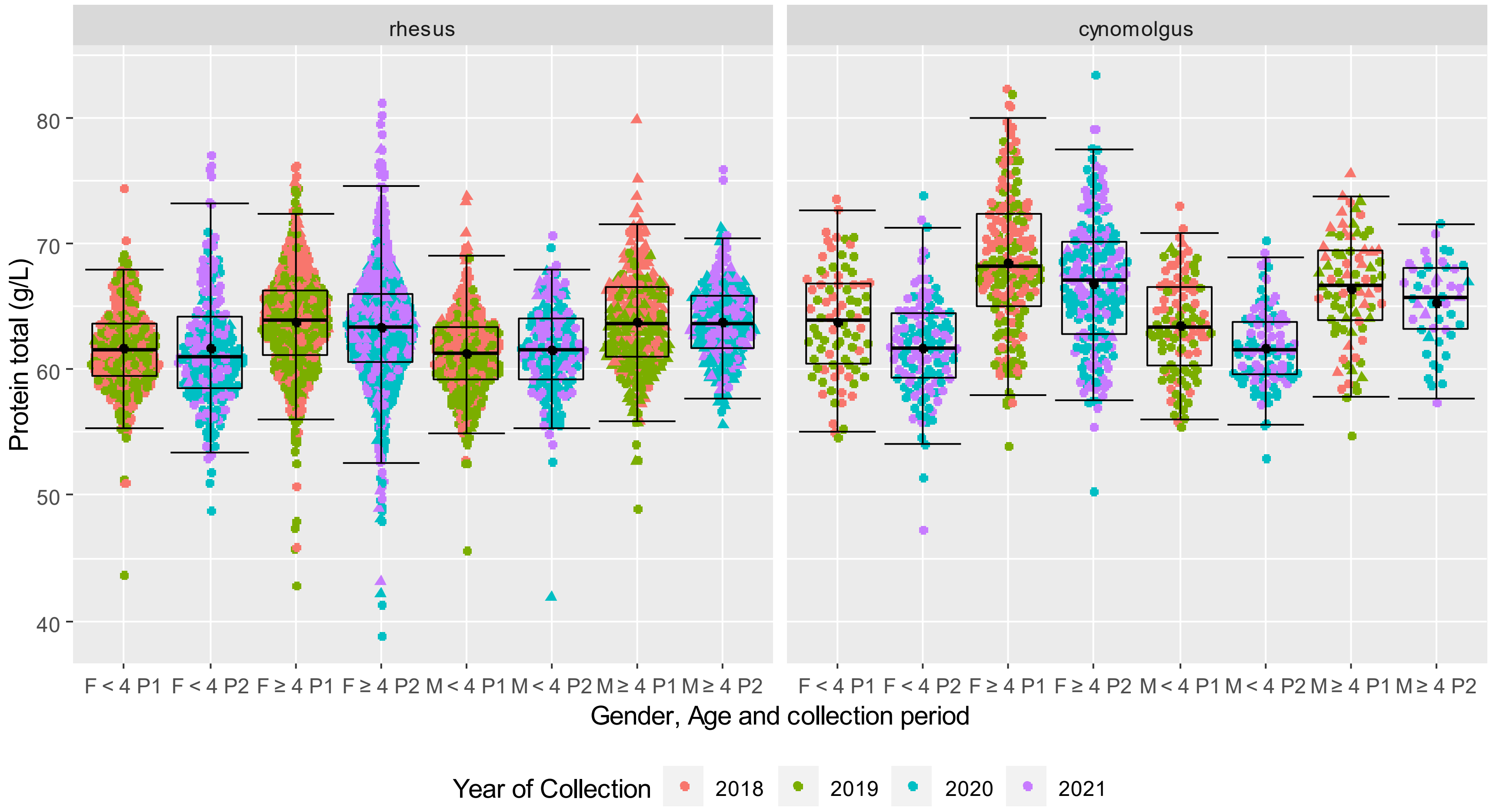

Figure S2 2. Total Protein levels according to gender, age and collection period in rhesus and cynomolgus macaques. P1 = 2018 and 2019, P2 = 2020 and 2021. Boxes indicate median and quartile ranges, black point indicates arithmetic mean and error bars indicate the normal range. Round symbols indicate animals with outdoor access and triangles indicate indoor housed animals

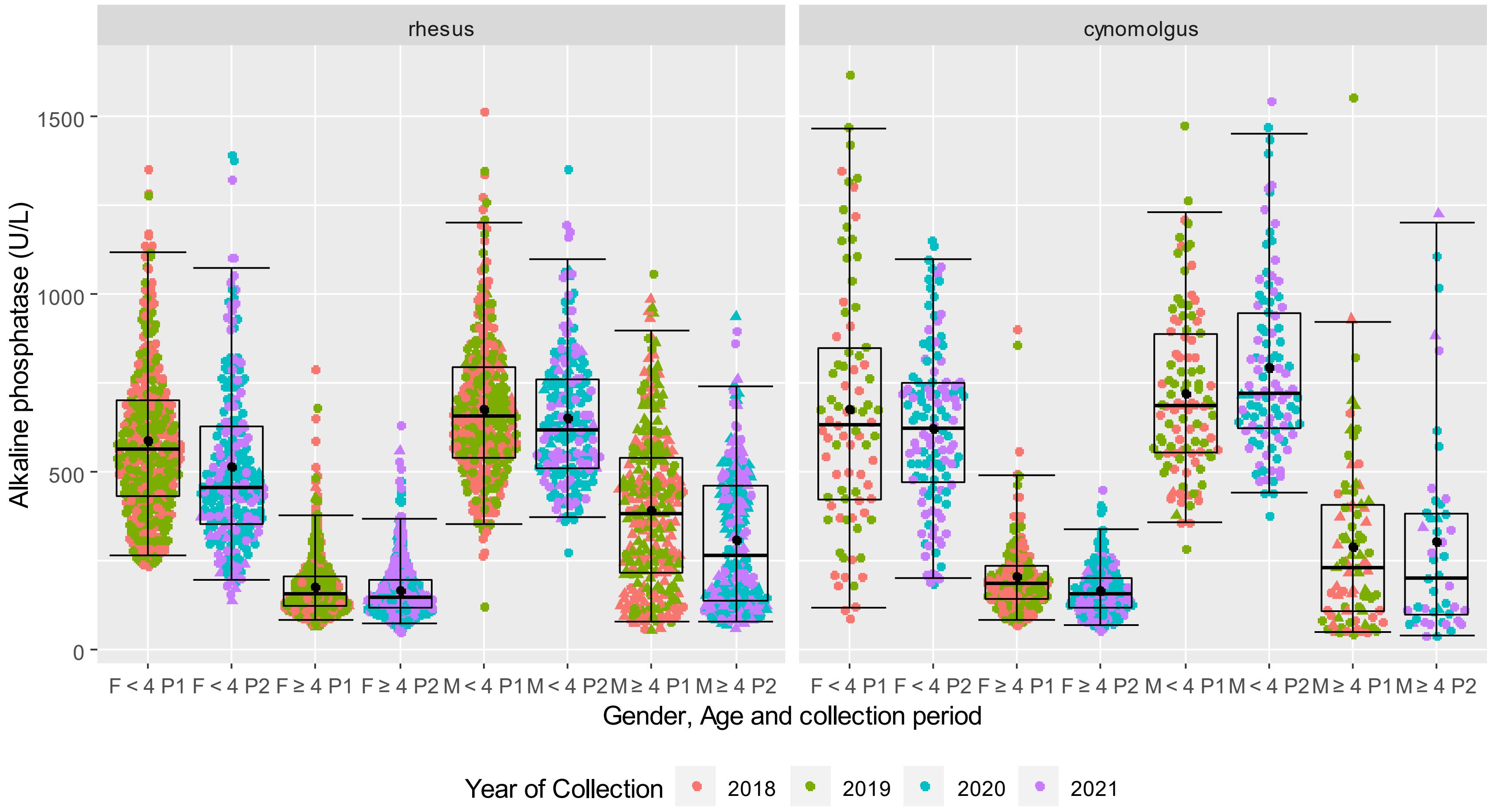

Figure S2 3. Alkaline Phosphatase levels according to year of collection in rhesus and cynomolgus macaques. P1 = 2018 and 2019, P2 = 2020 and 2021. Boxes indicate median and quartile ranges, black point indicates arithmetic mean and error bars indicate the normal range. Round symbols indicate animals with outdoor access and triangles indicate indoor housed animals

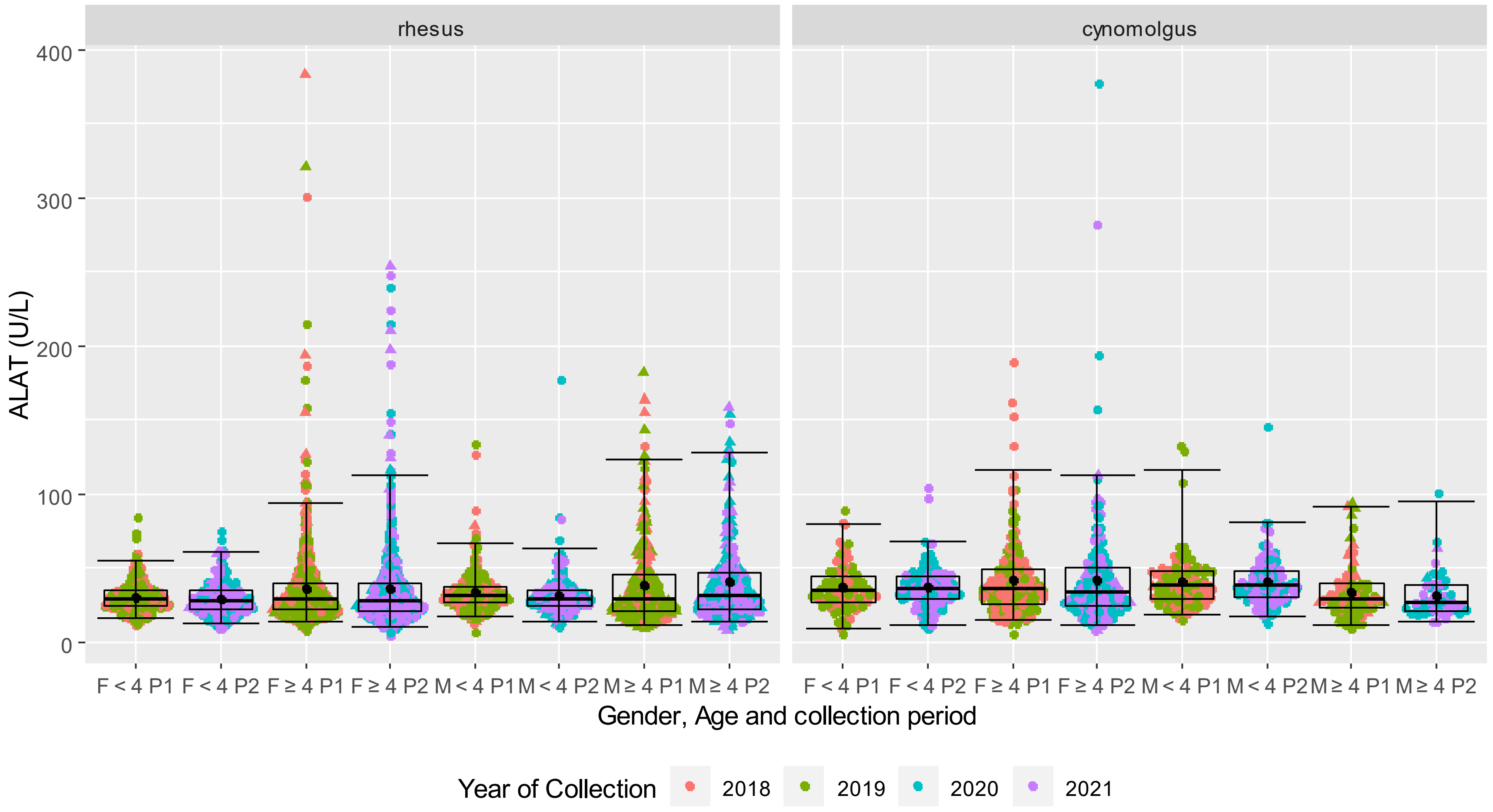

Figure S2 4. ALAT levels according to gender, age and collection period in rhesus and cynomolgus macaques. P1 = 2018 and 2019, P2 = 2020 and 2021. Boxes indicate median and quartile ranges, black point indicates arithmetic mean and error bars indicate the normal range. Round symbols indicate animals with outdoor access and triangles indicate indoor housed animals

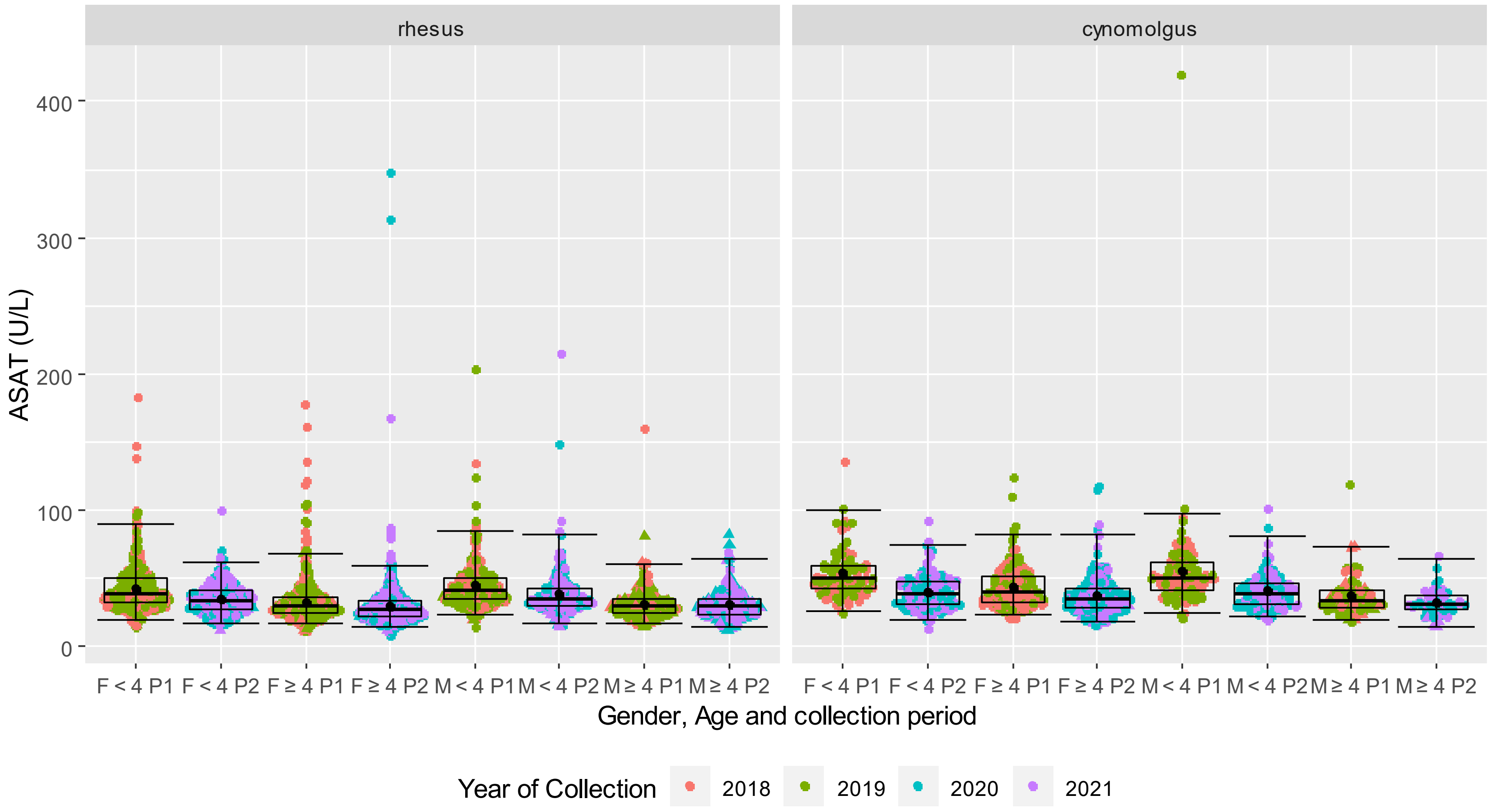

Figure S2 5. ASAT levels according to gender, age and collection period in rhesus and cynomolgus macaques. P1 = 2018 and 2019, P2 = 2020 and 2021. Boxes indicate median and quartile ranges, black point indicates arithmetic mean and error bars indicate the normal range. Round symbols indicate animals with outdoor access and triangles indicate indoor housed animals

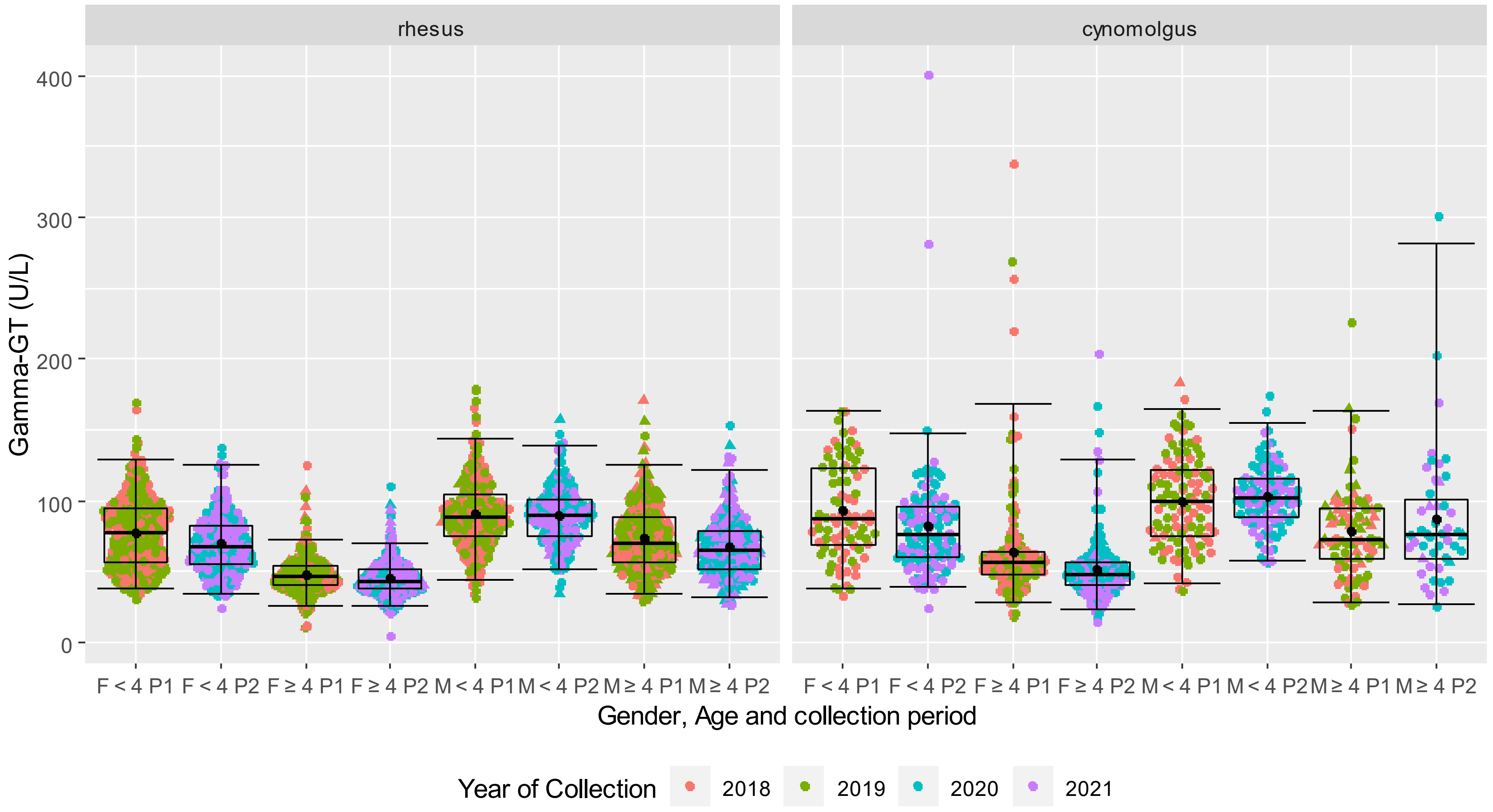

Figure S2 6. Gamma-GT levels according to gender, age and collection period in rhesus and cynomolgus macaques. P1 = 2018 and 2019, P2 = 2020 and 2021. Boxes indicate median and quartile ranges, black point indicates arithmetic mean and error bars indicate the normal range. Round symbols indicate animals with outdoor access and triangles indicate indoor housed animals

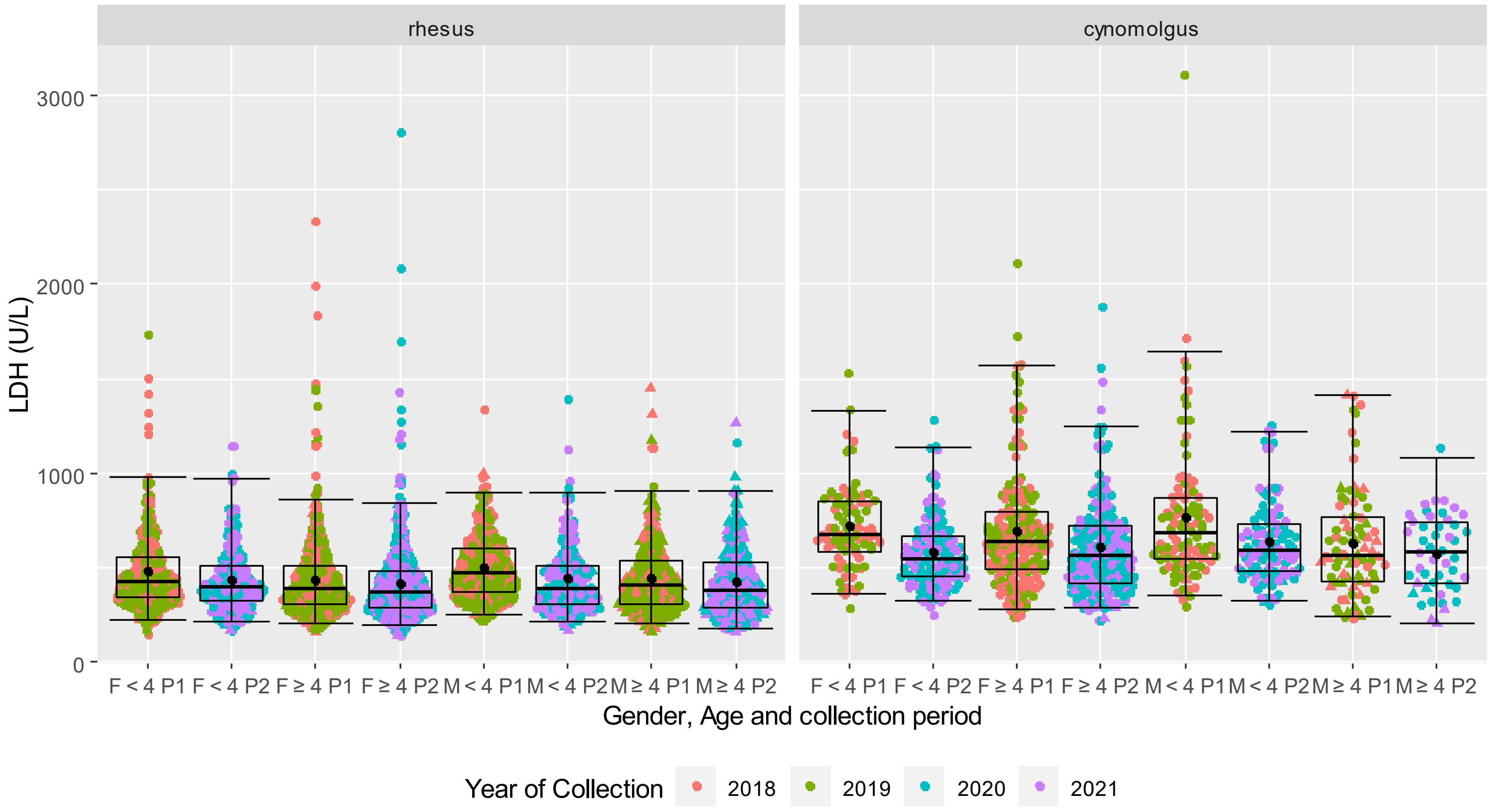

Figure S2 7. LDH levels according to gender, age and collection period in rhesus and cynomolgus macaques. P1 = 2018 and 2019, P2 = 2020 and 2021. Boxes indicate median and quartile ranges, black point indicates arithmetic mean and error bars indicate the normal range. Round symbols indicate animals with outdoor access and triangles indicate indoor housed animals

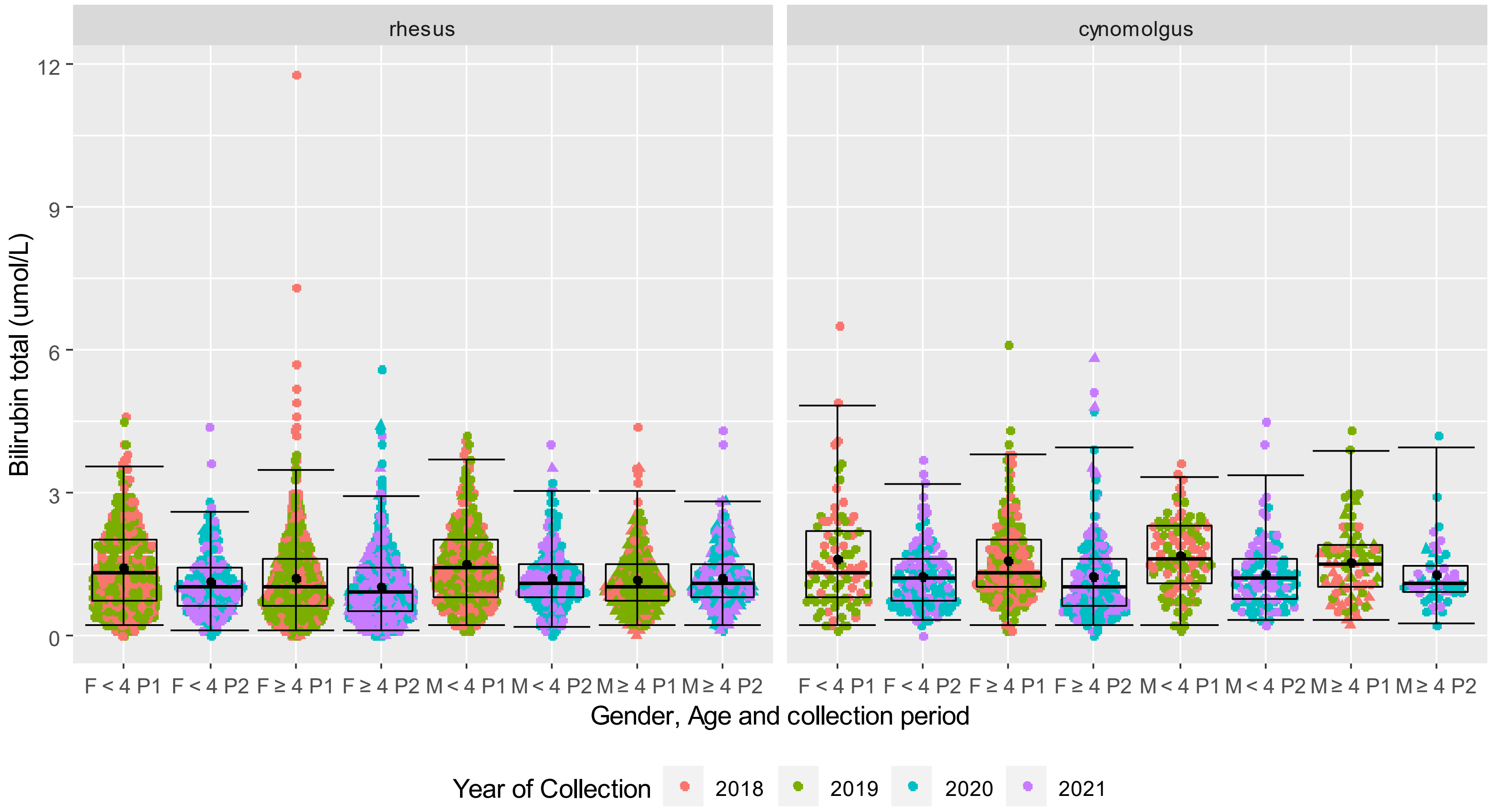

Figure S2 8. Total Bilirubin levels according to gender, age and collection period in rhesus and cynomolgus macaques. P1 = 2018 and 2019, P2 = 2020 and 2021. Boxes indicate median and quartile ranges, black point indicates arithmetic mean and error bars indicate the normal range. Round symbols indicate animals with outdoor access and triangles indicate indoor housed animals

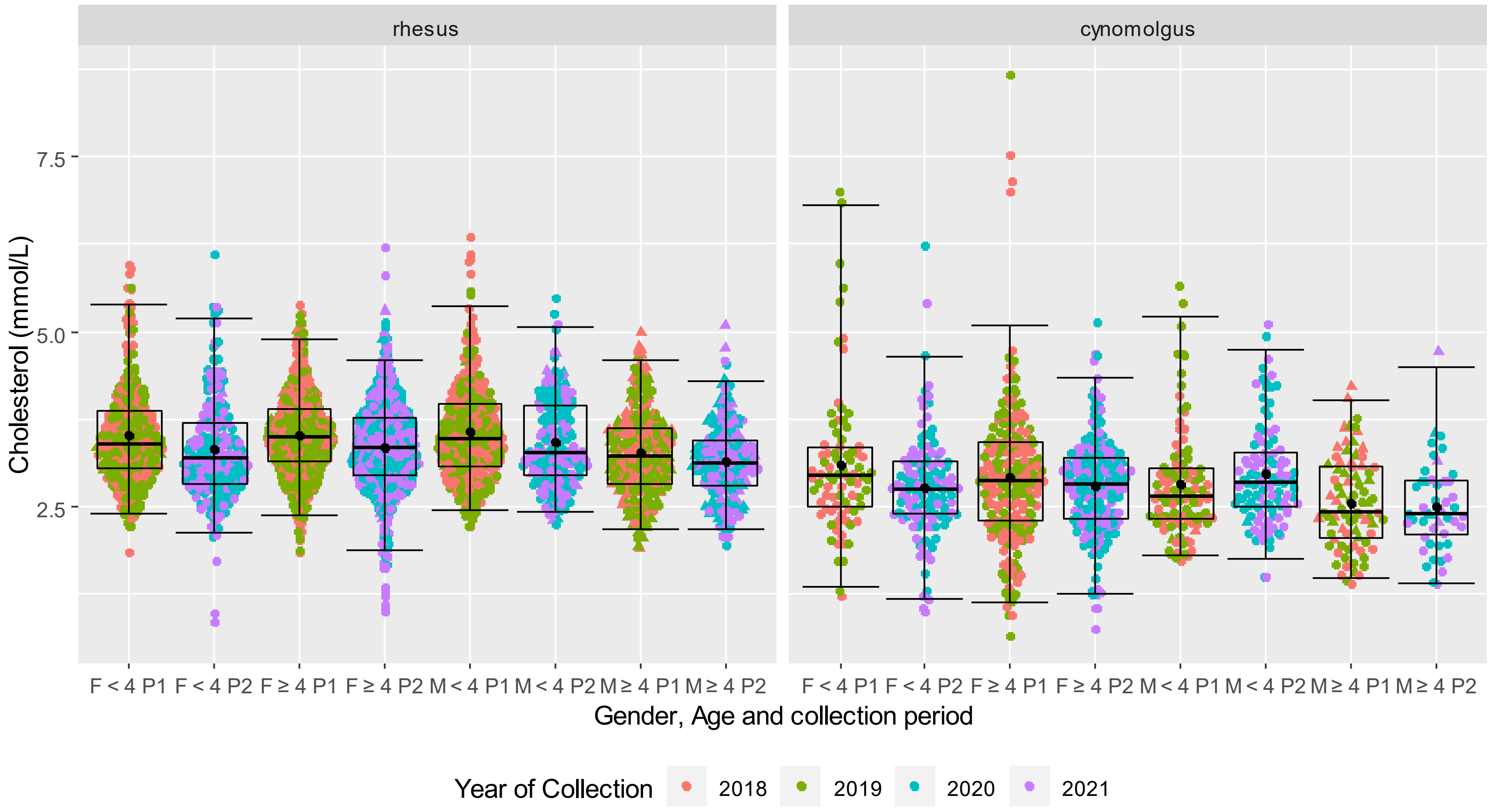

Figure S2 9. Cholesterol levels according to gender, age and collection period in rhesus and cynomolgus macaques. P1 = 2018 and 2019, P2 = 2020 and 2021. Boxes indicate median and quartile ranges, black point indicates arithmetic mean and error bars indicate the normal range. Round symbols indicate animals with outdoor access and triangles indicate indoor housed animals

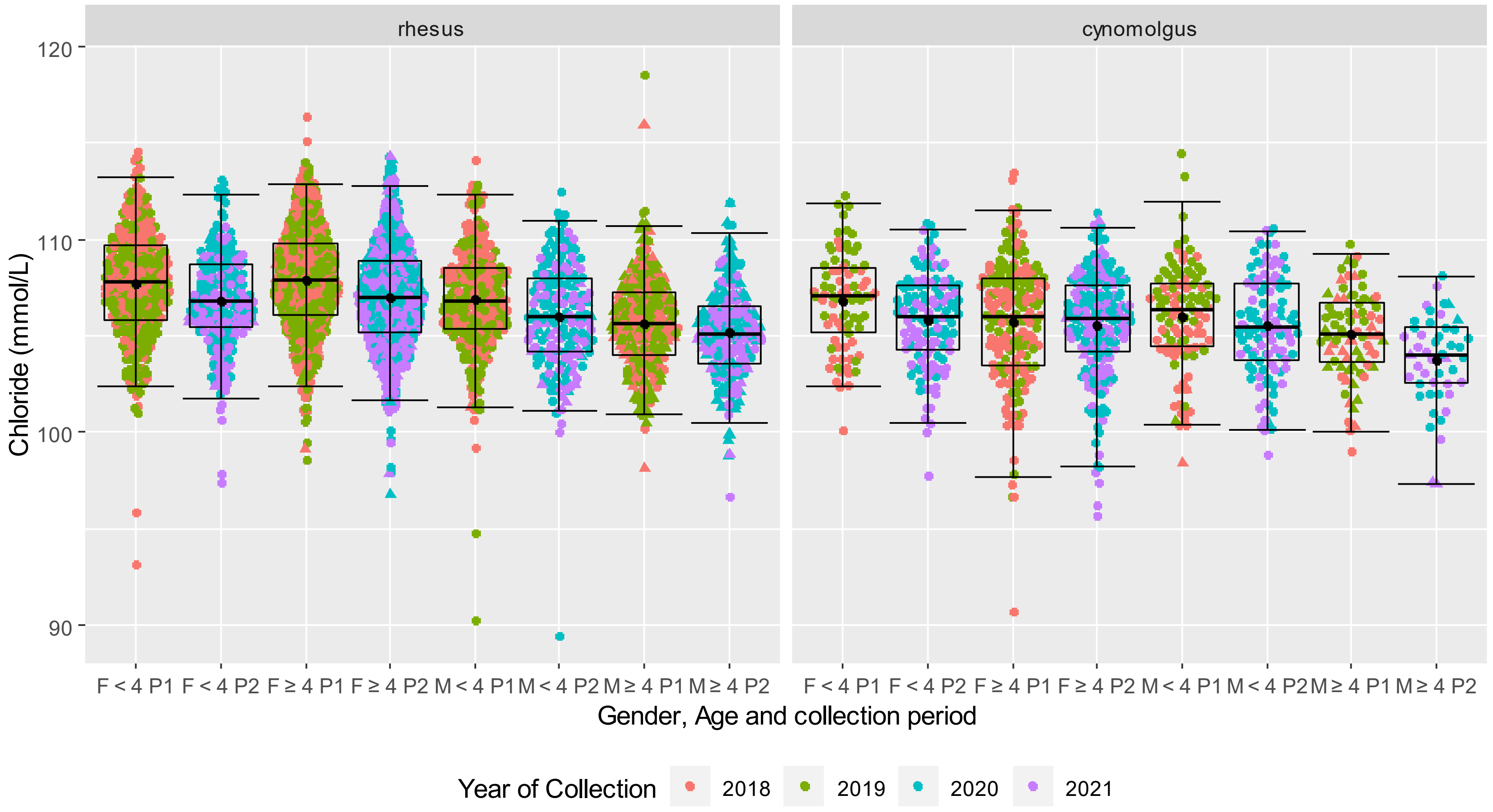

Figure S2 10. Chloride levels according to gender, age and collection period in rhesus and cynomolgus macaques. P1 = 2018 and 2019, P2 = 2020 and 2021. Boxes indicate median and quartile ranges, black point indicates arithmetic mean and error bars indicate the normal range. Round symbols indicate animals with outdoor access and triangles indicate indoor housed animals

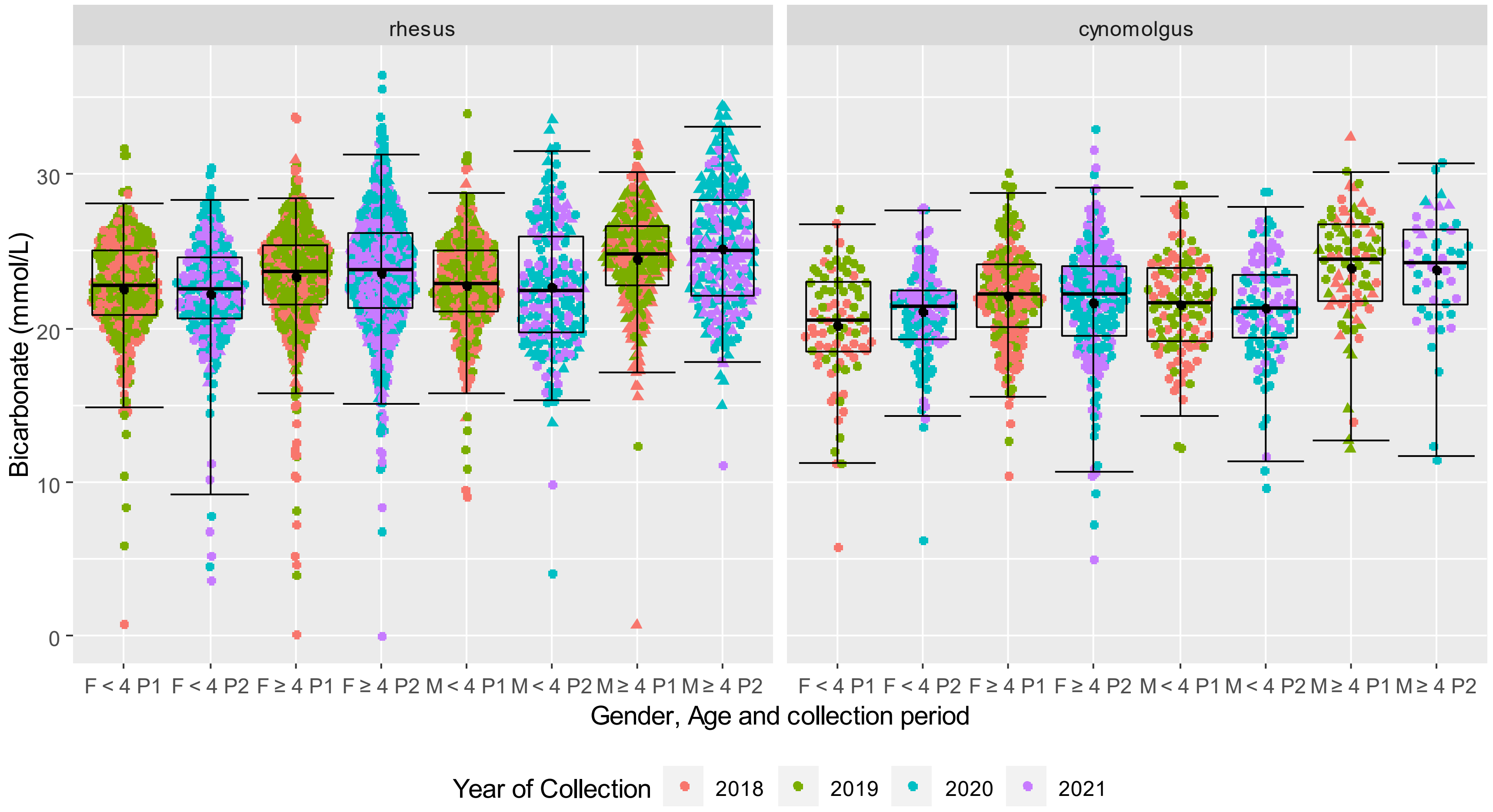

Figure S2 11. Bicarbonate levels according to gender, age and collection period in rhesus and cynomolgus macaques. P1 = 2018 and 2019, P2 = 2020 and 2021. Boxes indicate median and quartile ranges, black point indicates arithmetic mean and error bars indicate the normal range. Round symbols indicate animals with outdoor access and triangles indicate indoor housed animals

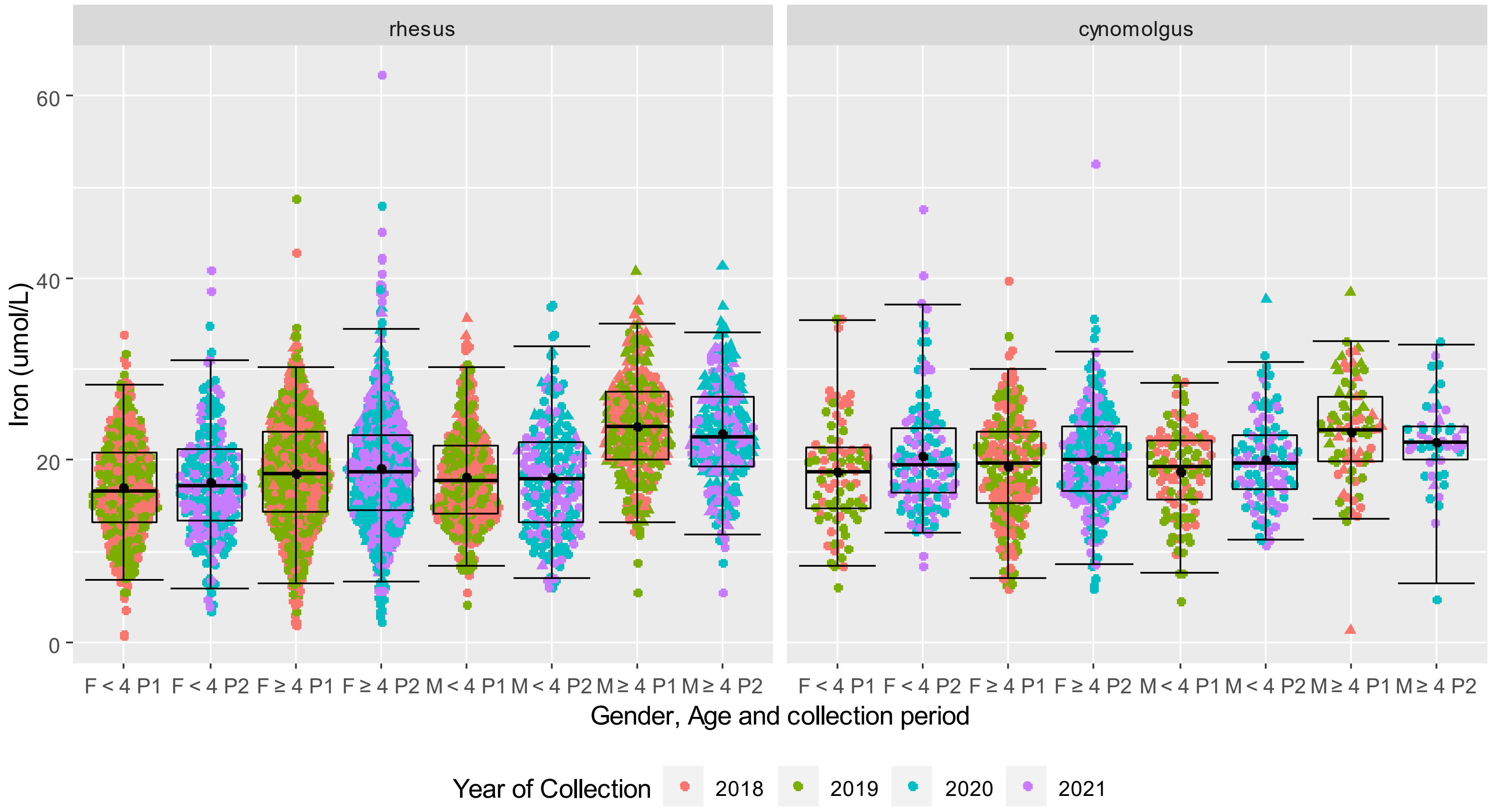

Figure S2 12. Iron levels according to gender, age and collection period in rhesus and cynomolgus macaques. P1 = 2018 and 2019, P2 = 2020 and 2021. Boxes indicate median and quartile ranges, black point indicates arithmetic mean and error bars indicate the normal range. Round symbols indicate animals with outdoor access and triangles indicate indoor housed animals

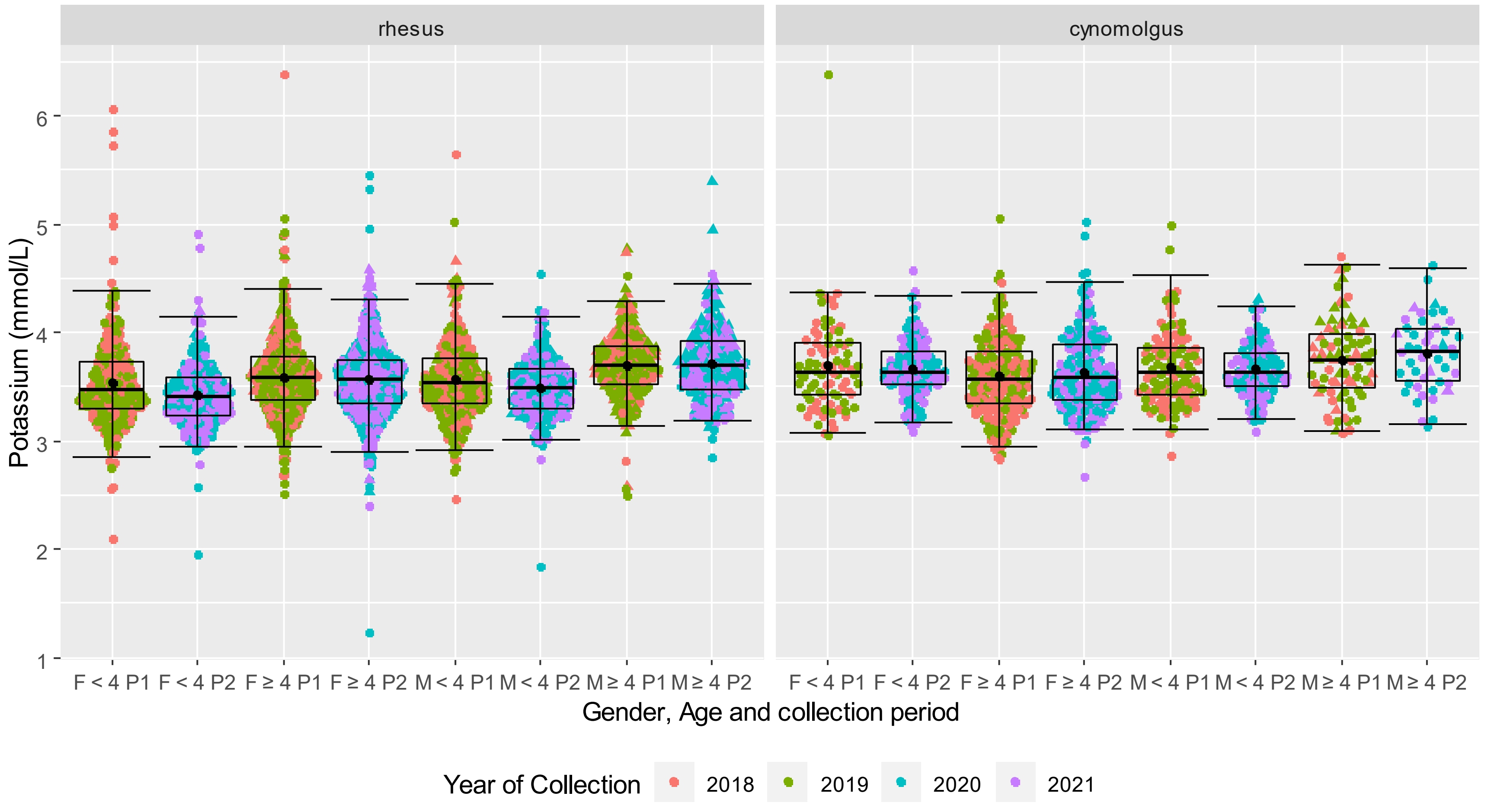

Figure S2 13. Potassium levels according to gender, age and collection period in rhesus and cynomolgus macaques. P1 = 2018 and 2019, P2 = 2020 and 2021. Boxes indicate median and quartile ranges, black point indicates arithmetic mean and error bars indicate the normal range. Round symbols indicate animals with outdoor access and triangles indicate indoor housed animals

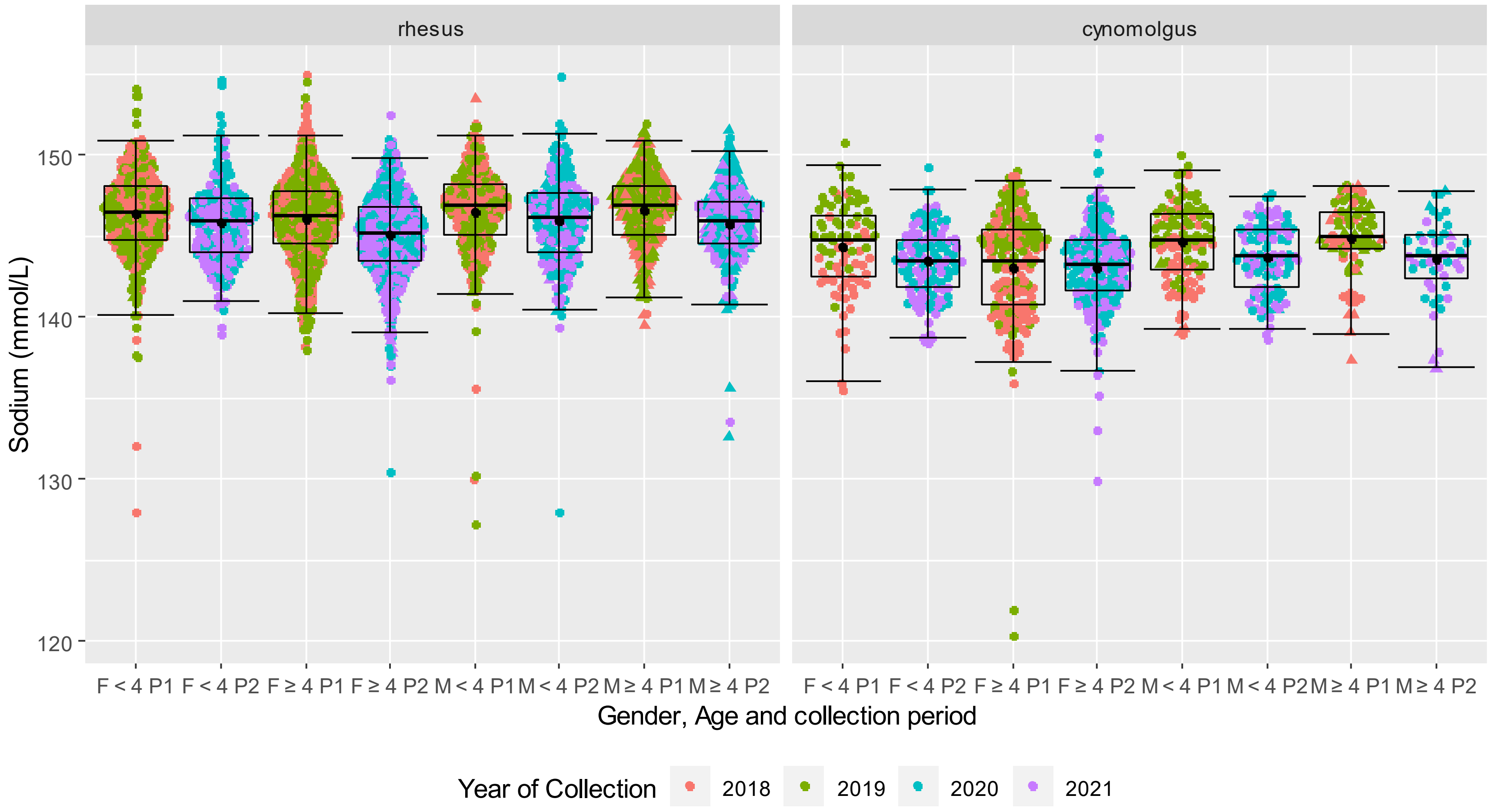

Figure S2 14. Sodium levels according to gender, age and collection period in rhesus and cynomolgus macaques. P1 = 2018 and 2019, P2 = 2020 and 2021. Boxes indicate median and quartile ranges, black point indicates arithmetic mean and error bars indicate the normal range. Round symbols indicate animals with outdoor access and triangles indicate indoor housed animals

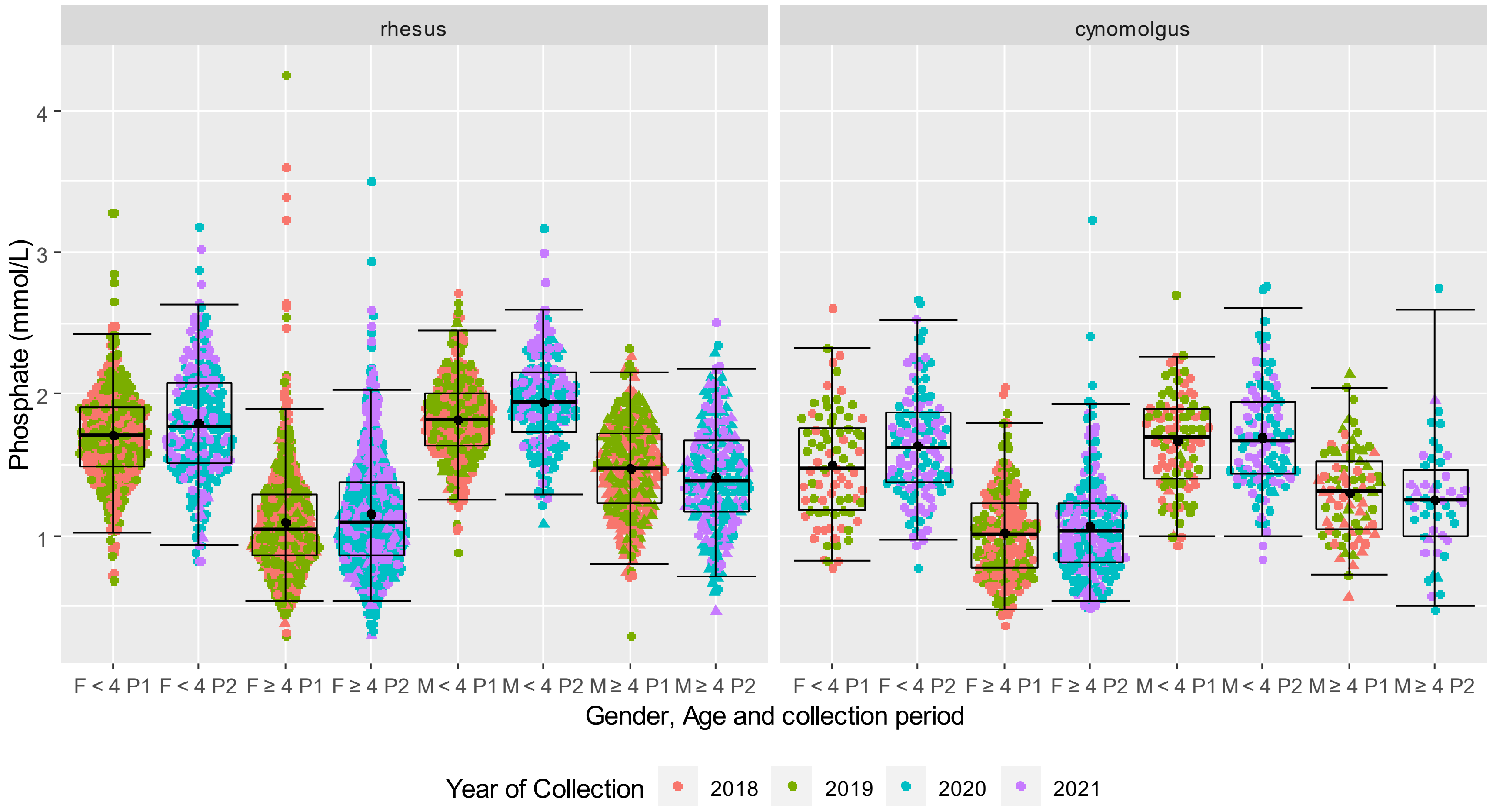

Figure S2 15. Phosphate levels according to gender, age and collection period in rhesus and cynomolgus macaques. P1 = 2018 and 2019, P2 = 2020 and 2021. Boxes indicate median and quartile ranges, black point indicates arithmetic mean and error bars indicate the normal range. Round symbols indicate animals with outdoor access and triangles indicate indoor housed animals

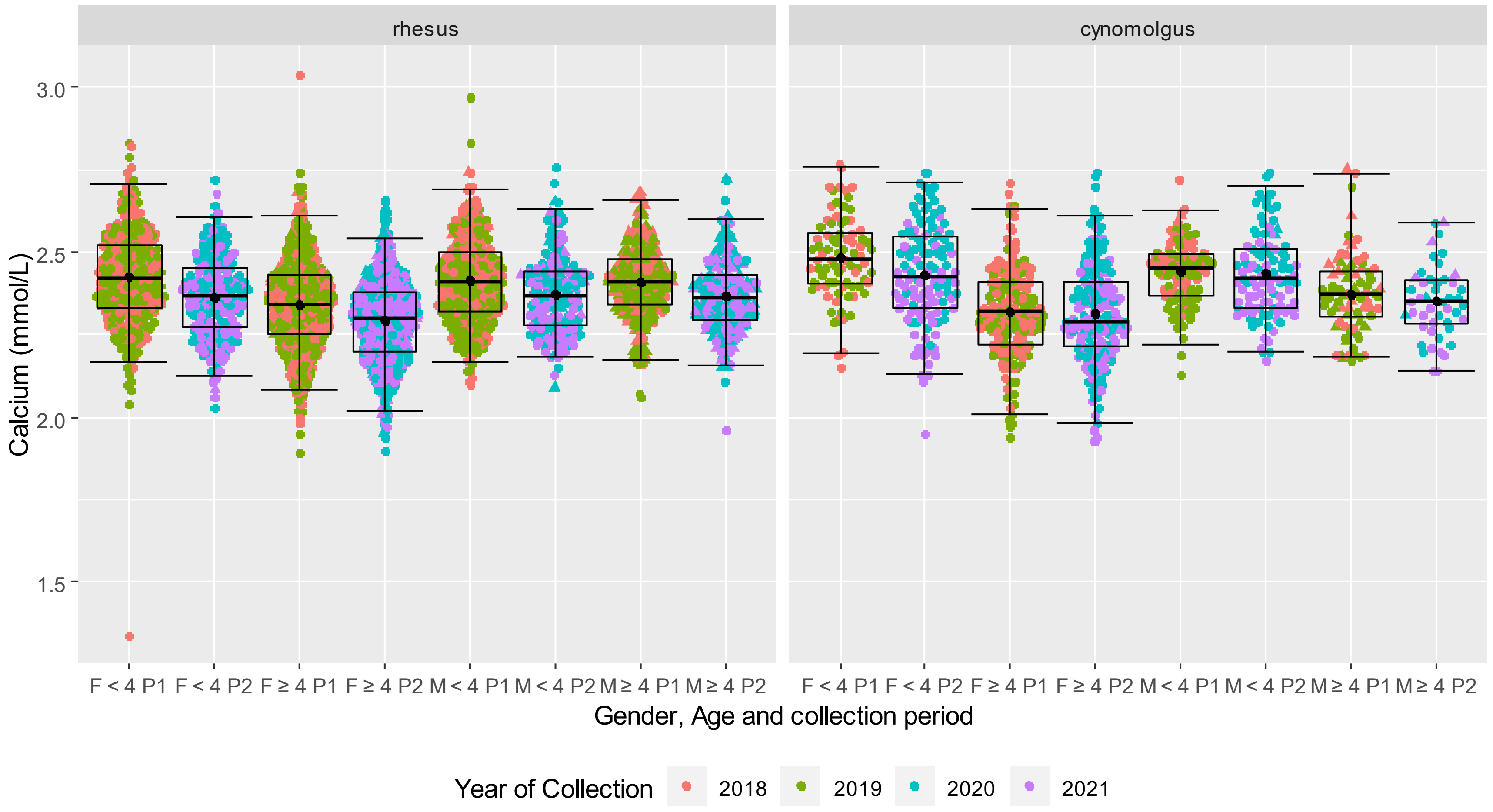

Figure S2 16. Calcium levels according to gender, age and collection period in rhesus and cynomolgus macaques. P1 = 2018 and 2019, P2 = 2020 and 2021. Boxes indicate median and quartile ranges, black point indicates arithmetic mean and error bars indicate the normal range. Round symbols indicate animals with outdoor access and triangles indicate indoor housed animals

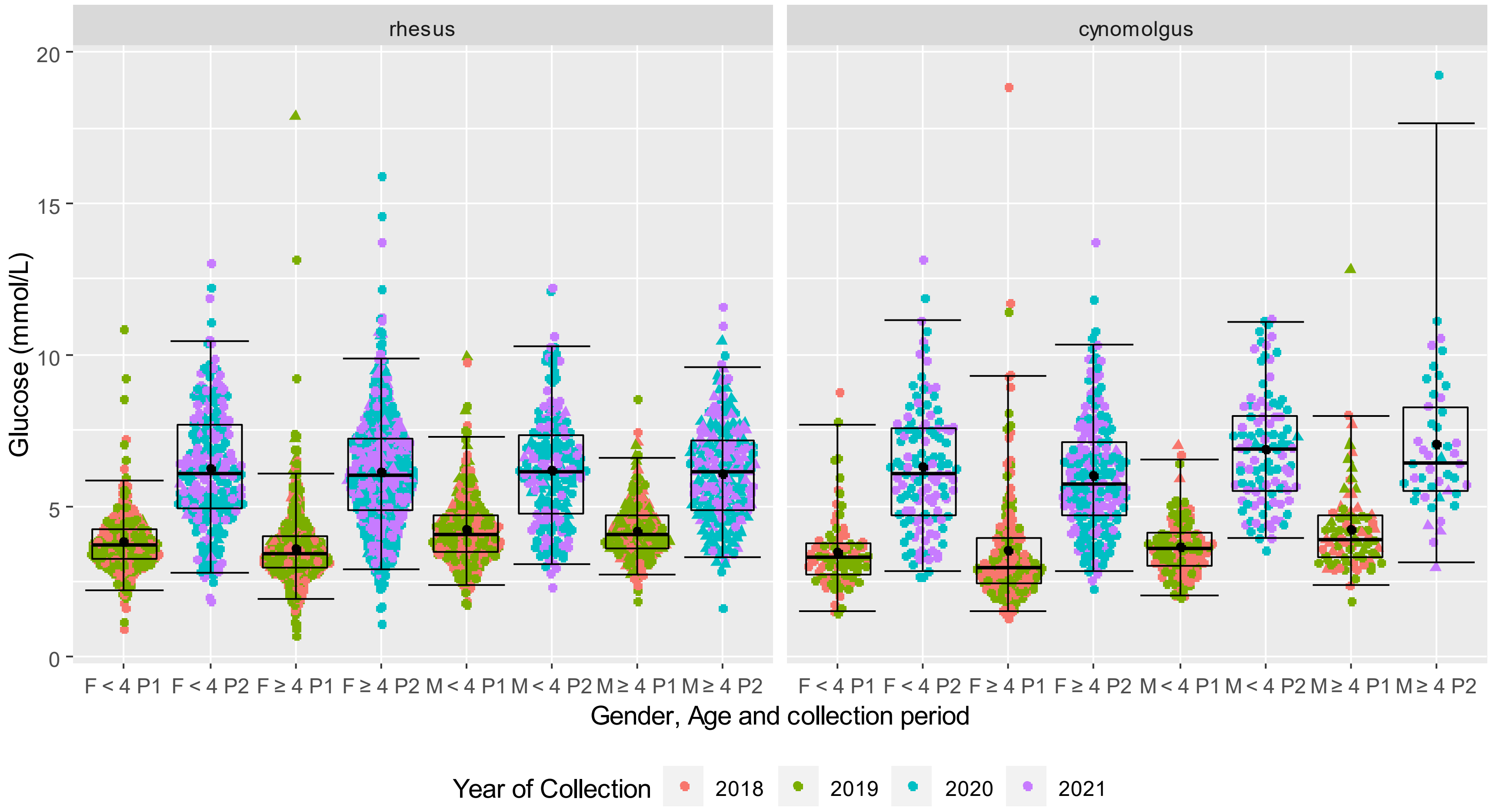

Figure S2 17. Glucose levels according to gender, age and collection period in rhesus and cynomolgus macaques. P1 = 2018 and 2019, P2 = 2020 and 2021. Boxes indicate median and quartile ranges, black point indicates arithmetic mean and error bars indicate the normal range. Round symbols indicate animals with outdoor access and triangles indicate indoor housed animals

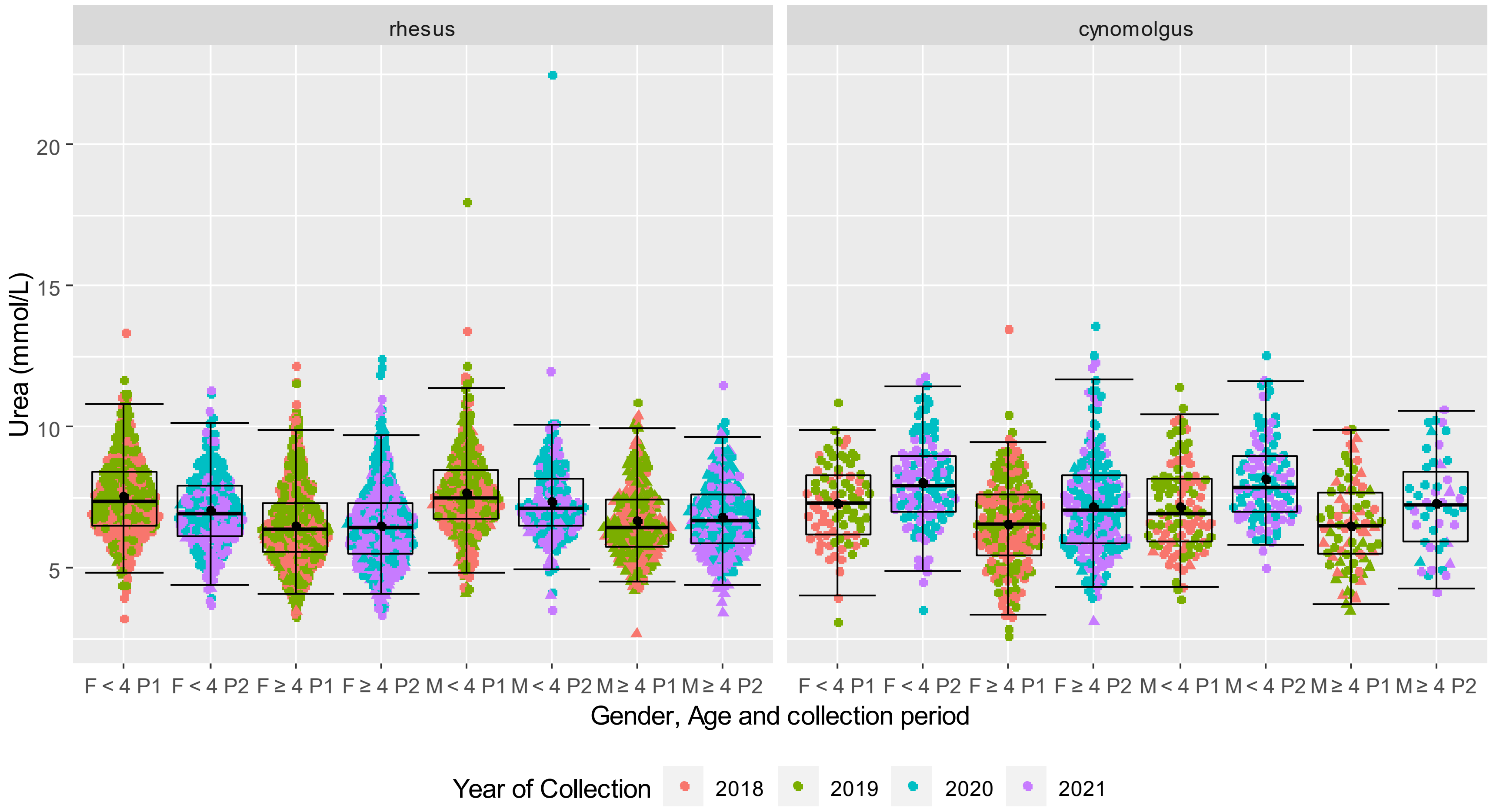

Figure S2 18. Urea levels according to gender, age and collection period in rhesus and cynomolgus macaques. P1 = 2018 and 2019, P2 = 2020 and 2021. Boxes indicate median and quartile ranges, black point indicates arithmetic mean and error bars indicate the normal range. Round symbols indicate animals with outdoor access and triangles indicate indoor housed animals

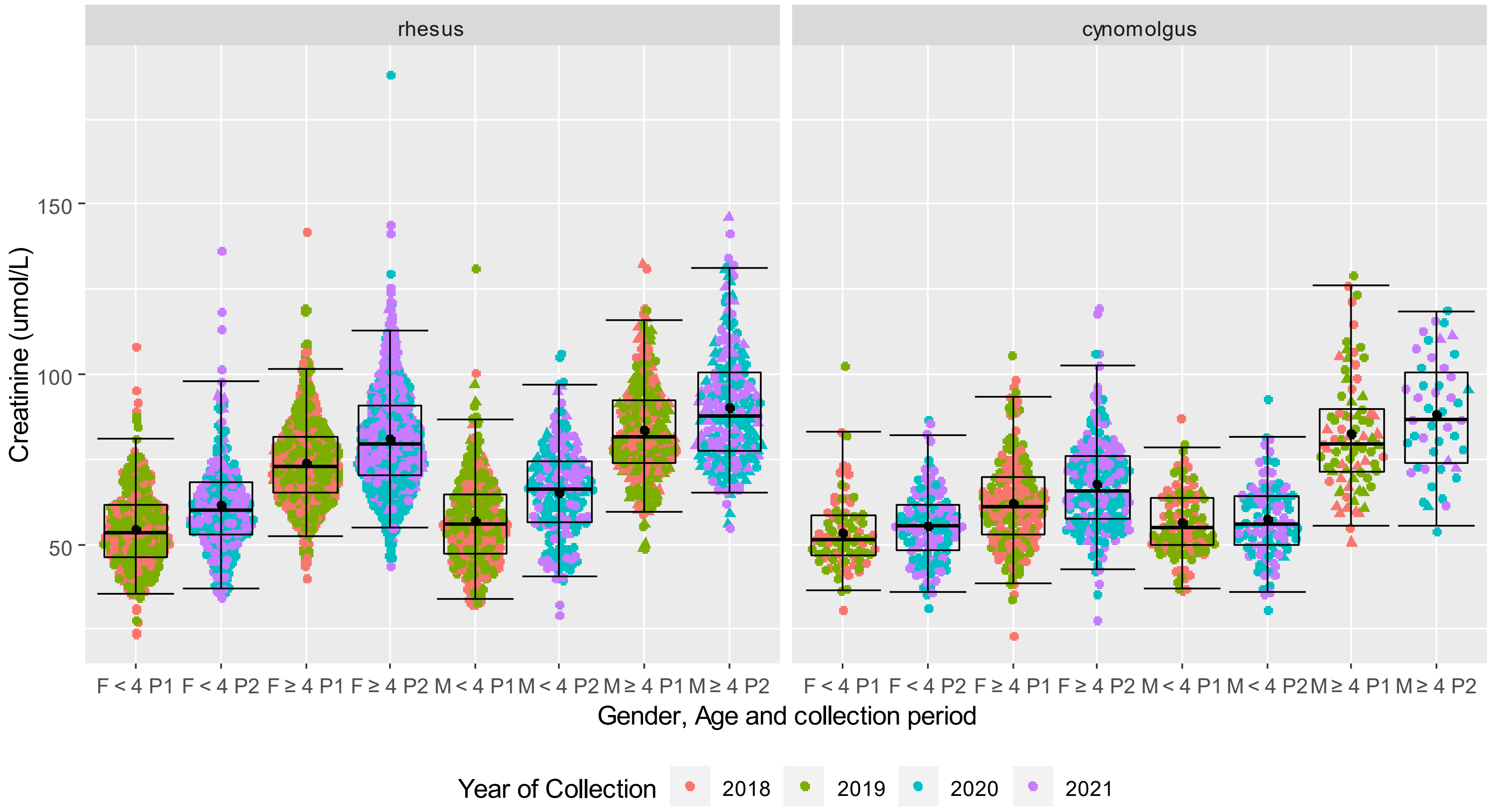

Figure S2 19. Creatinine levels according to gender, age and collection period in rhesus and cynomolgus macaques. P1 = 2018 and 2019, P2 = 2020 and 2021. Boxes indicate median and quartile ranges, black point indicates arithmetic mean and error bars indicate the normal range. Round symbols indicate animals with outdoor access and triangles indicate indoor housed animals

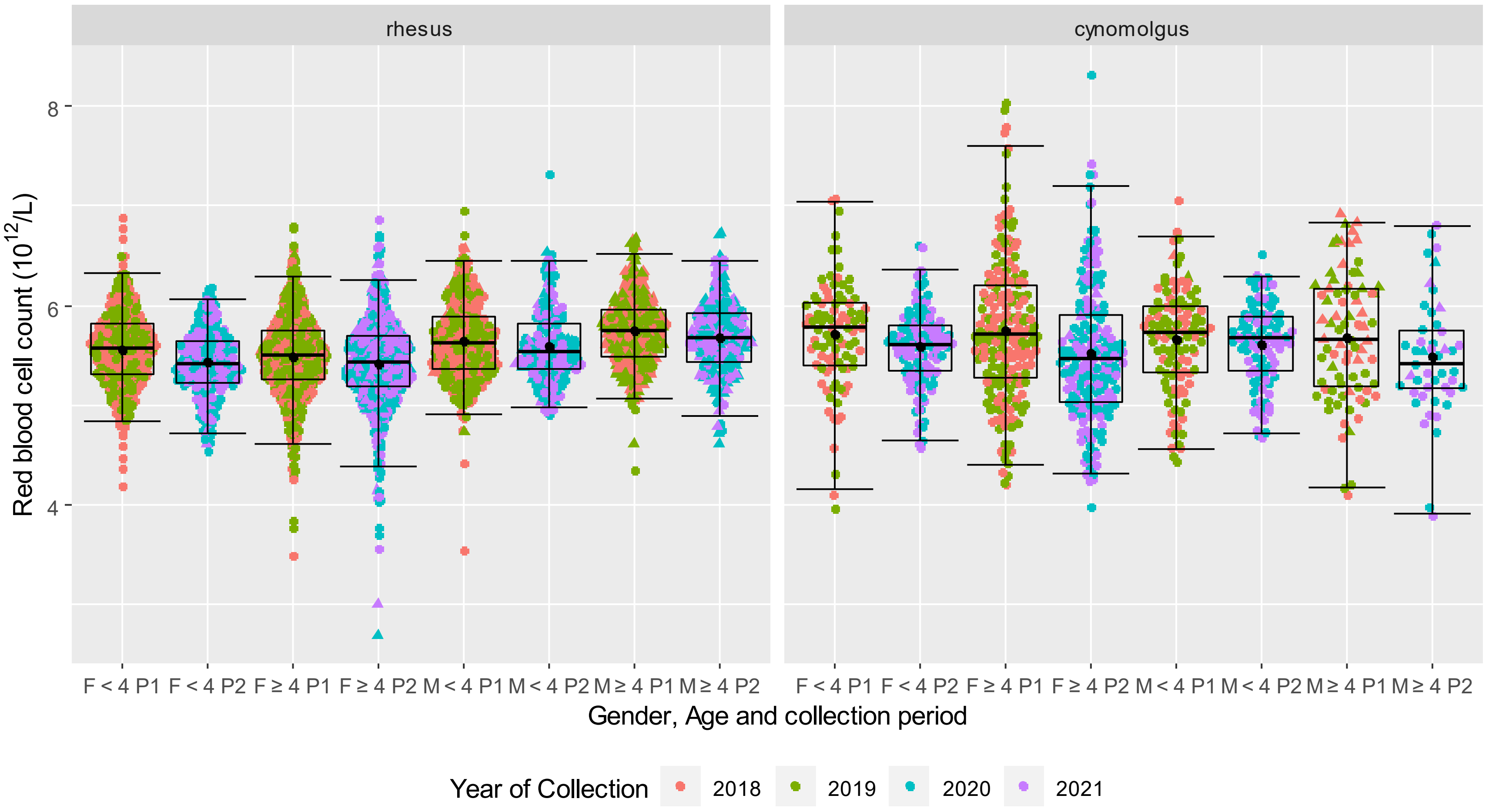

Figure S2 20. Red Blood Cell count according to gender, age and collection period in rhesus and cynomolgus macaques. P1 = 2018 and 2019, P2 = 2020 and 2021. Boxes indicate median and quartile ranges, black point indicates arithmetic mean and error bars indicate the normal range. Round symbols indicate animals with outdoor access and triangles indicate indoor housed animals

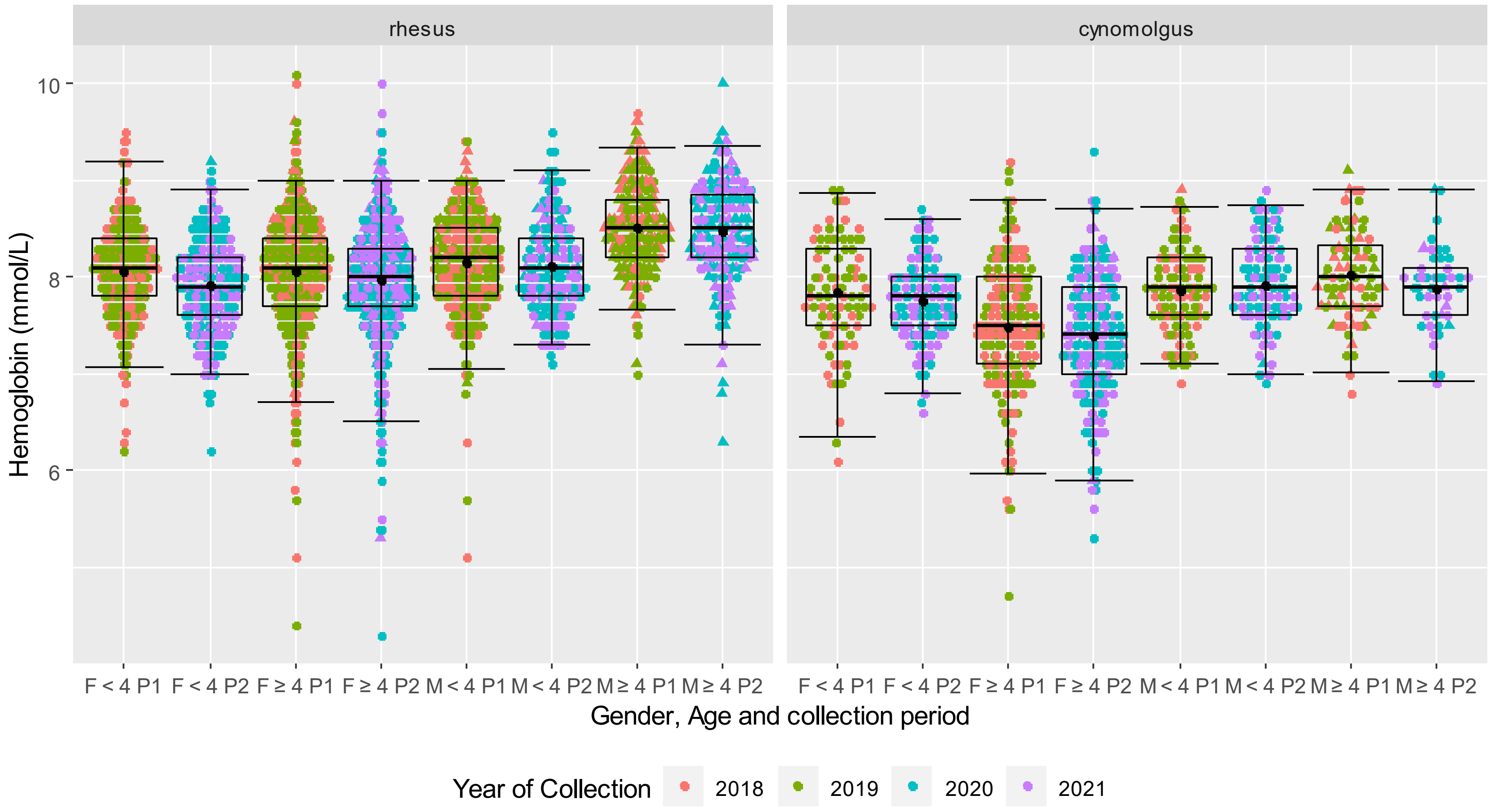

Figure S2 21. Hemoglobin levels according to gender, age and collection period in rhesus and cynomolgus macaques. P1 = 2018 and 2019, P2 = 2020 and 2021. Boxes indicate median and quartile ranges, black point indicates arithmetic mean and error bars indicate the normal range. Round symbols indicate animals with outdoor access and triangles indicate indoor housed animals

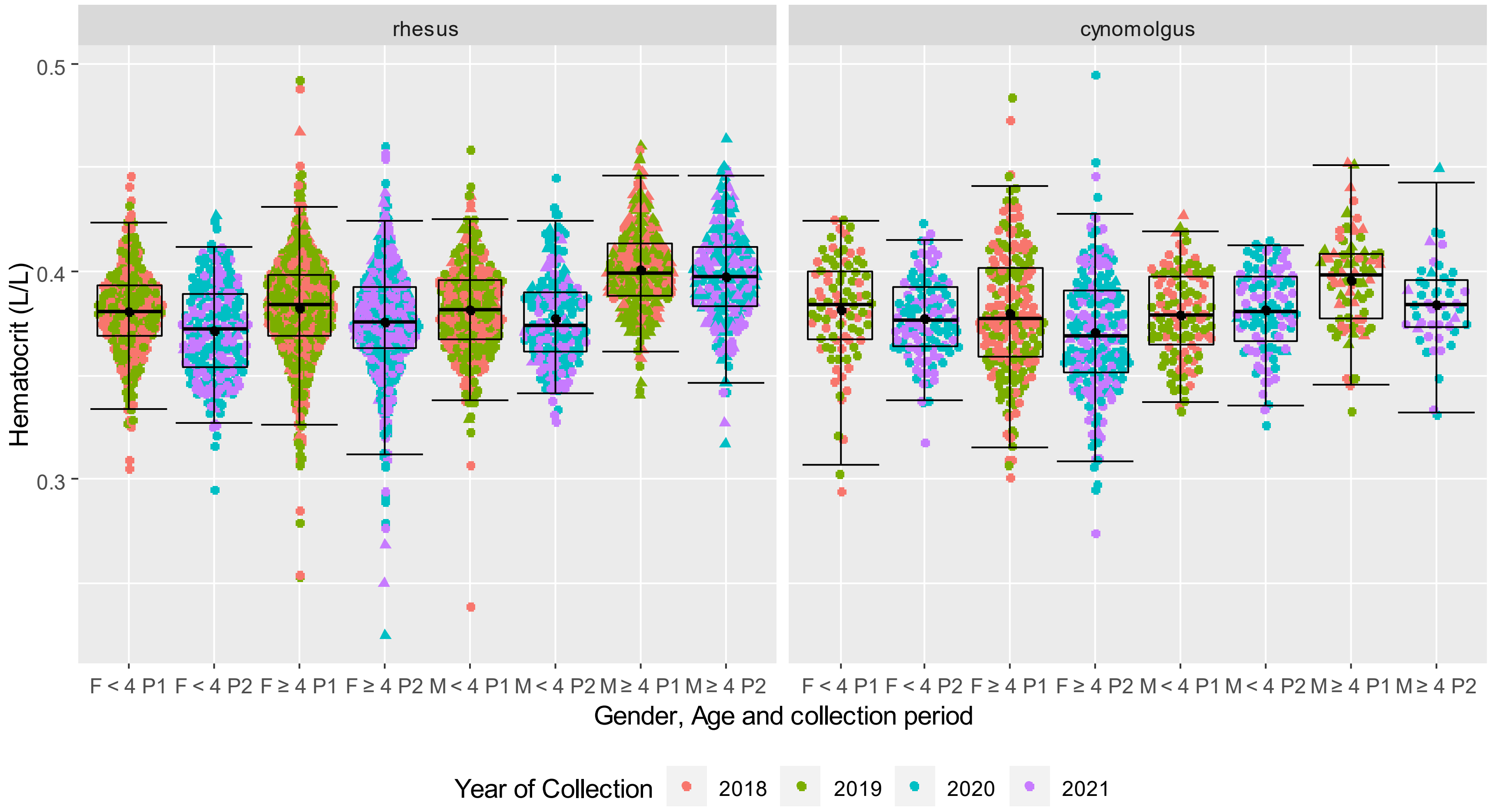

Figure S2 22. Hematocrit levels according to gender, age and collection period in rhesus and cynomolgus macaques. P1 = 2018 and 2019, P2 = 2020 and 2021. Boxes indicate median and quartile ranges, black point indicates arithmetic mean and error bars indicate the normal range. Round symbols indicate animals with outdoor access and triangles indicate indoor housed animals

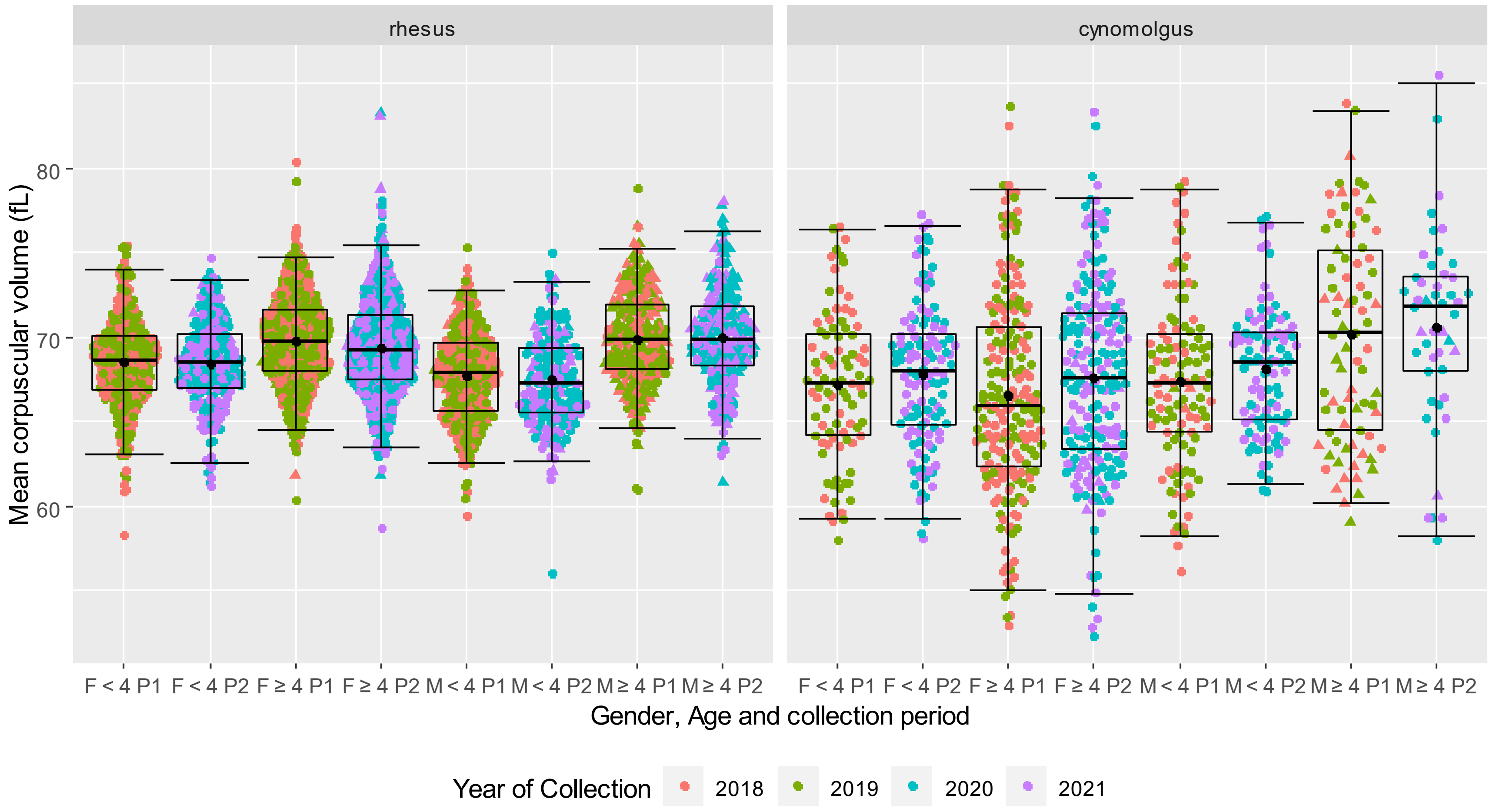

Figure S2 23. Mean Corpuscular Volume according to gender, age and collection period in rhesus and cynomolgus macaques. P1 = 2018 and 2019, P2 = 2020 and 2021. Boxes indicate median and quartile ranges, black point indicates arithmetic mean and error bars indicate the normal range. Round symbols indicate animals with outdoor access and triangles indicate indoor housed animals

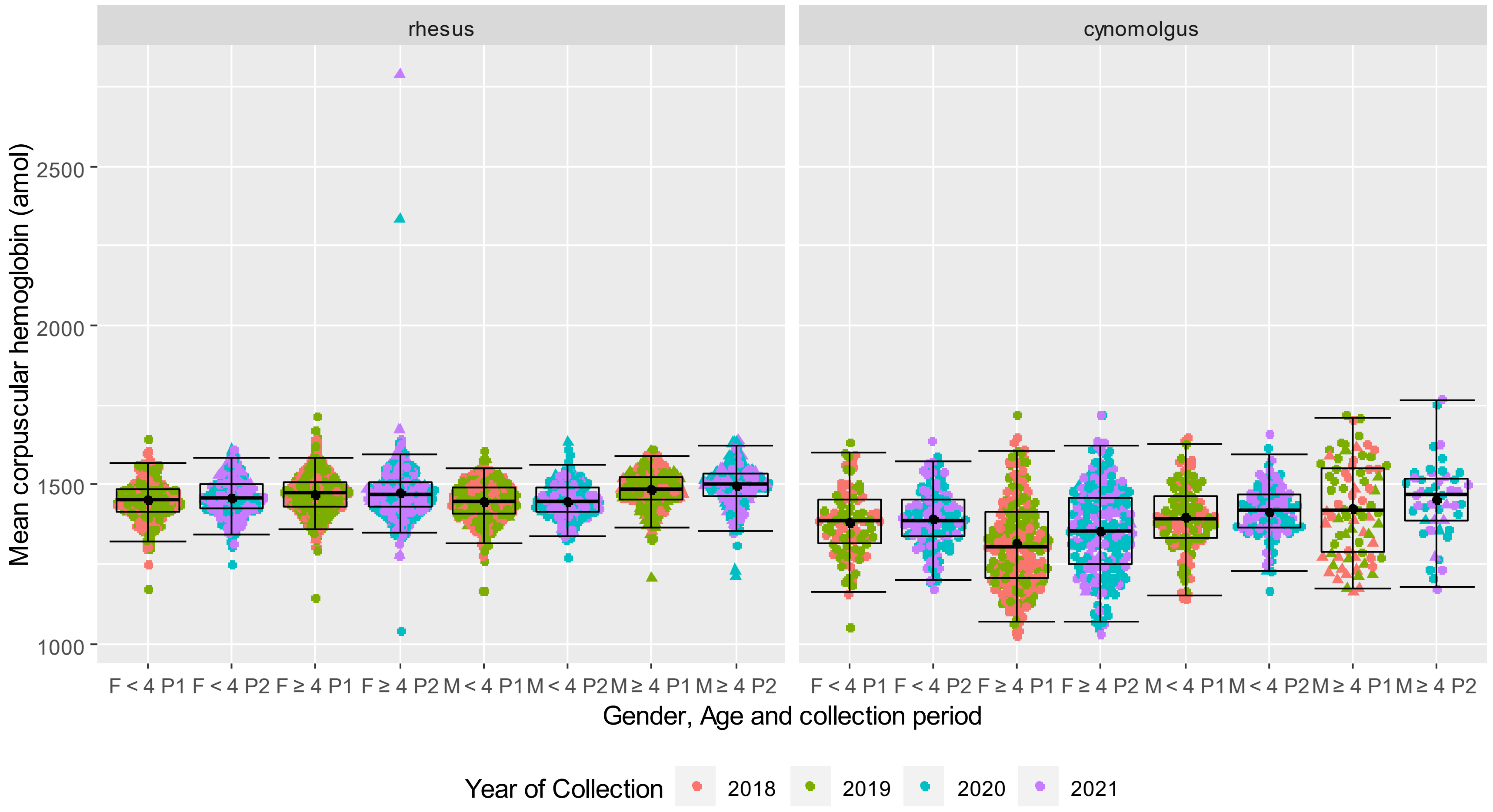

Figure S2 24. Mean Corpuscular Hemoglobin according to gender, age and collection period in rhesus and cynomolgus macaques. P1 = 2018 and 2019, P2 = 2020 and 2021. Boxes indicate median and quartile ranges, black point indicates arithmetic mean and error bars indicate the normal range. Round symbols indicate animals with outdoor access and triangles indicate indoor housed animals

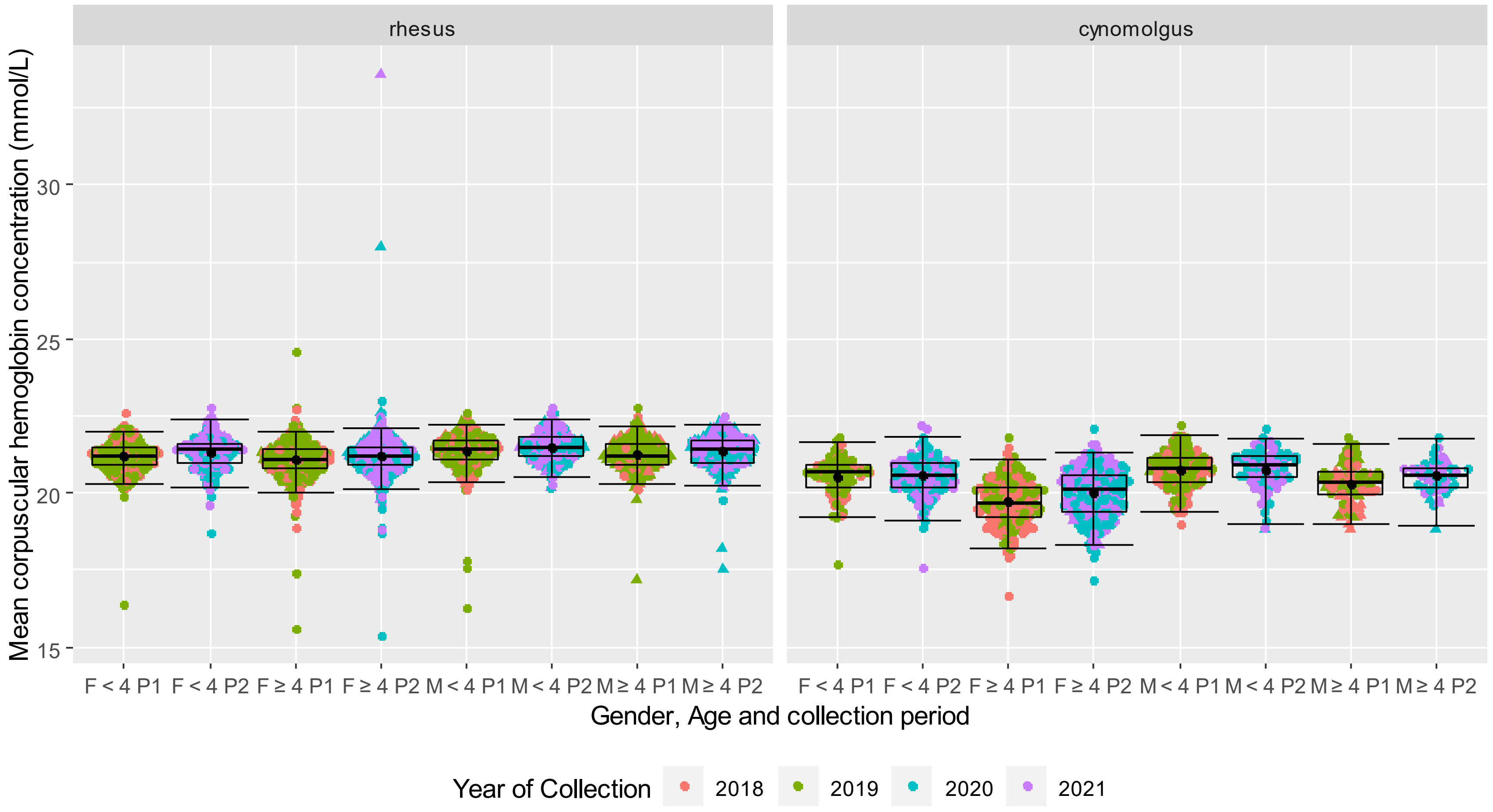

Figure S2 25. Mean Corpuscular Hemoglobin Concentration according to gender, age and collection period in rhesus and cynomolgus macaques. P1 = 2018 and 2019, P2 = 2020 and 2021. Boxes indicate median and quartile ranges, black point indicates arithmetic mean and error bars indicate the normal range. Round symbols indicate animals with outdoor access and triangles indicate indoor housed animals

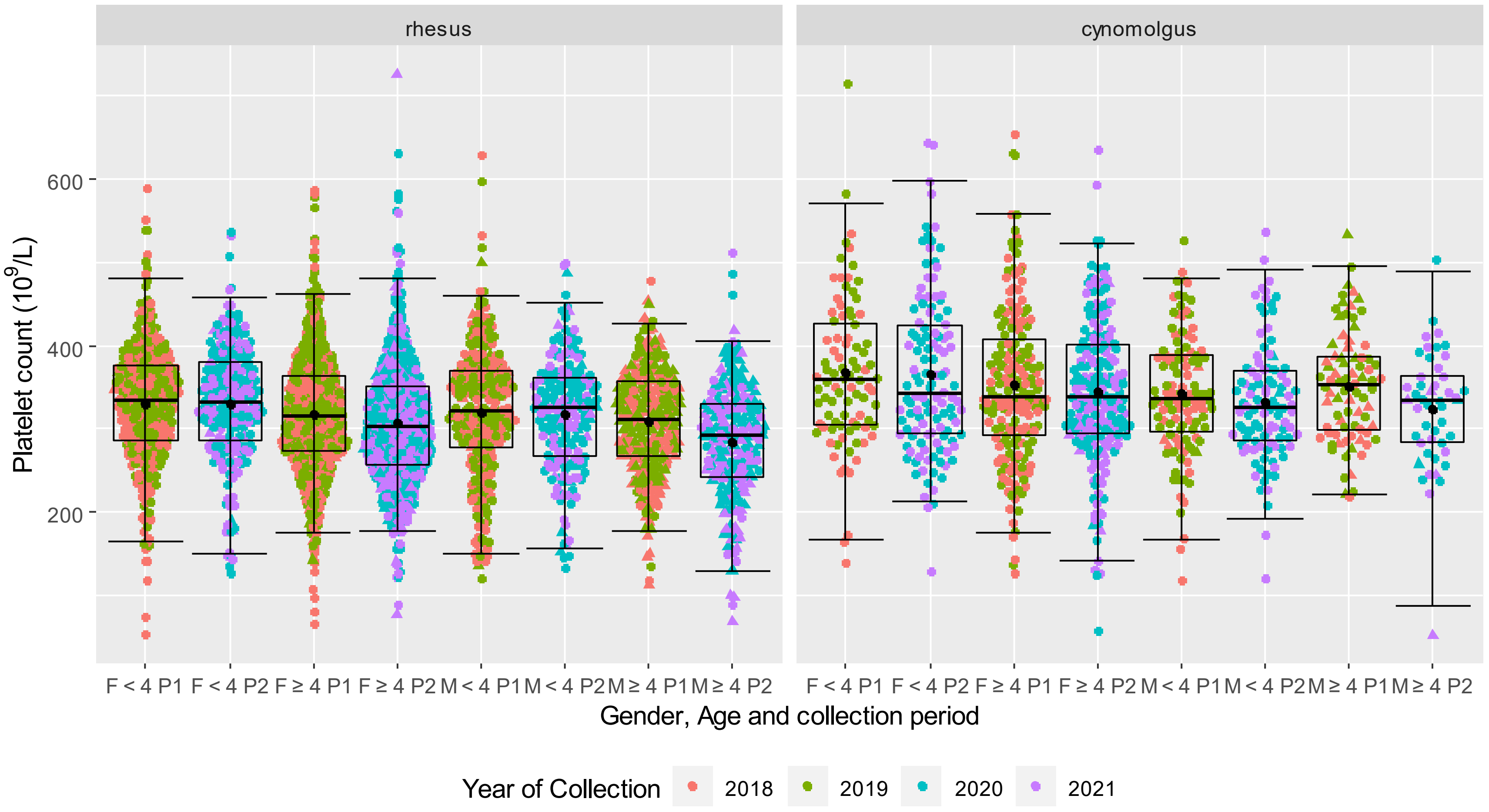

Figure S2 26. Platelet Count according to gender, age and collection period in rhesus and cynomolgus macaques. P1 = 2018 and 2019, P2 = 2020 and 2021. Boxes indicate median and quartile ranges, black point indicates arithmetic mean and error bars indicate the normal range. Round symbols indicate animals with outdoor access and triangles indicate indoor housed animals

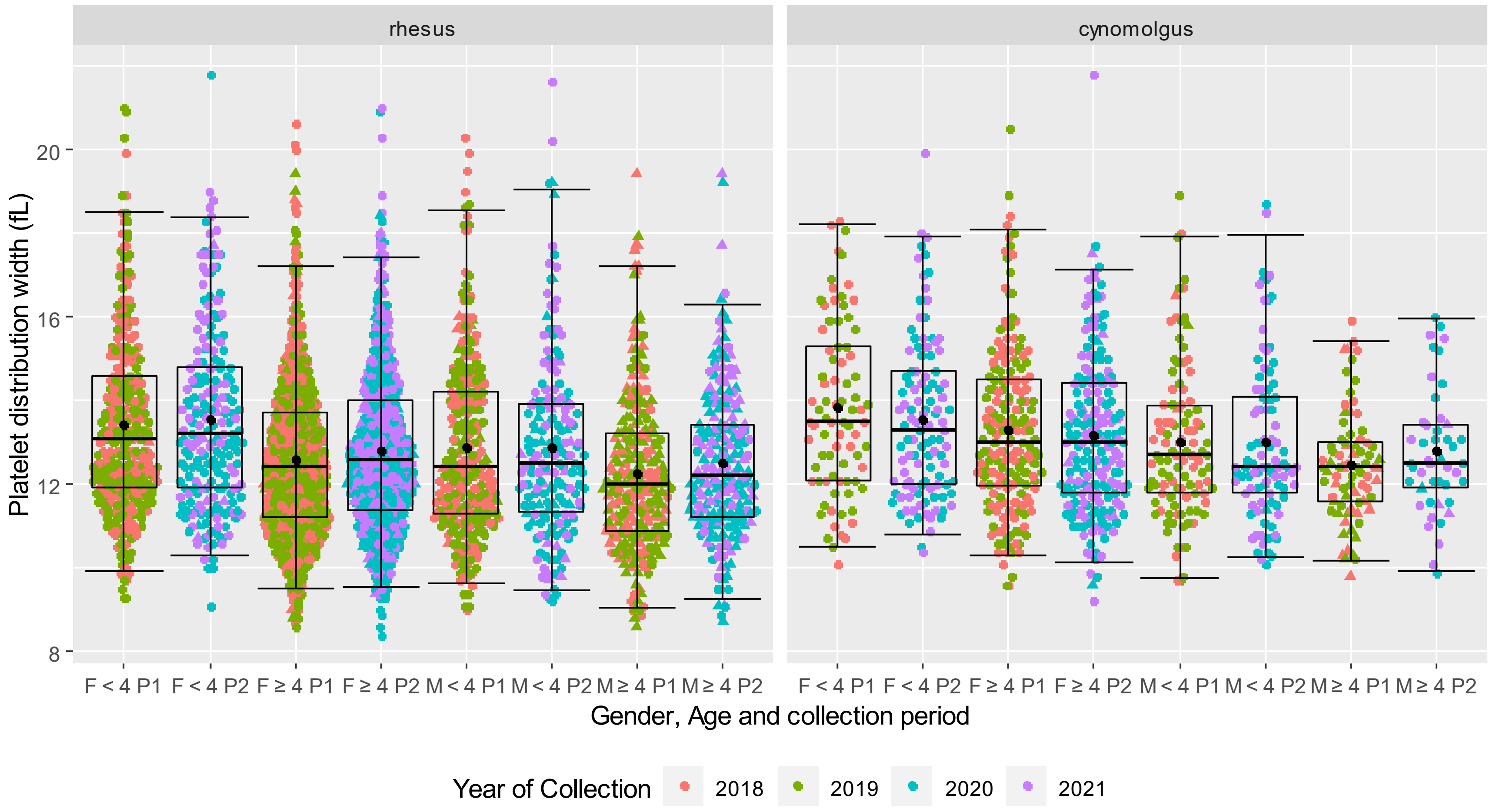

Figure S2 27. Platelet Distribution Width according to gender, age and collection period in rhesus and cynomolgus macaques. P1 = 2018 and 2019, P2 = 2020 and 2021. Boxes indicate median and quartile ranges, black point indicates arithmetic mean and error bars indicate the normal range. Round symbols indicate animals with outdoor access and triangles indicate indoor housed animals

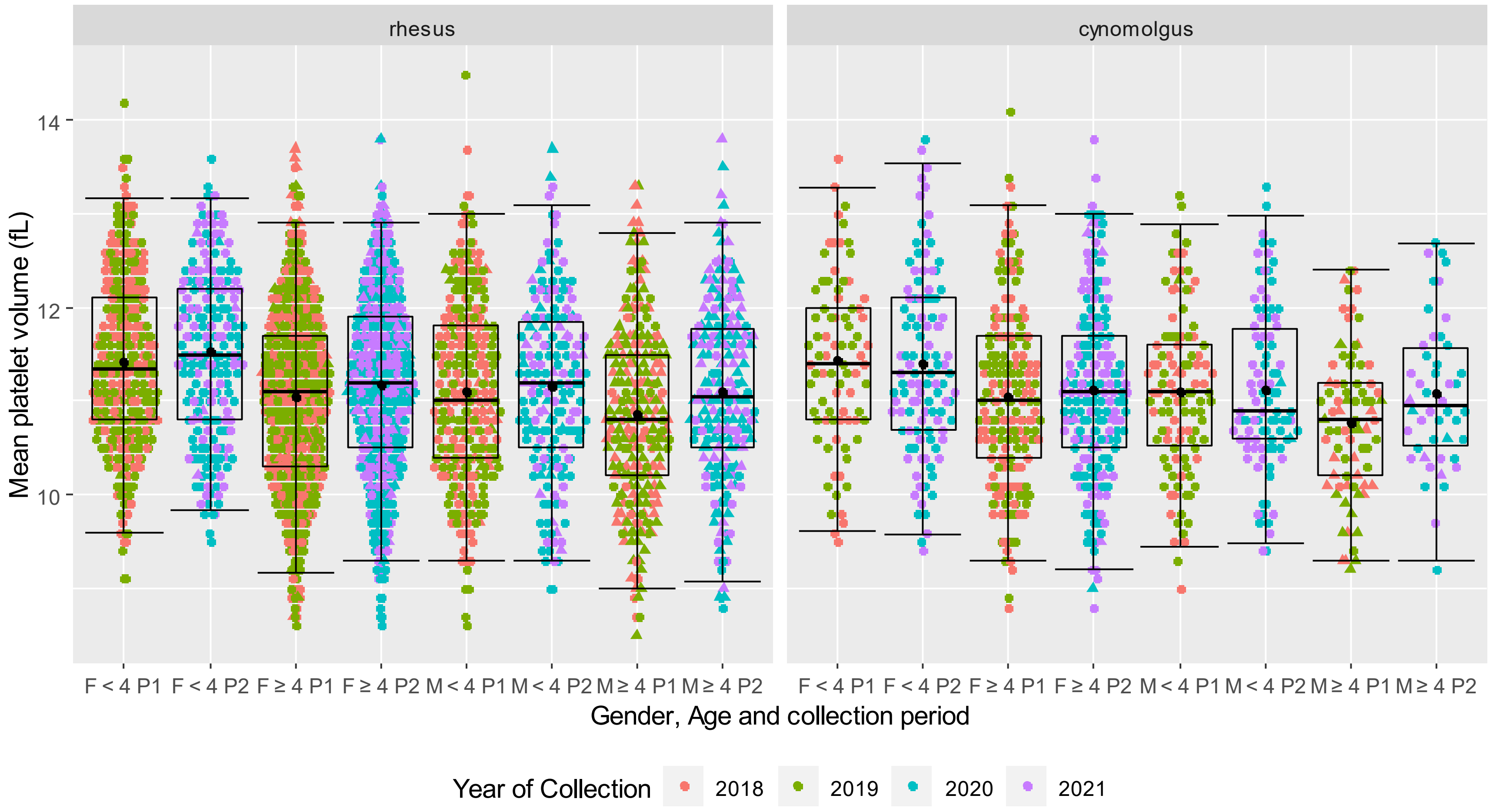

Figure S2 28. Mean Platelet Volume according to gender, age and collection period in rhesus and cynomolgus macaques. P1 = 2018 and 2019, P2 = 2020 and 2021. Boxes indicate median and quartile ranges, black point indicates arithmetic mean and error bars indicate the normal range. Round symbols indicate animals with outdoor access and triangles indicate indoor housed animals

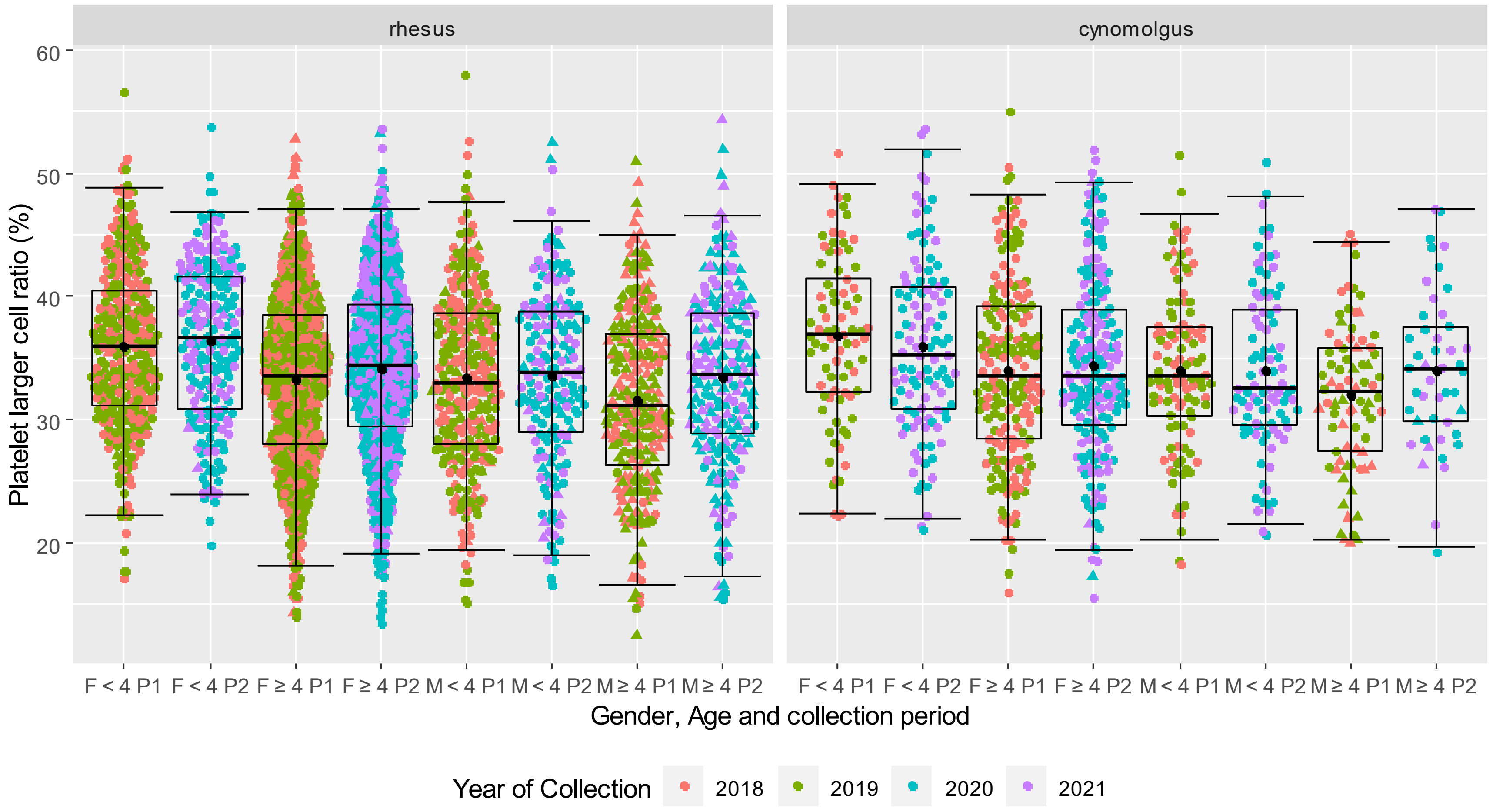

Figure S2 29. Platelet Larger Cell Ratio according to gender, age and collection period in rhesus and cynomolgus macaques. P1 = 2018 and 2019, P2 = 2020 and 2021. Boxes indicate median and quartile ranges, black point indicates arithmetic mean and error bars indicate the normal range. Round symbols indicate animals with outdoor access and triangles indicate indoor housed animals

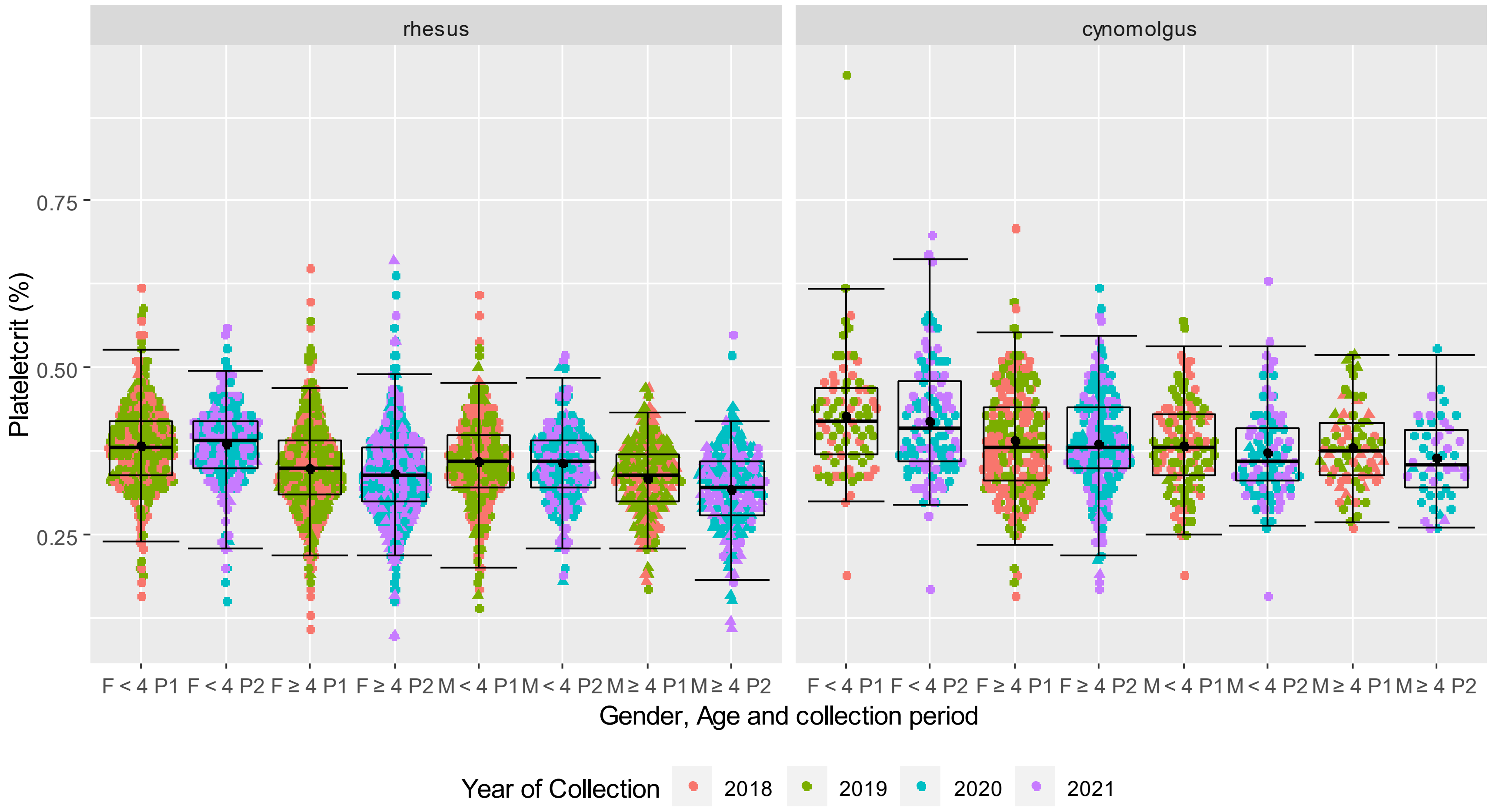

Figure S2 30. Plateletcrit according to gender, age and collection period in rhesus and cynomolgus macaques. P1 = 2018 and 2019, P2 = 2020 and 2021. Boxes indicate median and quartile ranges, black point indicates arithmetic mean and error bars indicate the normal range. Round symbols indicate animals with outdoor access and triangles indicate indoor housed animals

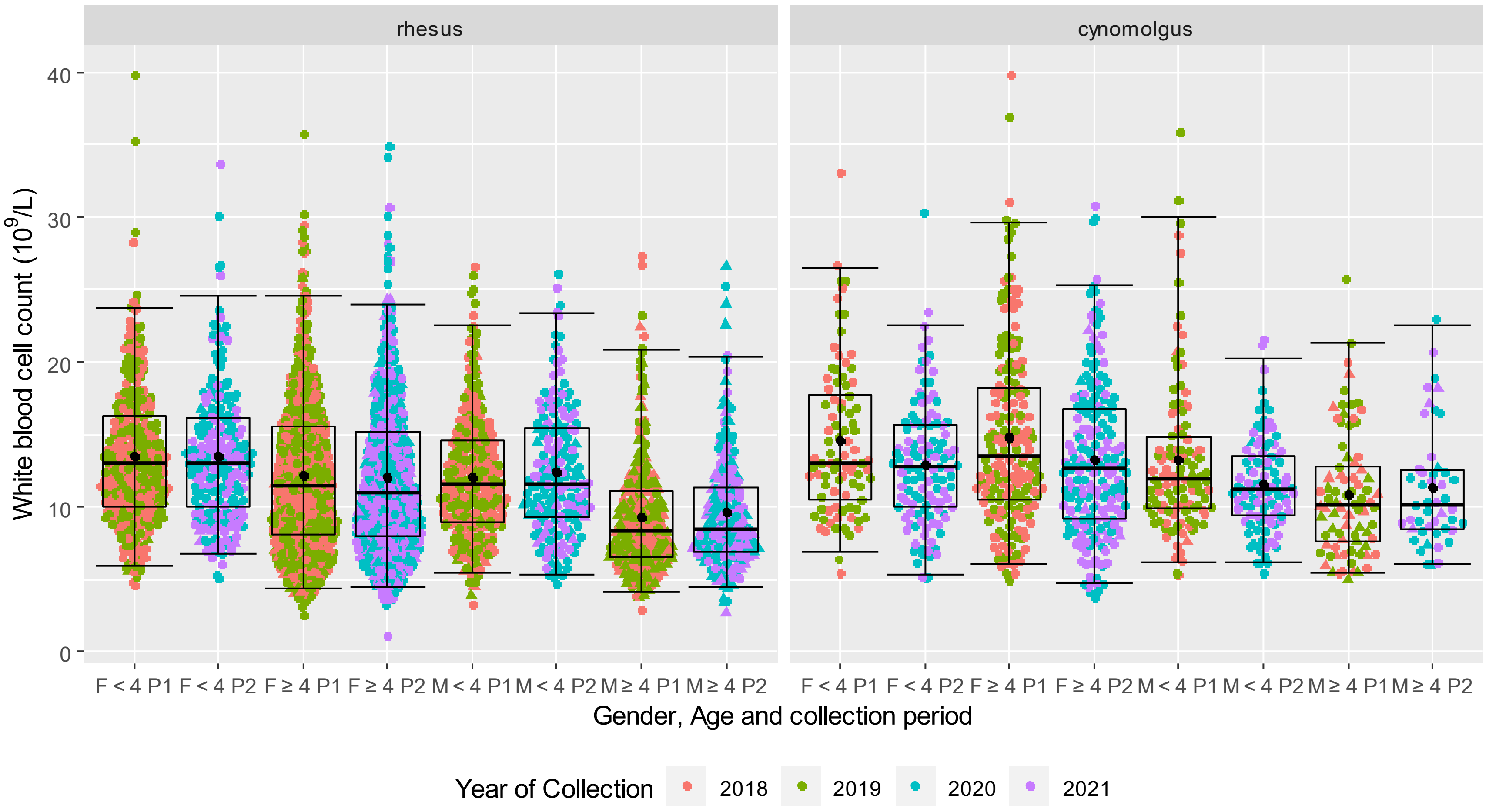

Figure S2 31. White Blood Cell Count according to gender, age and collection period in rhesus and cynomolgus macaques. P1 = 2018 and 2019, P2 = 2020 and 2021. Boxes indicate median and quartile ranges, black point indicates arithmetic mean and error bars indicate the normal range. Round symbols indicate animals with outdoor access and triangles indicate indoor housed animals

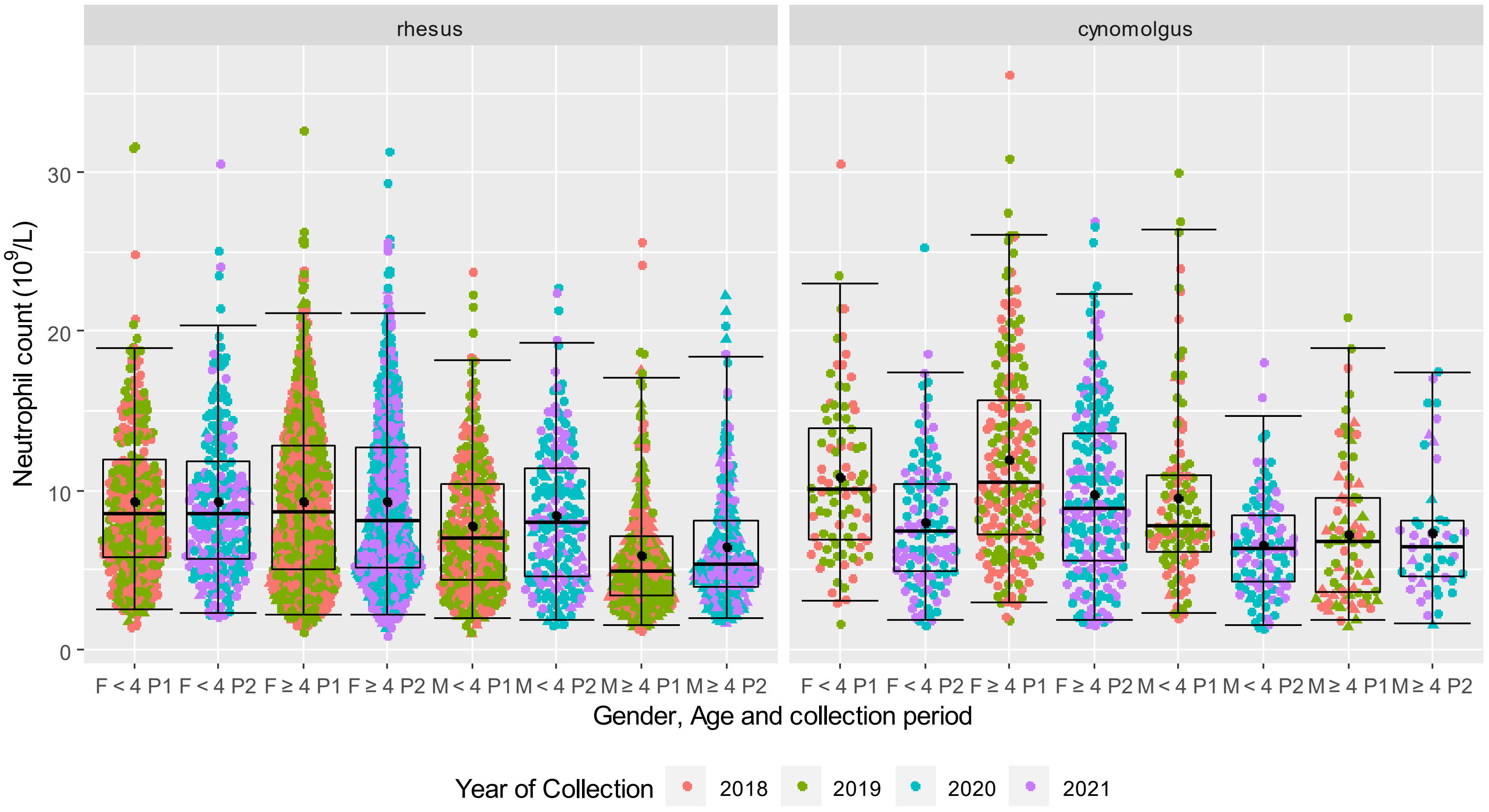

Figure S2 32. Neutrophil Count according to gender, age and collection period in rhesus and cynomolgus macaques. P1 = 2018 and 2019, P2 = 2020 and 2021. Boxes indicate median and quartile ranges, black point indicates arithmetic mean and error bars indicate the normal range. Round symbols indicate animals with outdoor access and triangles indicate indoor housed animals

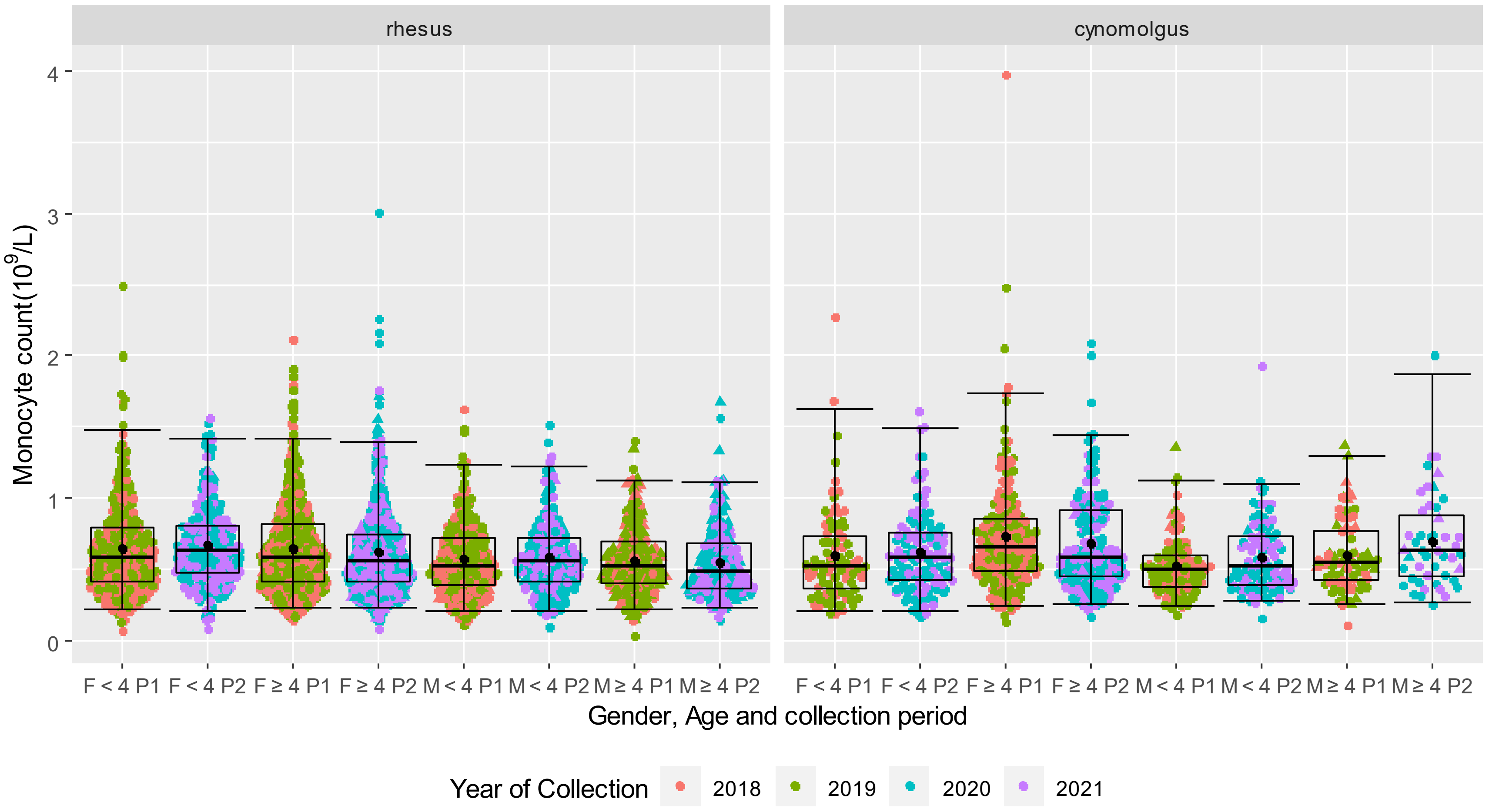

Figure S2 33. Monocyte Count according to gender, age and collection period in rhesus and cynomolgus macaques. P1 = 2018 and 2019, P2 = 2020 and 2021. Boxes indicate median and quartile ranges, black point indicates arithmetic mean and error bars indicate the normal range. Round symbols indicate animals with outdoor access and triangles indicate indoor housed animals

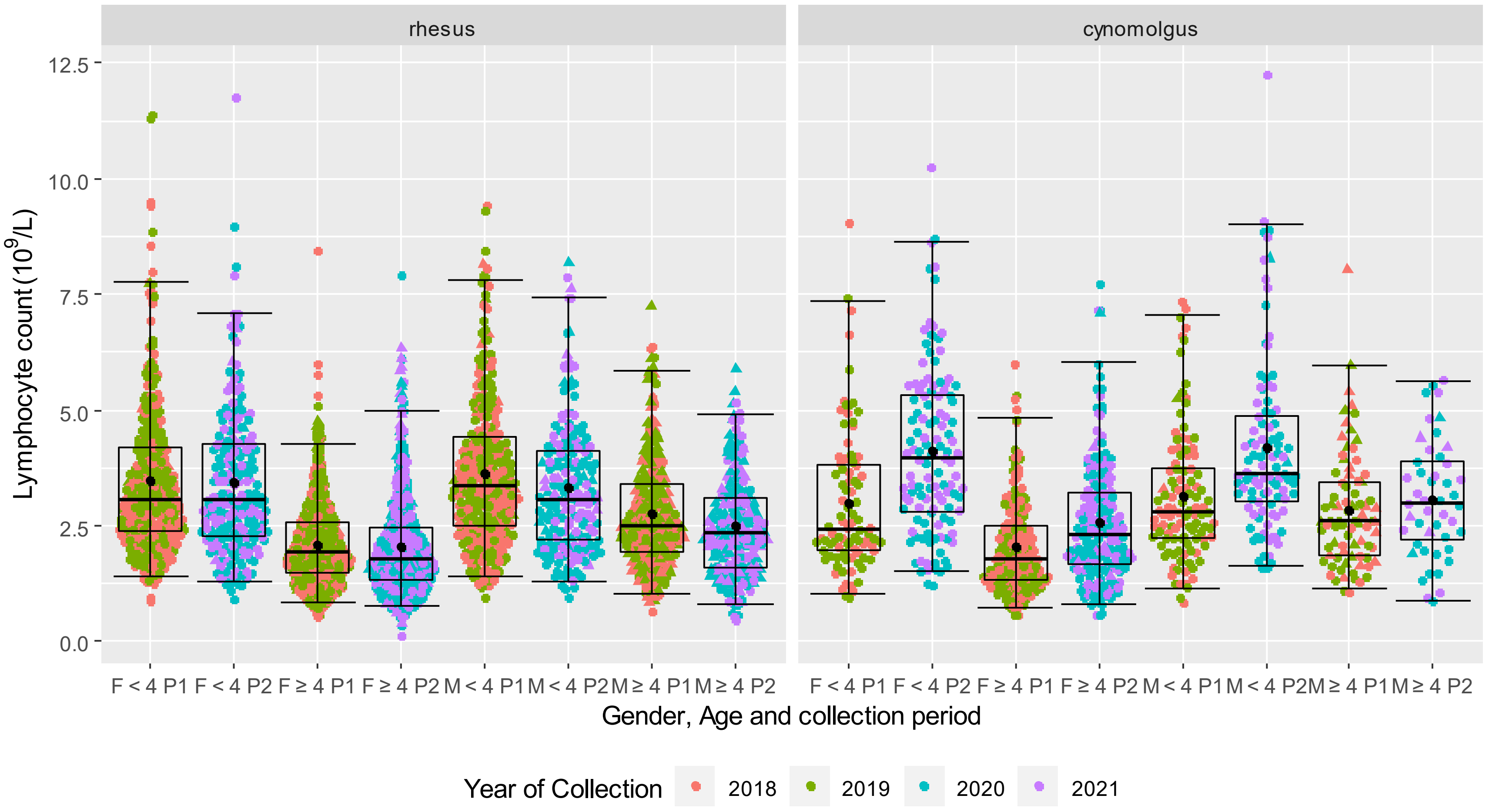

Figure S2 34. Lymphocyte Count according to gender, age and collection period in rhesus and cynomolgus macaques. P1 = 2018 and 2019, P2 = 2020 and 2021. Boxes indicate median and quartile ranges, black point indicates arithmetic mean and error bars indicate the normal range. Round symbols indicate animals with outdoor access and triangles indicate indoor housed animals

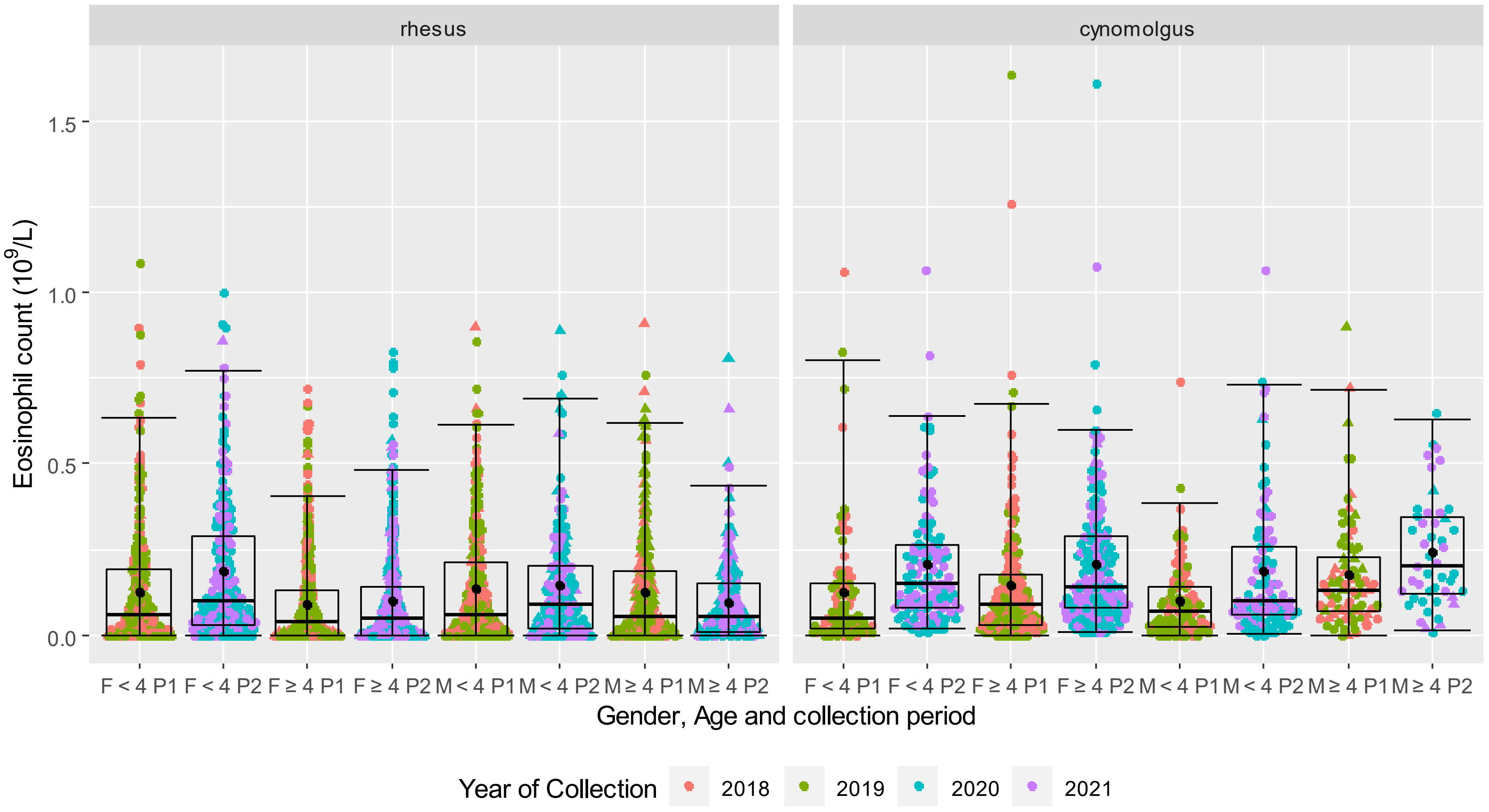

Figure S2 35. Eosinophil Count according to gender, age and collection period in rhesus and cynomolgus macaques. P1 = 2018 and 2019, P2 = 2020 and 2021. Boxes indicate median and quartile ranges, black point indicates arithmetic mean and error bars indicate the normal range. Round symbols indicate animals with outdoor access and triangles indicate indoor housed animals

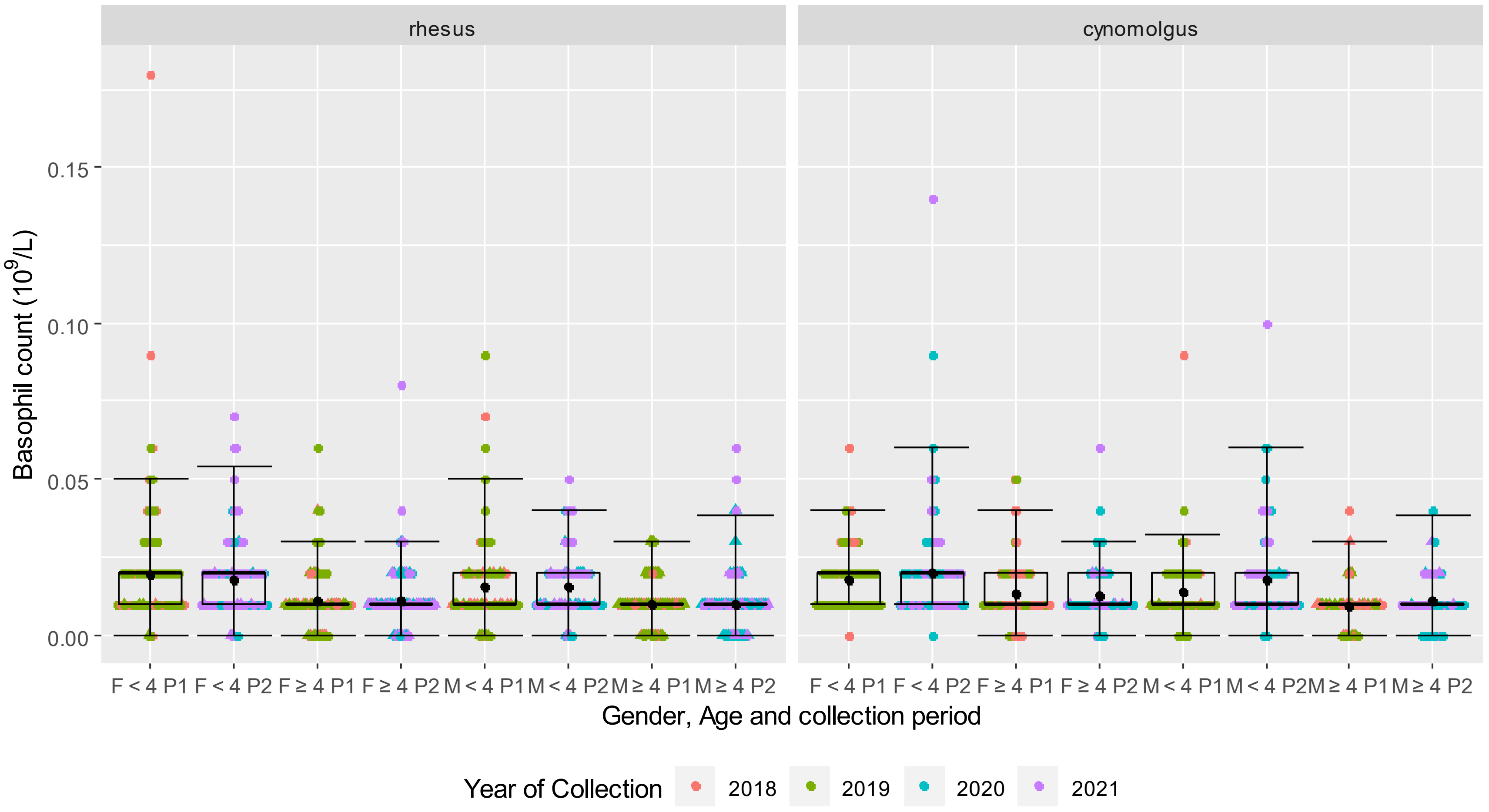

Figure S2 36. Basophil Count according to gender, age and collection period in rhesus and cynomolgus macaques. P1 = 2018 and 2019, P2 = 2020 and 2021. Boxes indicate median and quartile ranges, black point indicates arithmetic mean and error bars indicate the normal range. Round symbols indicate animals with outdoor access and triangles indicate indoor housed animals
