# Supplementary material for: Reference Intervals and Percentiles for Hematologic and Serum Biochemical Values in Captive Bred Rhesus (Macaca mulatta) and Cynomolgus Macaques (Macaca fascicularis)
Source: Animals (Basel). 2023 Jan 28;13(3):445. doi: 10.3390/ani13030445 (PMC9913310; doi:10.3390/ani13030445)
Supplement: Supplementary file 1 [file animals-13-00445-s001.zip › Figure S3.pdf]

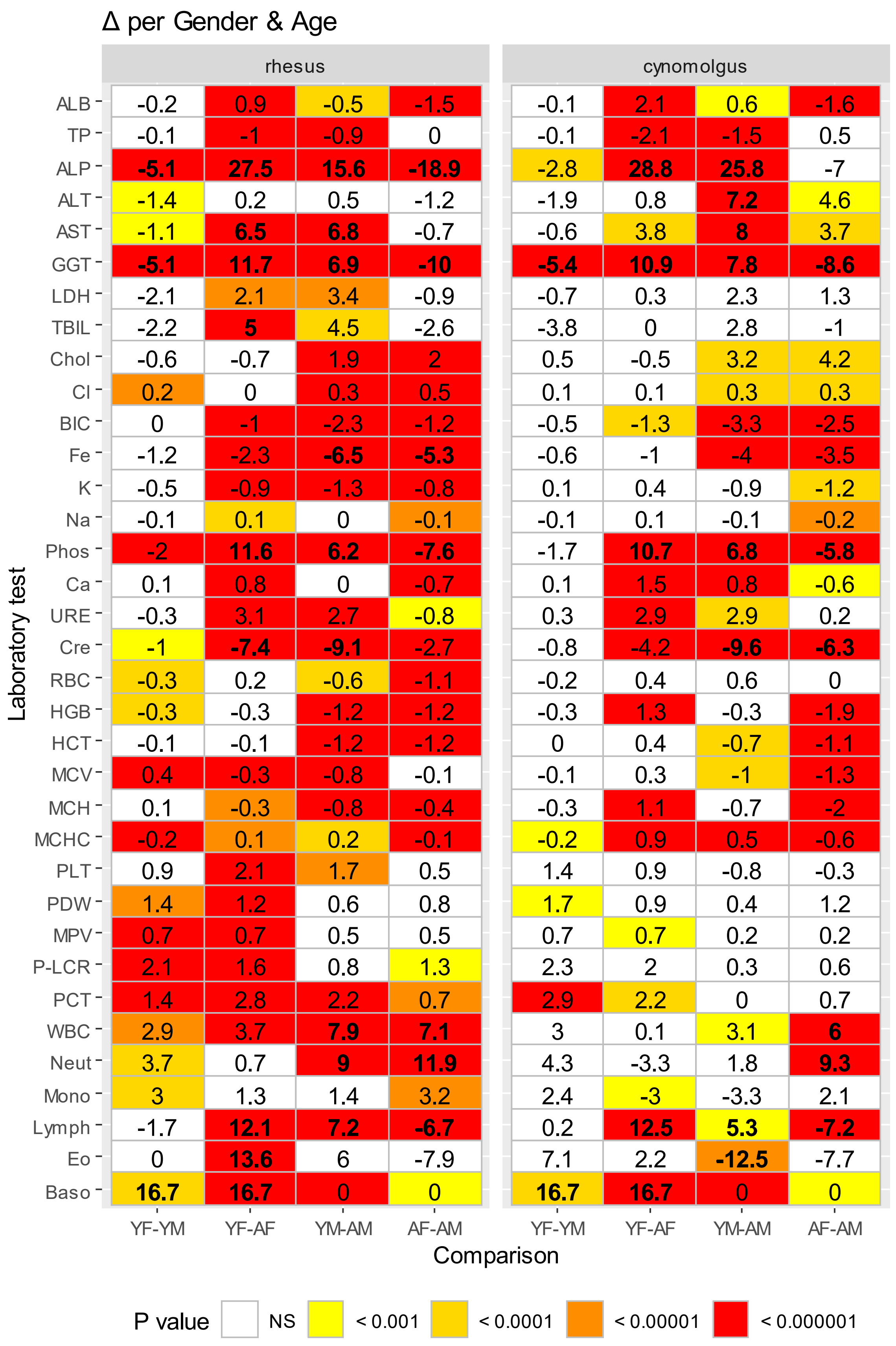

Figure S3A. Comparisons between gender and age in rhesus and cynomolgus macaques, numbers indicate percentual differences (Delta) between, Delta values greater than 5% are in bold. YF-YM = Young Females and Males, YF-AF = Young and Adult Females, YM-AM = Young and Adult Males, AF-AM = Adult Females and Males.

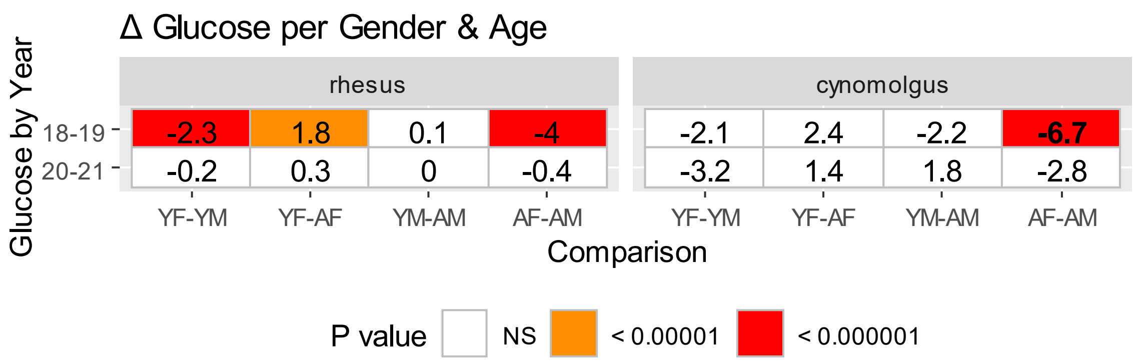

Figure S3B. Glucose comparison between 2018-2019 and 2020-201, gender and age in rhesus and cynomolgus macaques, numbers indicate percentual differences (Delta) between, Delta values greater than 5% are in bold. YF-YM = Young Females and Males, YF-AF = Young and Adult Females, YM-AM = Young and Adult Males, AF-AM = Adult Females and Males.
