# Supplementary material for: Reference Intervals and Percentiles for Hematologic and Serum Biochemical Values in Captive Bred Rhesus (Macaca mulatta) and Cynomolgus Macaques (Macaca fascicularis)
Source: Animals (Basel). 2023 Jan 28;13(3):445. doi: 10.3390/ani13030445 (PMC9913310; doi:10.3390/ani13030445)
Supplement: Supplementary file 1 [file animals-13-00445-s001.zip › Figure S4.pdf]

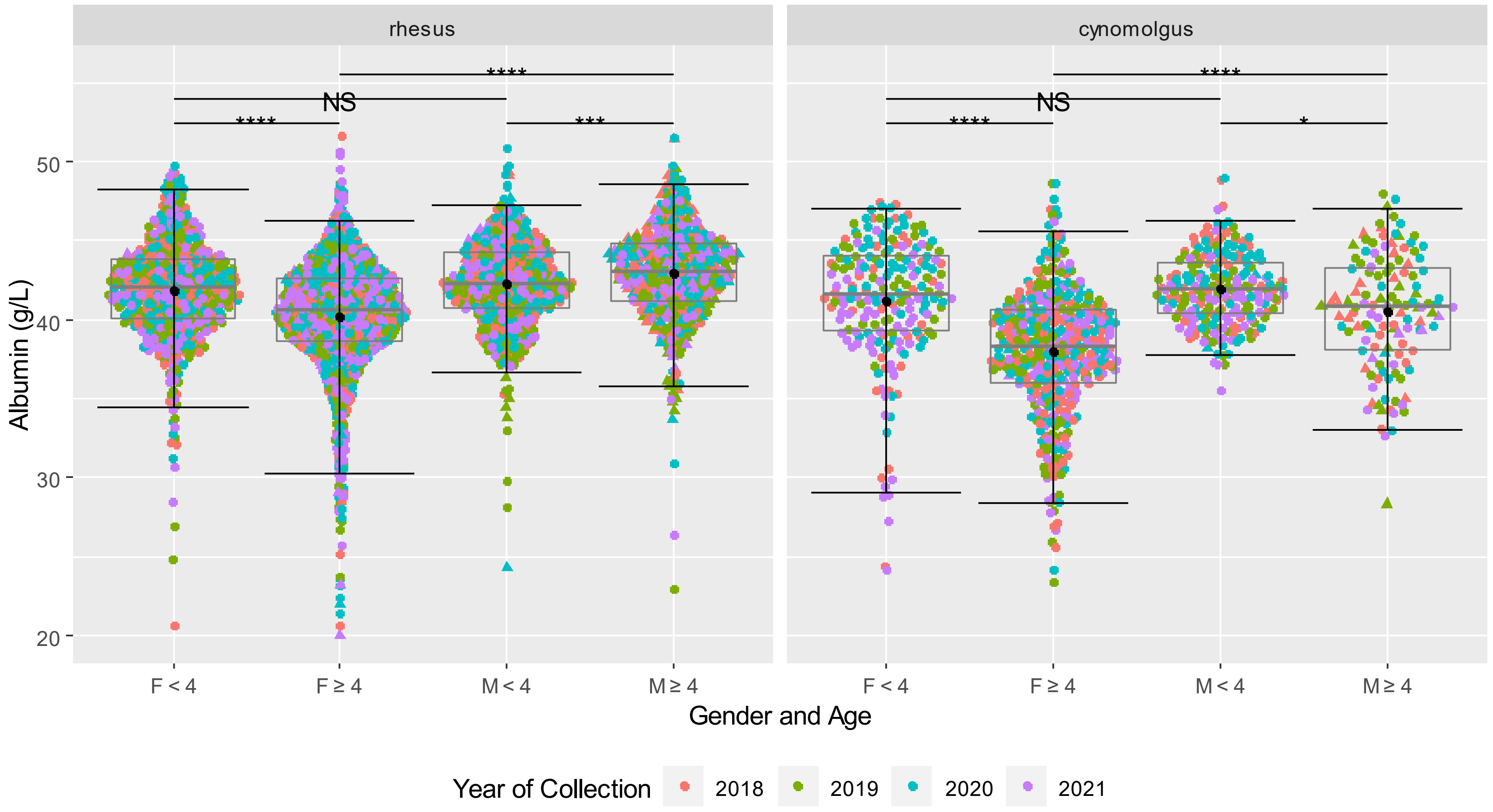

Figure S4 1. Albumin levels according to gender and age in rhesus and cynomolgus macaques. Boxes indicate median and quartile ranges, black point indicates arithmetic mean and error bars indicate the normal range. Round symbols indicate animals with outdoor access and triangles indicate indoor housed animals

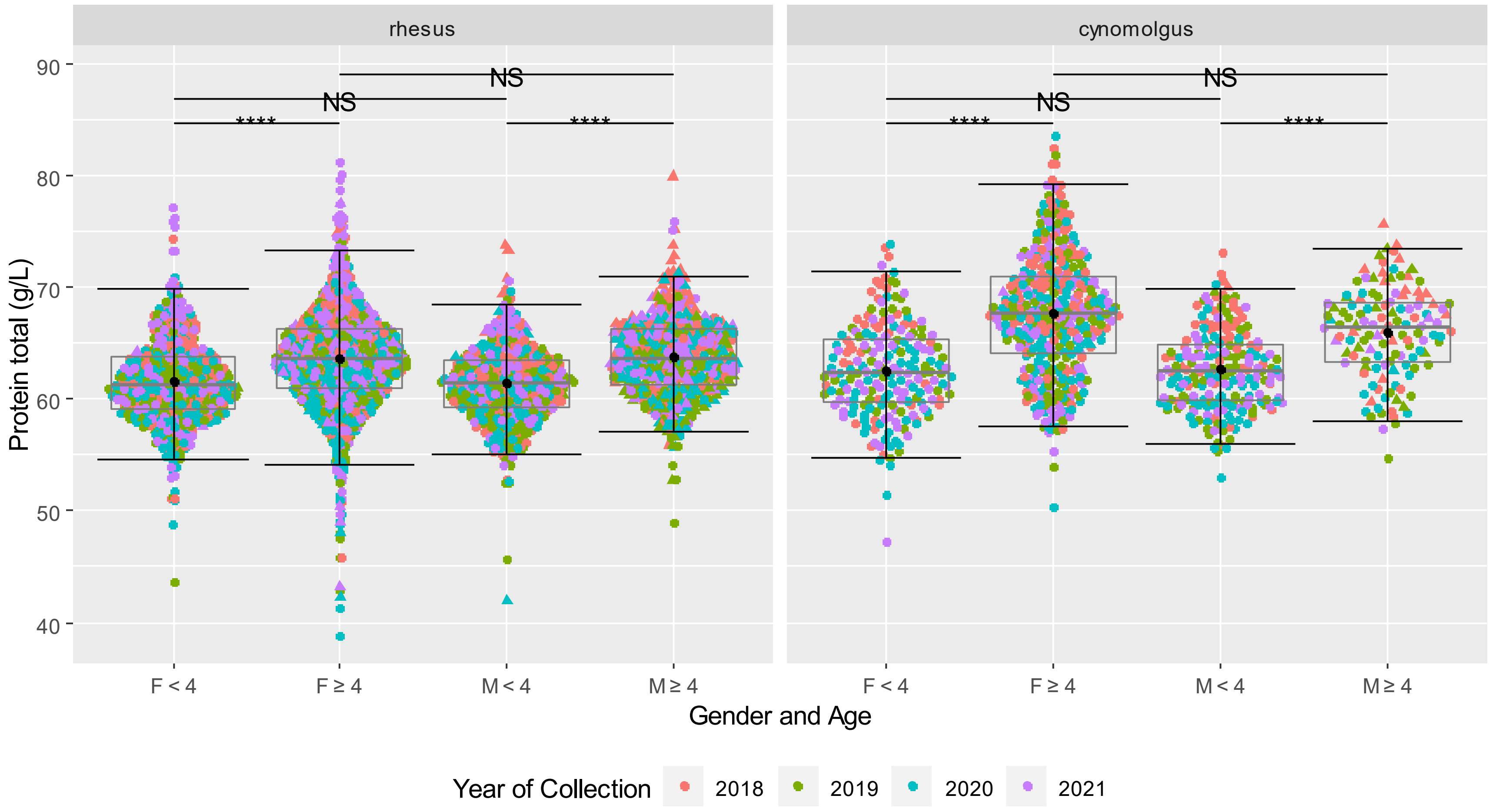

Figure S4 2. Total Protein levels according to gender and age in rhesus and cynomolgus macaques. Boxes indicate median and quartile ranges, black point indicates arithmetic mean and error bars indicate the normal range. Round symbols indicate animals with outdoor access and triangles indicate indoor housed animals

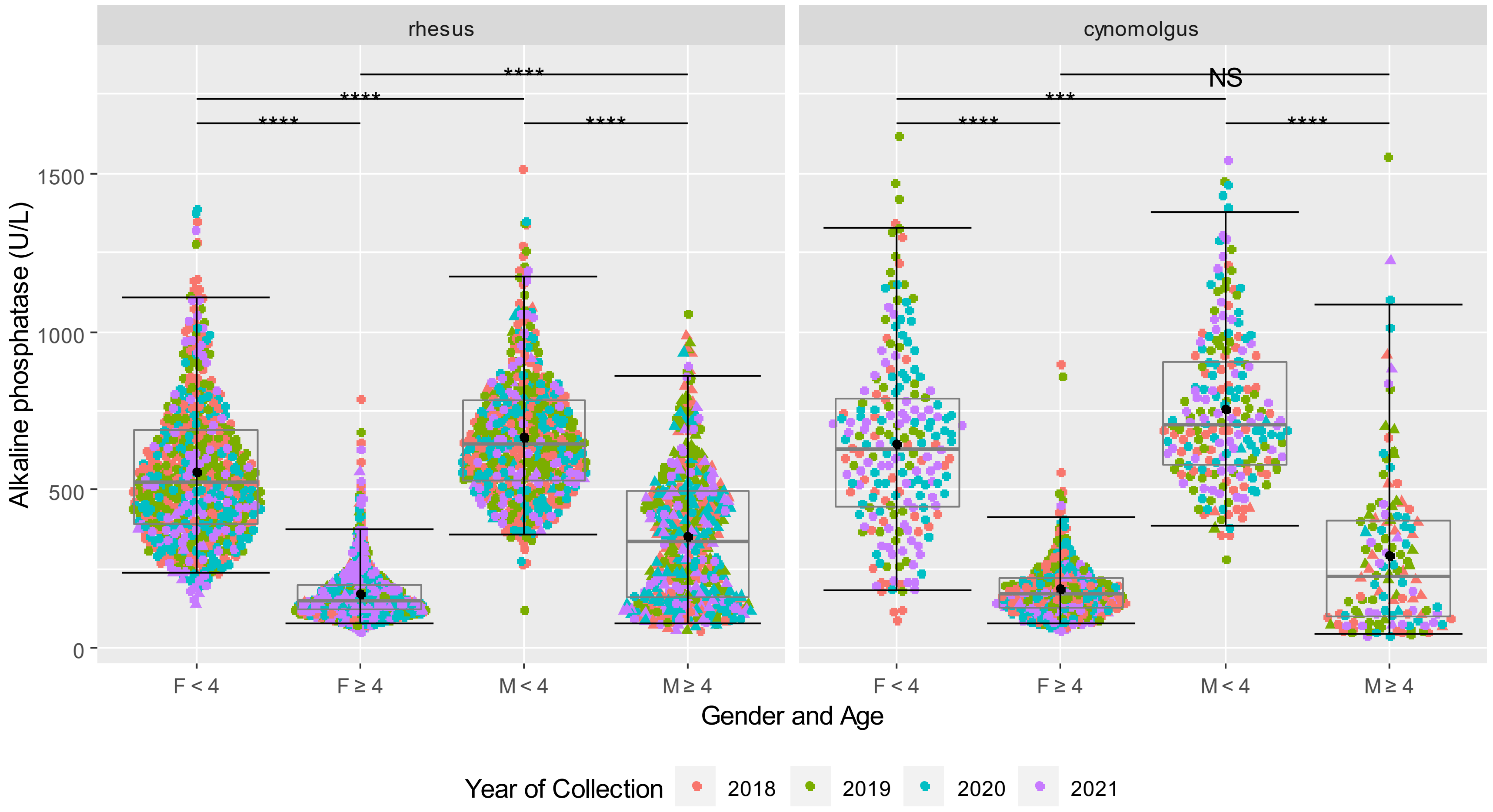

Figure S4 3. Alkaline Phosphatase levels according to year of collection in rhesus and cynomolgus macaques. Boxes indicate median and quartile ranges, black point indicates arithmetic mean and error bars indicate the normal range. Round symbols indicate animals with outdoor access and triangles indicate indoor housed animals

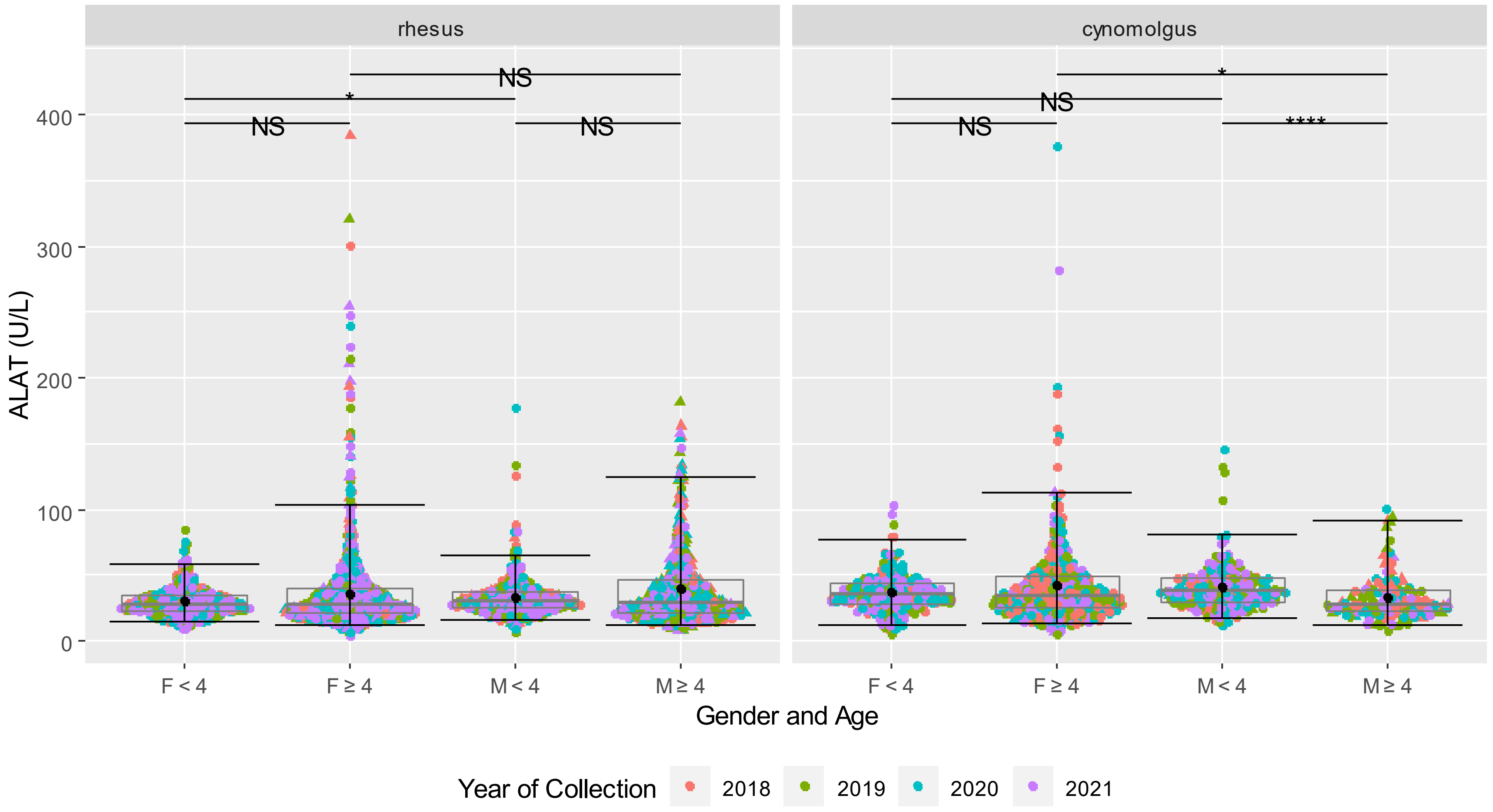

Figure S4 4. ALAT levels according to gender and age in rhesus and cynomolgus macaques. Boxes indicate median and quartile ranges, black point indicates arithmetic mean and error bars indicate the normal range. Round symbols indicate animals with outdoor access and triangles indicate indoor housed animals

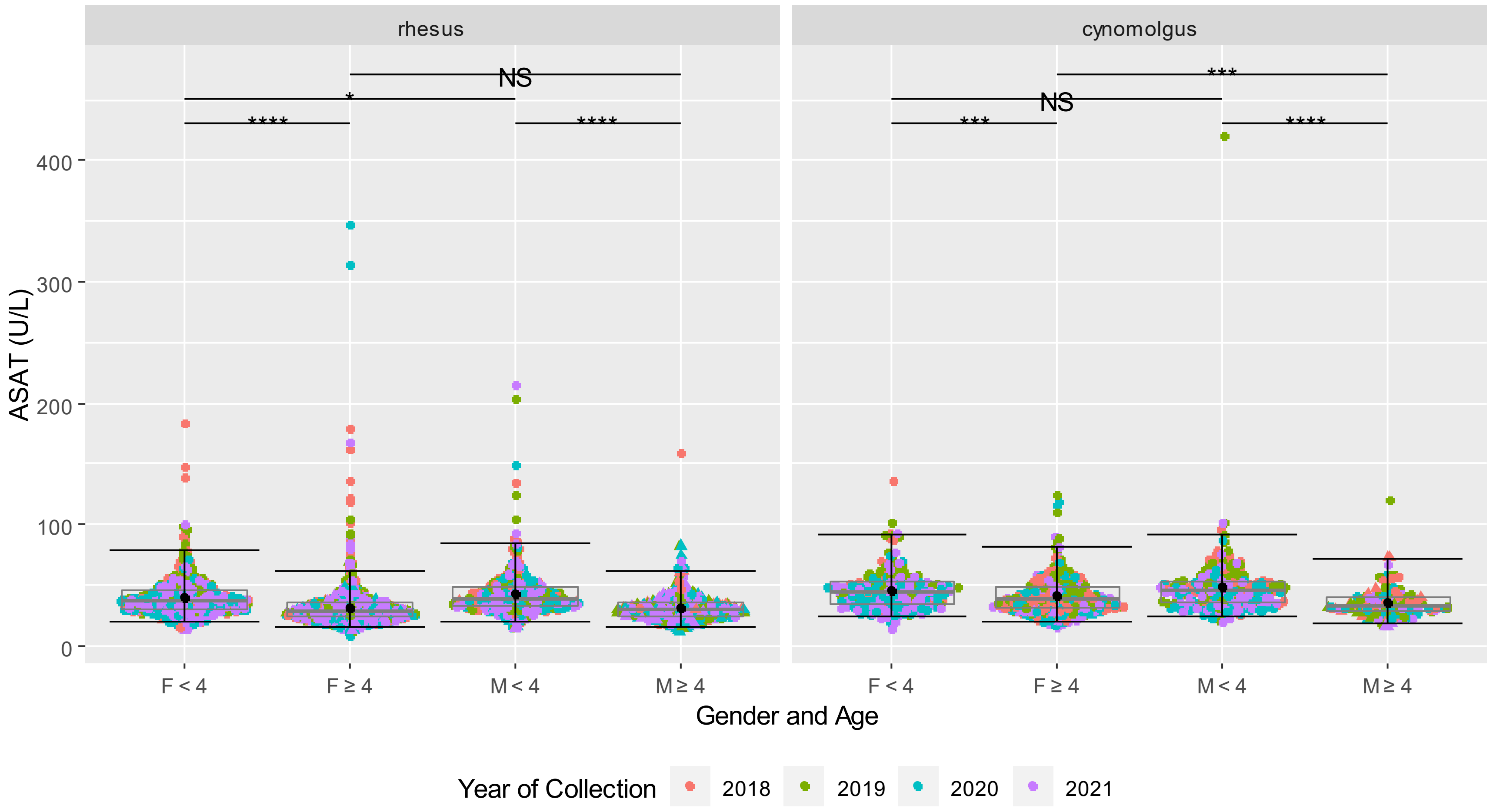

Figure S4 5. ASAT levels according to gender and age in rhesus and cynomolgus macaques. Boxes indicate median and quartile ranges, black point indicates arithmetic mean and error bars indicate the normal range. Round symbols indicate animals with outdoor access and triangles indicate indoor housed animals

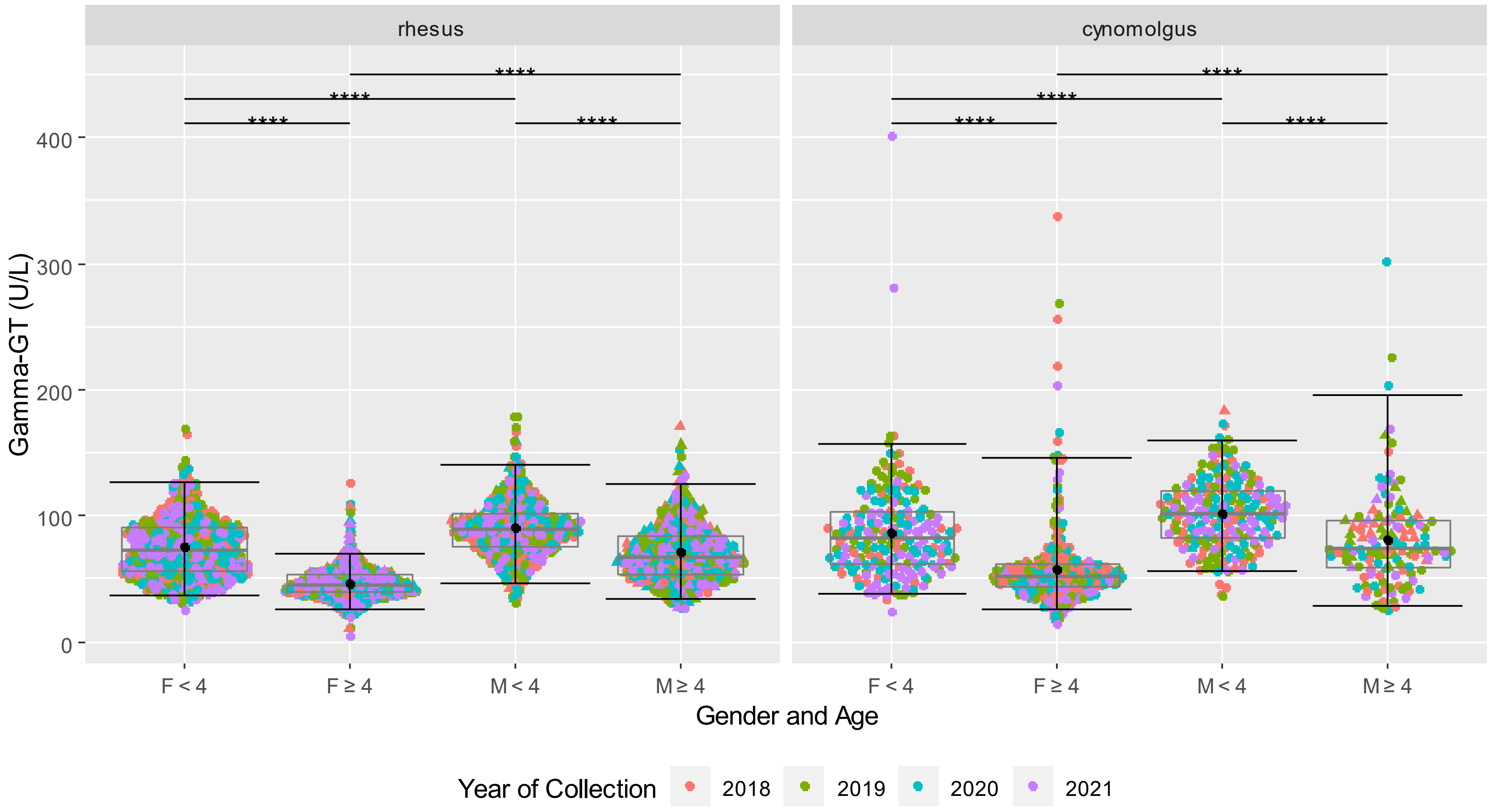

Figure S4 6. Gamma-GT levels according to gender and age in rhesus and cynomolgus macaques. Boxes indicate median and quartile ranges, black point indicates arithmetic mean and error bars indicate the normal range. Round symbols indicate animals with outdoor access and triangles indicate indoor housed animals

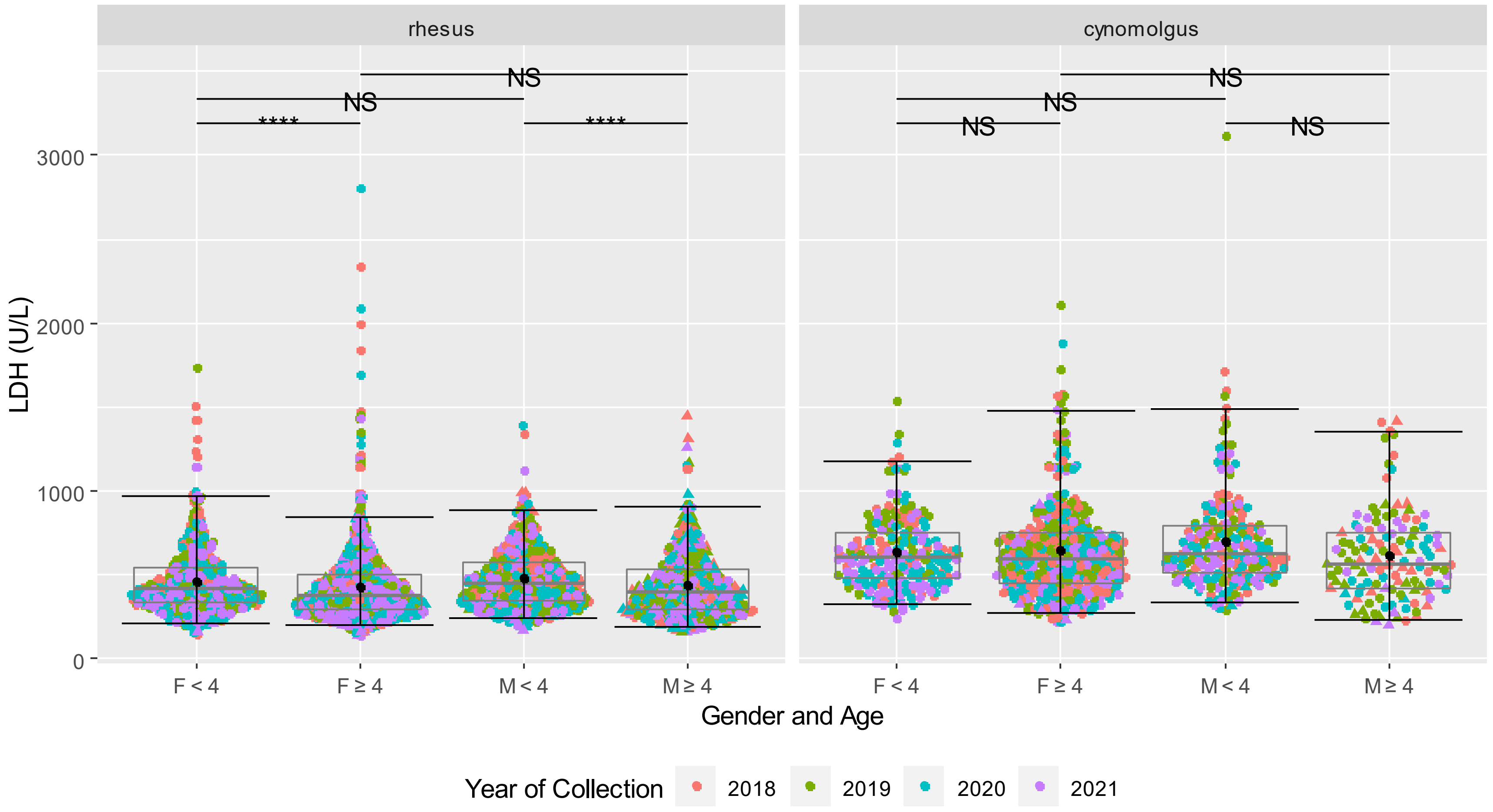

Figure S4 7. LDH levels according to gender and age in rhesus and cynomolgus macaques. Boxes indicate median and quartile ranges, black point indicates arithmetic mean and error bars indicate the normal range. Round symbols indicate animals with outdoor access and triangles indicate indoor housed animals

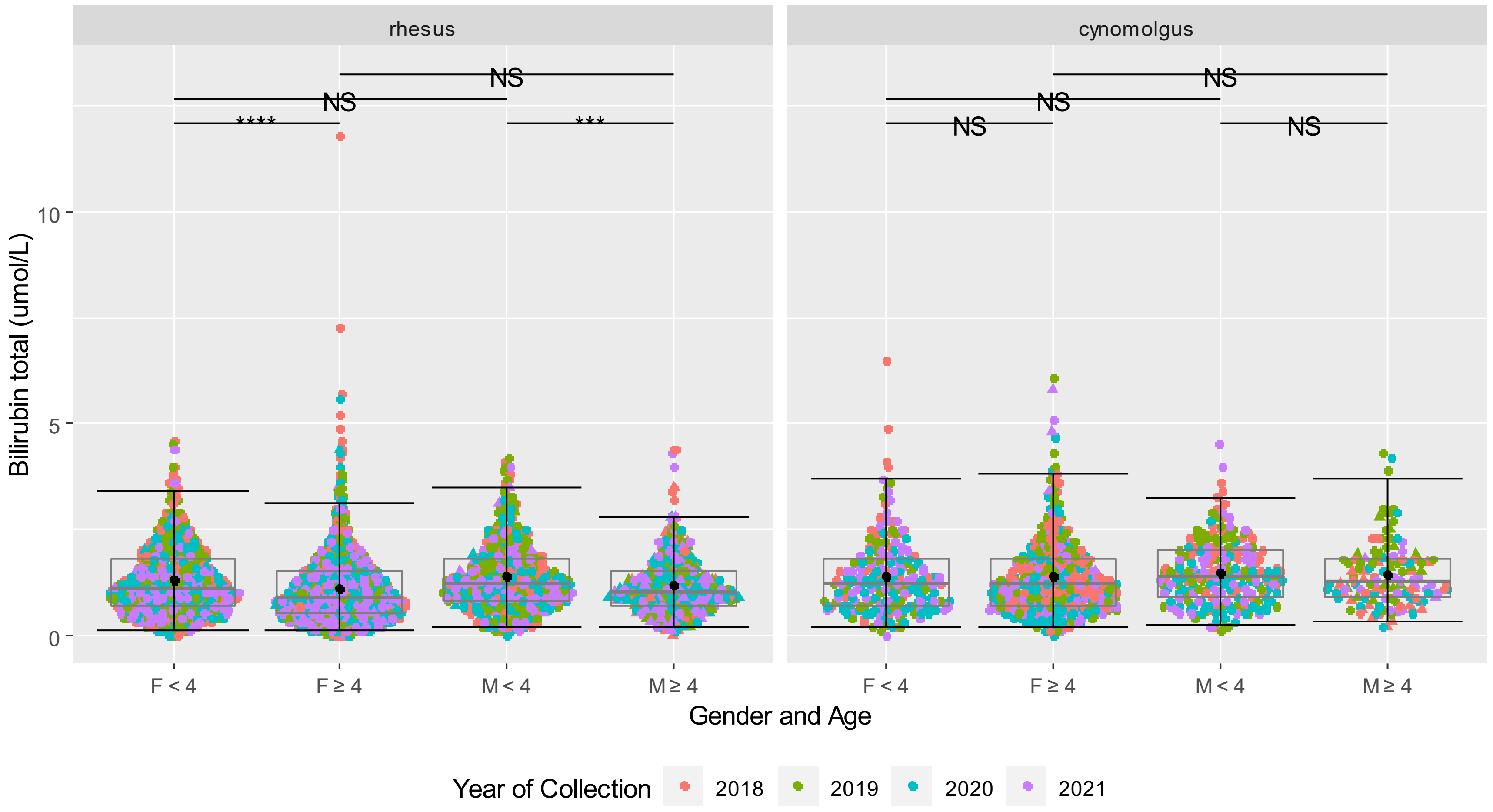

Figure S4 8. Total Bilirubin levels according to gender and age in rhesus and cynomolgus macaques. Boxes indicate median and quartile ranges, black point indicates arithmetic mean and error bars indicate the normal range. Round symbols indicate animals with outdoor access and triangles indicate indoor housed animals

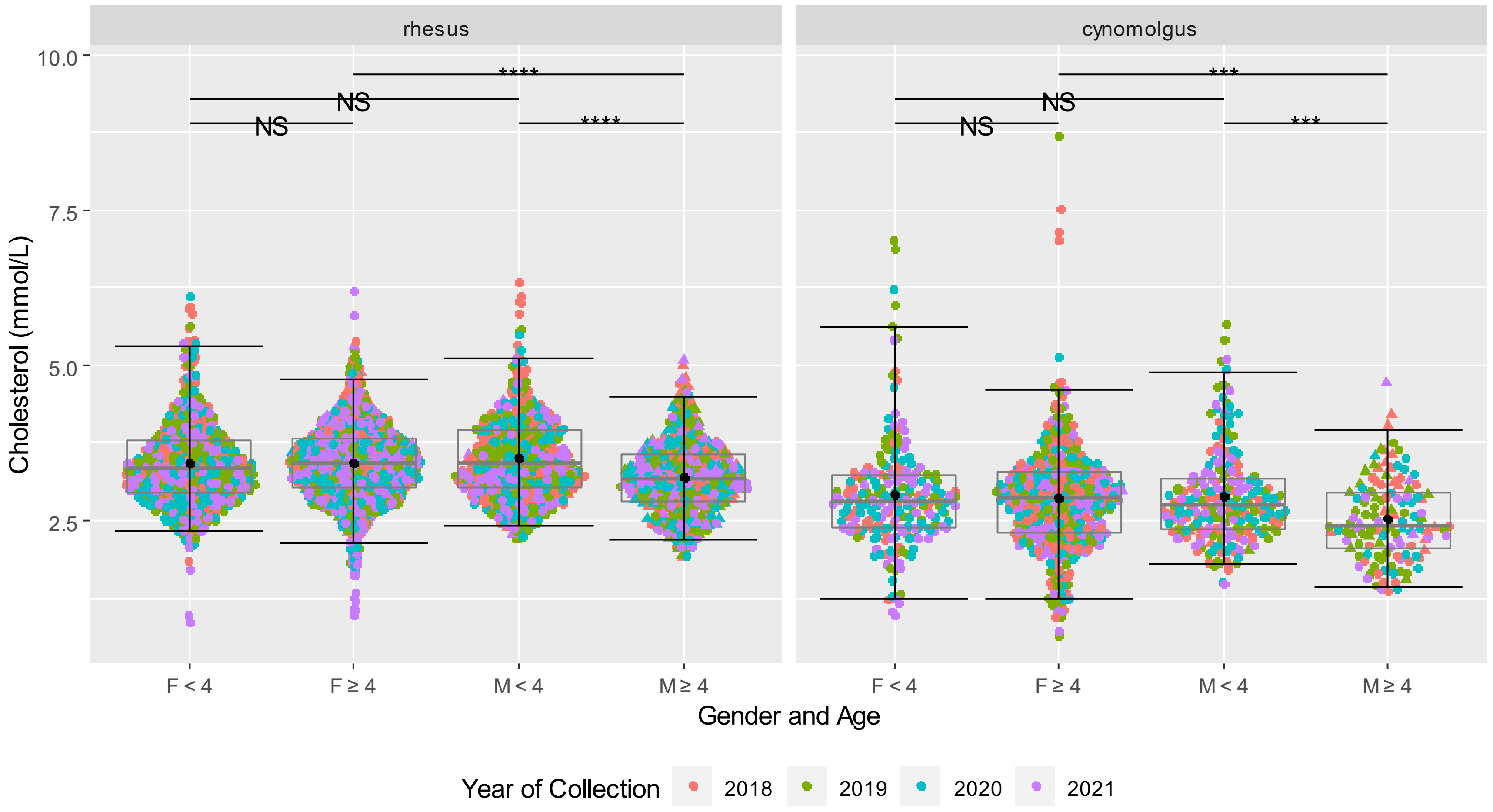

Figure S4 9. Cholesterol levels according to gender and age in rhesus and cynomolgus macaques. Boxes indicate median and quartile ranges, black point indicates arithmetic mean and error bars indicate the normal range. Round symbols indicate animals with outdoor access and triangles indicate indoor housed animals

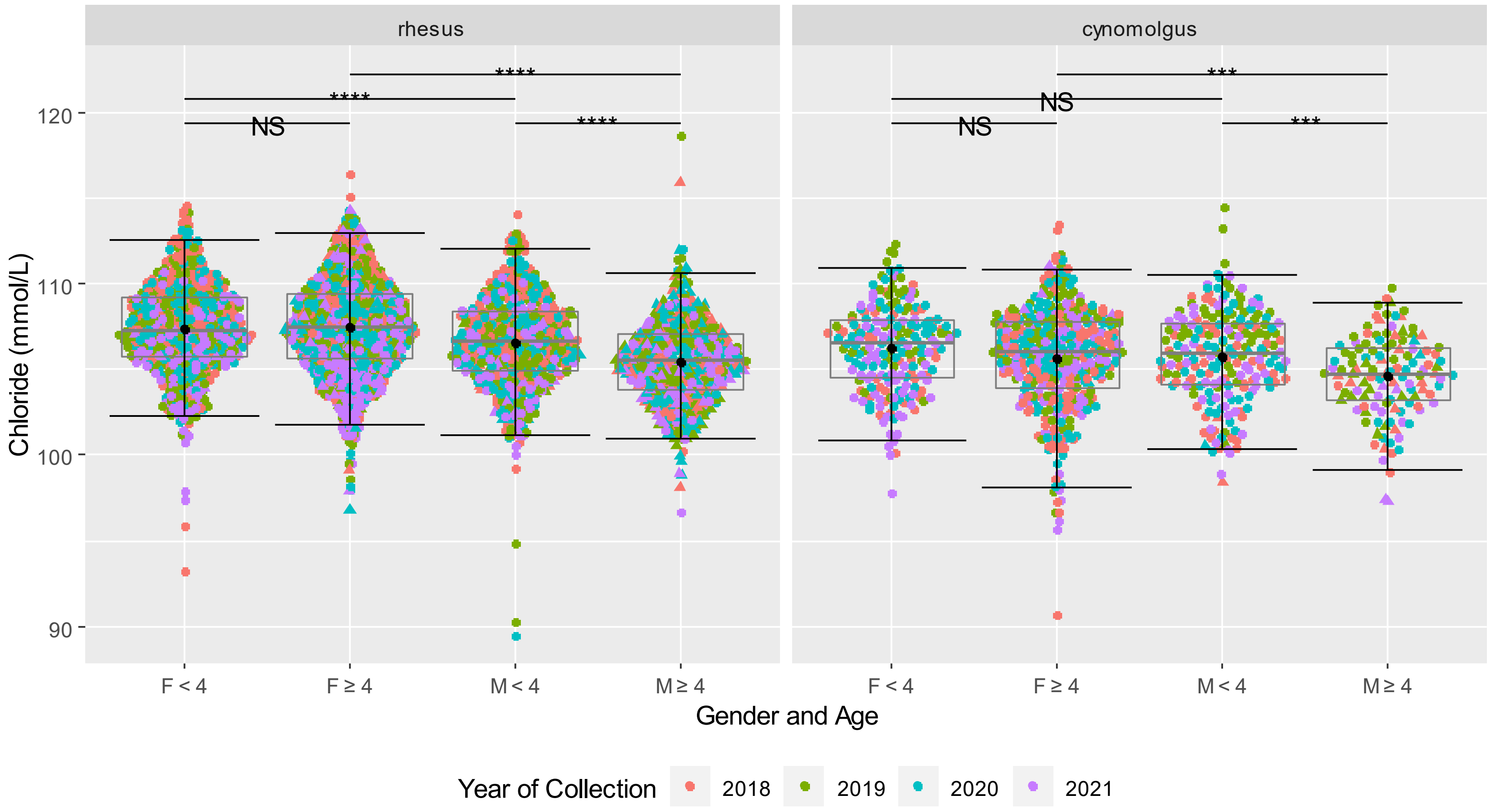

Figure S4 10. Chloride levels according to gender and age in rhesus and cynomolgus macaques. Boxes indicate median and quartile ranges, black point indicates arithmetic mean and error bars indicate the normal range. Round symbols indicate animals with outdoor access and triangles indicate indoor housed animals

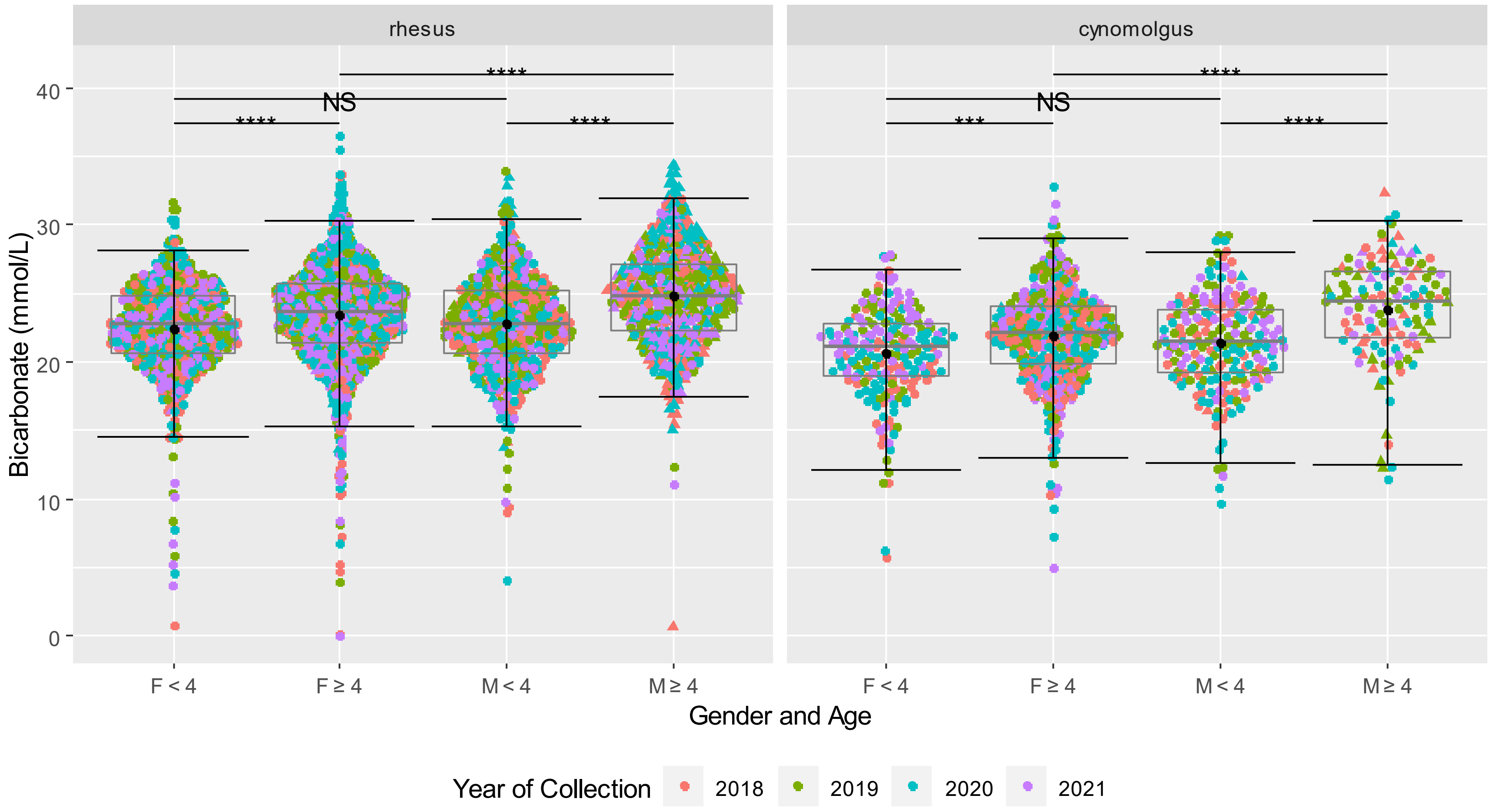

Figure S4 11. Bicarbonate levels according to gender and age in rhesus and cynomolgus macaques. Boxes indicate median and quartile ranges, black point indicates arithmetic mean and error bars indicate the normal range. Round symbols indicate animals with outdoor access and triangles indicate indoor housed animals

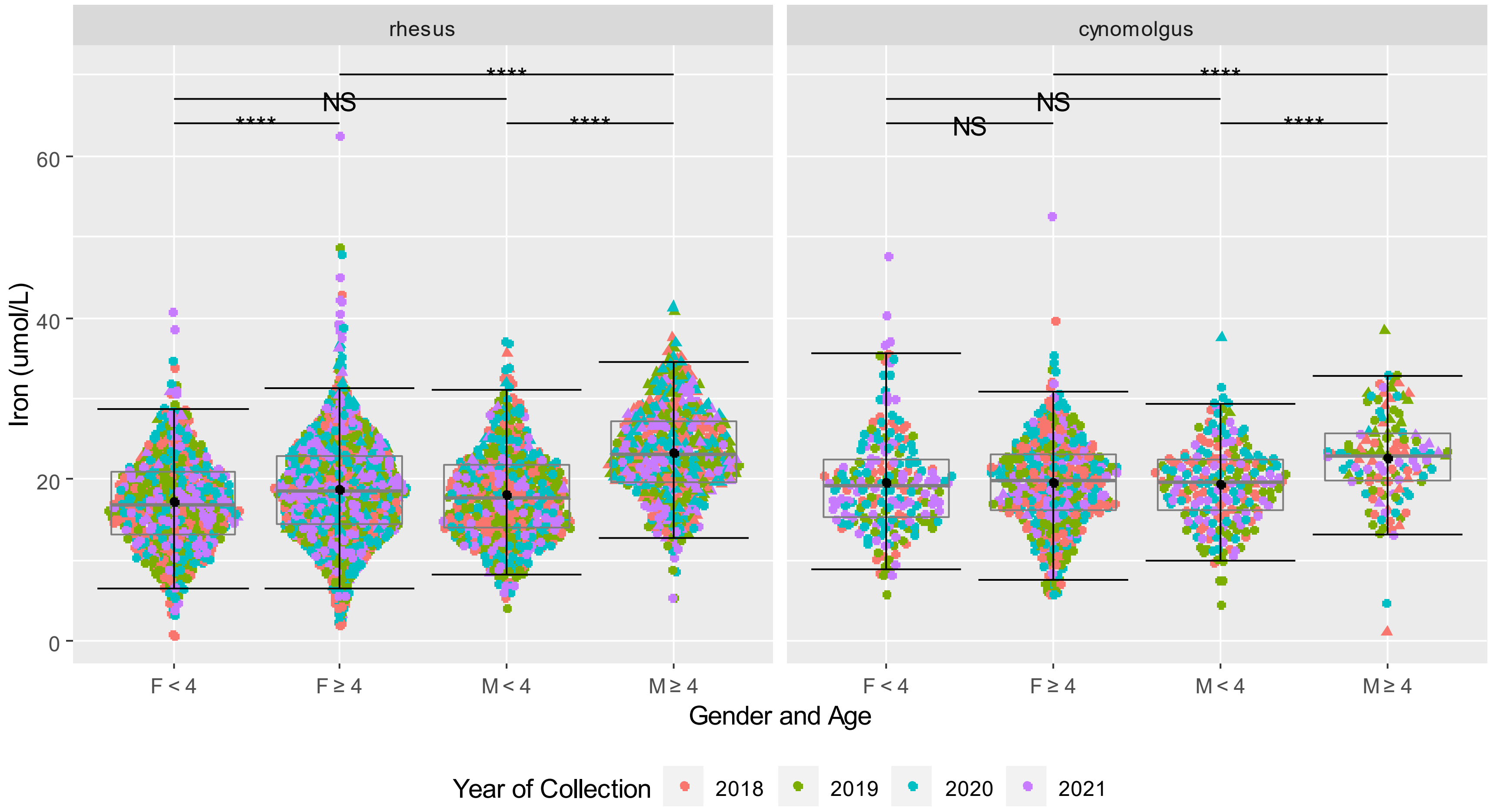

Figure S4 12. Iron levels according to gender and age in rhesus and cynomolgus macaques. Boxes indicate median and quartile ranges, black point indicates arithmetic mean and error bars indicate the normal range. Round symbols indicate animals with outdoor access and triangles indicate indoor housed animals

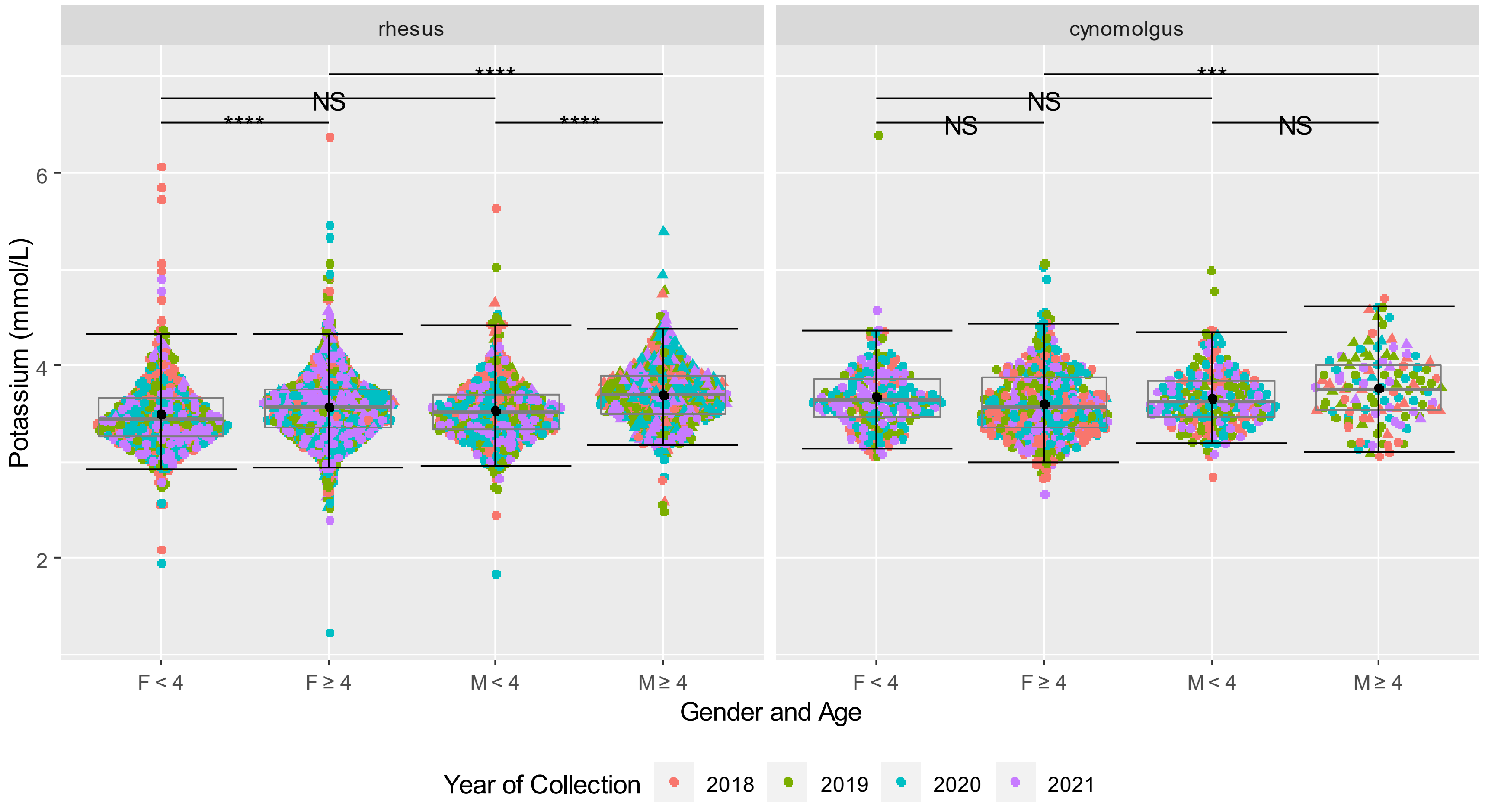

Figure S4 13. Potassium levels according to gender and age in rhesus and cynomolgus macaques. Boxes indicate median and quartile ranges, black point indicates arithmetic mean and error bars indicate the normal range. Round symbols indicate animals with outdoor access and triangles indicate indoor housed animals

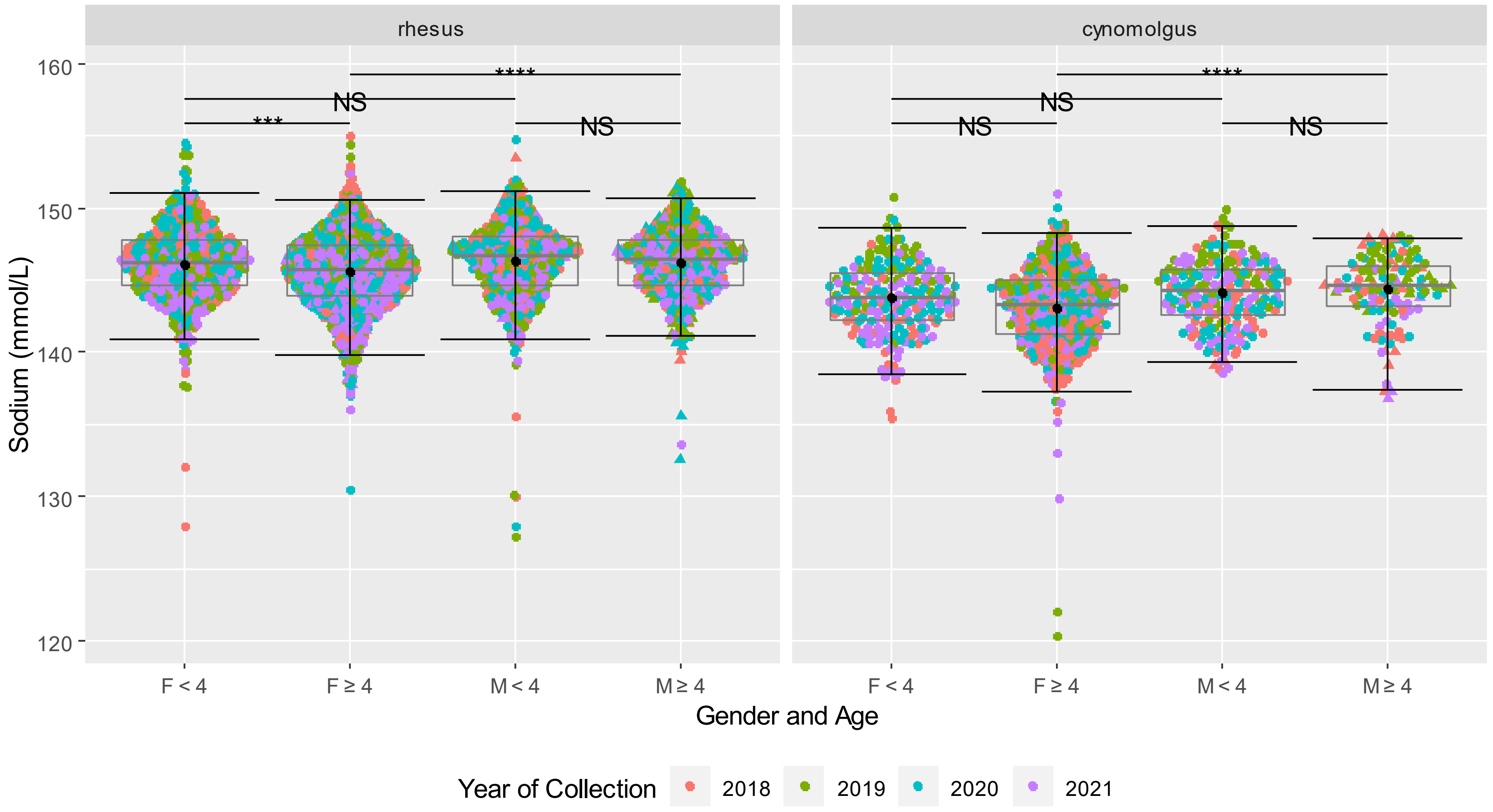

Figure S4 14. Sodium levels according to gender and age in rhesus and cynomolgus macaques. Boxes indicate median and quartile ranges, black point indicates arithmetic mean and error bars indicate the normal range. Round symbols indicate animals with outdoor access and triangles indicate indoor housed animals

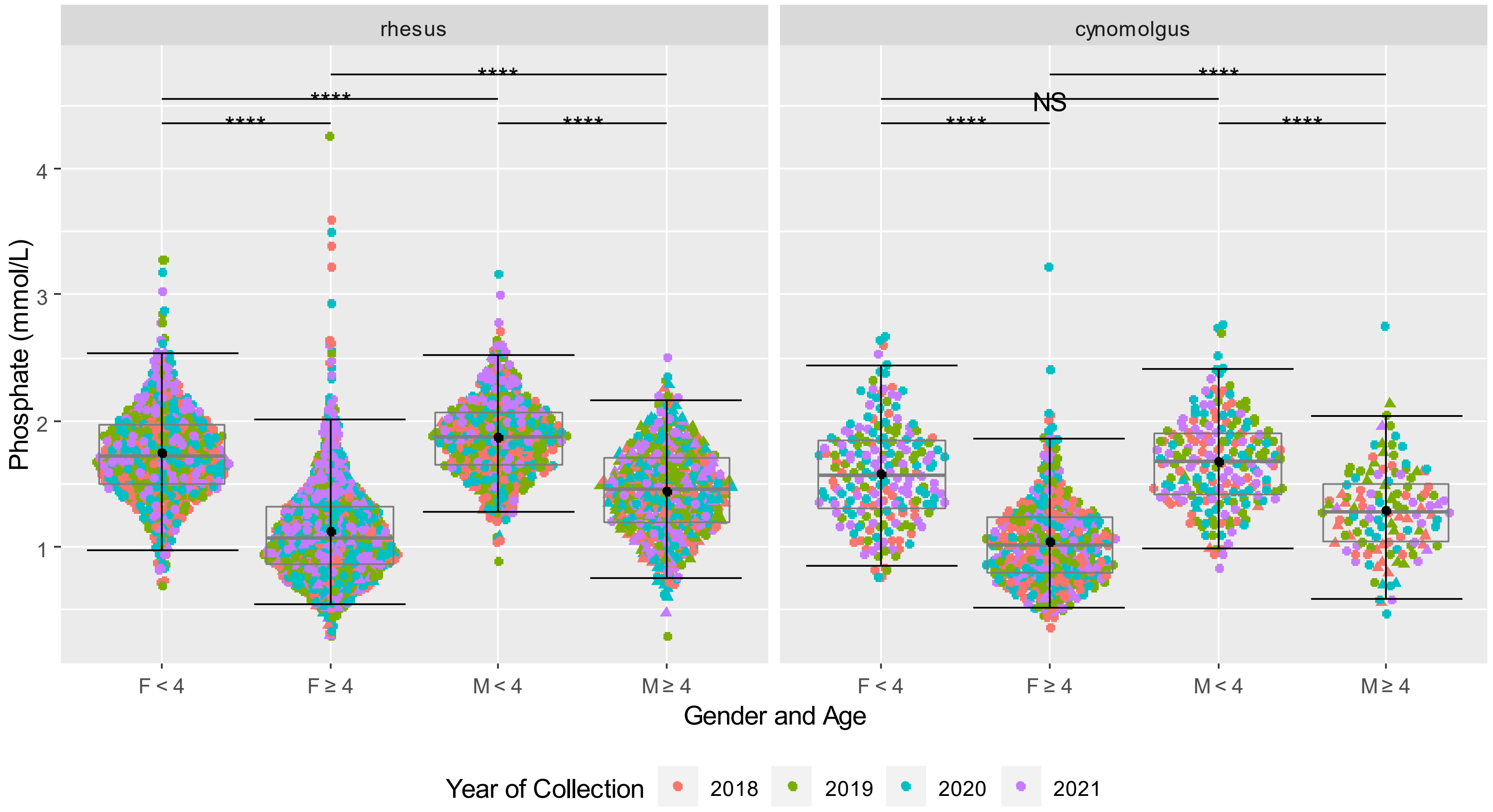

Figure S4 15. Phosphate levels according to gender and age in rhesus and cynomolgus macaques. Boxes indicate median and quartile ranges, black point indicates arithmetic mean and error bars indicate the normal range. Round symbols indicate animals with outdoor access and triangles indicate indoor housed animals

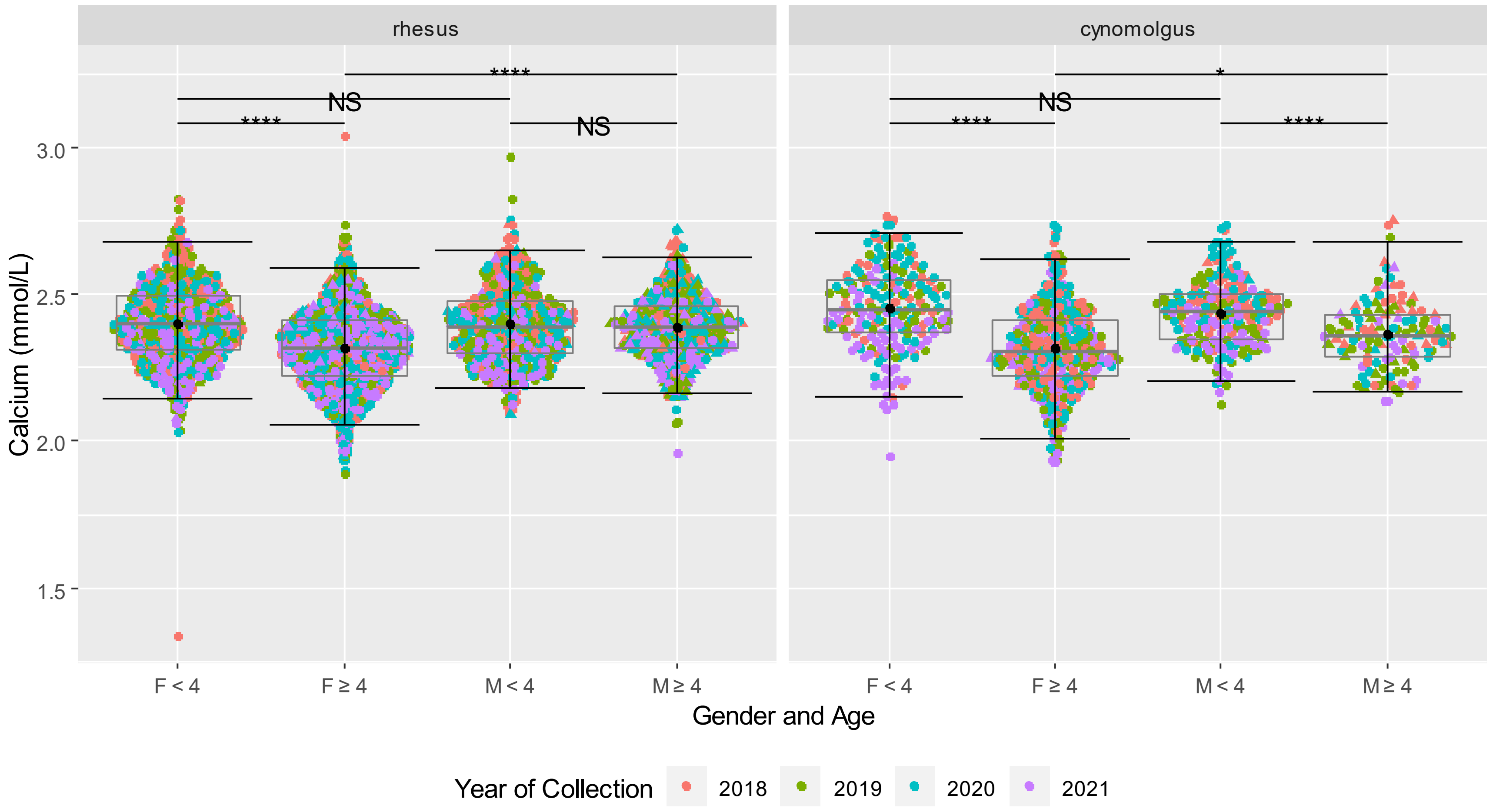

Figure S4 16. Calcium levels according to gender and age in rhesus and cynomolgus macaques. Boxes indicate median and quartile ranges, black point indicates arithmetic mean and error bars indicate the normal range. Round symbols indicate animals with outdoor access and triangles indicate indoor housed animals

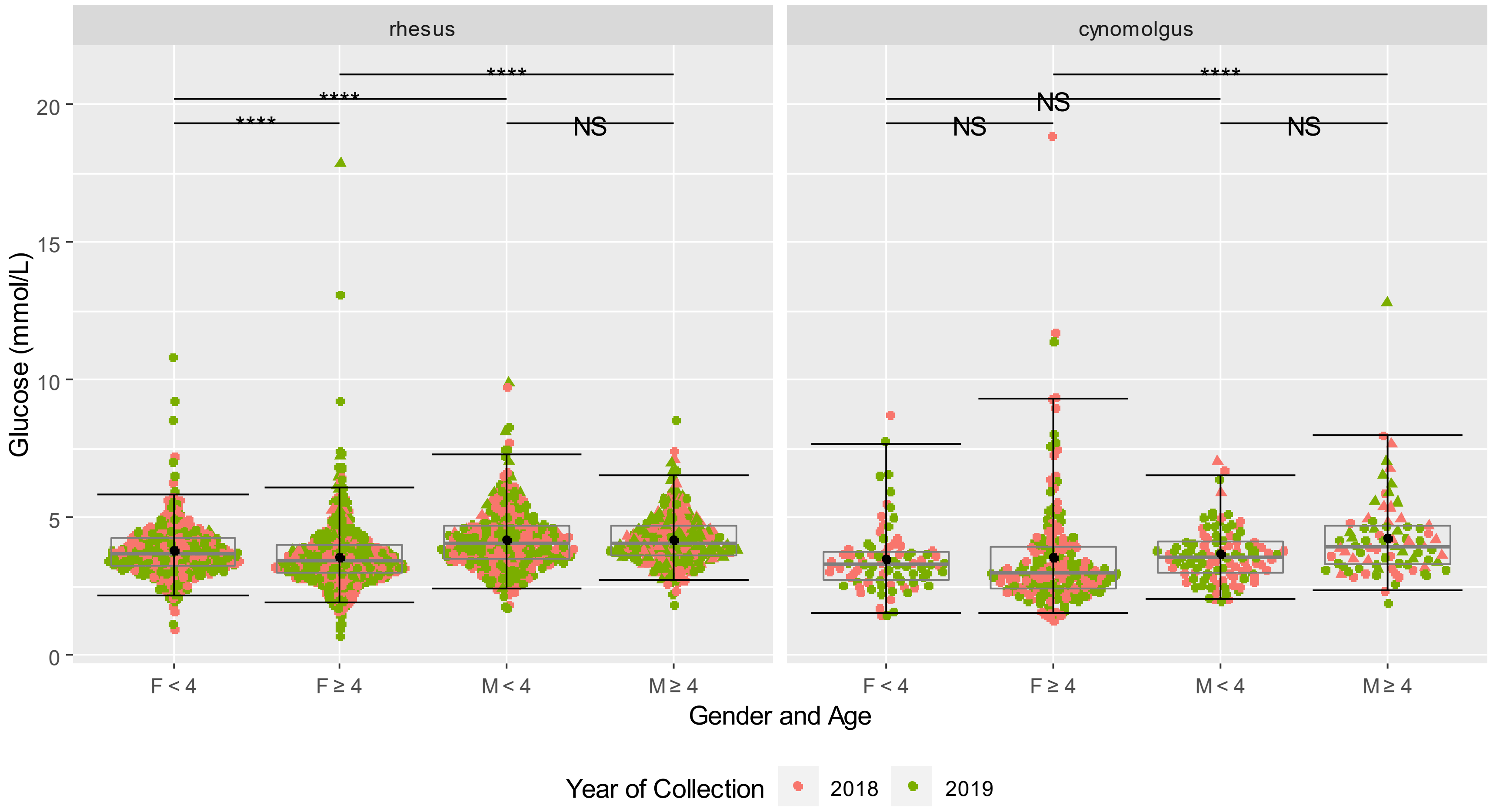

Figure S4 17A. Glucose levels in 2018 and 2019 according to gender and age in rhesus and cynomolgus macaques. Boxes indicate median and quartile ranges, black point indicates arithmetic mean and error bars indicate the normal range. Round symbols indicate animals with outdoor access and triangles indicate indoor housed animals

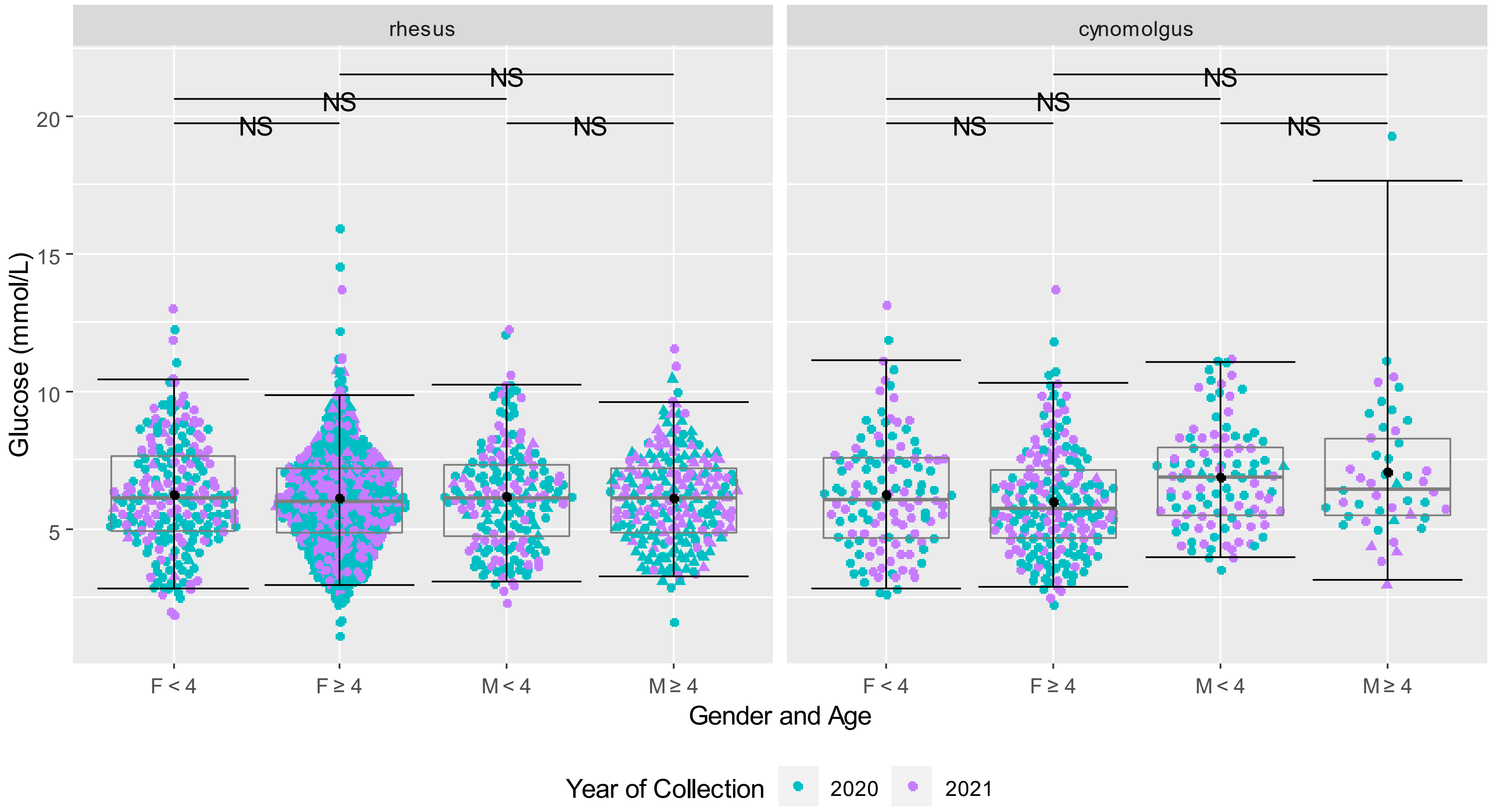

Figure S4

17B. Glucose levels in 2020 and 2021 according to gender and age in rhesus and cynomolgus macaques. Boxes indicate median and quartile ranges, black point indicates arithmetic mean and error bars indicate the normal range. Round symbols indicate animals with outdoor access and triangles indicate indoor housed animals

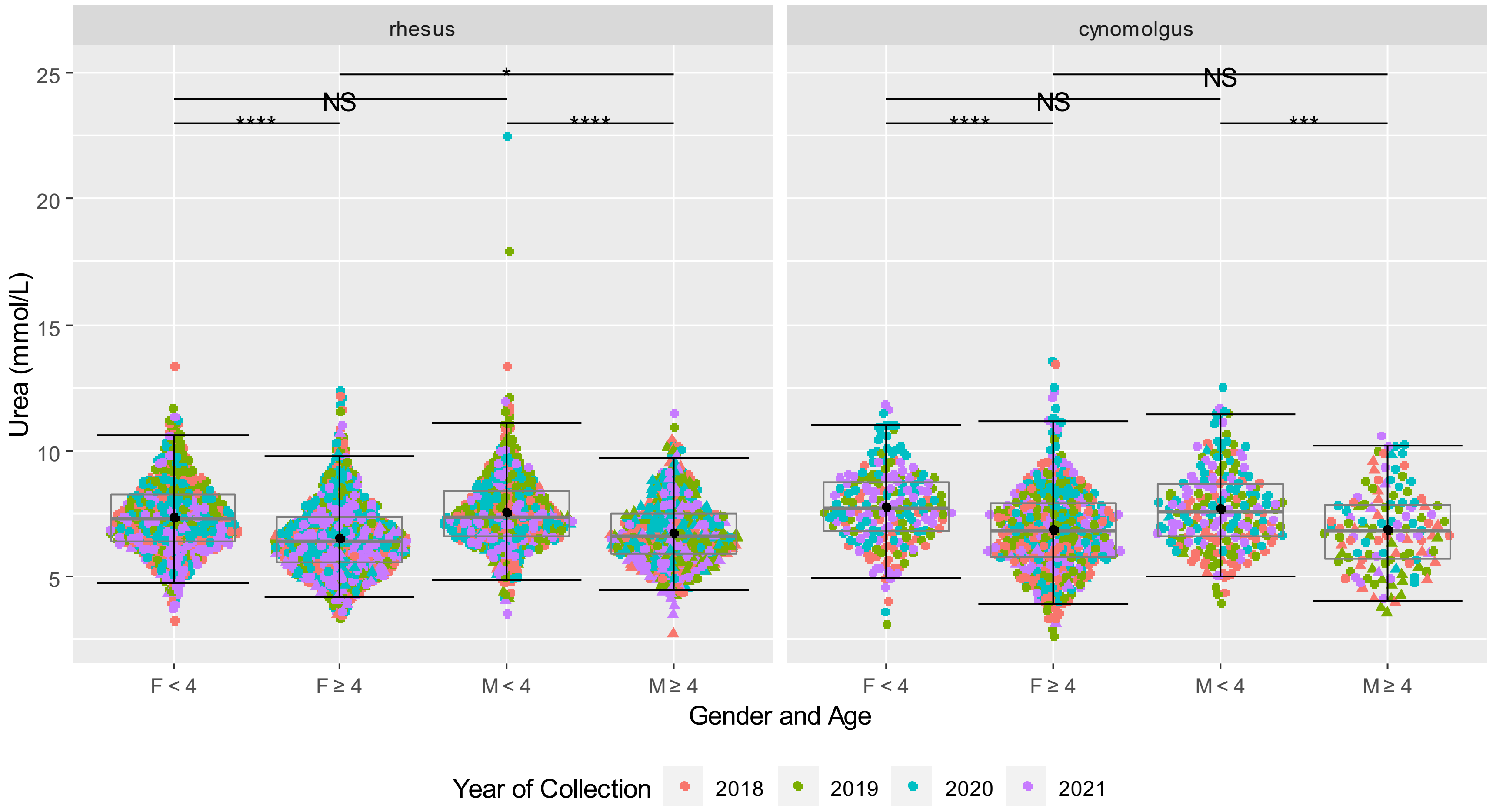

Figure S4 18. Urea levels according to gender and age in rhesus and cynomolgus macaques. Boxes indicate median and quartile ranges, black point indicates arithmetic mean and error bars indicate the normal range. Round symbols indicate animals with outdoor access and triangles indicate indoor housed animals

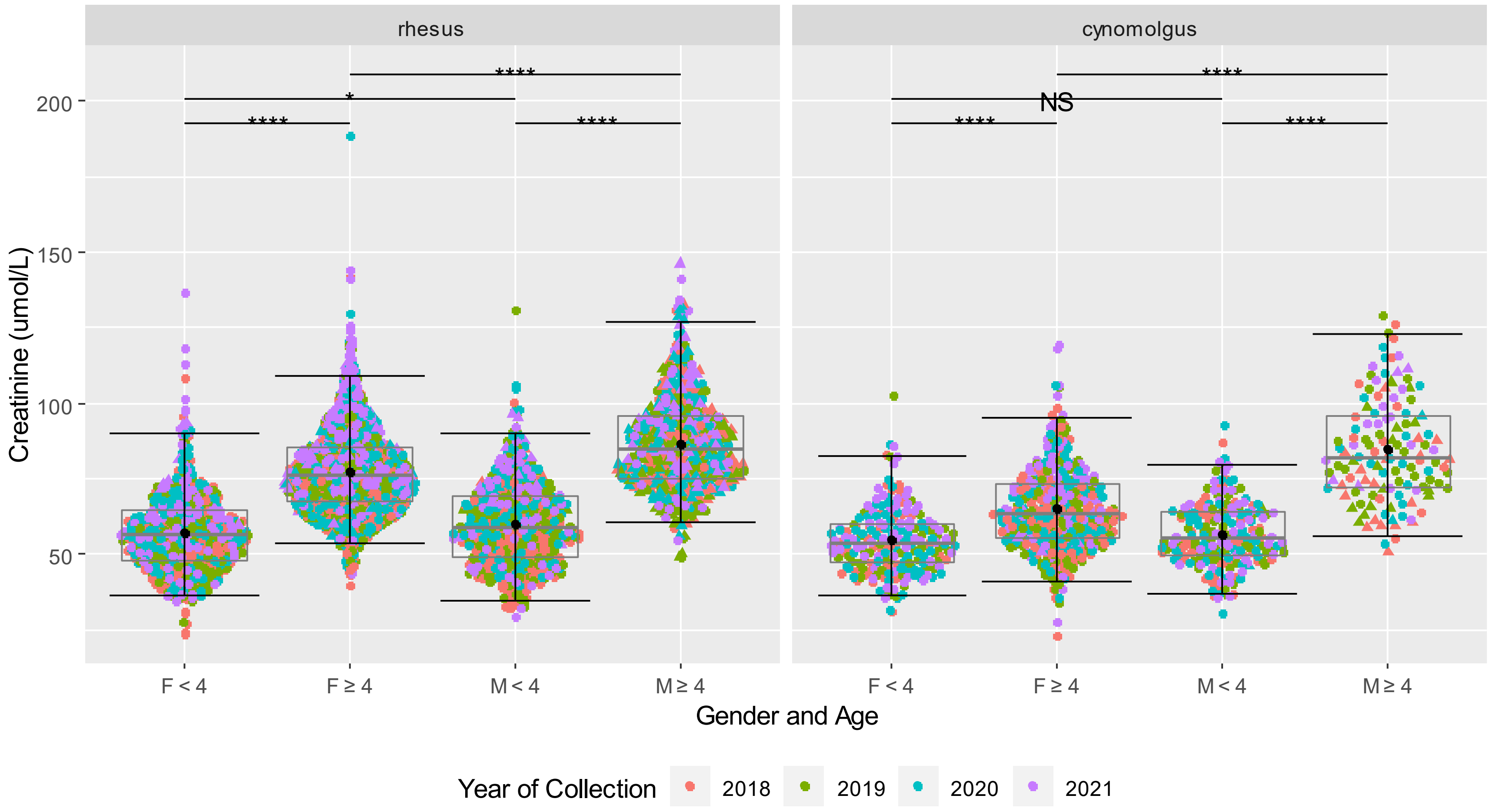

Figure S4 19. Creatinine levels according to gender and age in rhesus and cynomolgus macaques. Boxes indicate median and quartile ranges, black point indicates arithmetic mean and error bars indicate the normal range. Round symbols indicate animals with outdoor access and triangles indicate indoor housed animals

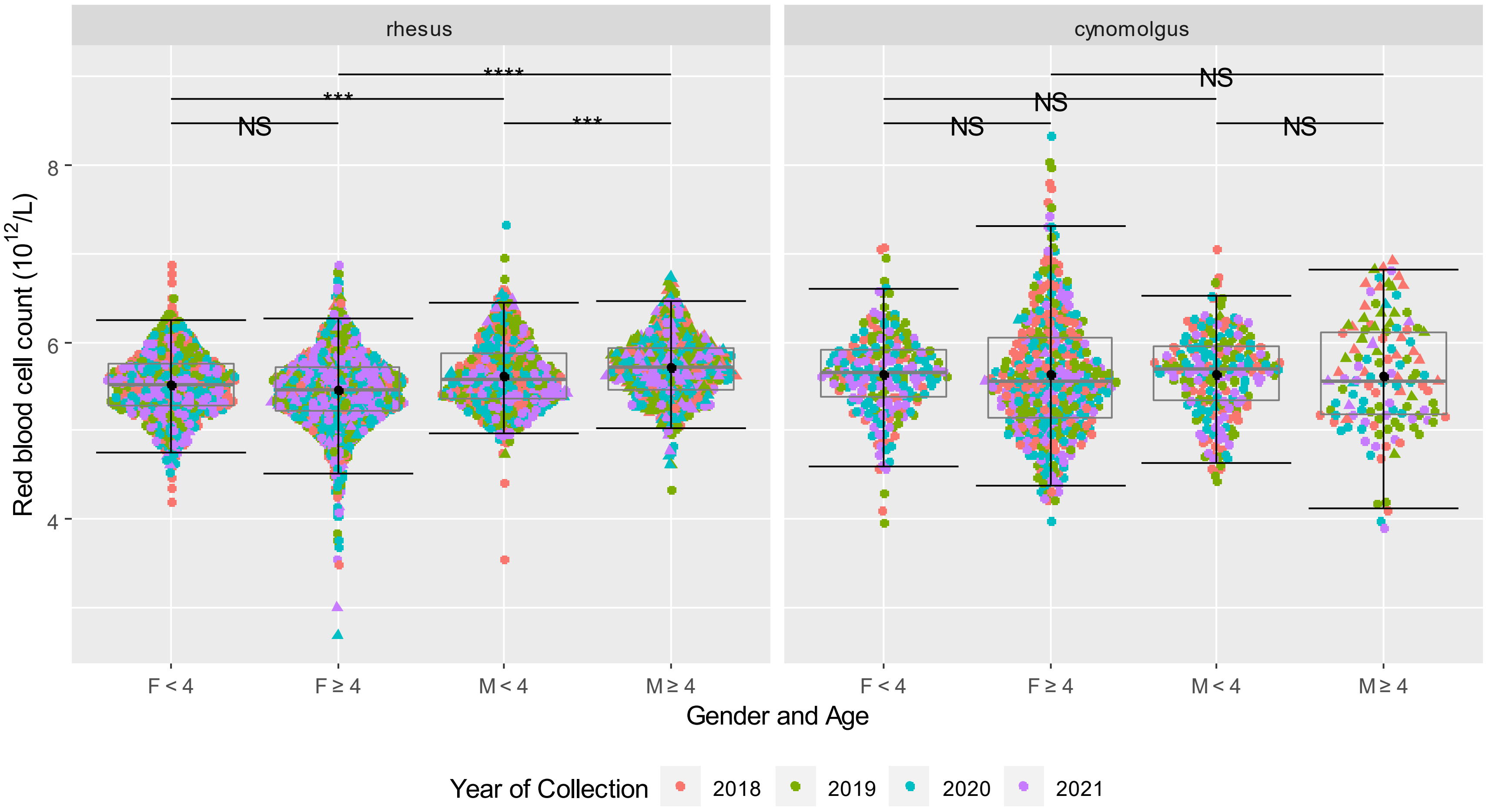

Figure S4 20. Red Blood Cell count according to gender and age in rhesus and cynomolgus macaques. Boxes indicate median and quartile ranges, black point indicates arithmetic mean and error bars indicate the normal range. Round symbols indicate animals with outdoor access and triangles indicate indoor housed animals

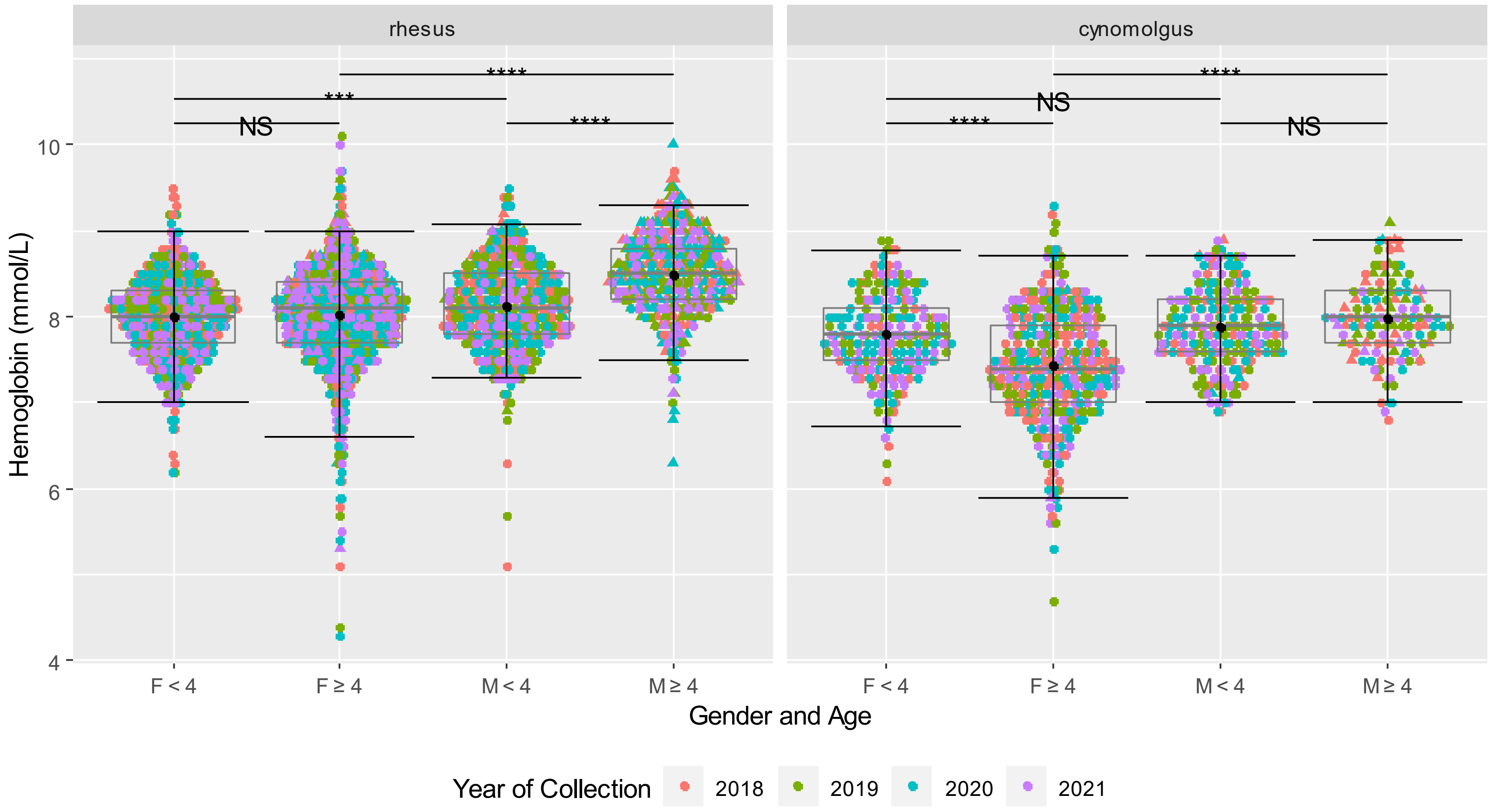

Figure S4 21. Hemoglobin levels according to gender and age in rhesus and cynomolgus macaques. Boxes indicate median and quartile ranges, black point indicates arithmetic mean and error bars indicate the normal range. Round symbols indicate animals with outdoor access and triangles indicate indoor housed animals

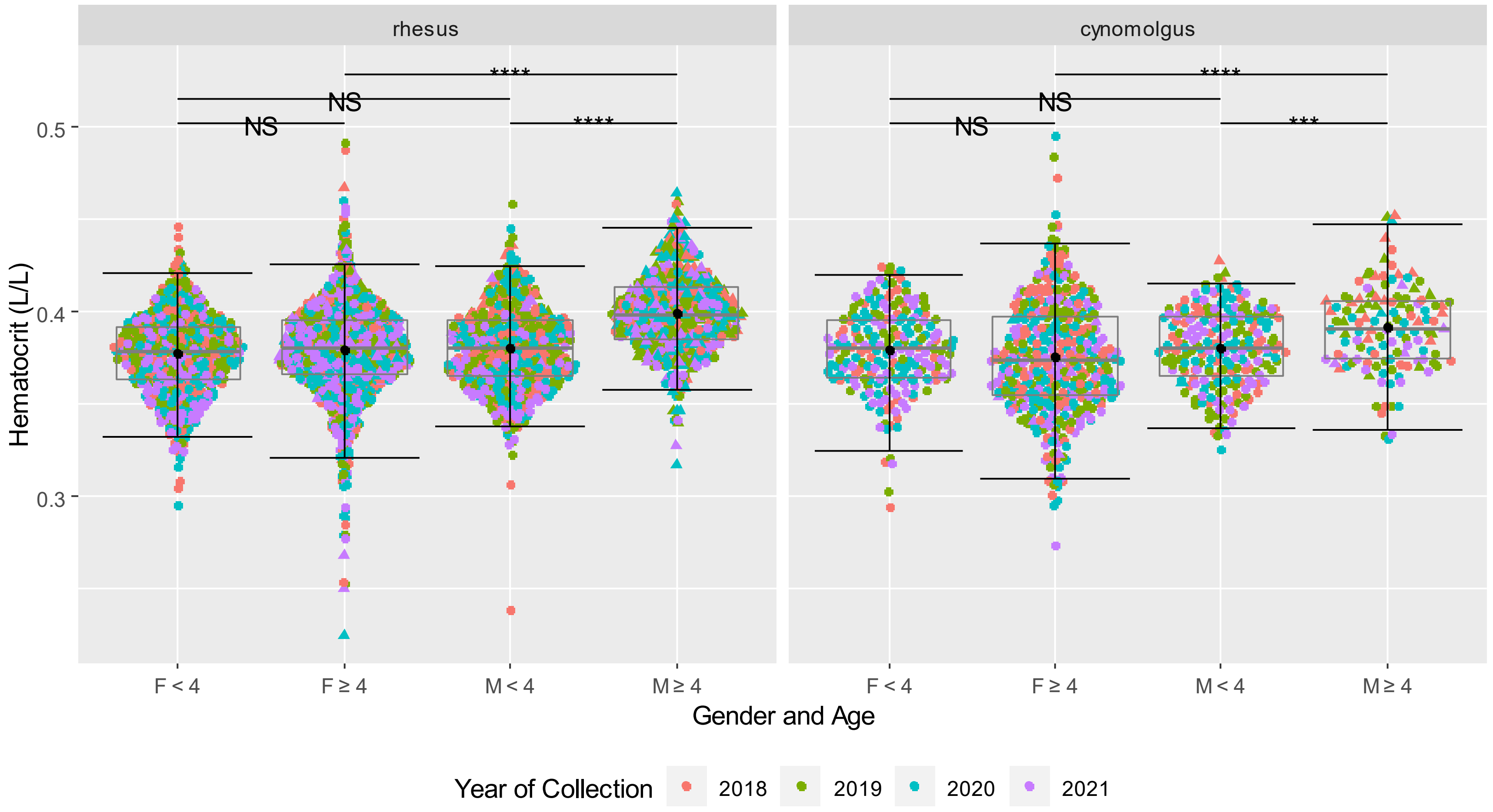

Figure S4 22. Hematocrit levels according to gender and age in rhesus and cynomolgus macaques. Boxes indicate median and quartile ranges, black point indicates arithmetic mean and error bars indicate the normal range. Round symbols indicate animals with outdoor access and triangles indicate indoor housed animals

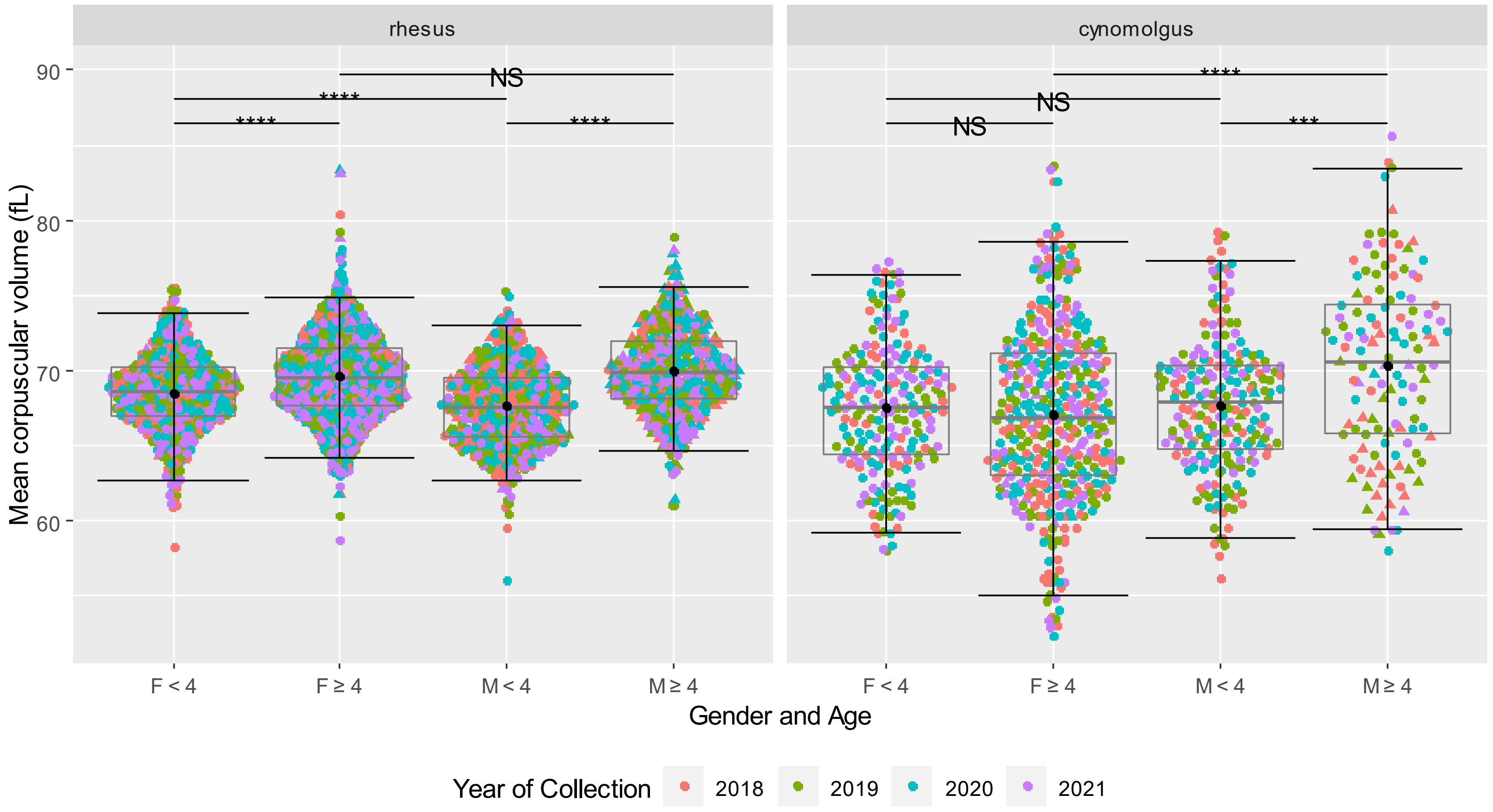

Figure S4 23. Mean Corpuscular Volume according to gender and age in rhesus and cynomolgus macaques. Boxes indicate median and quartile ranges, black point indicates arithmetic mean and error bars indicate the normal range. Round symbols indicate animals with outdoor access and triangles indicate indoor housed animals

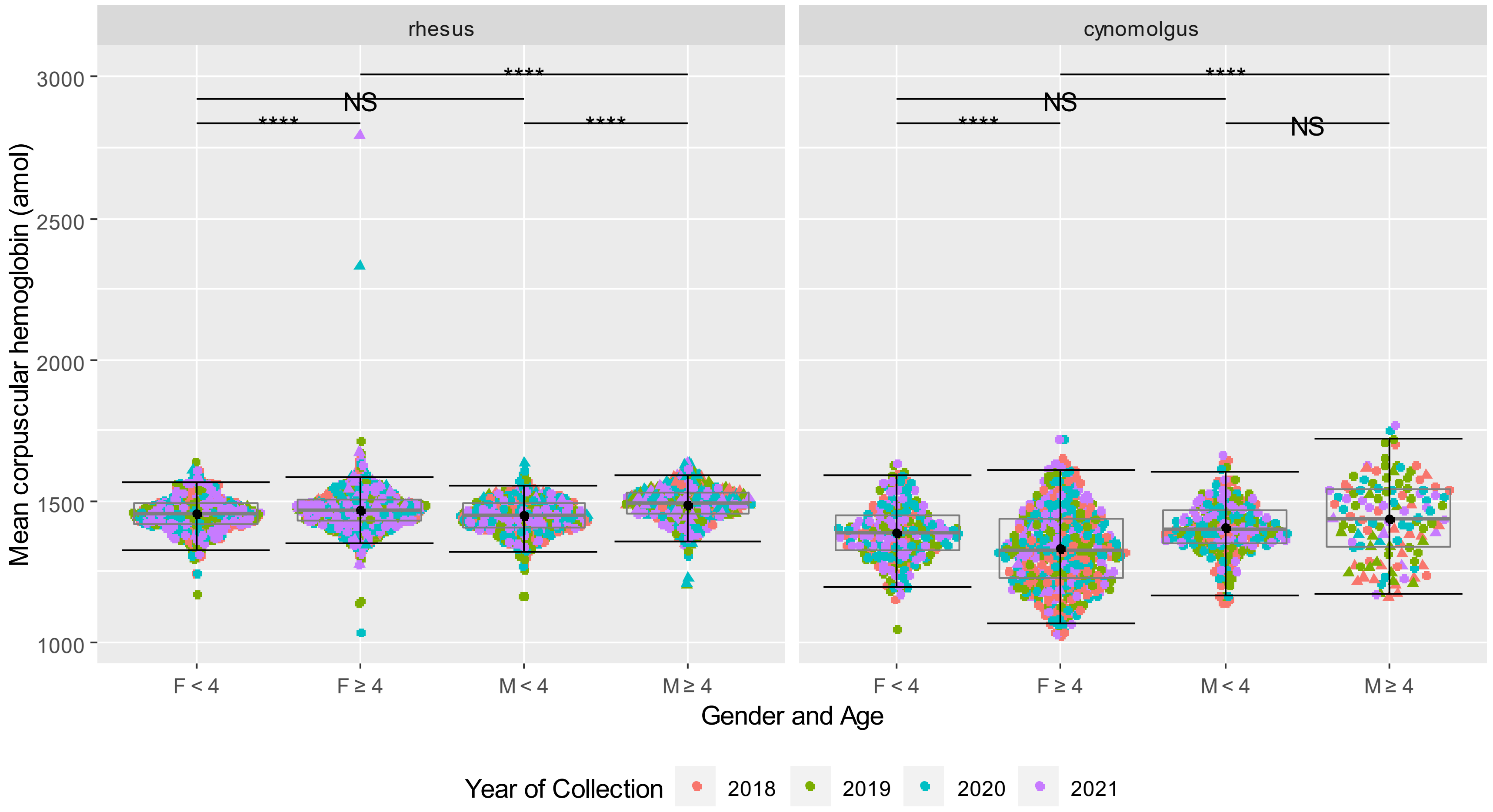

Figure S4 24. Mean Corpuscular Hemoglobin according to gender and age in rhesus and cynomolgus macaques. Boxes indicate median and quartile ranges, black point indicates arithmetic mean and error bars indicate the normal range. Round symbols indicate animals with outdoor access and triangles indicate indoor housed animals

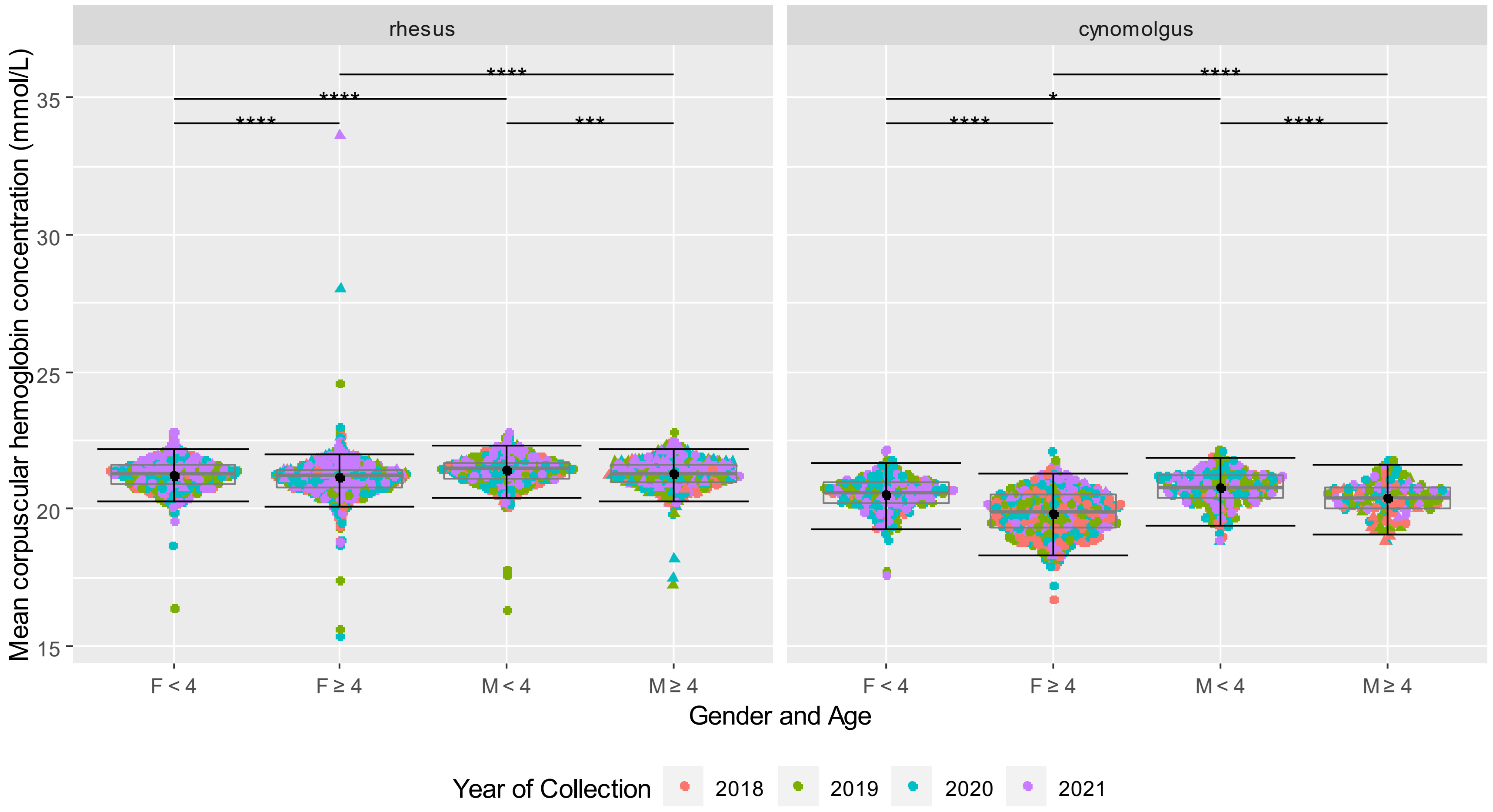

Figure S4 25. Mean Corpuscular Hemoglobin Concentration according to gender and age in rhesus and cynomolgus macaques. Boxes indicate median and quartile ranges, black point indicates arithmetic mean and error bars indicate the normal range. Round symbols indicate animals with outdoor access and triangles indicate indoor housed animals

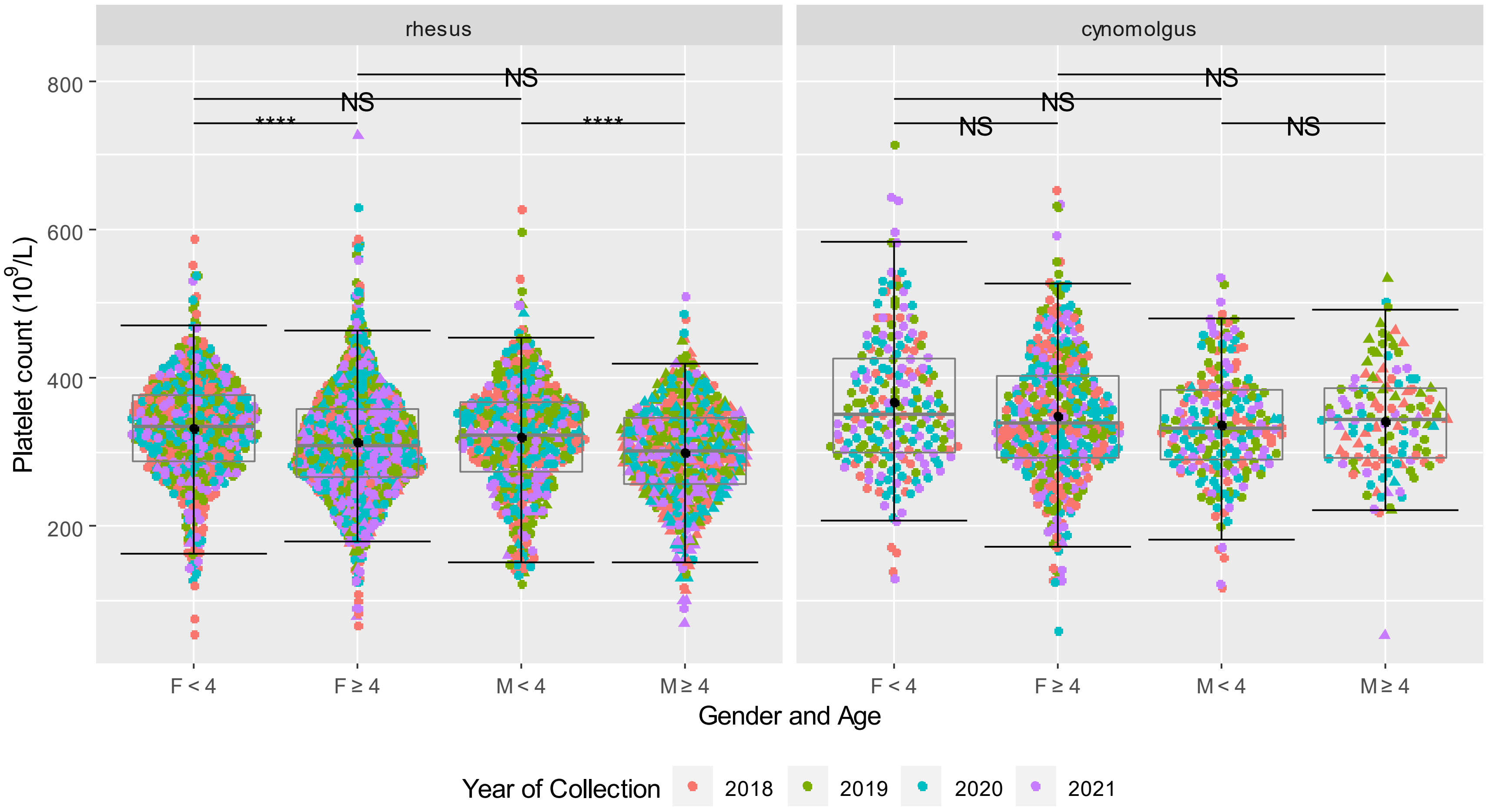

Figure S4 26. Platelet Count according to gender and age in rhesus and cynomolgus macaques. Boxes indicate median and quartile ranges, black point indicates arithmetic mean and error bars indicate the normal range. Round symbols indicate animals with outdoor access and triangles indicate indoor housed animals

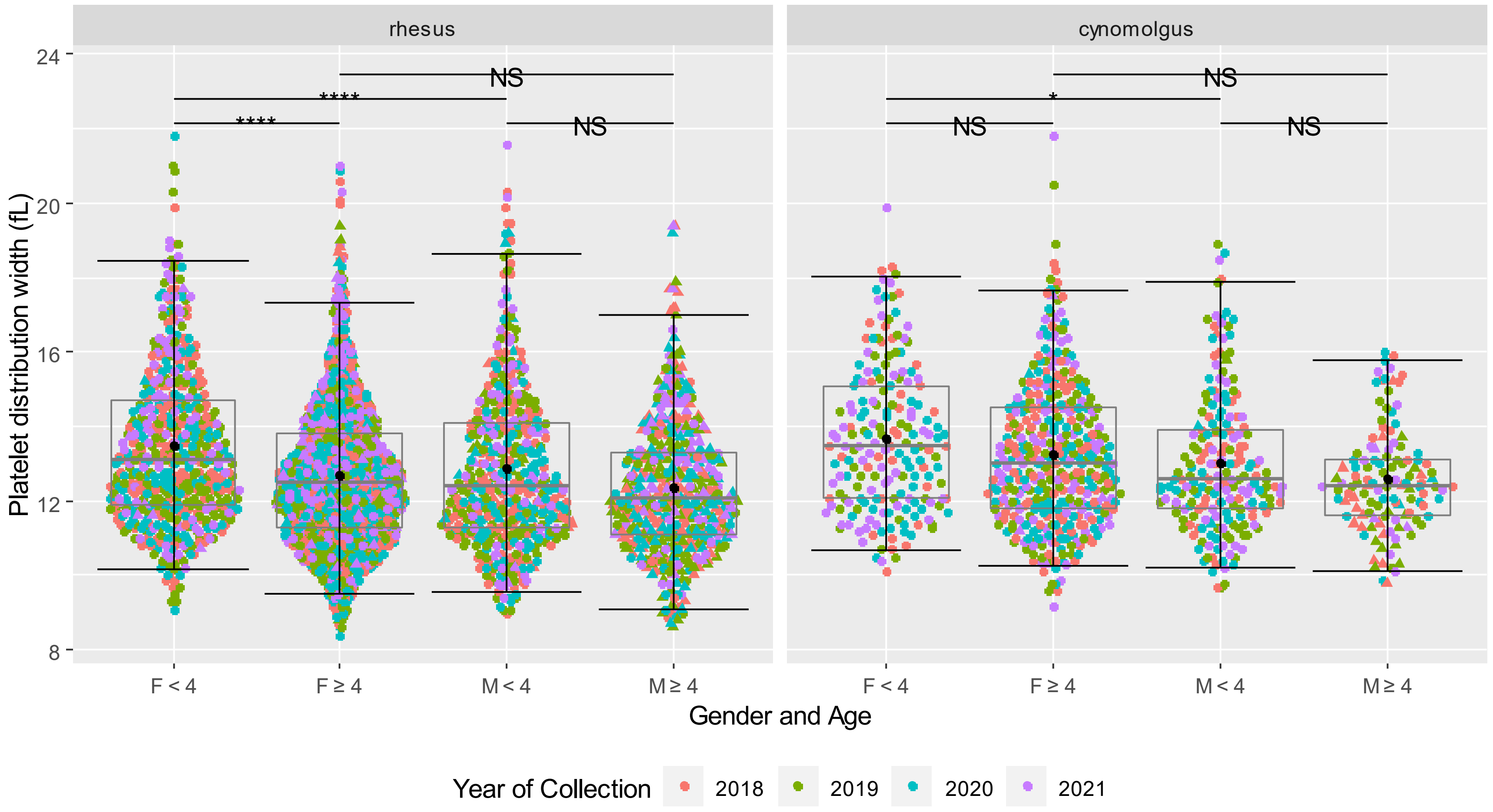

Figure S4 27. Platelet Distribution Width according to gender and age in rhesus and cynomolgus macaques. Boxes indicate median and quartile ranges, black point indicates arithmetic mean and error bars indicate the normal range. Round symbols indicate animals with outdoor access and triangles indicate indoor housed animals

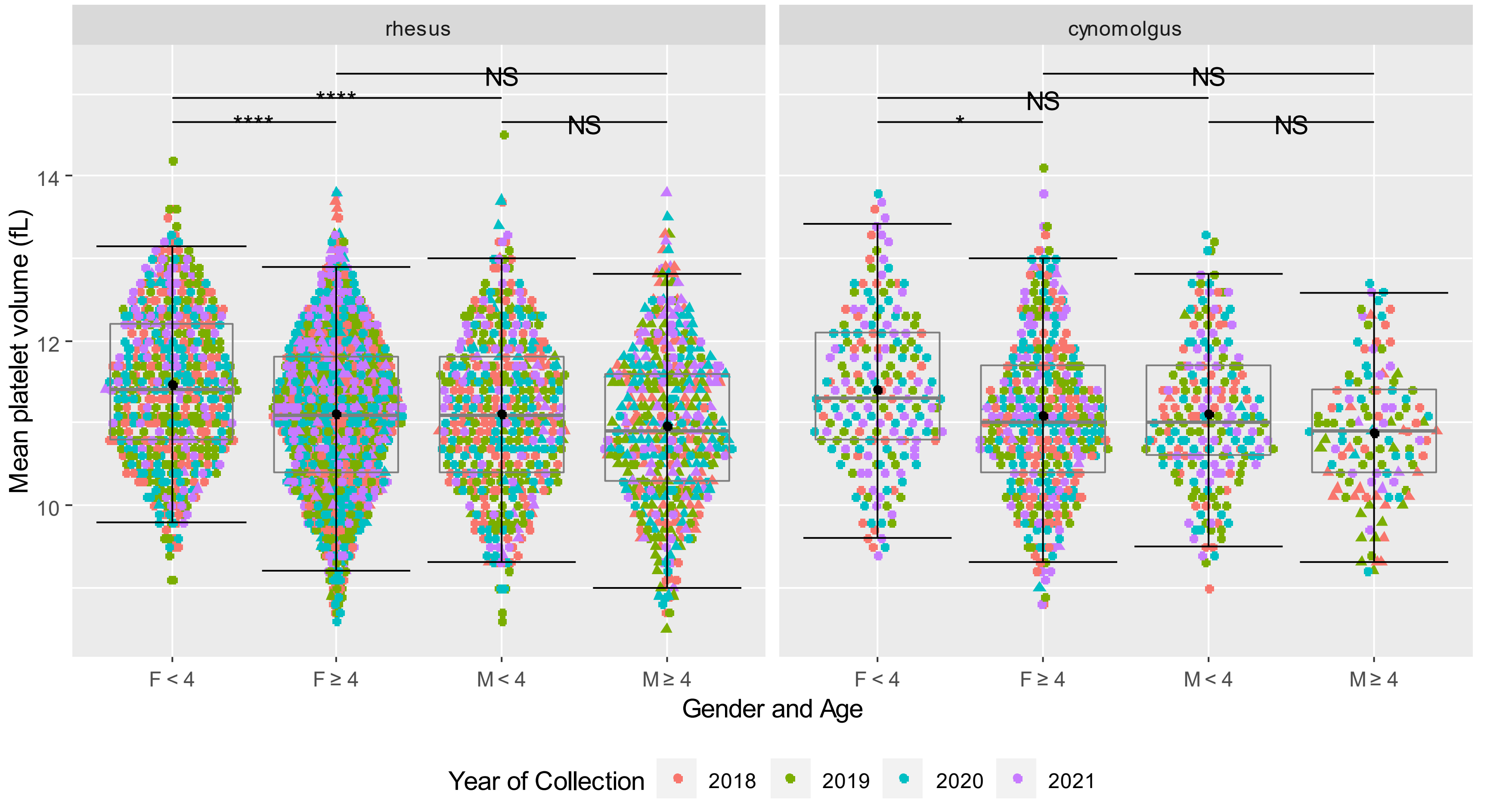

Figure S4 28. Mean Platelet Volume according to gender and age in rhesus and cynomolgus macaques. Boxes indicate median and quartile ranges, black point indicates arithmetic mean and error bars indicate the normal range. Round symbols indicate animals with outdoor access and triangles indicate indoor housed animals

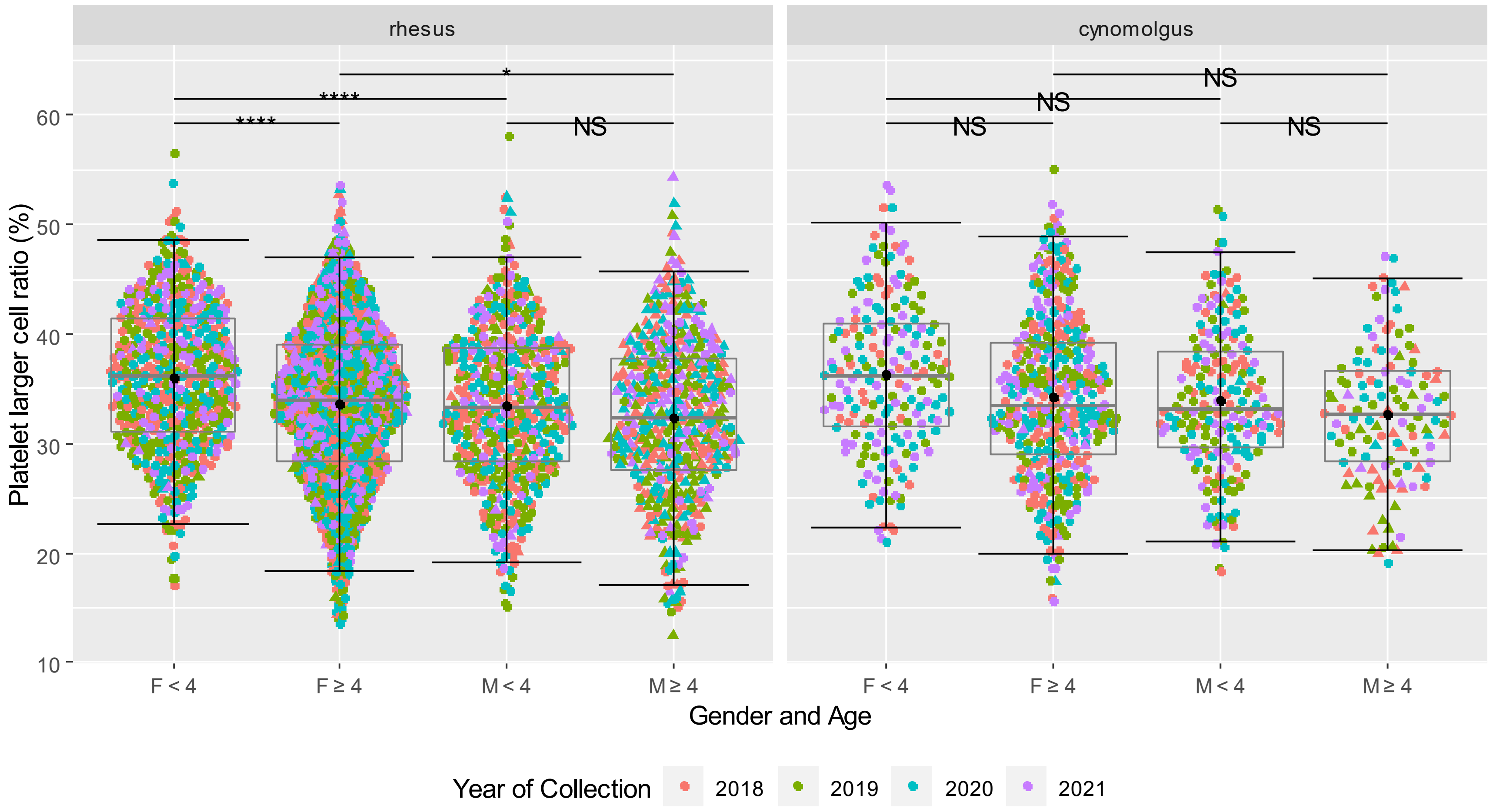

Figure S4 29. Platelet Larger Cell Ratio according to gender and age in rhesus and cynomolgus macaques. Boxes indicate median and quartile ranges, black point indicates arithmetic mean and error bars indicate the normal range. Round symbols indicate animals with outdoor access and triangles indicate indoor housed animals

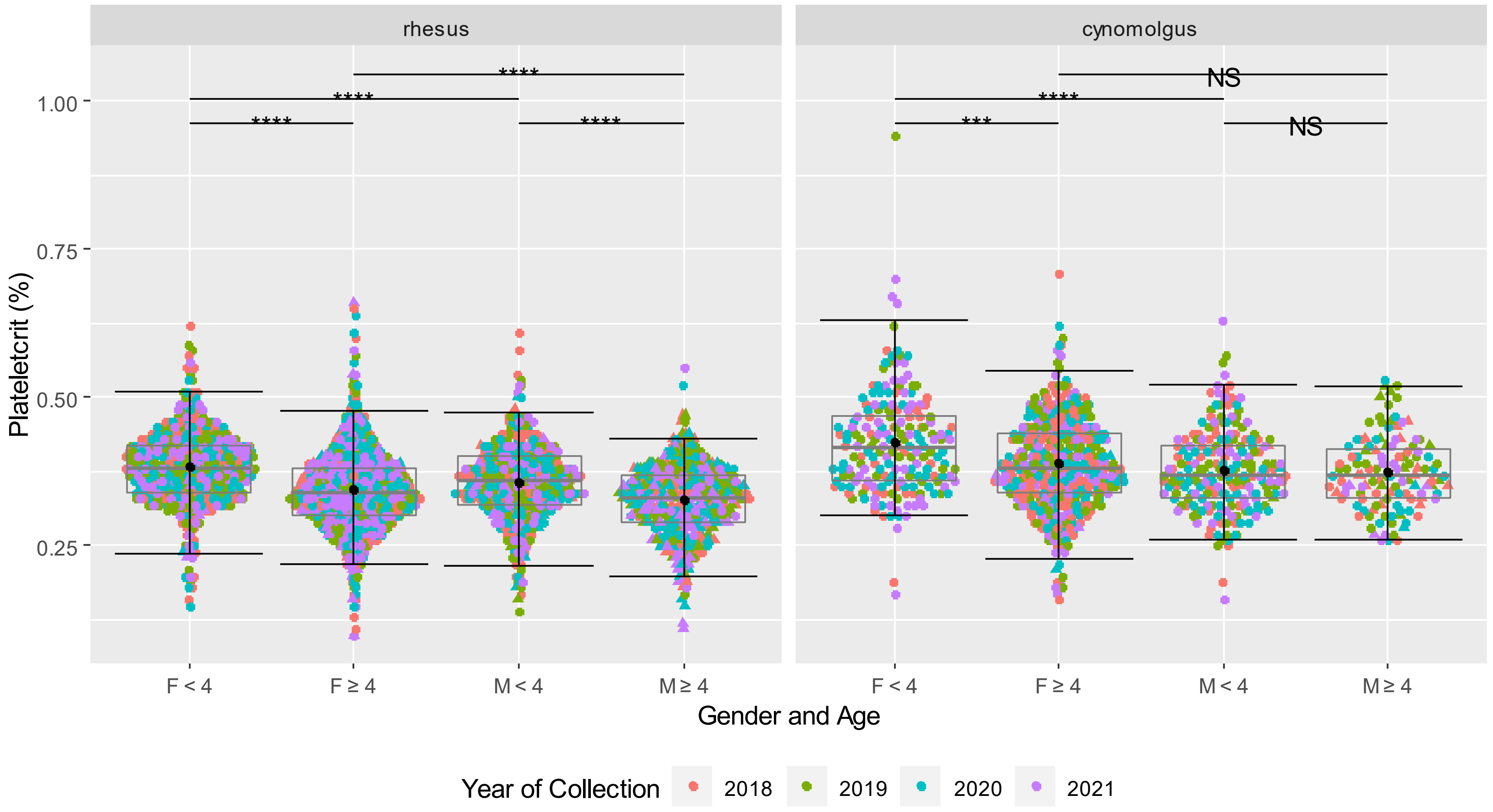

Figure S4 30. Plateletcrit according to gender and age in rhesus and cynomolgus macaques. Boxes indicate median and quartile ranges, black point indicates arithmetic mean and error bars indicate the normal range. Round symbols indicate animals with outdoor access and triangles indicate indoor housed animals

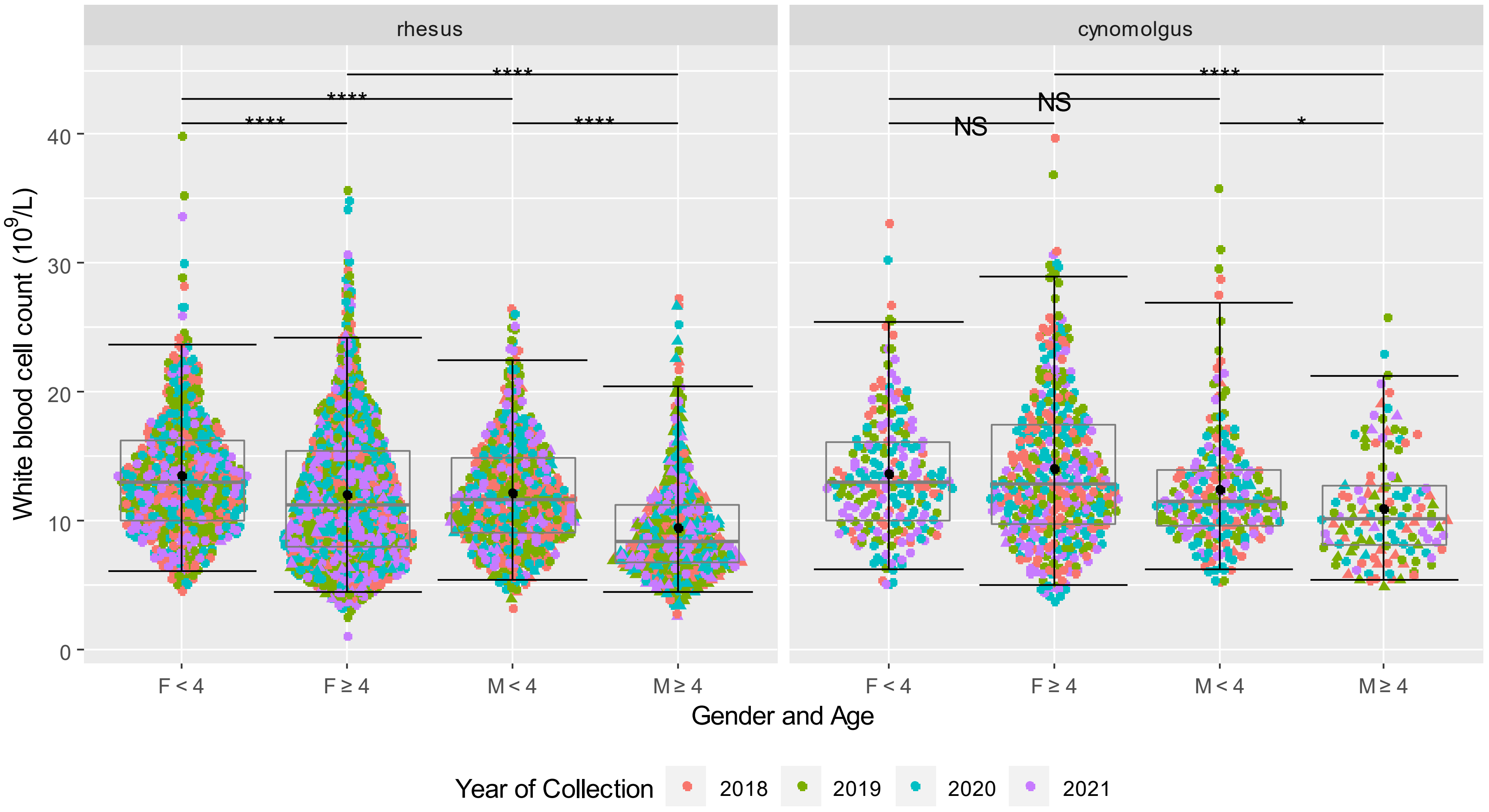

Figure S4 31. White Blood Cell Count according to gender and age in rhesus and cynomolgus macaques. Boxes indicate median and quartile ranges, black point indicates arithmetic mean and error bars indicate the normal range. Round symbols indicate animals with outdoor access and triangles indicate indoor housed animals

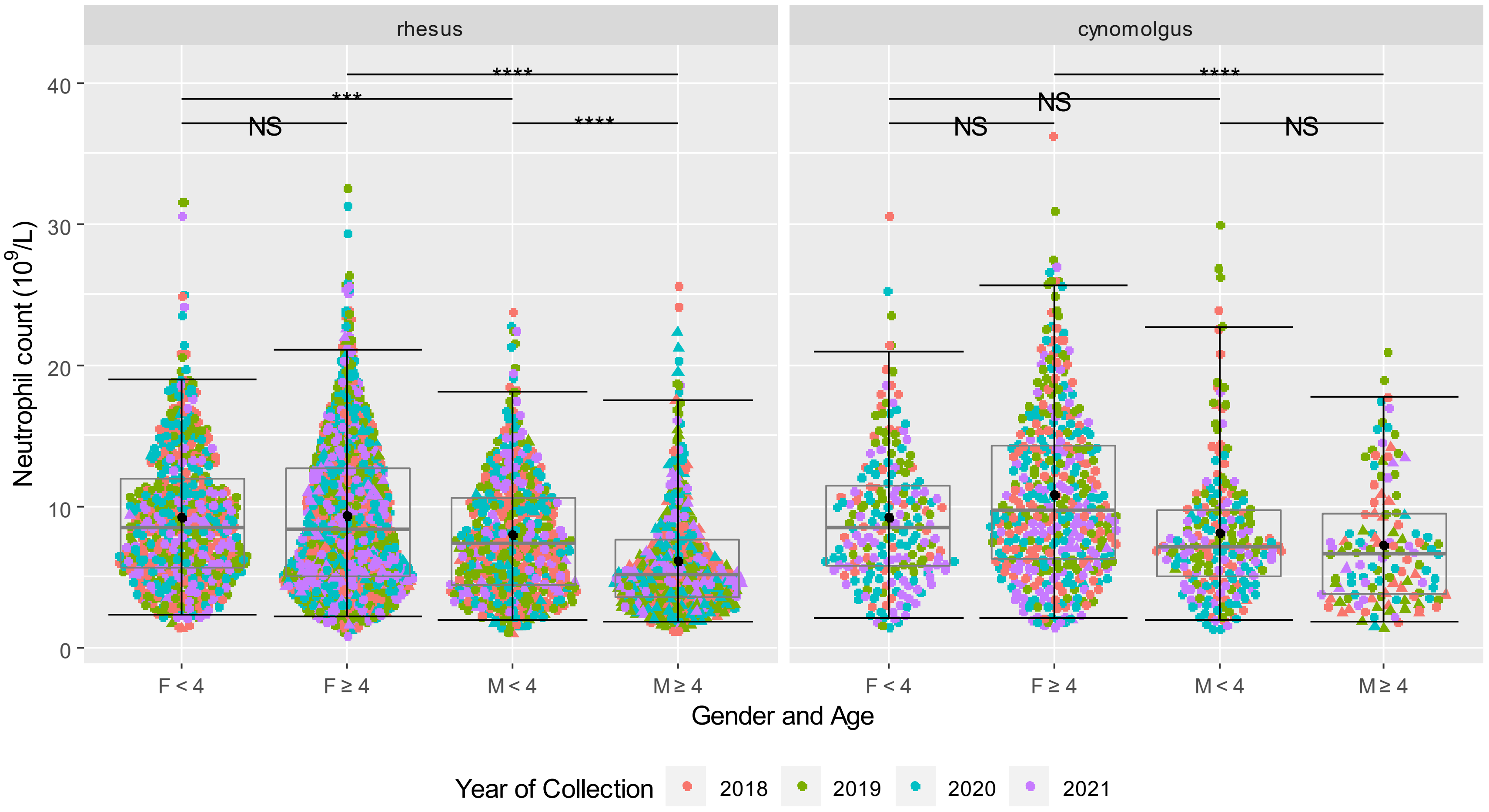

Figure S4 32. Neutrophil Count according to gender and age in rhesus and cynomolgus macaques. Boxes indicate median and quartile ranges, black point indicates arithmetic mean and error bars indicate the normal range. Round symbols indicate animals with outdoor access and triangles indicate indoor housed animals

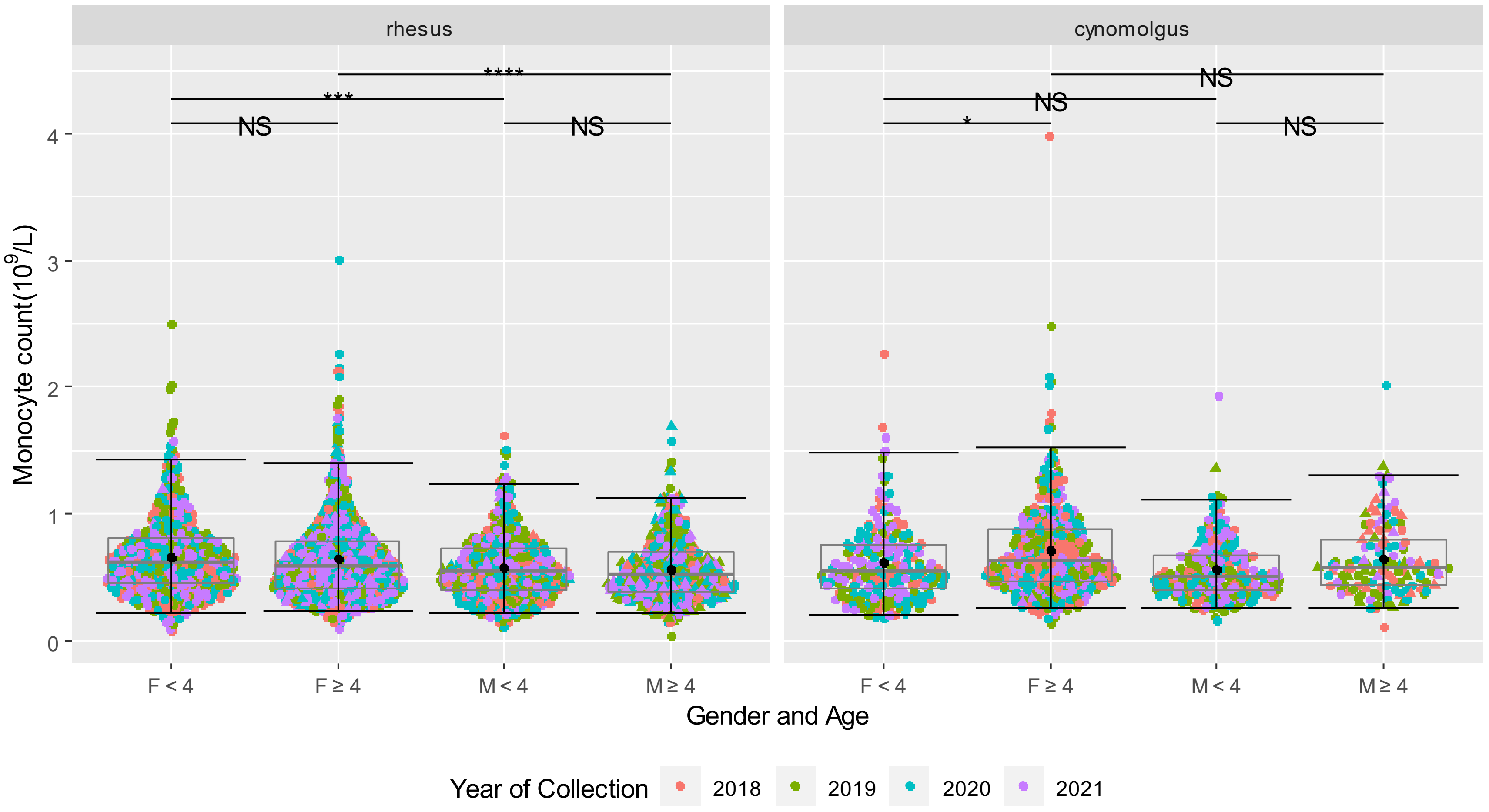

Figure S4 33. Monocyte Count according to gender and age in rhesus and cynomolgus macaques. Boxes indicate median and quartile ranges, black point indicates arithmetic mean and error bars indicate the normal range. Round symbols indicate animals with outdoor access and triangles indicate indoor housed animals

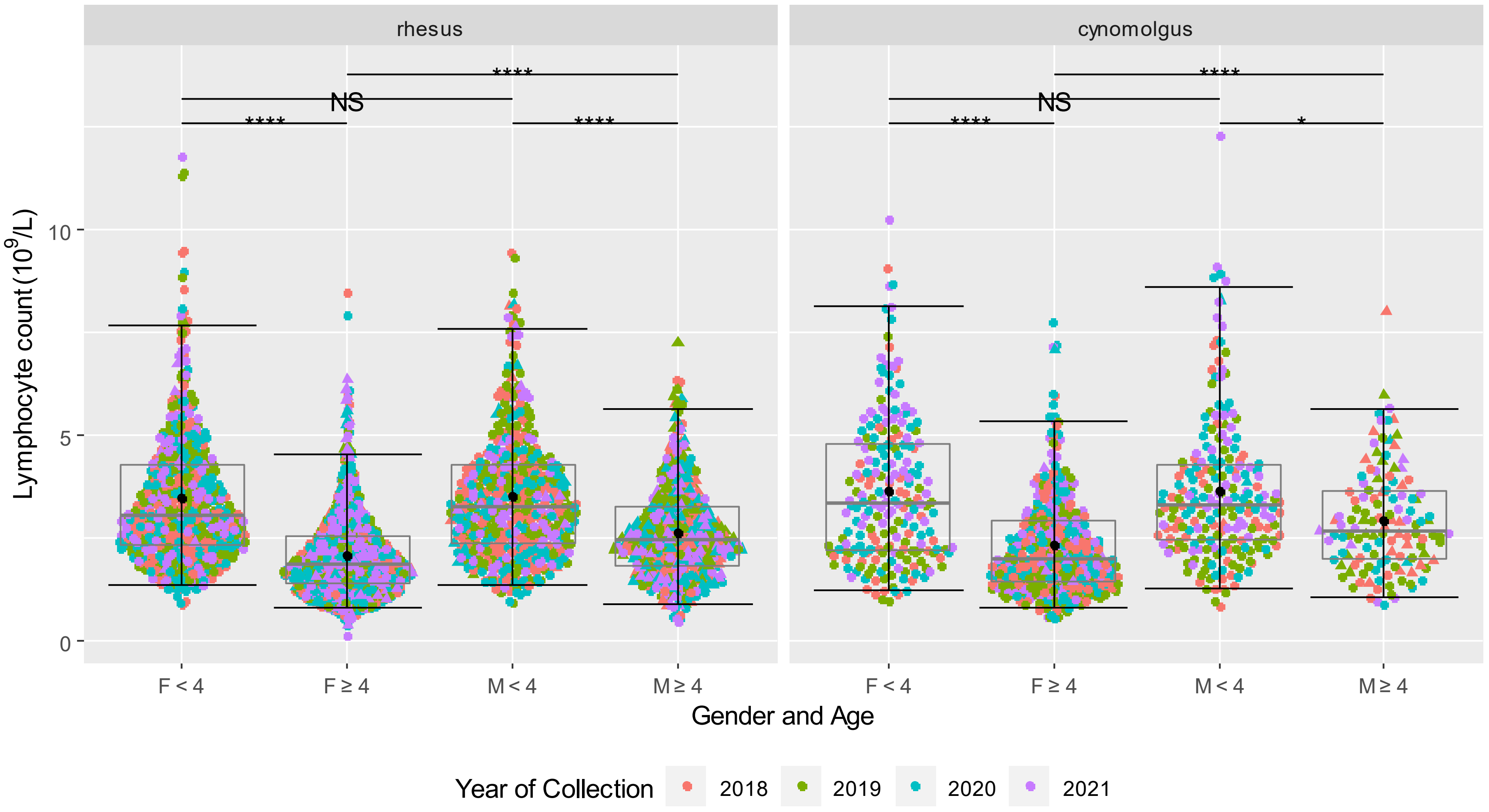

Figure S4 34. Lymphocyte Count according to gender and age in rhesus and cynomolgus macaques. Boxes indicate median and quartile ranges, black point indicates arithmetic mean and error bars indicate the normal range. Round symbols indicate animals with outdoor access and triangles indicate indoor housed animals

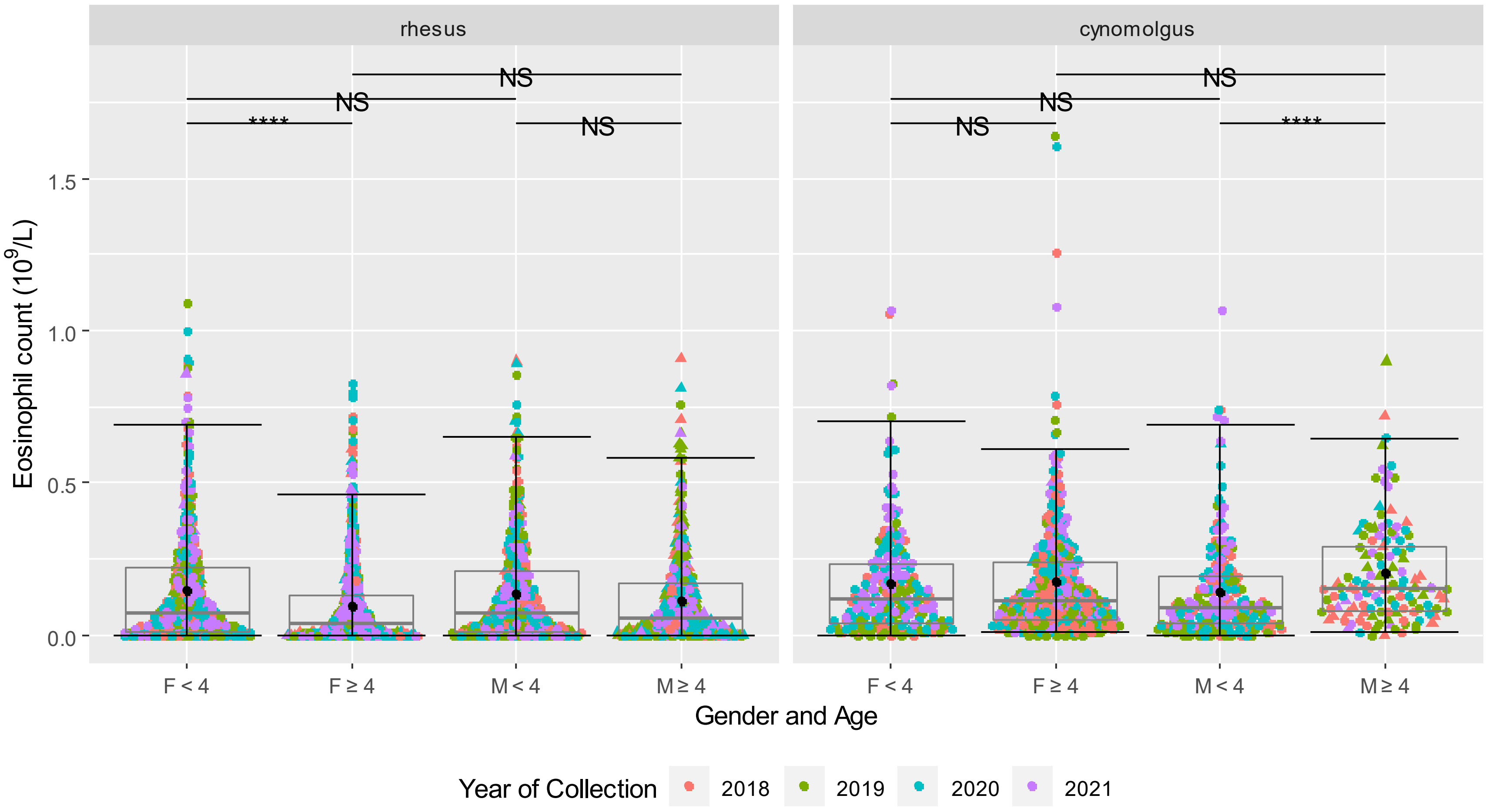

Figure S4 35. Eosinophil Count according to gender and age in rhesus and cynomolgus macaques. Boxes indicate median and quartile ranges, black point indicates arithmetic mean and error bars indicate the normal range. Round symbols indicate animals with outdoor access and triangles indicate indoor housed animals

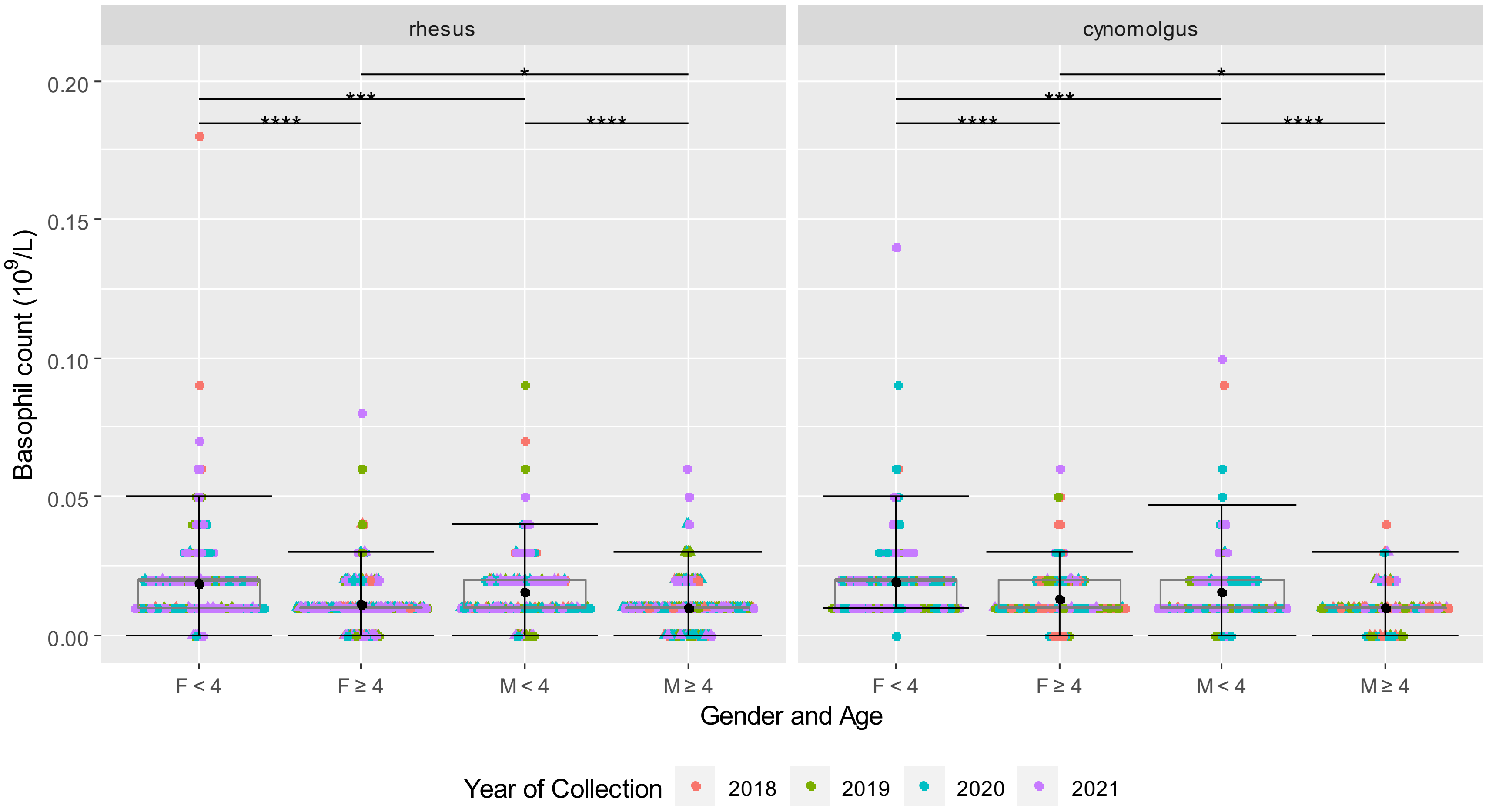

Figure S4 36. Basophil Count according to gender and age in rhesus and cynomolgus macaques. Boxes indicate median and quartile ranges, black point indicates arithmetic mean and error bars indicate the normal range. Round symbols indicate animals with outdoor access and triangles indicate indoor housed animals
