# Supplementary material for: Reference Intervals and Percentiles for Hematologic and Serum Biochemical Values in Captive Bred Rhesus (Macaca mulatta) and Cynomolgus Macaques (Macaca fascicularis)
Source: Animals (Basel). 2023 Jan 28;13(3):445. doi: 10.3390/ani13030445 (PMC9913310; doi:10.3390/ani13030445)
Supplement: Supplementary file 1 [file animals-13-00445-s001.zip › Figure S5.pdf]

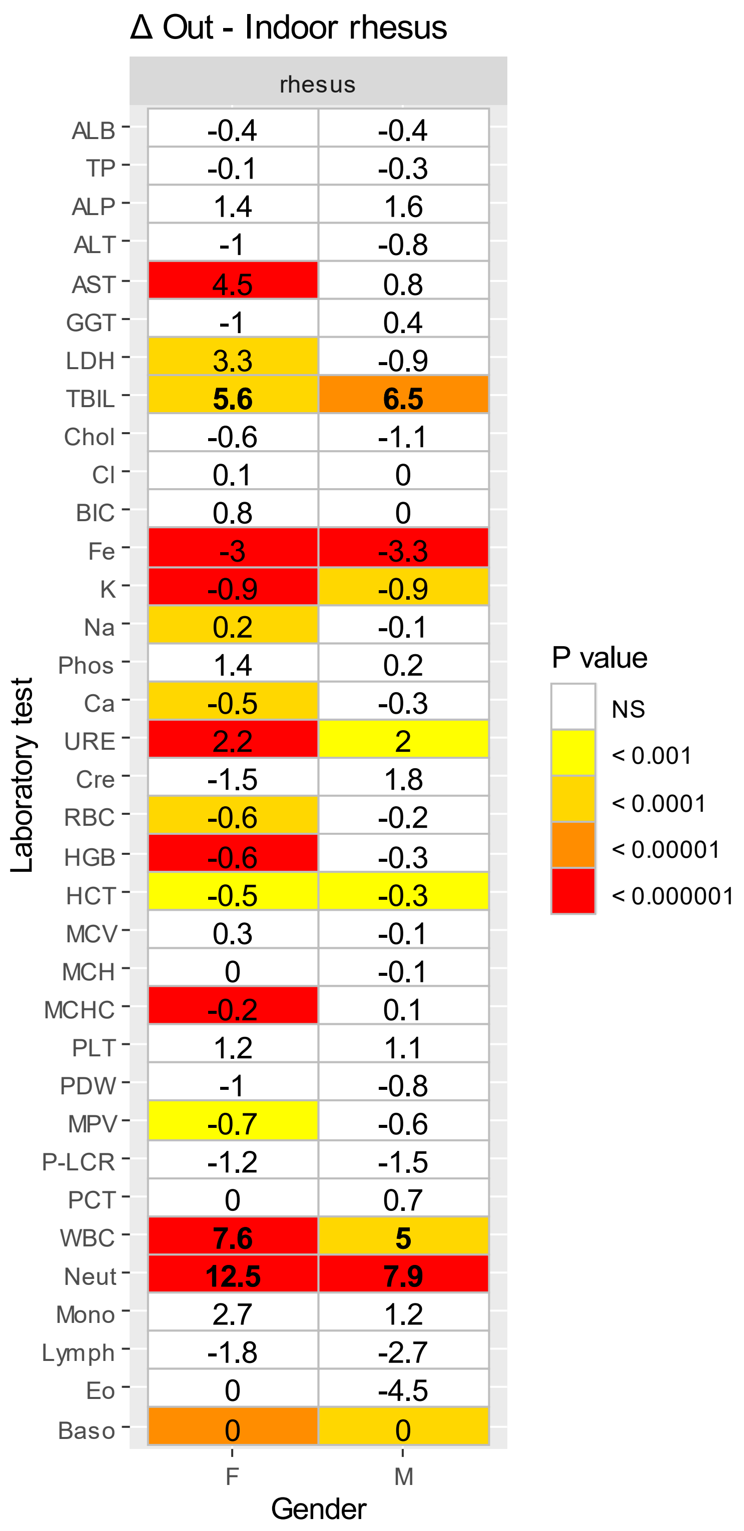


Figure S5A. Comparisons between adult rhesus macaques with or without outdoor access, numbers indicate percentual differences (Delta) between Outdoor – Indoor, Delta values greater than 5% are in bold.

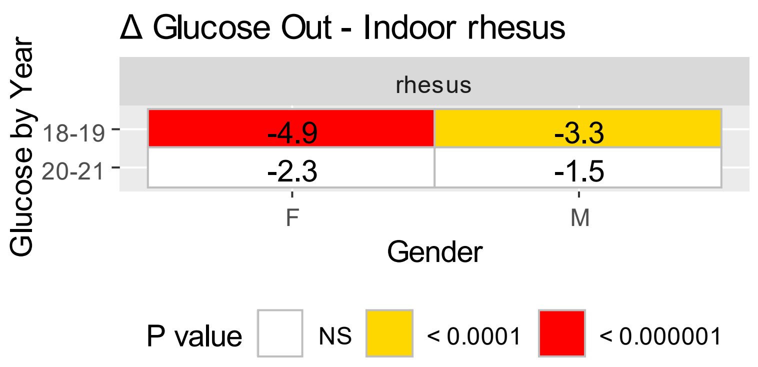


Figure S5B. Glucose comparison between 2018-2019 and 2020-201 in adult rhesus macaques with or without outdoor access, numbers indicate percentual differences (Delta) between Outdoor – Indoor, Delta values greater than 5% are in bold.
