# Supplementary material for: Reference Intervals and Percentiles for Hematologic and Serum Biochemical Values in Captive Bred Rhesus (Macaca mulatta) and Cynomolgus Macaques (Macaca fascicularis)
Source: Animals (Basel). 2023 Jan 28;13(3):445. doi: 10.3390/ani13030445 (PMC9913310; doi:10.3390/ani13030445)
Supplement: Supplementary file 1 [file animals-13-00445-s001.zip › Figure S6.pdf]

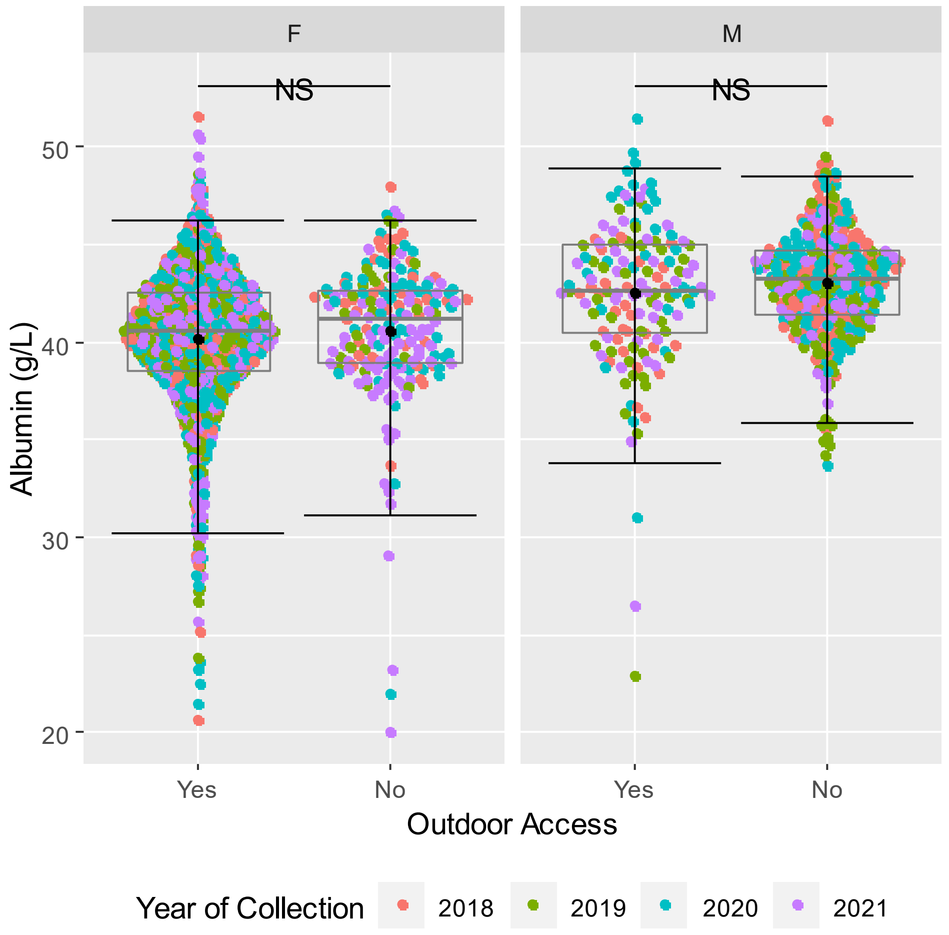

Figure S6 1. Albumin levels according to outdoor access in adult rhesus macaques. Boxes indicate median and quartile ranges, black point indicates arithmetic mean and error bars indicate the normal range.

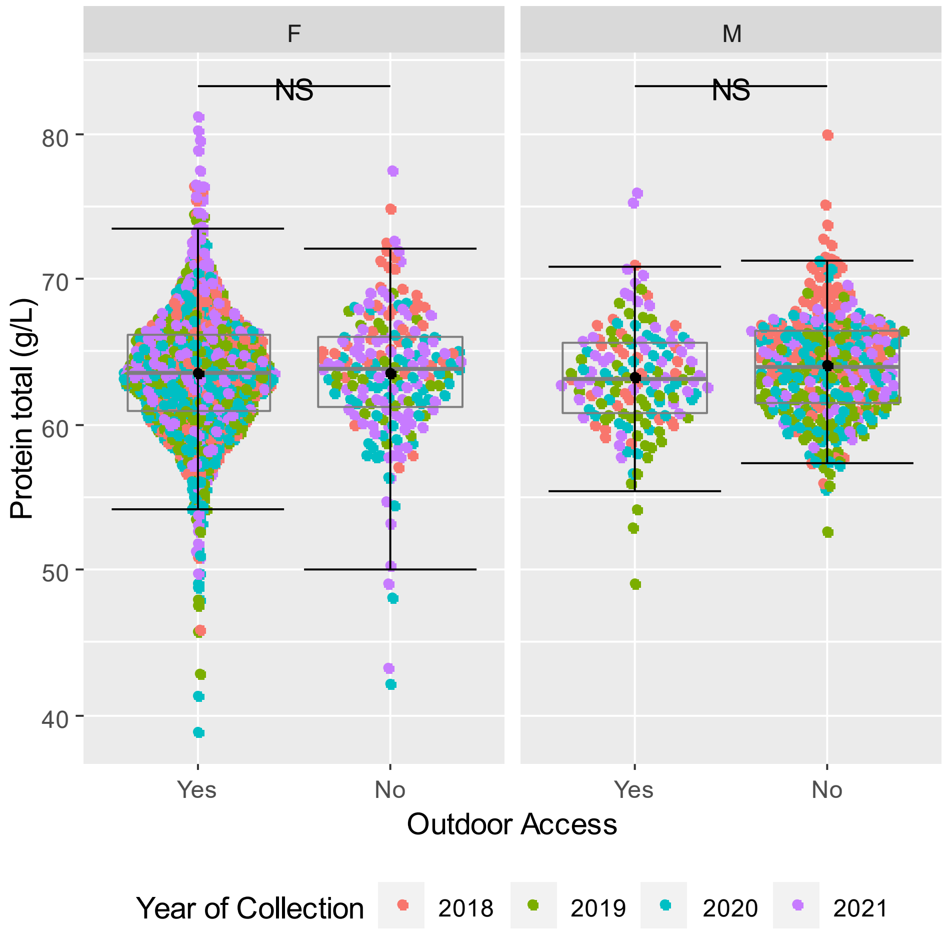

Figure S6 2. Total Protein levels according to outdoor access in adult rhesus macaques. Boxes indicate median and quartile ranges, black point indicates arithmetic mean and error bars indicate the normal range.

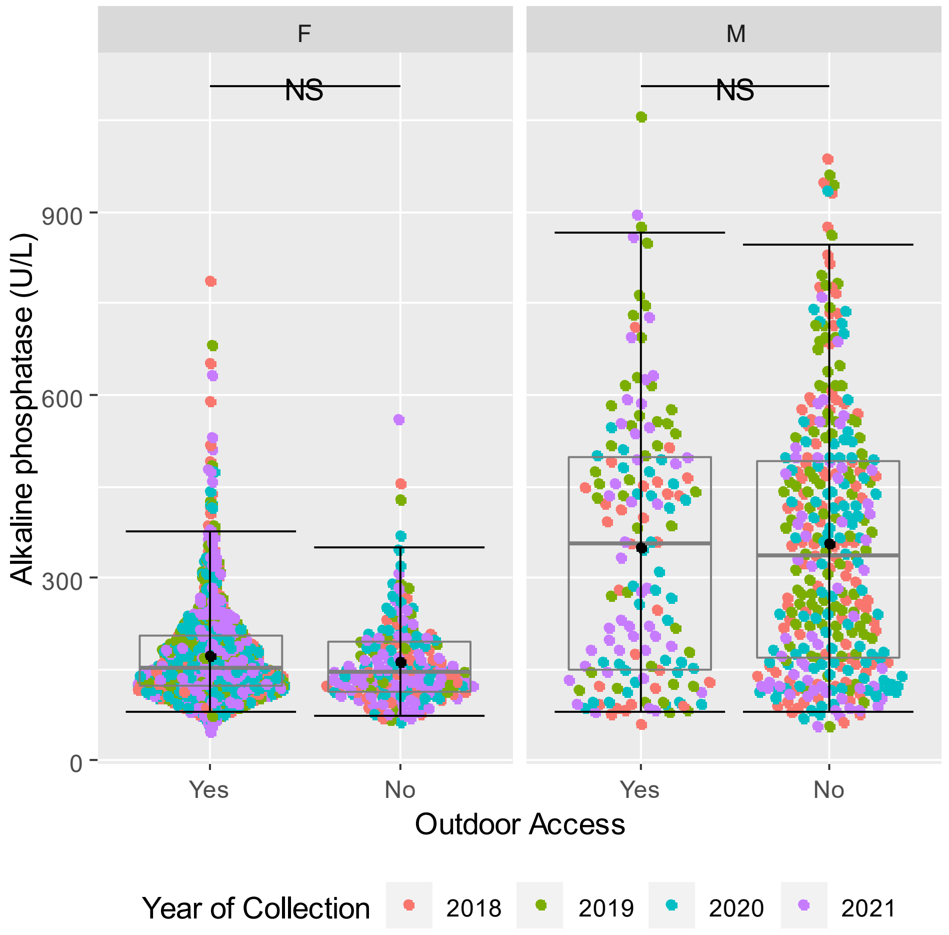

Figure S6 3. Alkaline Phosphatase levels according to period in adult rhesus macaques. Boxes indicate median and quartile ranges, black point indicates arithmetic mean and error bars indicate the normal range.

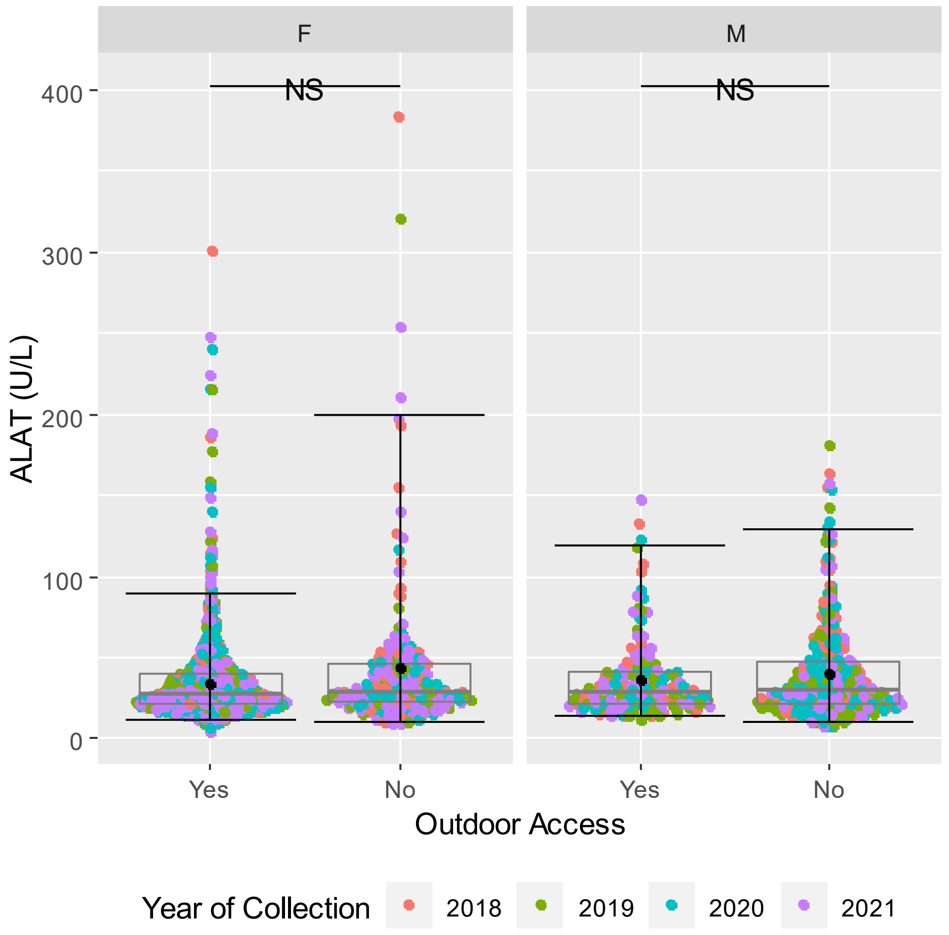

Figure S6 4. ALAT levels according to outdoor access in adult rhesus macaques. Boxes indicate median and quartile ranges, black point indicates arithmetic mean and error bars indicate the normal range.

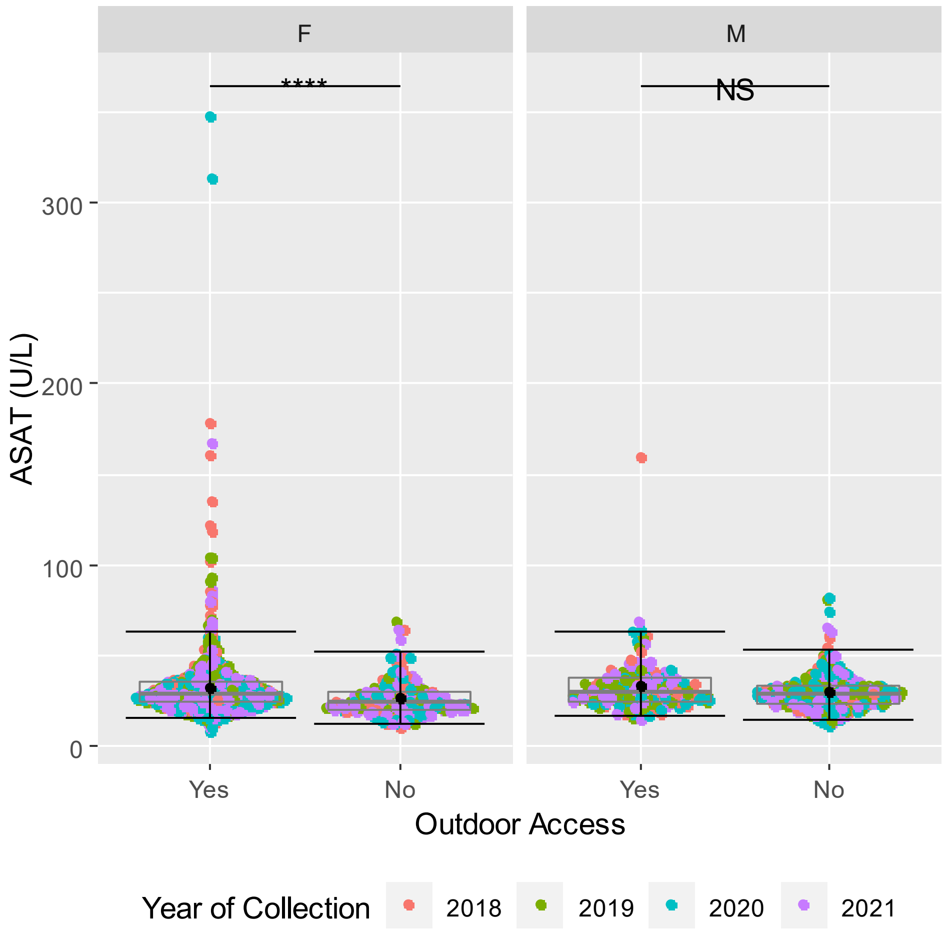

Figure S6 5. ASAT levels according to outdoor access in adult rhesus macaques. Boxes indicate median and quartile ranges, black point indicates arithmetic mean and error bars indicate the normal range.

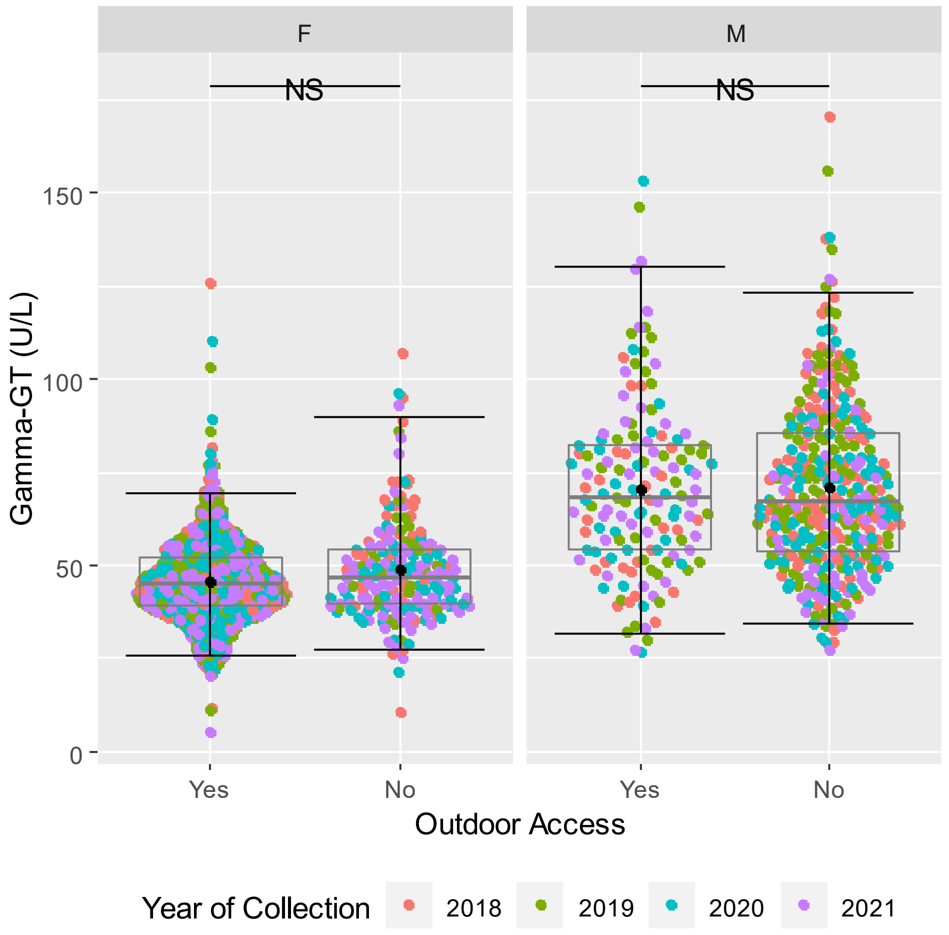

Figure S6 6. Gamma-GT levels according to outdoor access in adult rhesus macaques. Boxes indicate median and quartile ranges, black point indicates arithmetic mean and error bars indicate the normal range.

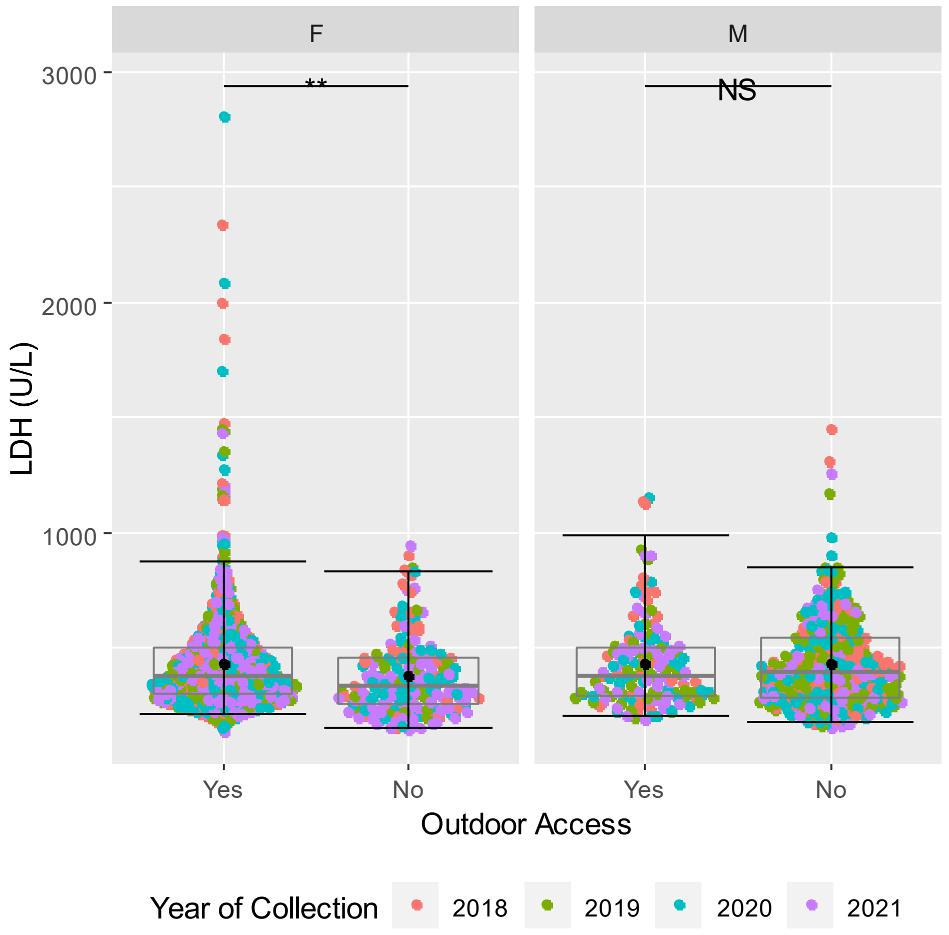

Figure S6 7. LDH levels according to outdoor access in adult rhesus macaques. Boxes indicate median and quartile ranges, black point indicates arithmetic mean and error bars indicate the normal range.

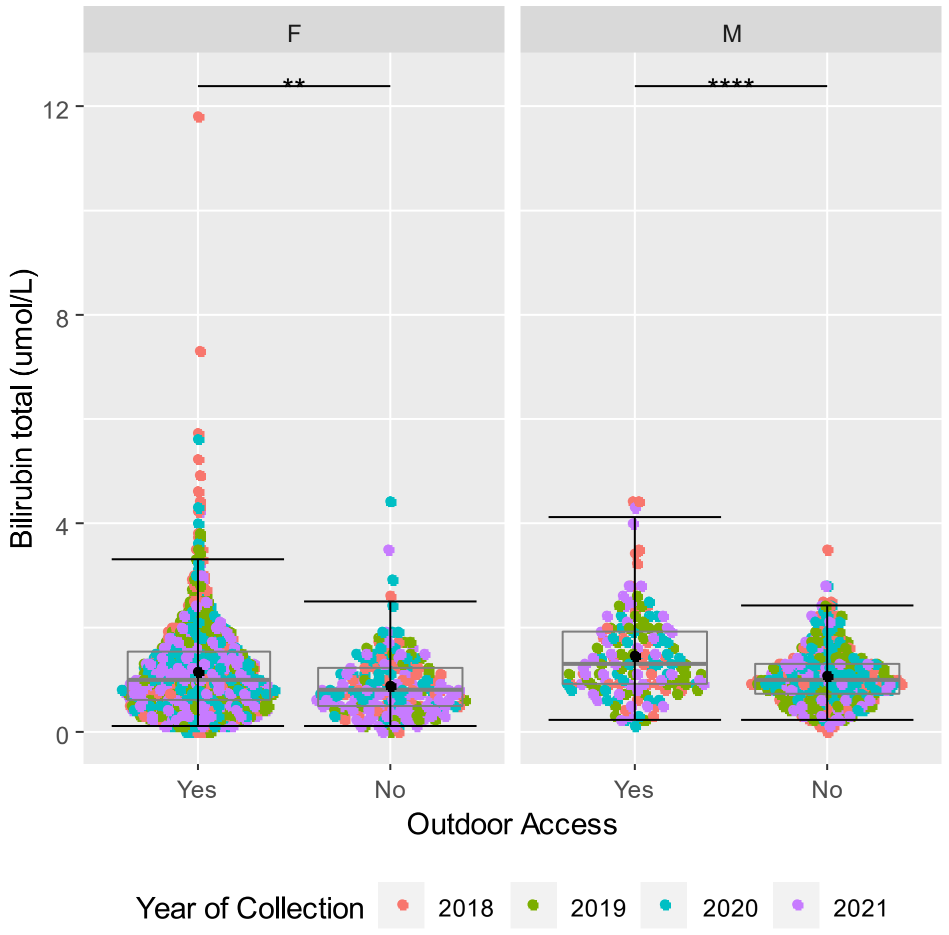

Figure S6 8. Total Bilirubin levels according to outdoor access in adult rhesus macaques. Boxes indicate median and quartile ranges, black point indicates arithmetic mean and error bars indicate the normal range.

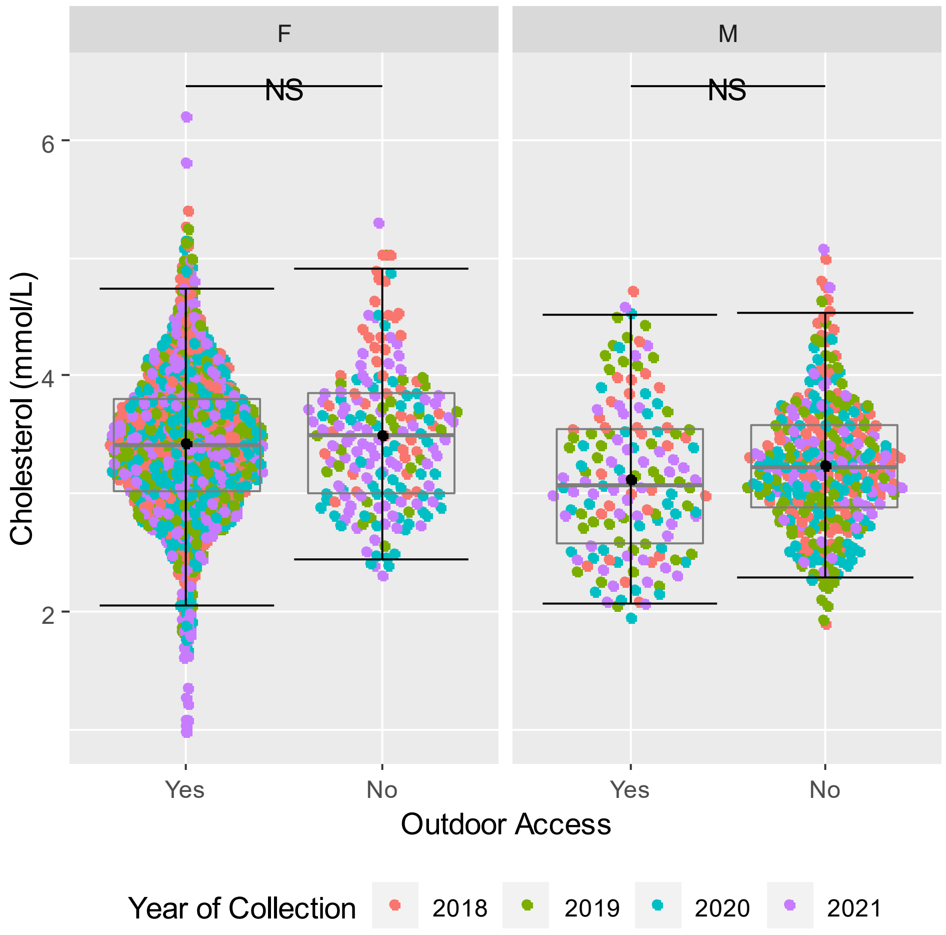

Figure S6 9. Cholesterol levels according to outdoor access in adult rhesus macaques. Boxes indicate median and quartile ranges, black point indicates arithmetic mean and error bars indicate the normal range.

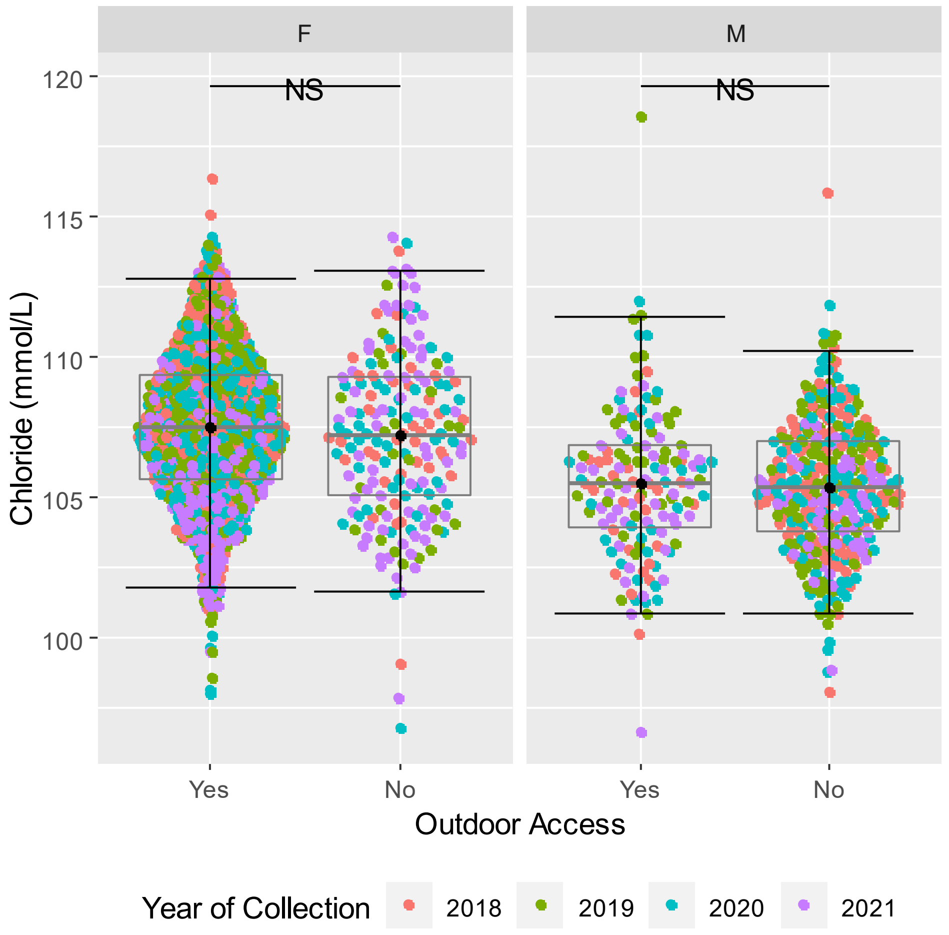

Figure S6 10. Chloride levels according to outdoor access in adult rhesus macaques. Boxes indicate median and quartile ranges, black point indicates arithmetic mean and error bars indicate the normal range.

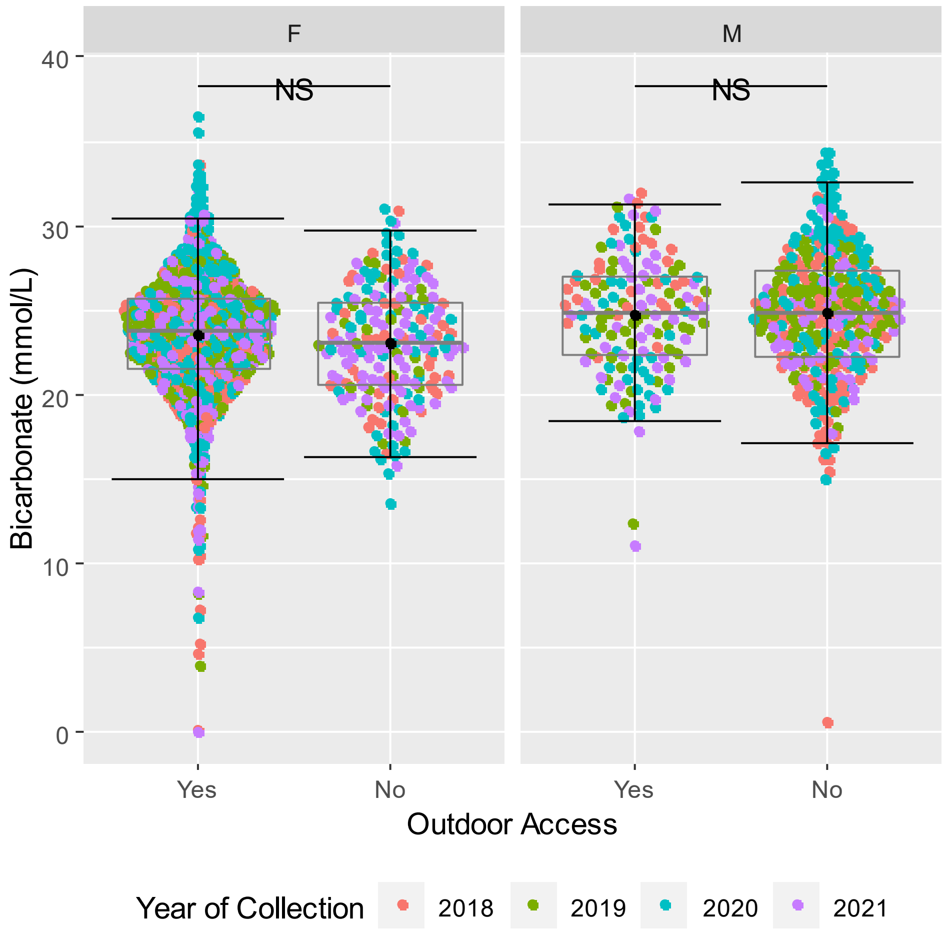

Figure S6 11. Bicarbonate levels according to outdoor access in adult rhesus macaques. Boxes indicate median and quartile ranges, black point indicates arithmetic mean and error bars indicate the normal range.

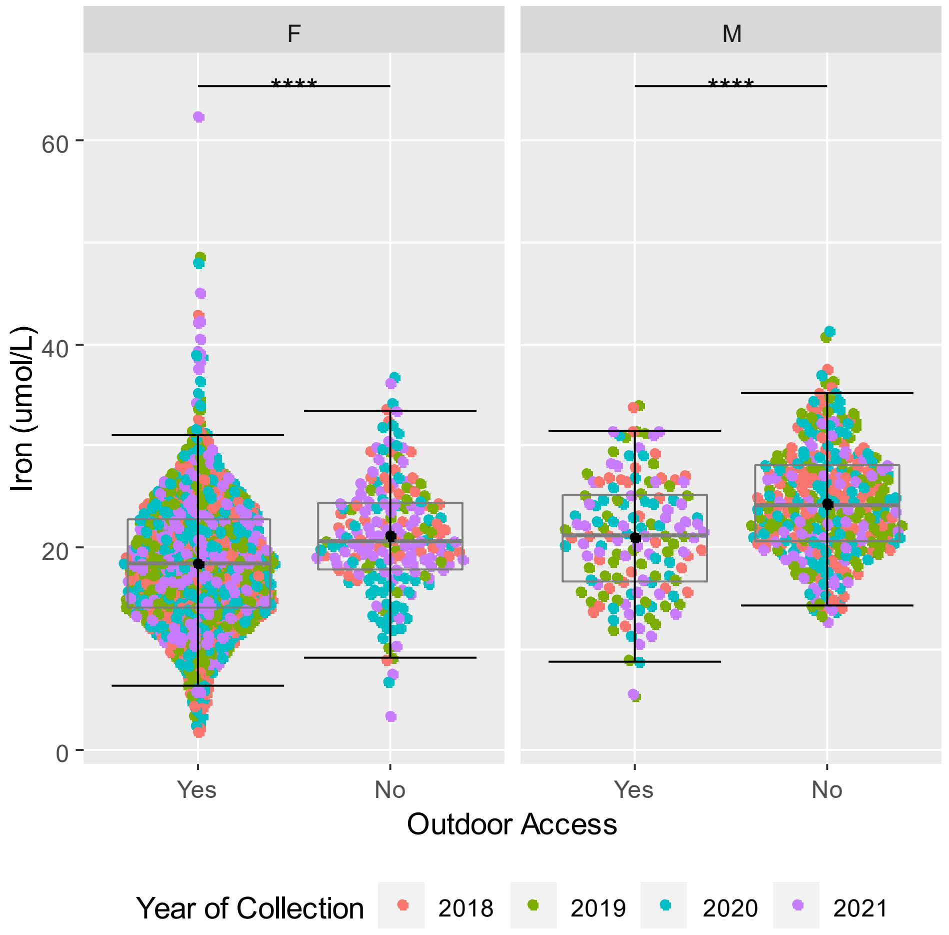

Figure S6 12. Iron levels according to outdoor access in adult rhesus macaques. Boxes indicate median and quartile ranges, black point indicates arithmetic mean and error bars indicate the normal range.

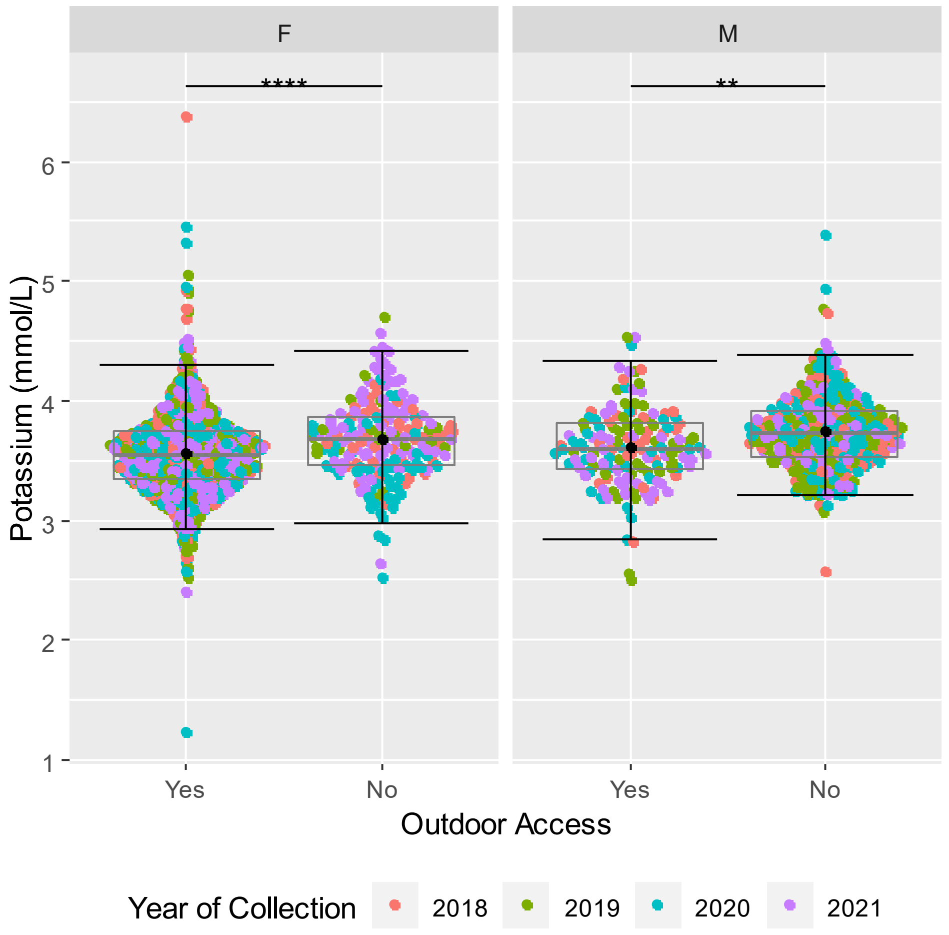

Figure S6 13. Potassium levels according to outdoor access in adult rhesus macaques. Boxes indicate median and quartile ranges, black point indicates arithmetic mean and error bars indicate the normal range.

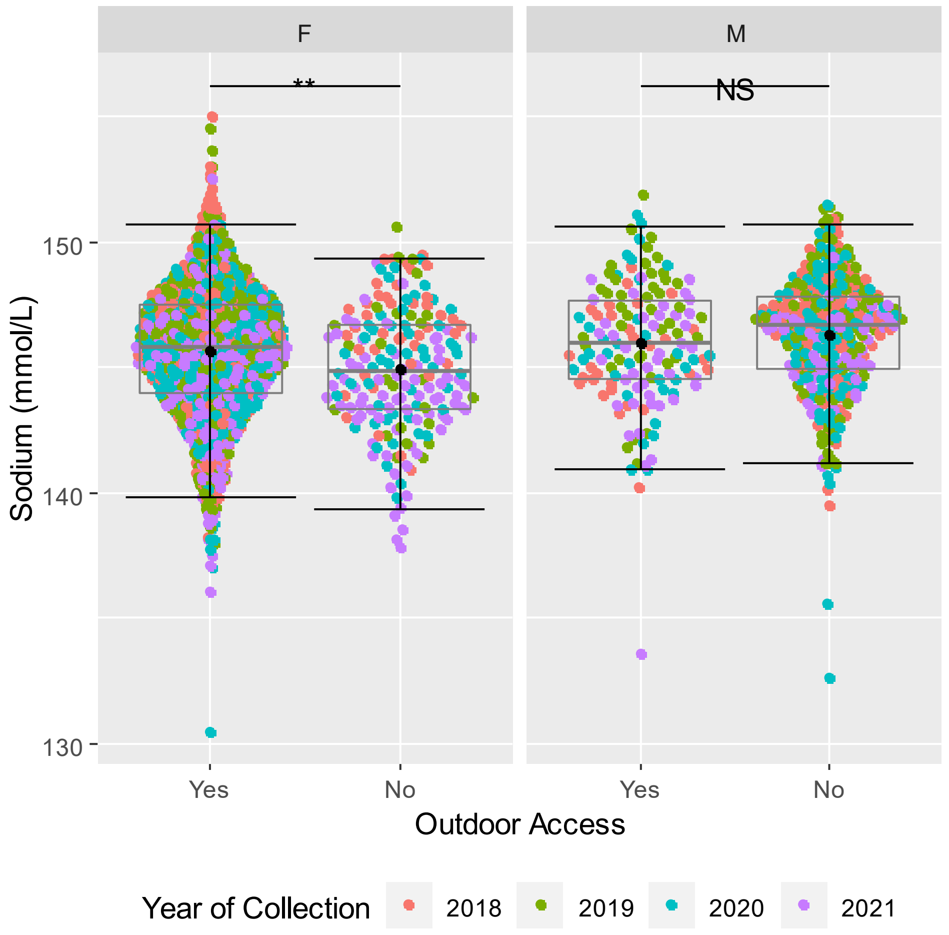

Figure S6 14. Sodium levels according to outdoor access in adult rhesus macaques. Boxes indicate median and quartile ranges, black point indicates arithmetic mean and error bars indicate the normal range.

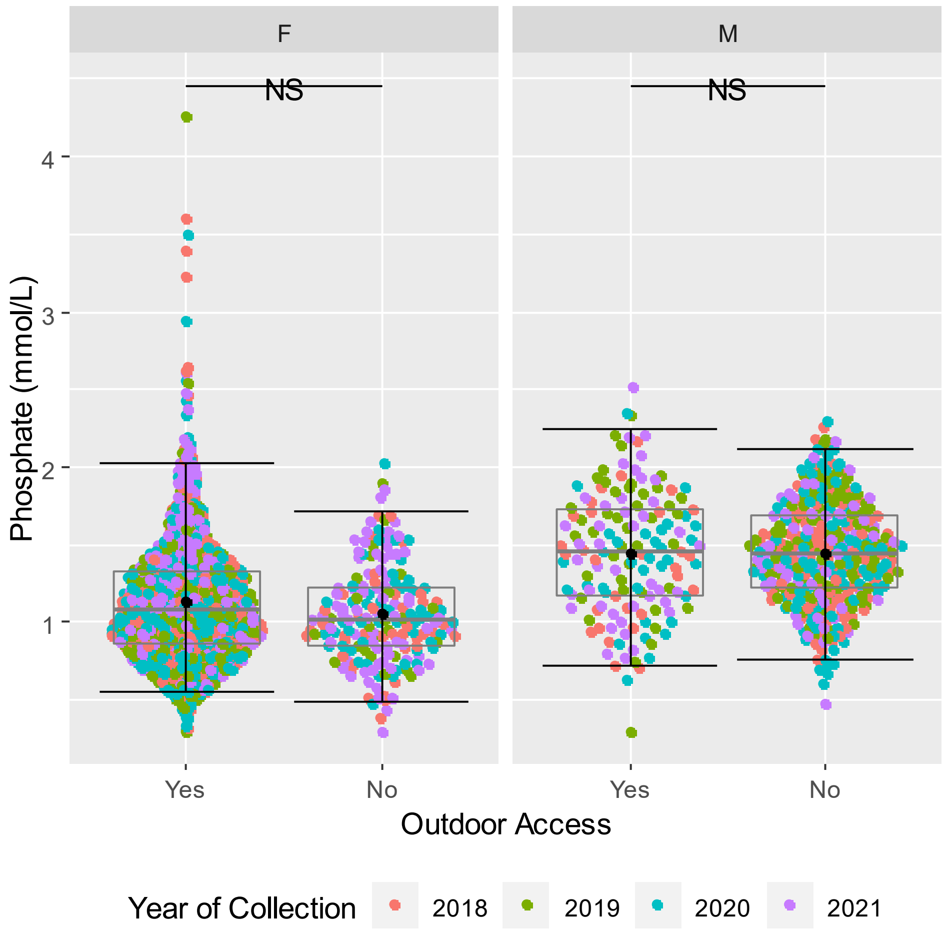

Figure S6 15. Phosphate levels according to outdoor access in adult rhesus macaques. Boxes indicate median and quartile ranges, black point indicates arithmetic mean and error bars indicate the normal range.

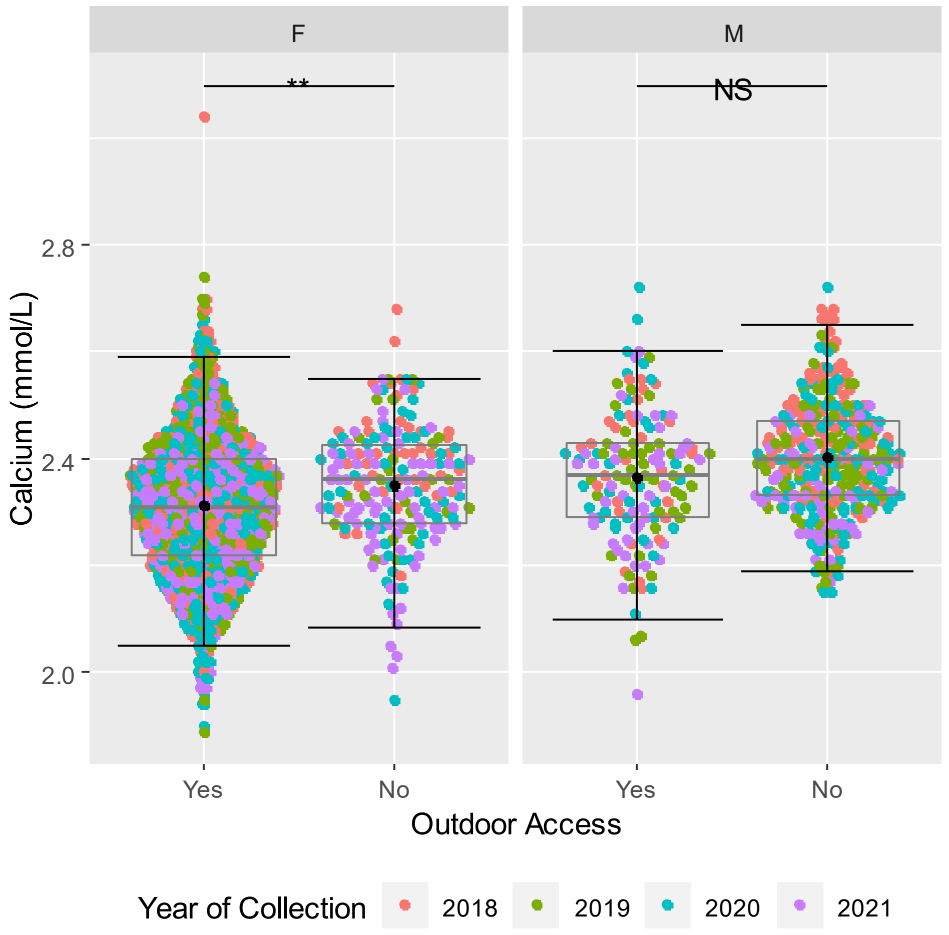

Figure S6 16. Calcium levels according to outdoor access in adult rhesus macaques. Boxes indicate median and quartile ranges, black point indicates arithmetic mean and error bars indicate the normal range.

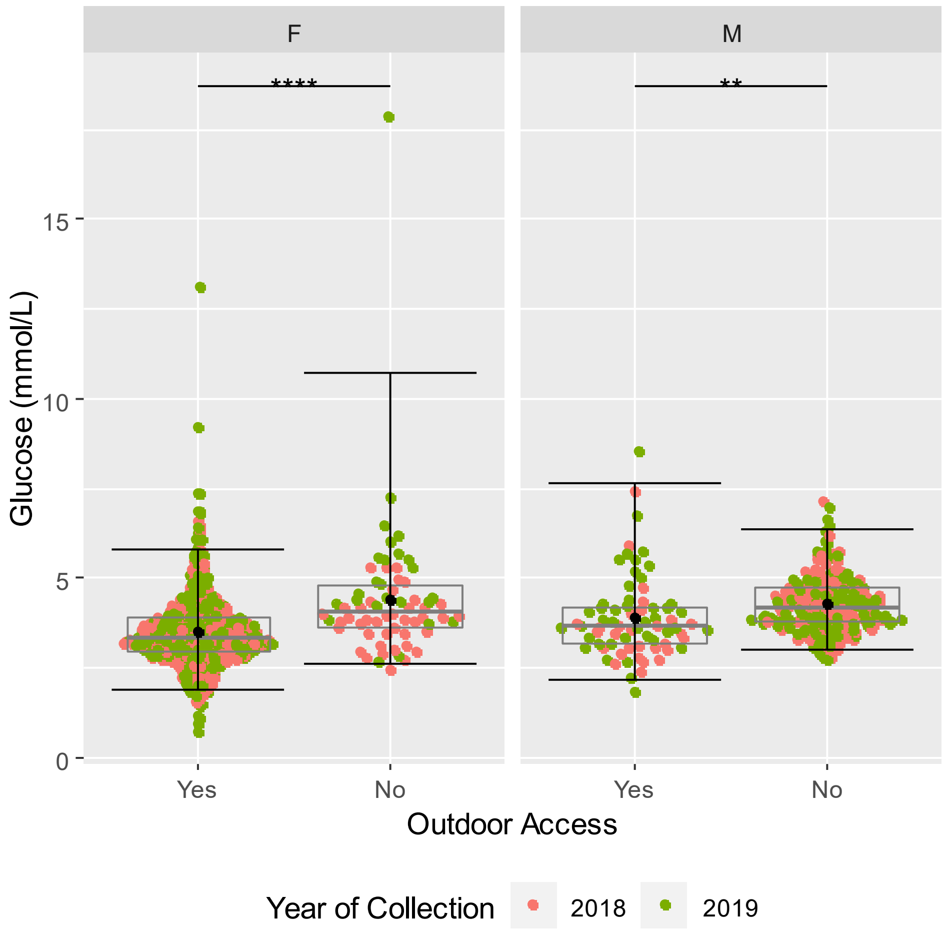

Figure S6 17A. Glucose levels in 2018-2019 according to outdoor access in adult rhesus macaques. Boxes indicate median and quartile ranges, black point indicates arithmetic mean and error bars indicate the normal range.

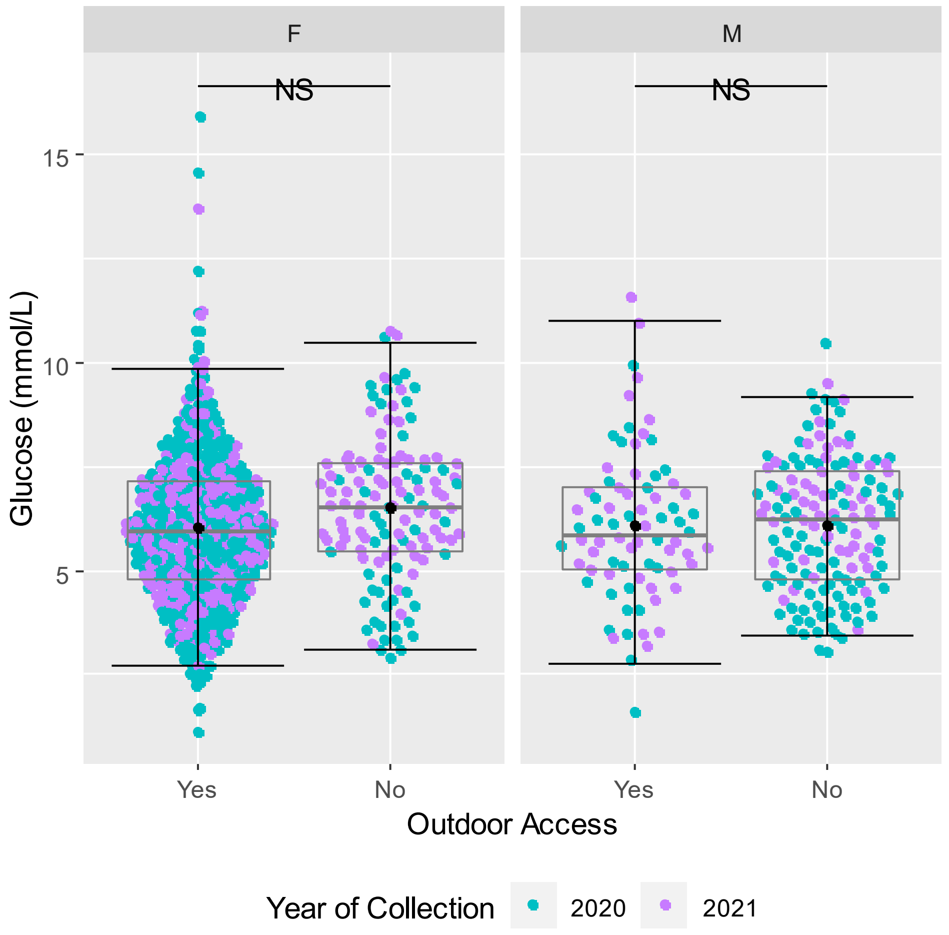

Figure S6 17B. Glucose levels in 2020-2021 according to outdoor access in adult rhesus macaques. Boxes indicate median and quartile ranges, black point indicates arithmetic mean and error bars indicate the normal range.

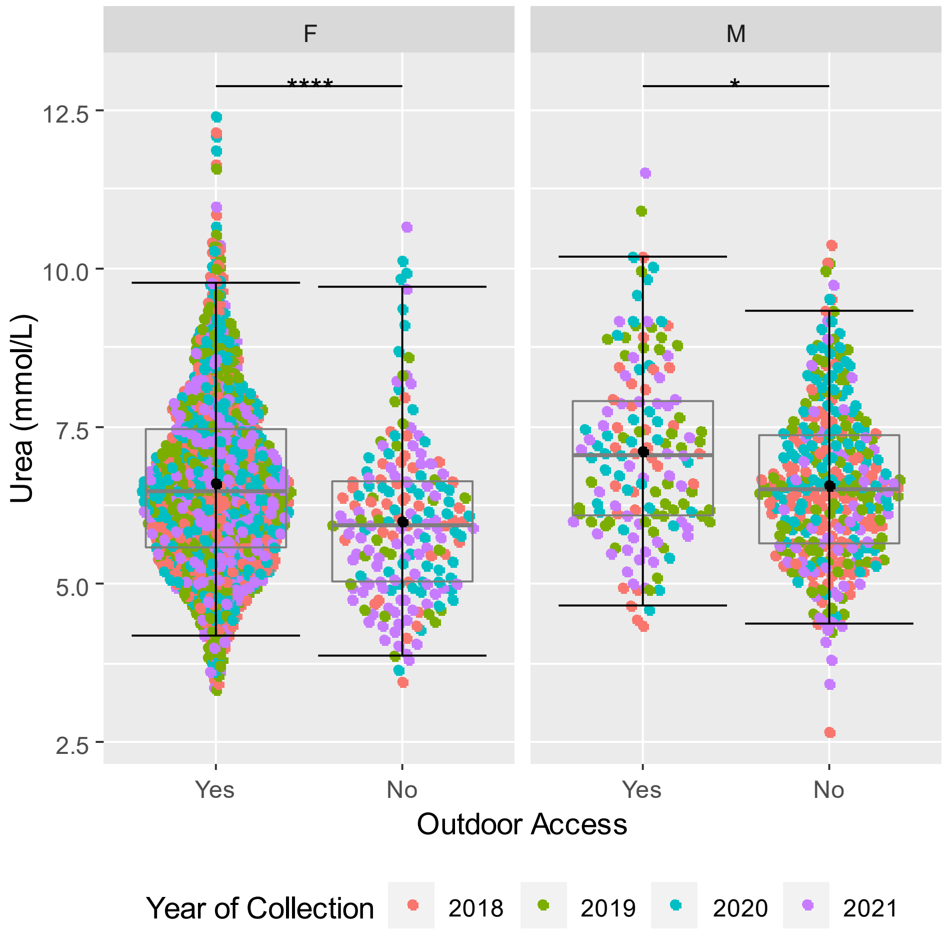

Figure S6 18. Urea levels according to outdoor access in adult rhesus macaques. Boxes indicate median and quartile ranges, black point indicates arithmetic mean and error bars indicate the normal range.

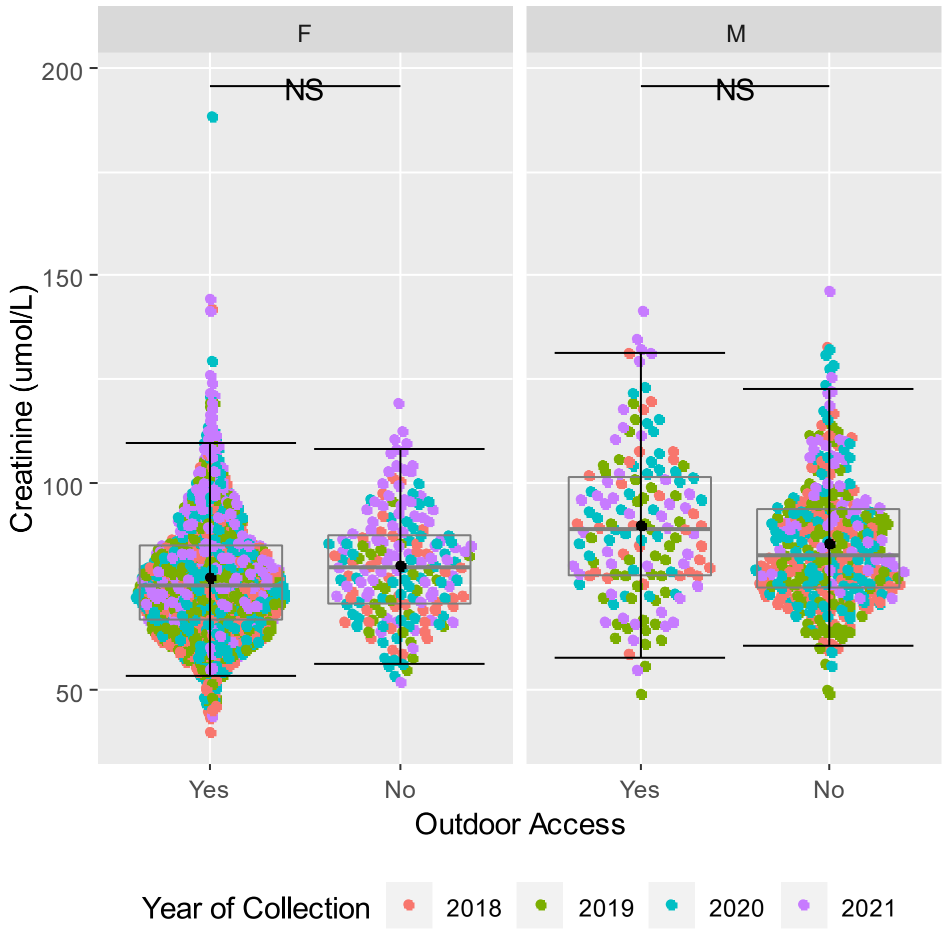

Figure S6 19. Creatinine levels according to outdoor access in adult rhesus macaques. Boxes indicate median and quartile ranges, black point indicates arithmetic mean and error bars indicate the normal range.

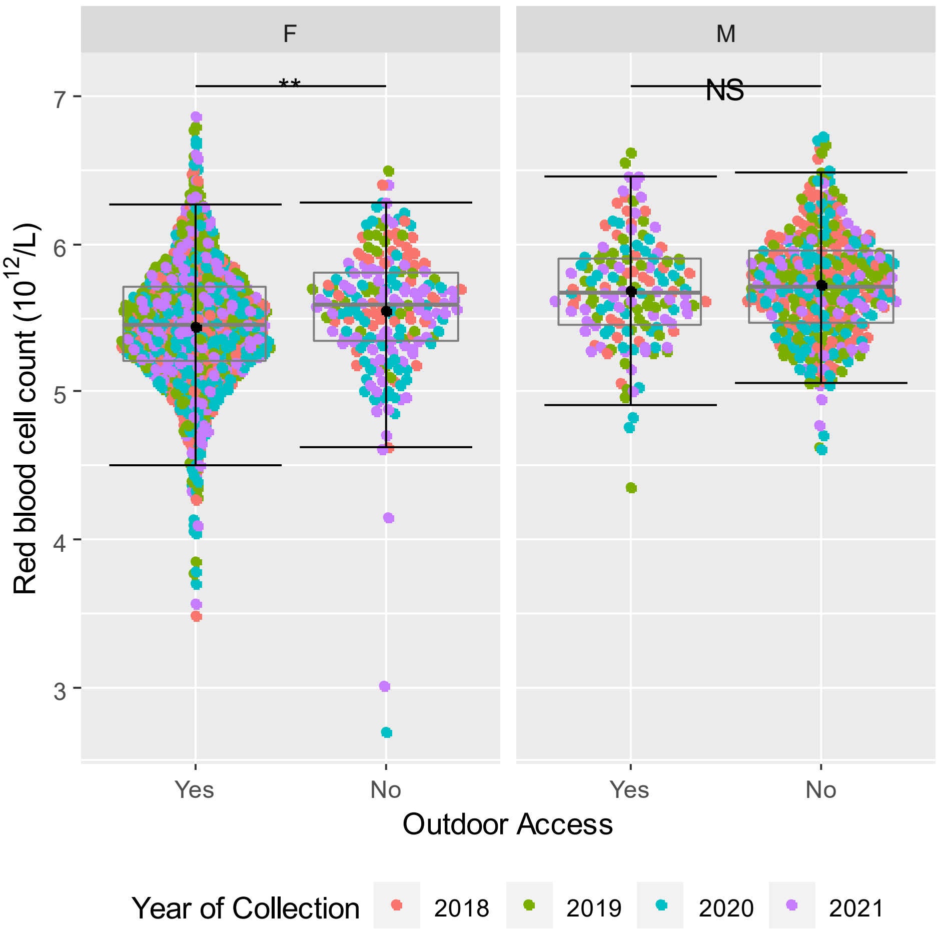

Figure S6 20. Red Blood Cell count according to outdoor access in adult rhesus macaques. Boxes indicate median and quartile ranges, black point indicates arithmetic mean and error bars indicate the normal range.

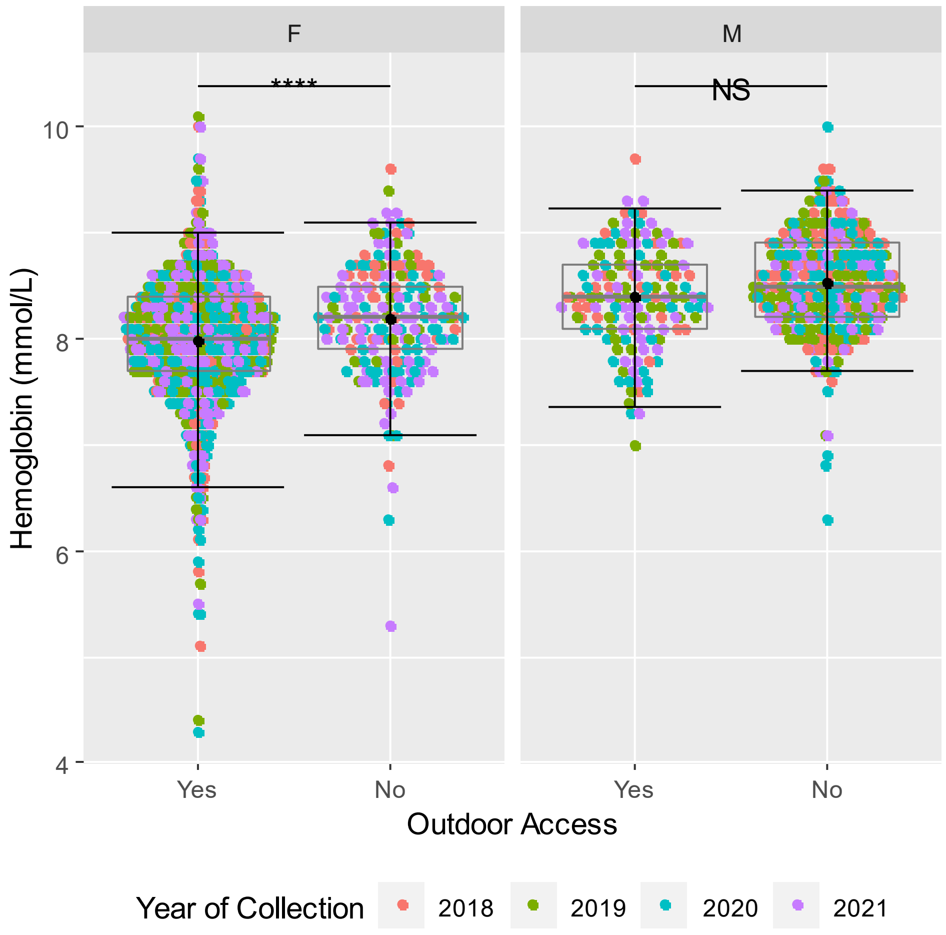

Figure S6 21. Hemoglobin levels according to outdoor access in adult rhesus macaques. Boxes indicate median and quartile ranges, black point indicates arithmetic mean and error bars indicate the normal range.

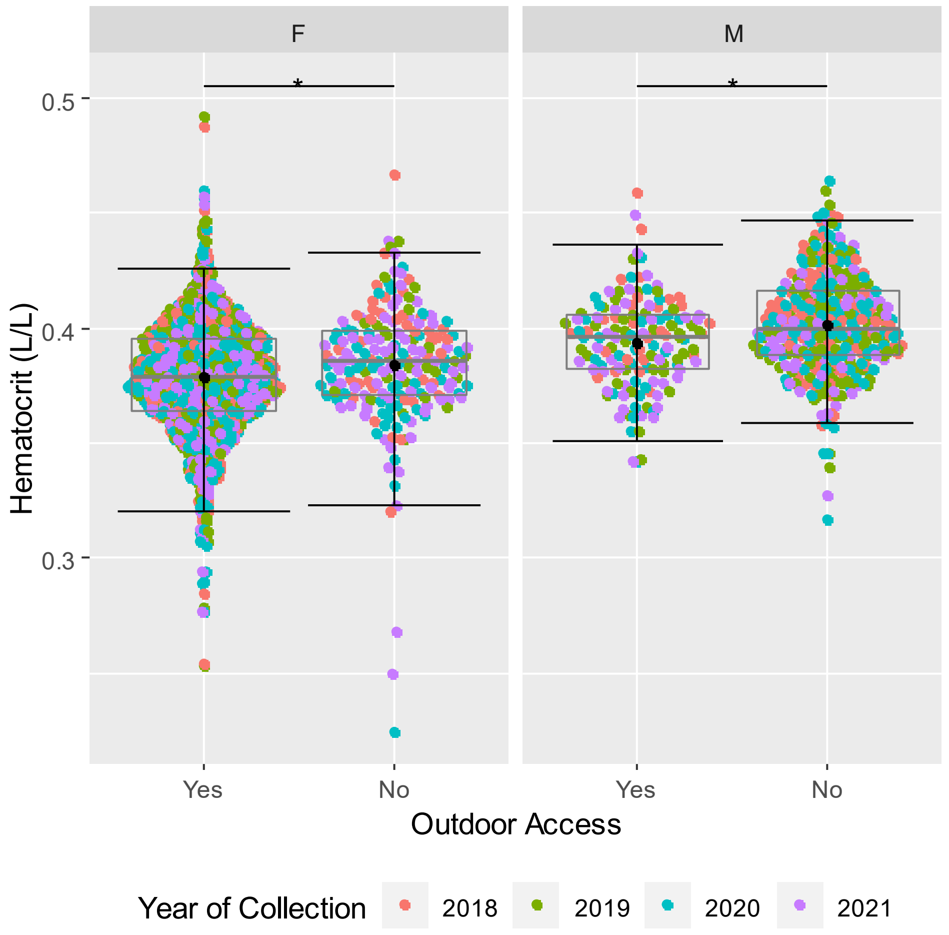

Figure S6 22. Hematocrit levels according to outdoor access in adult rhesus macaques. Boxes indicate median and quartile ranges, black point indicates arithmetic mean and error bars indicate the normal range.

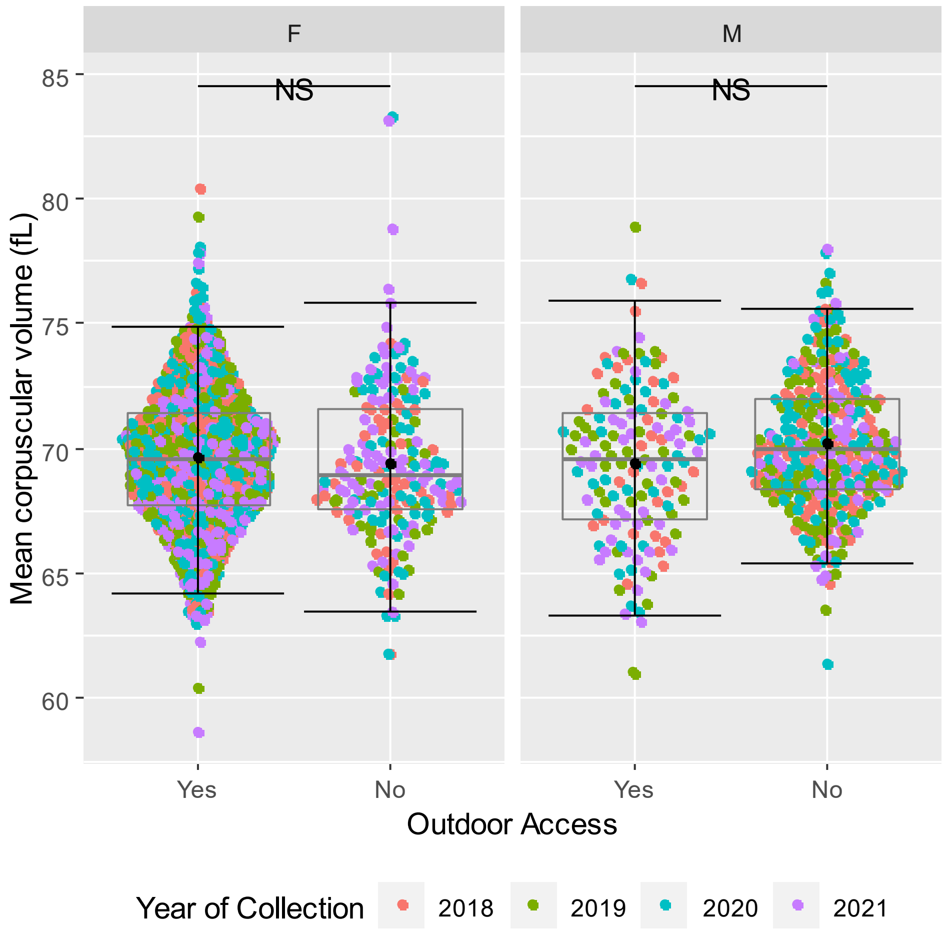

Figure S6 23. Mean Corpuscular Volume according to outdoor access in adult rhesus macaques. Boxes indicate median and quartile ranges, black point indicates arithmetic mean and error bars indicate the normal range.

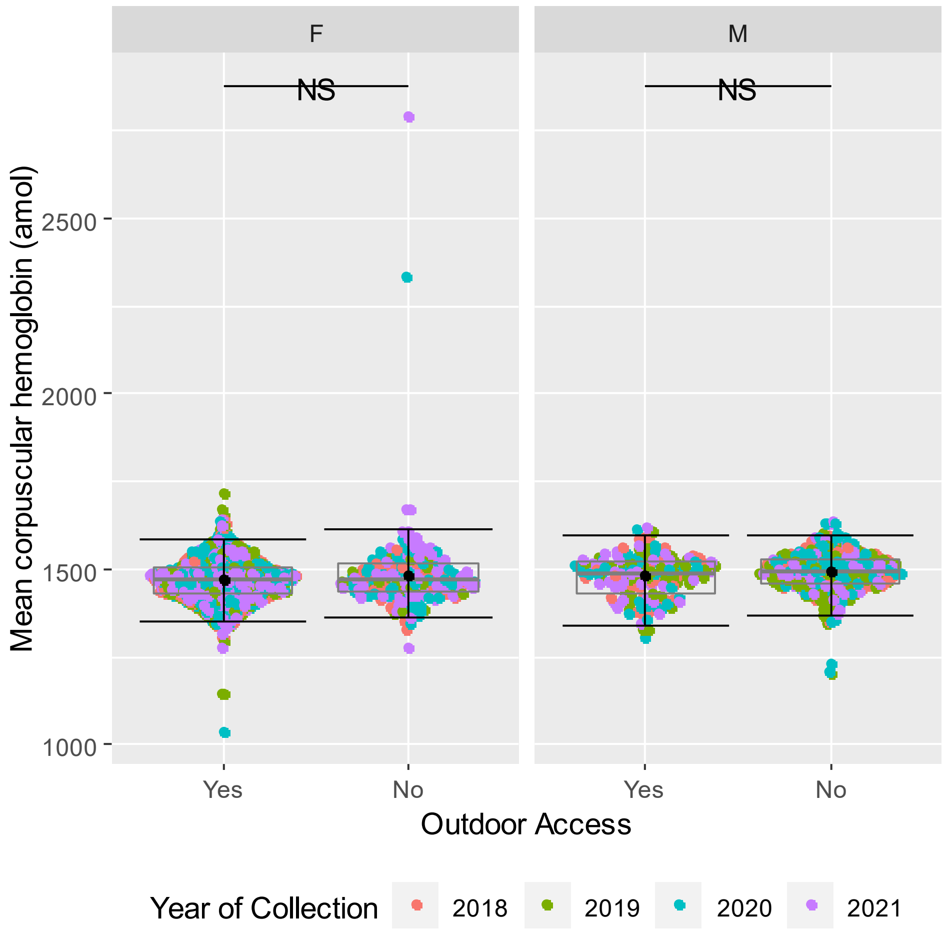

Figure S6 24. Mean Corpuscular Hemoglobin according to outdoor access in adult rhesus macaques. Boxes indicate median and quartile ranges, black point indicates arithmetic mean and error bars indicate the normal range.

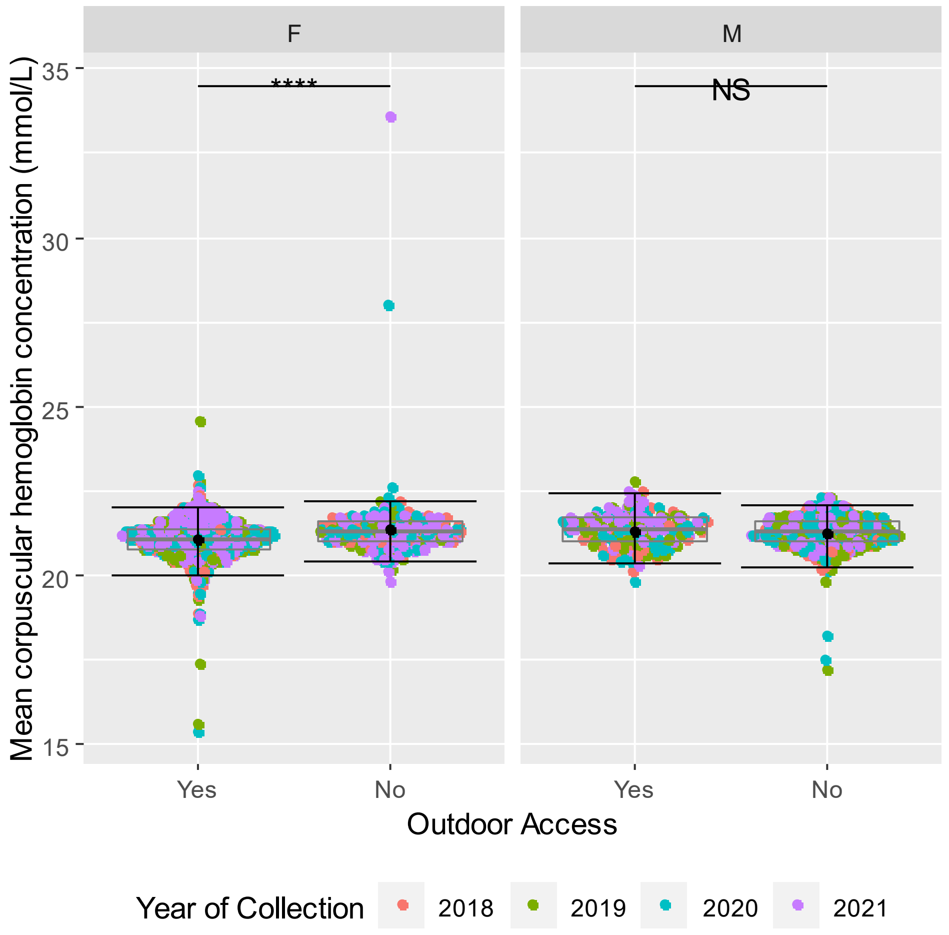

Figure S6 25. Mean Corpuscular Hemoglobin Concentration according to outdoor access in adult rhesus macaques. Boxes indicate median and quartile ranges, black point indicates arithmetic mean and error bars indicate the normal range.

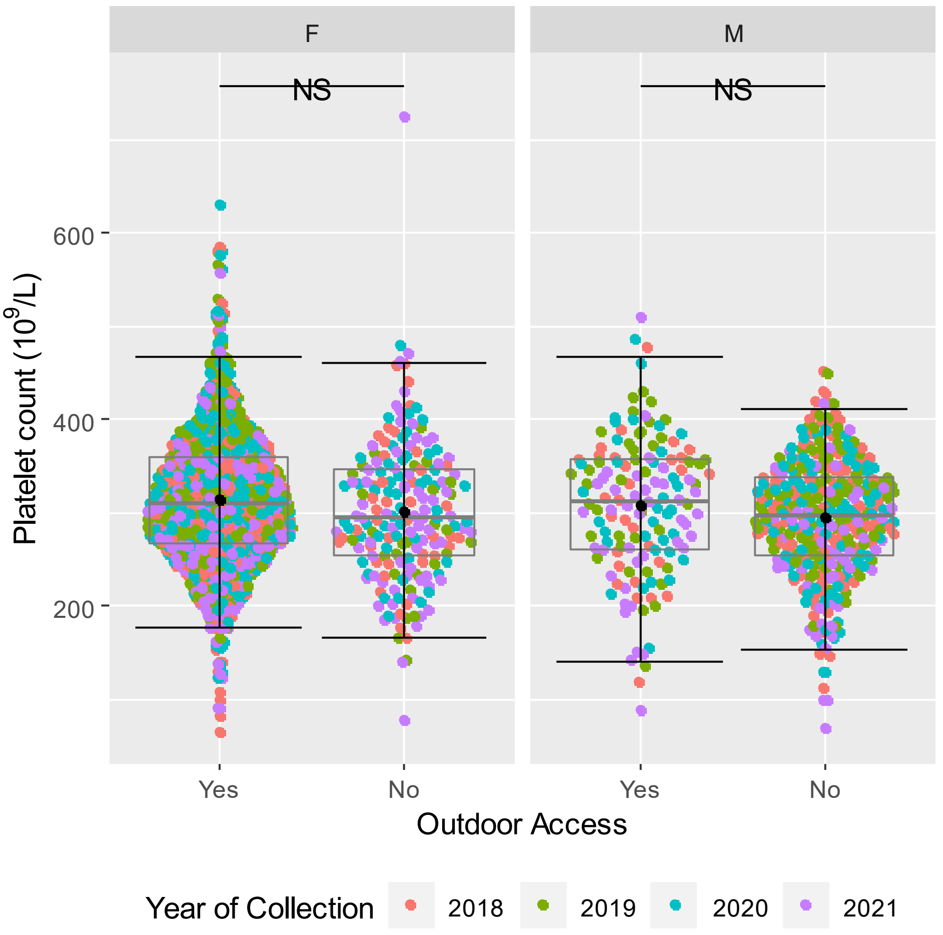

Figure S6 26. Platelet Count according to outdoor access in adult rhesus macaques. Boxes indicate median and quartile ranges, black point indicates arithmetic mean and error bars indicate the normal range.

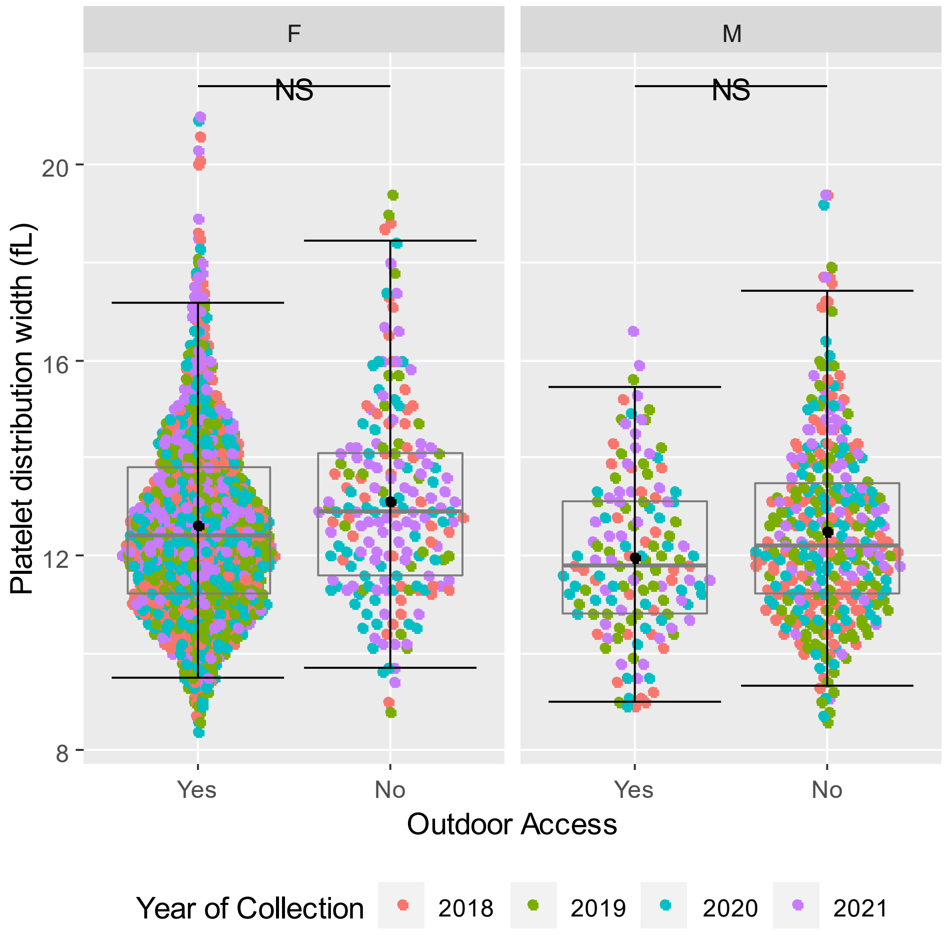

Figure S6 27. Platelet Distribution Width according to outdoor access in adult rhesus macaques. Boxes indicate median and quartile ranges, black point indicates arithmetic mean and error bars indicate the normal range.

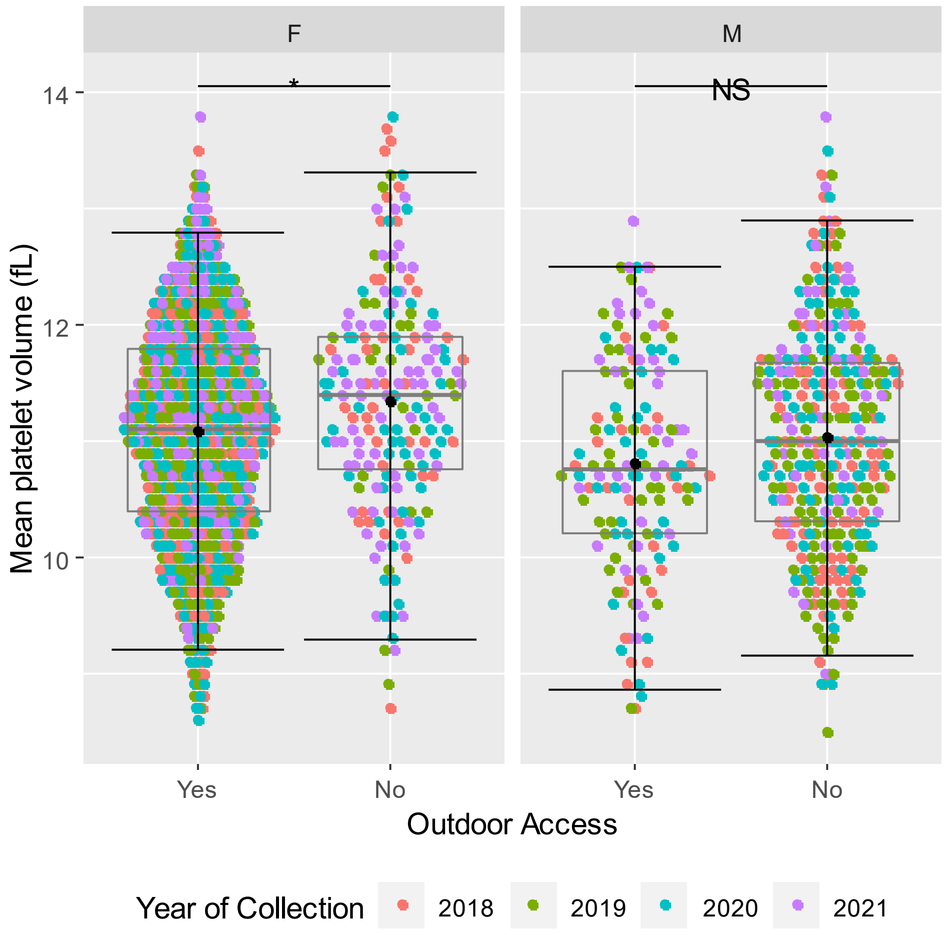

Figure S6 28. Mean Platelet Volume according to outdoor access in adult rhesus macaques. Boxes indicate median and quartile ranges, black point indicates arithmetic mean and error bars indicate the normal range.

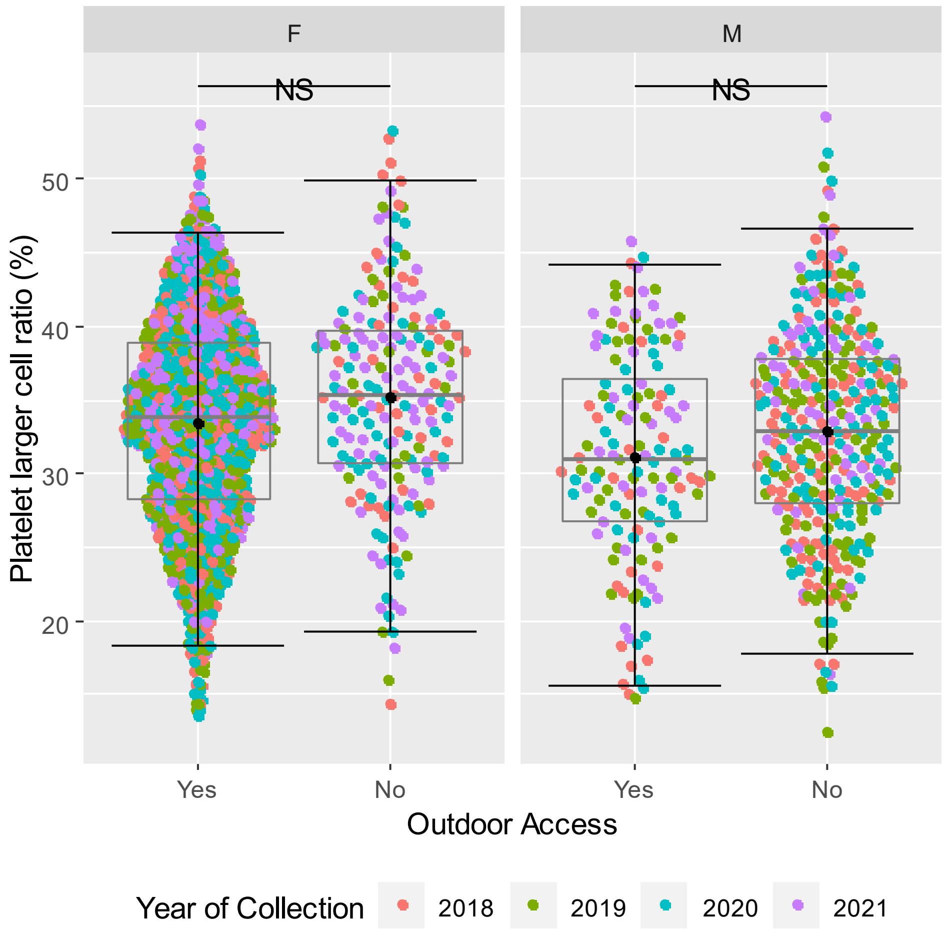

Figure S6 29. Platelet Larger Cell Ratio according to outdoor access in adult rhesus macaques. Boxes indicate median and quartile ranges, black point indicates arithmetic mean and error bars indicate the normal range.

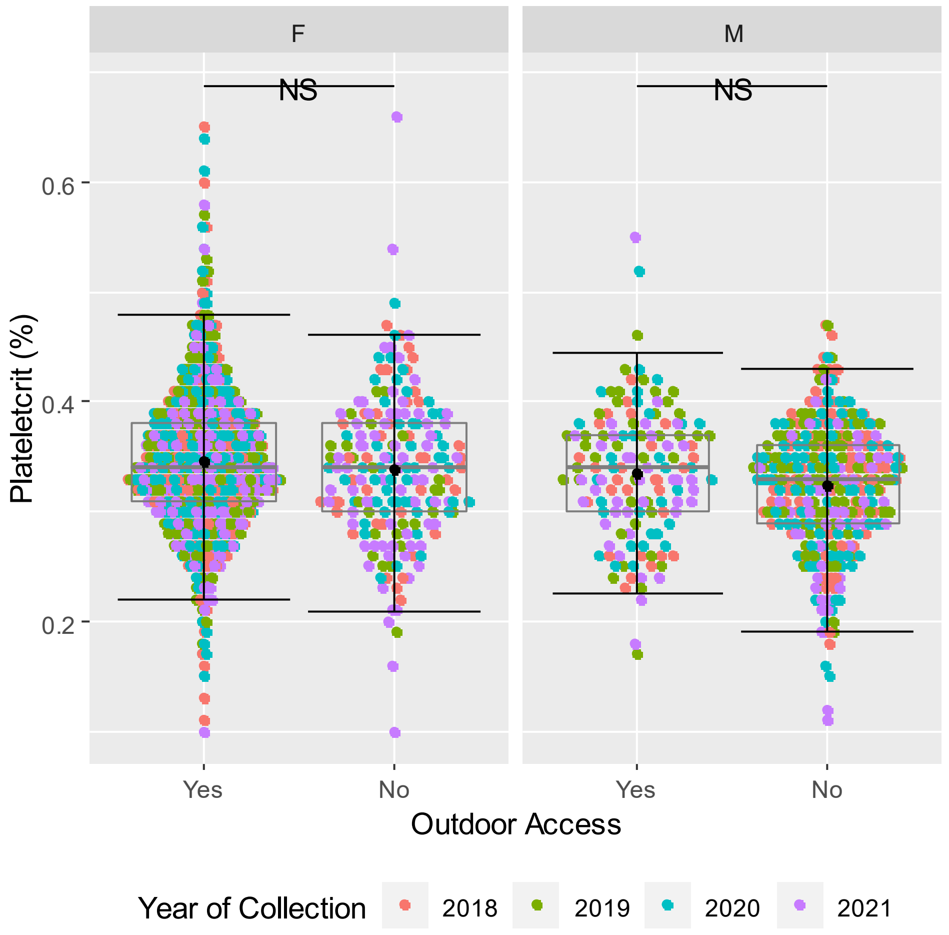

Figure S6 30. Plateletcrit according to outdoor access in adult rhesus macaques. Boxes indicate median and quartile ranges, black point indicates arithmetic mean and error bars indicate the normal range.

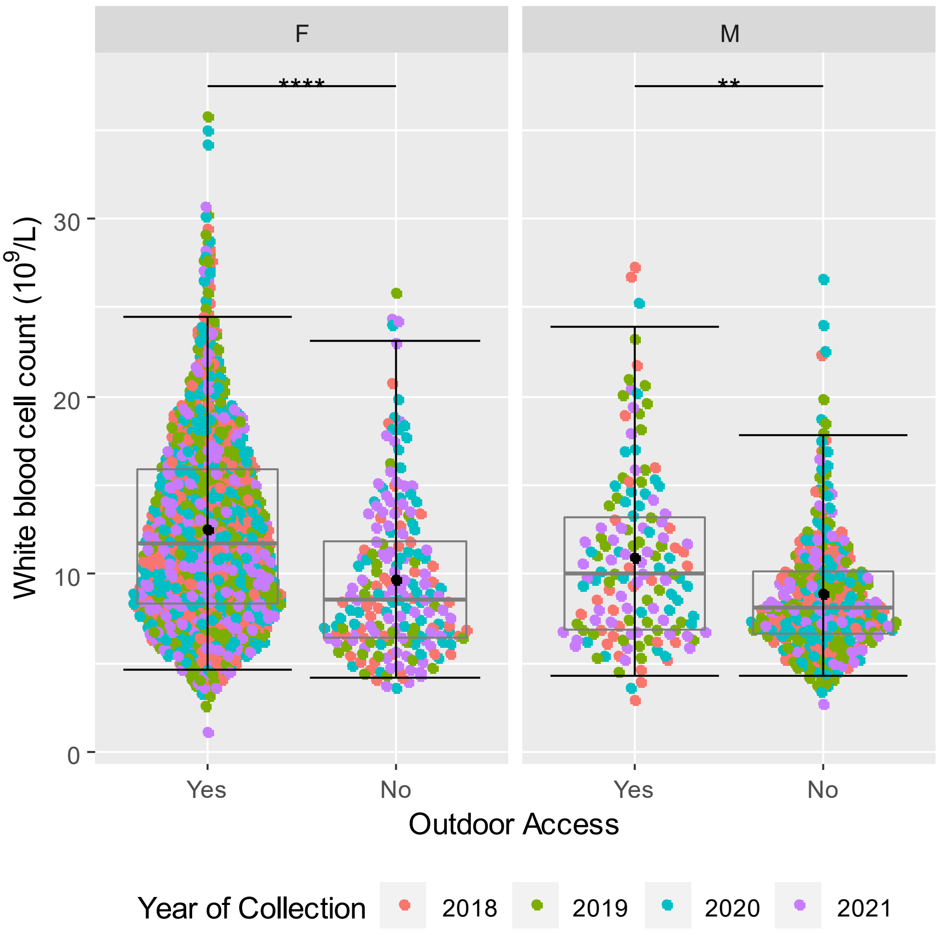

Figure S6 31. White Blood Cell Count according to outdoor access in adult rhesus macaques. Boxes indicate median and quartile ranges, black point indicates arithmetic mean and error bars indicate the normal range.

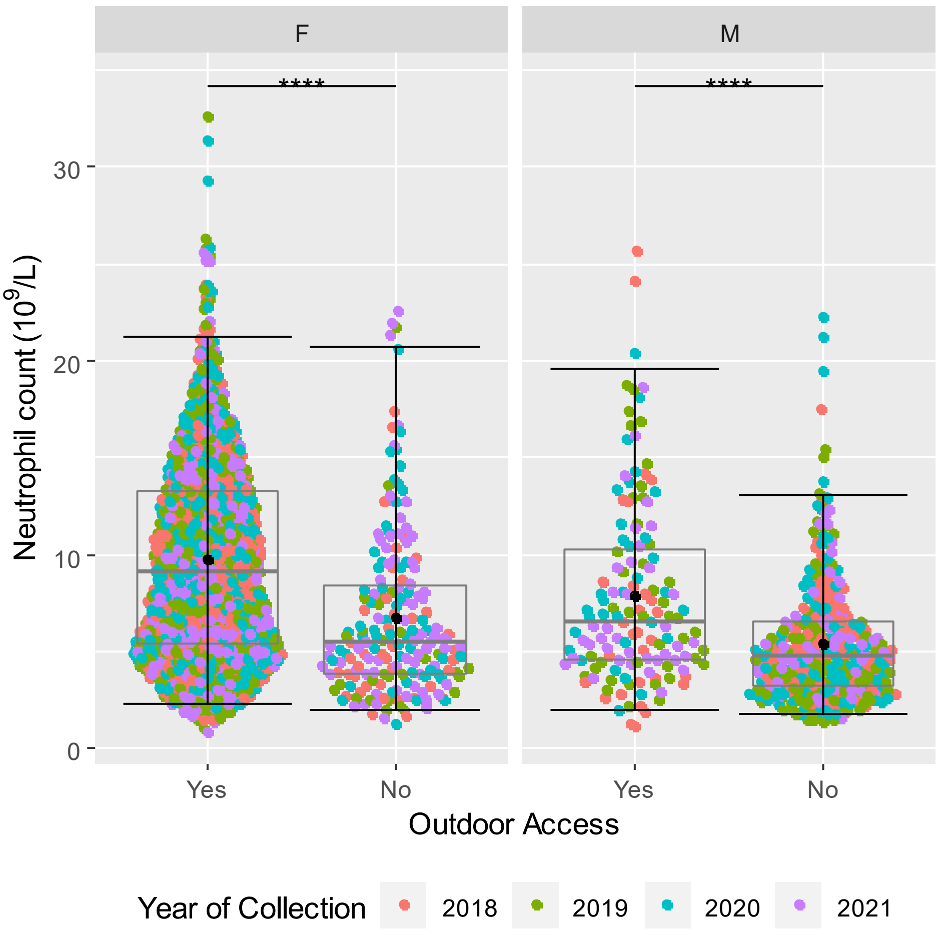

Figure S6 32. Neutrophil Count according to outdoor access in adult rhesus macaques. Boxes indicate median and quartile ranges, black point indicates arithmetic mean and error bars indicate the normal range.

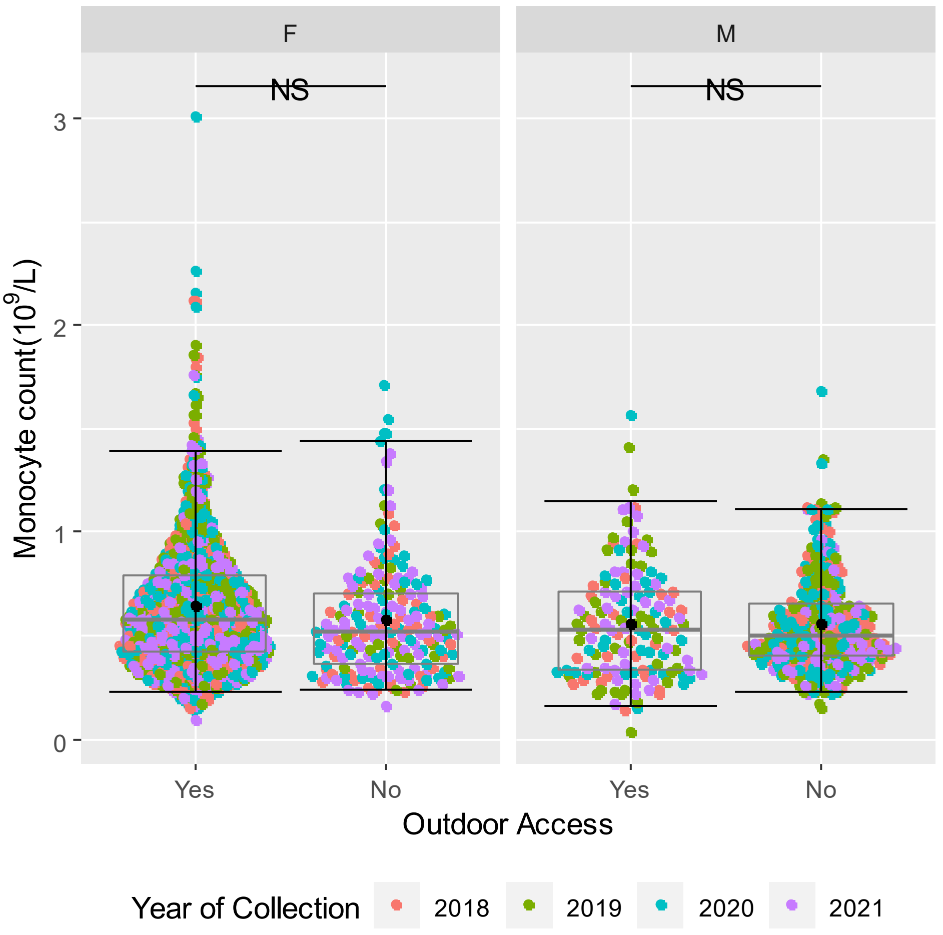

Figure S6 33. Monocyte Count according to outdoor access in adult rhesus macaques. Boxes indicate median and quartile ranges, black point indicates arithmetic mean and error bars indicate the normal range.

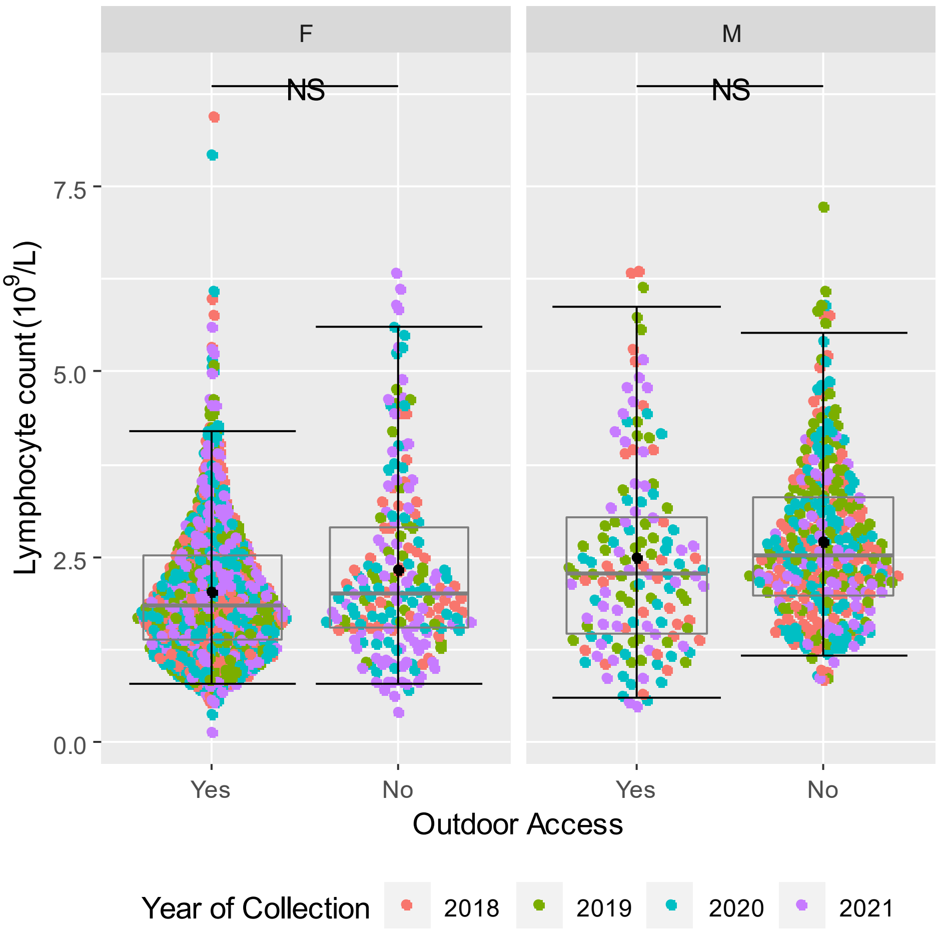

Figure S6 34. Lymphocyte Count according to outdoor access in adult rhesus macaques. Boxes indicate median and quartile ranges, black point indicates arithmetic mean and error bars indicate the normal range.

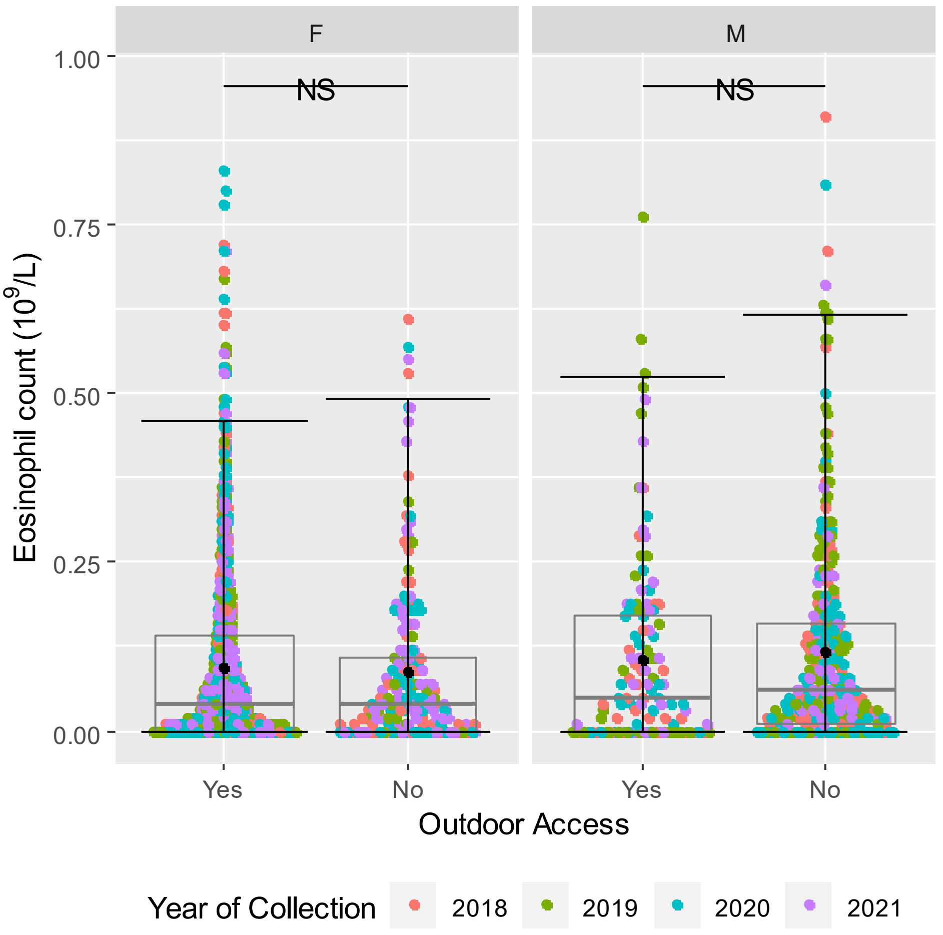

Figure S6 35. Eosinophil Count according to outdoor access in adult rhesus macaques. Boxes indicate median and quartile ranges, black point indicates arithmetic mean and error bars indicate the normal range.

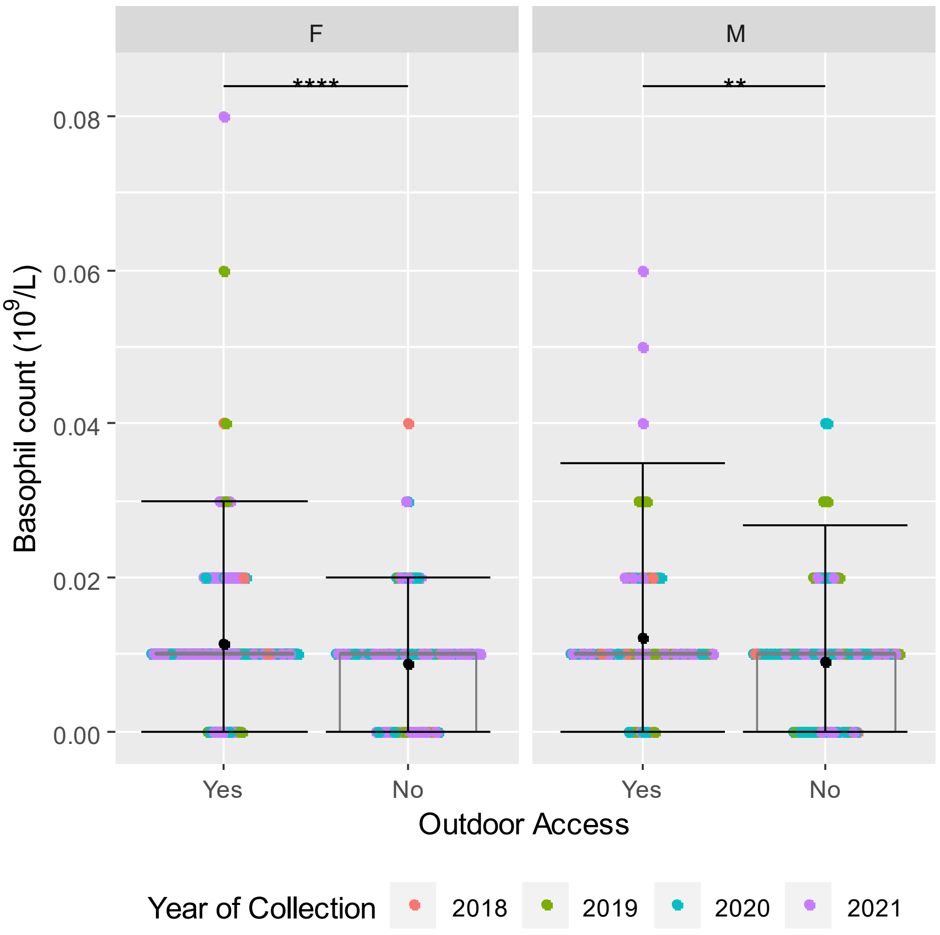

Figure S6 36. Basophil Count according to outdoor access in adult rhesus macaques. Boxes indicate median and quartile ranges, black point indicates arithmetic mean and error bars indicate the normal range.
