# Supplementary material for: Reference Intervals and Percentiles for Hematologic and Serum Biochemical Values in Captive Bred Rhesus (Macaca mulatta) and Cynomolgus Macaques (Macaca fascicularis)
Source: Animals (Basel). 2023 Jan 28;13(3):445. doi: 10.3390/ani13030445 (PMC9913310; doi:10.3390/ani13030445)
Supplement: Supplementary file 1 [file animals-13-00445-s001.zip › Figure S8.pdf]

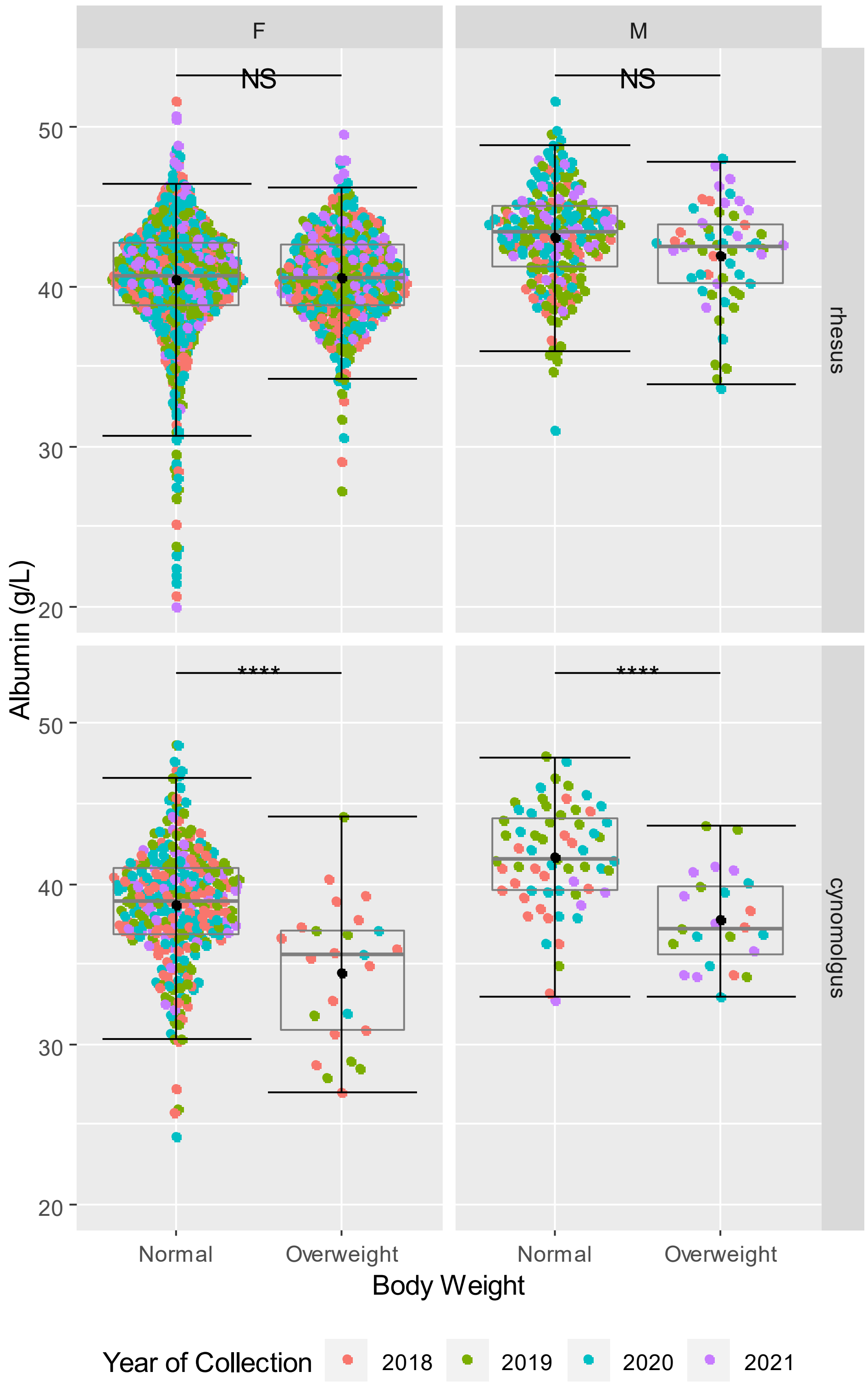

Figure S8 1. Albumin levels according to body weight in adult rhesus and cynomolgus macaques. Boxes indicate median and quartile ranges, black point indicates arithmetic mean and error bars indicate the normal range.

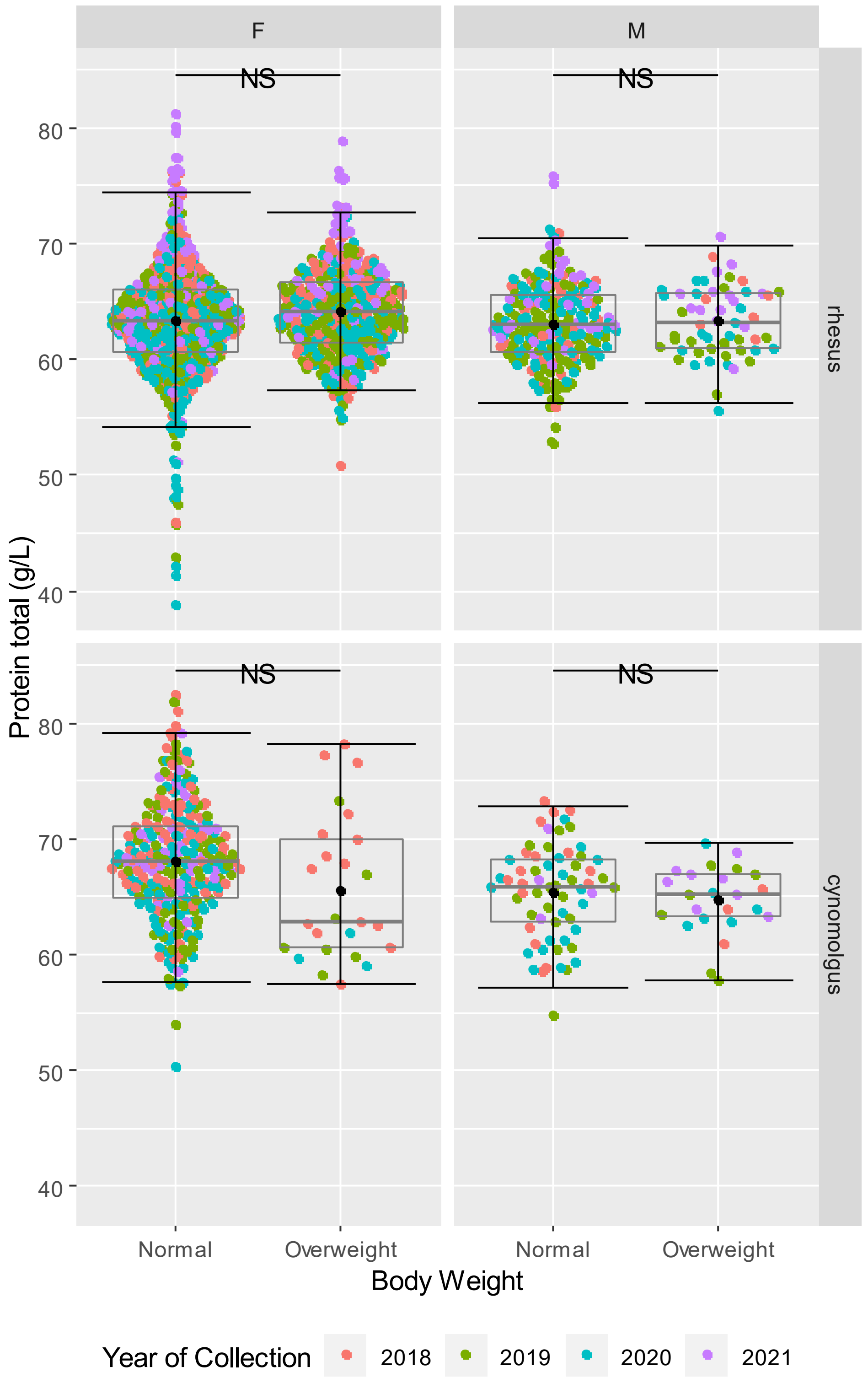

Figure S8 2. Total Protein levels according to body weight in adult rhesus and cynomolgus macaques. Boxes indicate median and quartile ranges, black point indicates arithmetic mean and error bars indicate the normal range.

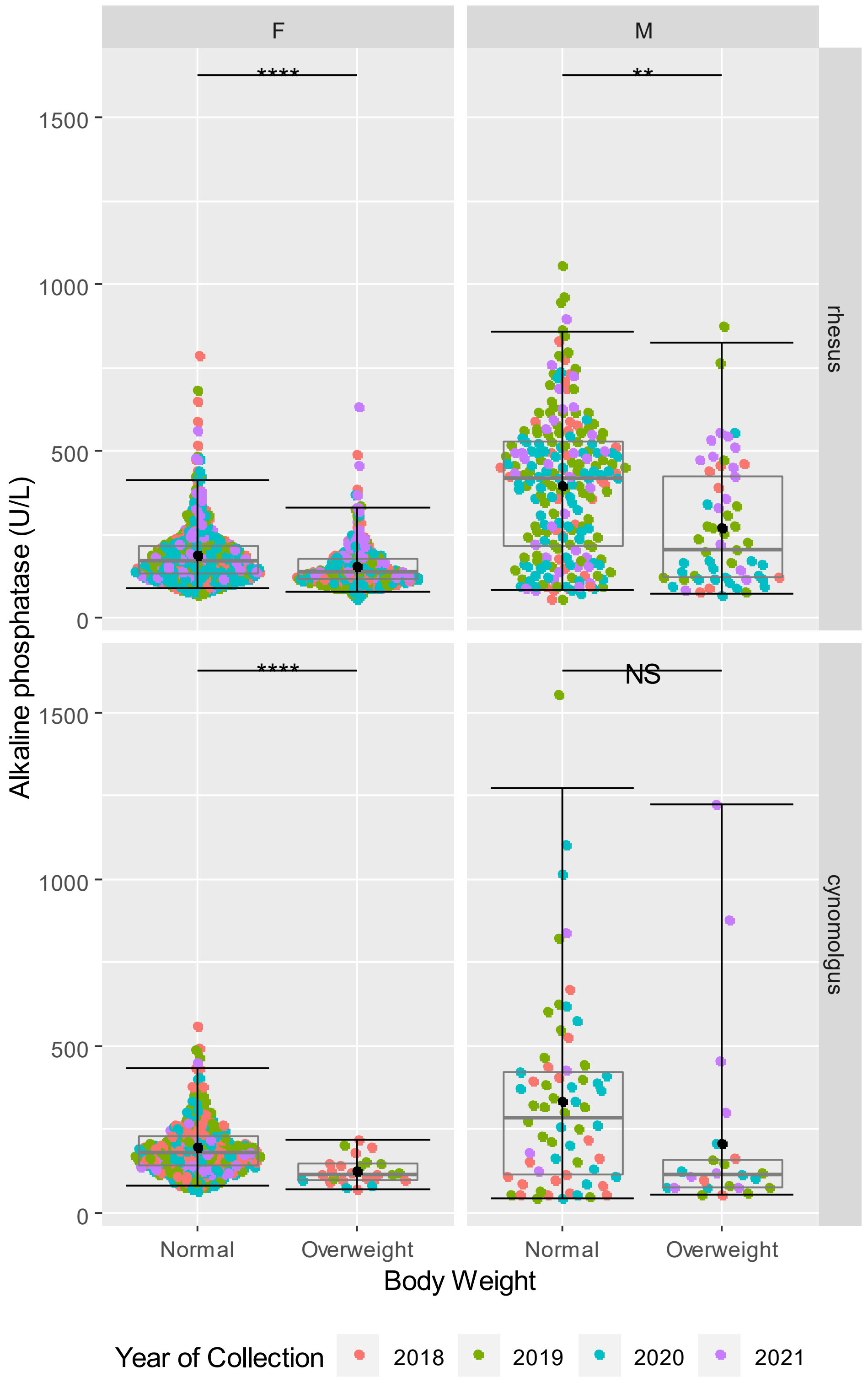

Figure S8 3. Alkaline Phosphatase levels according to period in adult rhesus and cynomolgus macaques. Boxes indicate median and quartile ranges, black point indicates arithmetic mean and error bars indicate the normal range.

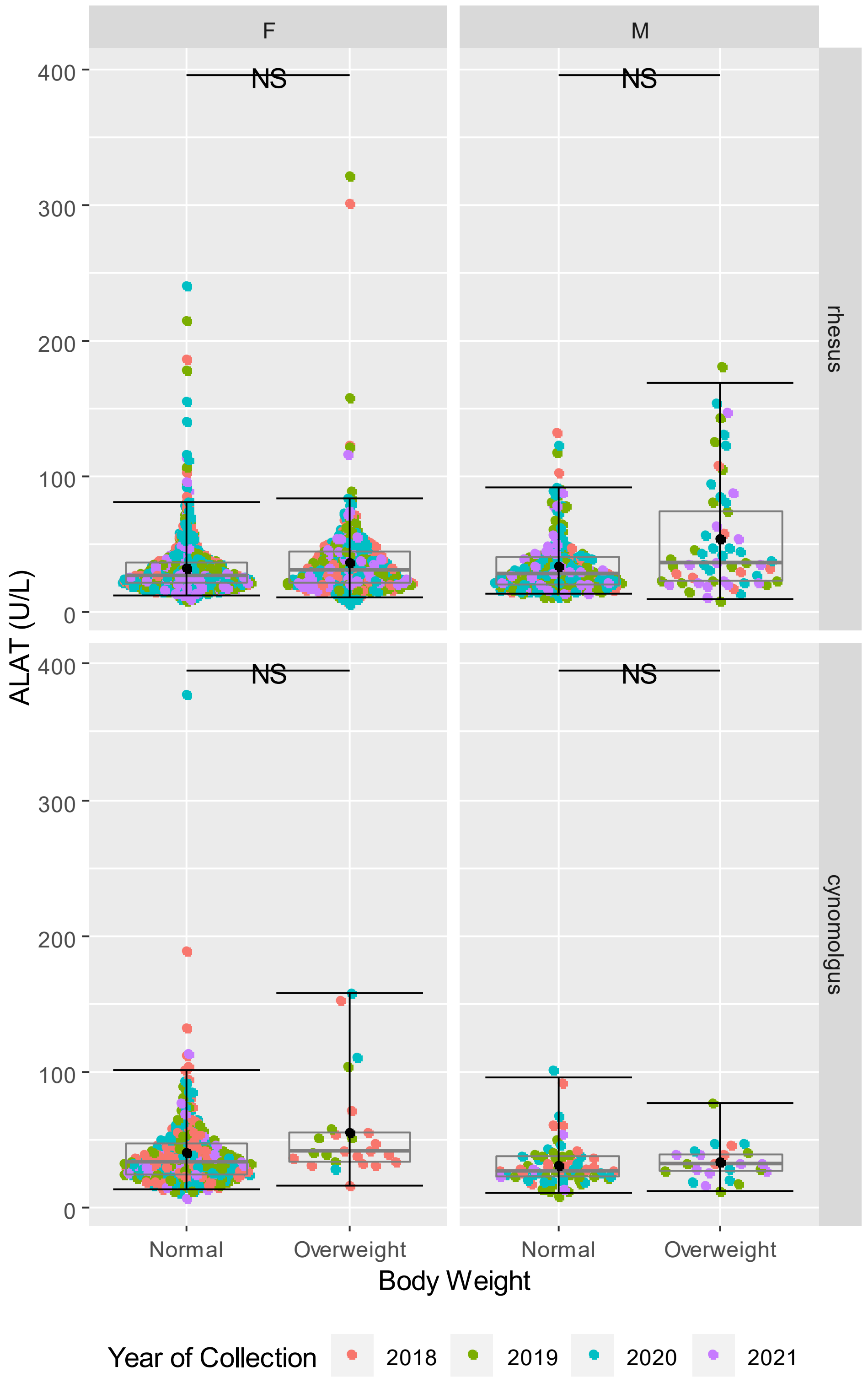

Figure S8 4. ALAT levels according to body weight in adult rhesus and cynomolgus macaques. Boxes indicate median and quartile ranges, black point indicates arithmetic mean and error bars indicate the normal range.

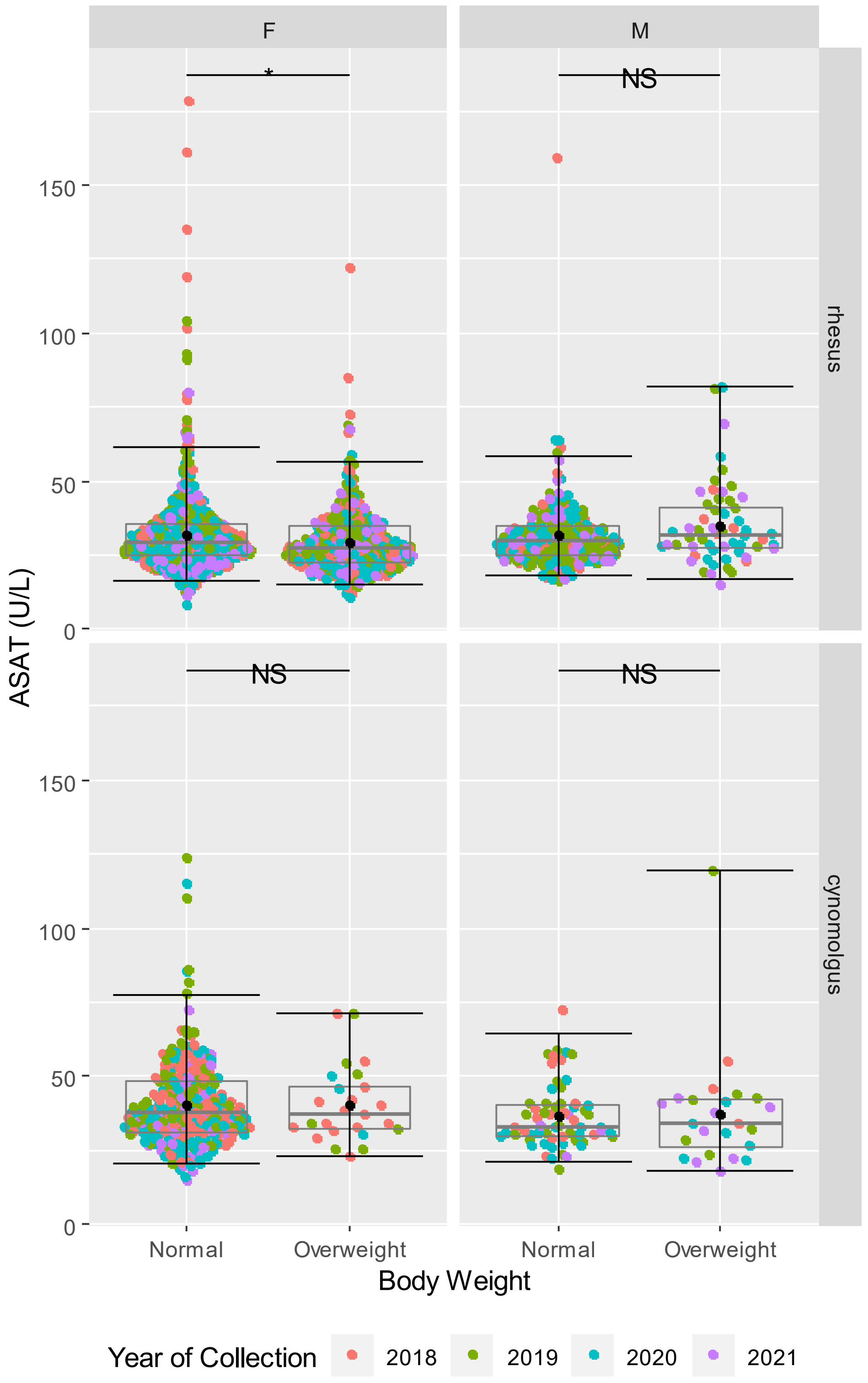

Figure S8 5. ASAT levels according to body weight in adult rhesus and cynomolgus macaques. Boxes indicate median and quartile ranges, black point indicates arithmetic mean and error bars indicate the normal range.

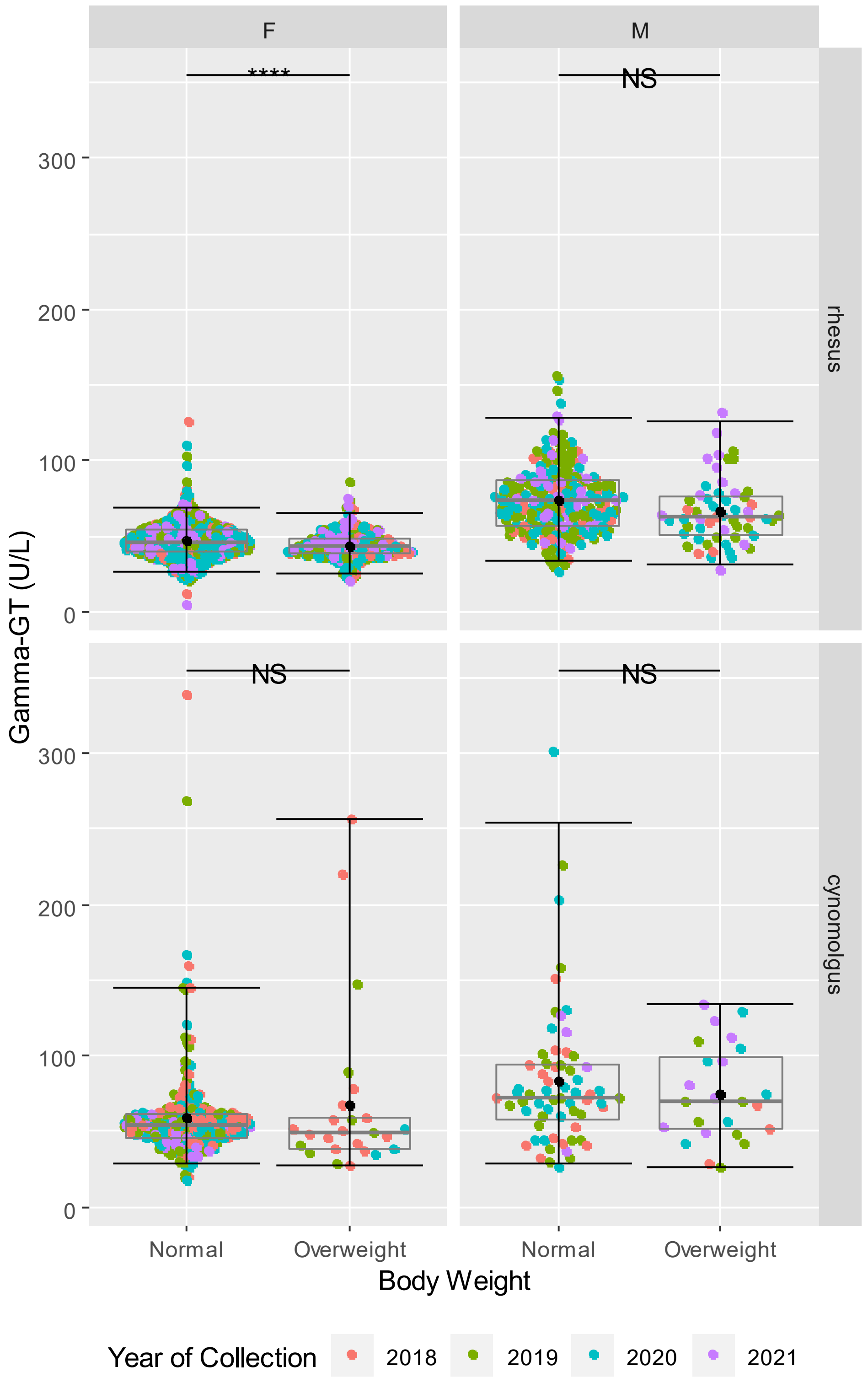

Figure S8 6. Gamma-GT levels according to body weight in adult rhesus and cynomolgus macaques. Boxes indicate median and quartile ranges, black point indicates arithmetic mean and error bars indicate the normal range.

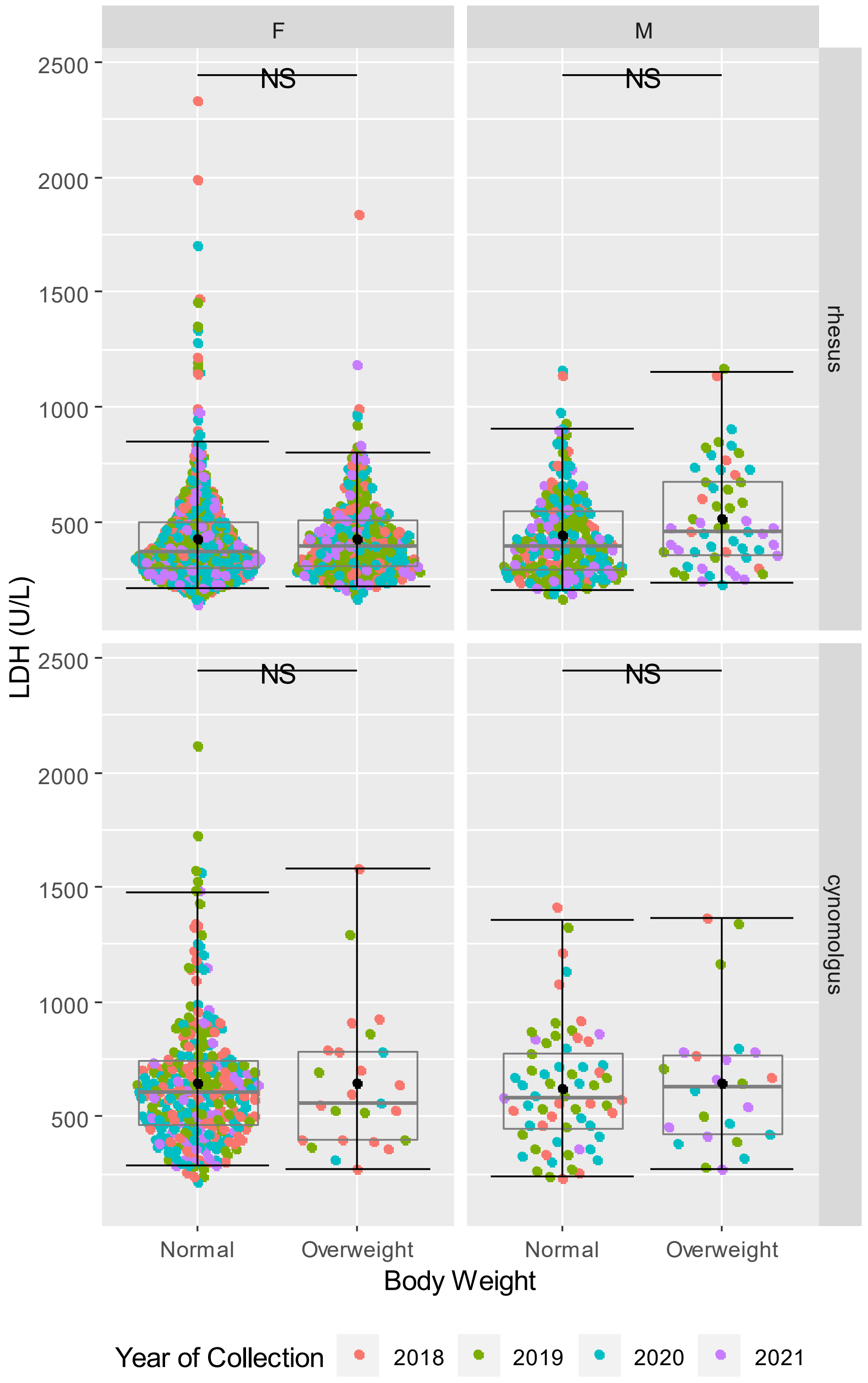

Figure S8 7. LDH levels according to body weight in adult rhesus and cynomolgus macaques. Boxes indicate median and quartile ranges, black point indicates arithmetic mean and error bars indicate the normal range.

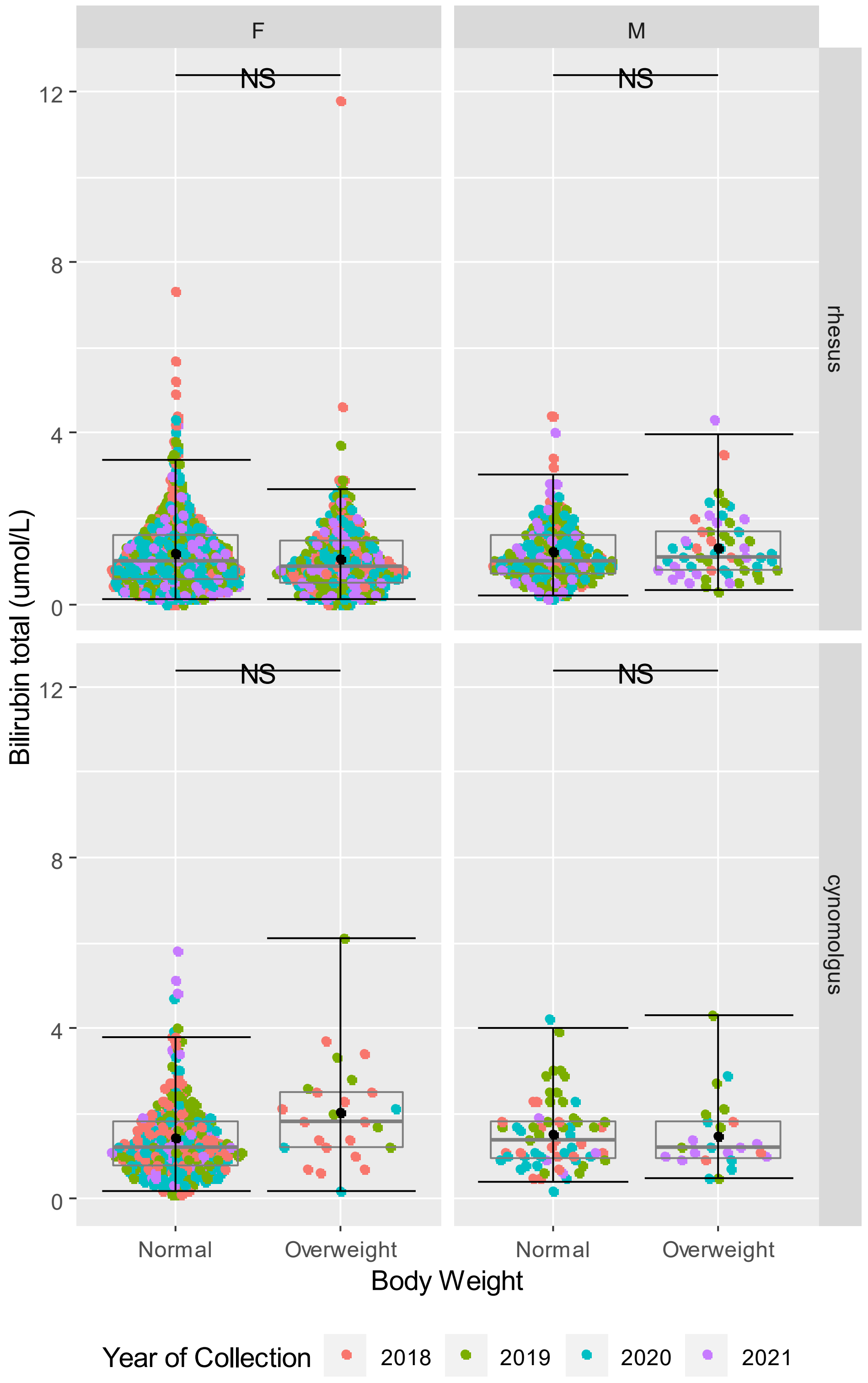

Figure S8 8. Total Bilirubin levels according to body weight in adult rhesus and cynomolgus macaques. Boxes indicate median and quartile ranges, black point indicates arithmetic mean and error bars indicate the normal range.

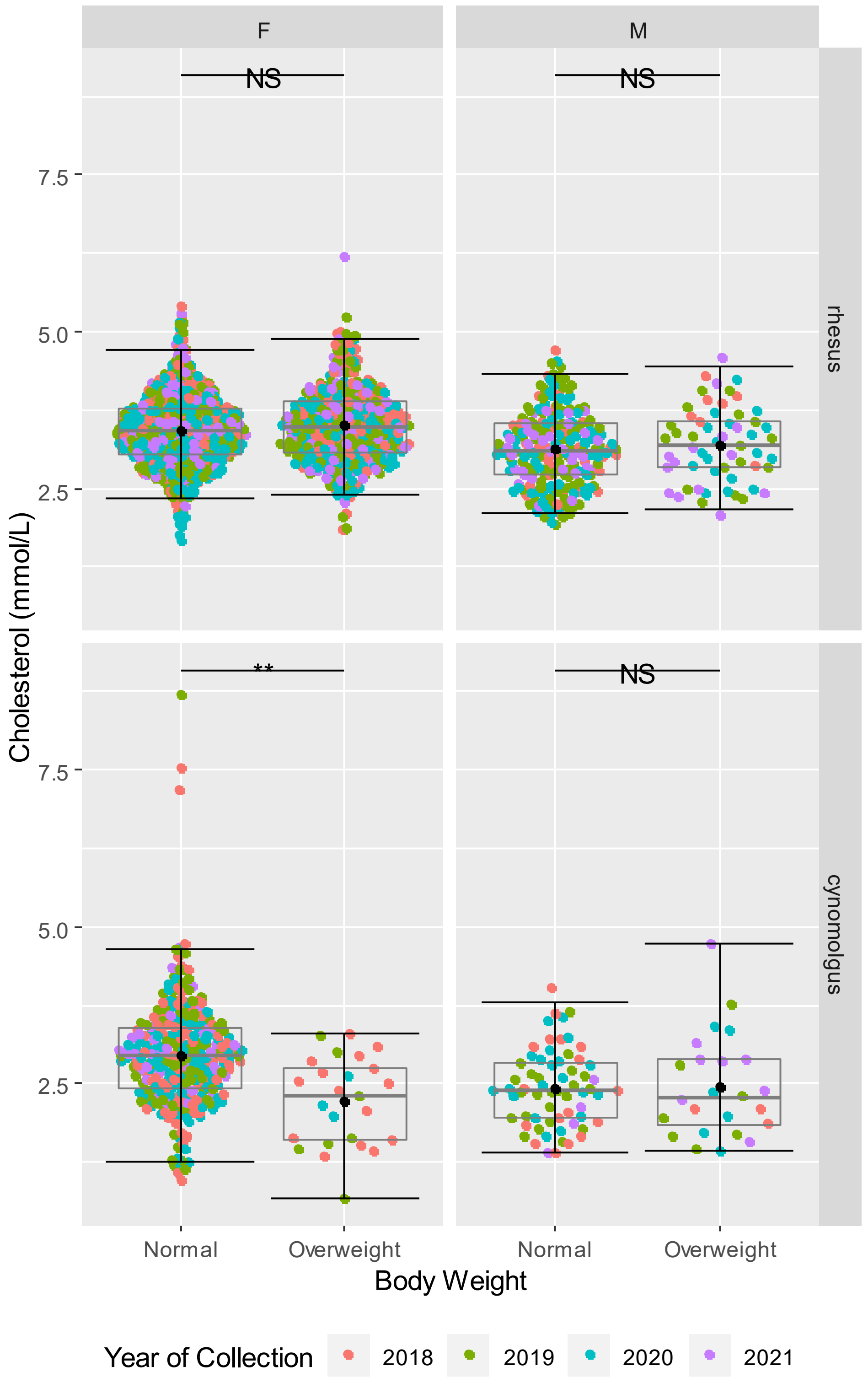

Figure S8 9. Cholesterol levels according to body weight in adult rhesus and cynomolgus macaques. Boxes indicate median and quartile ranges, black point indicates arithmetic mean and error bars indicate the normal range.

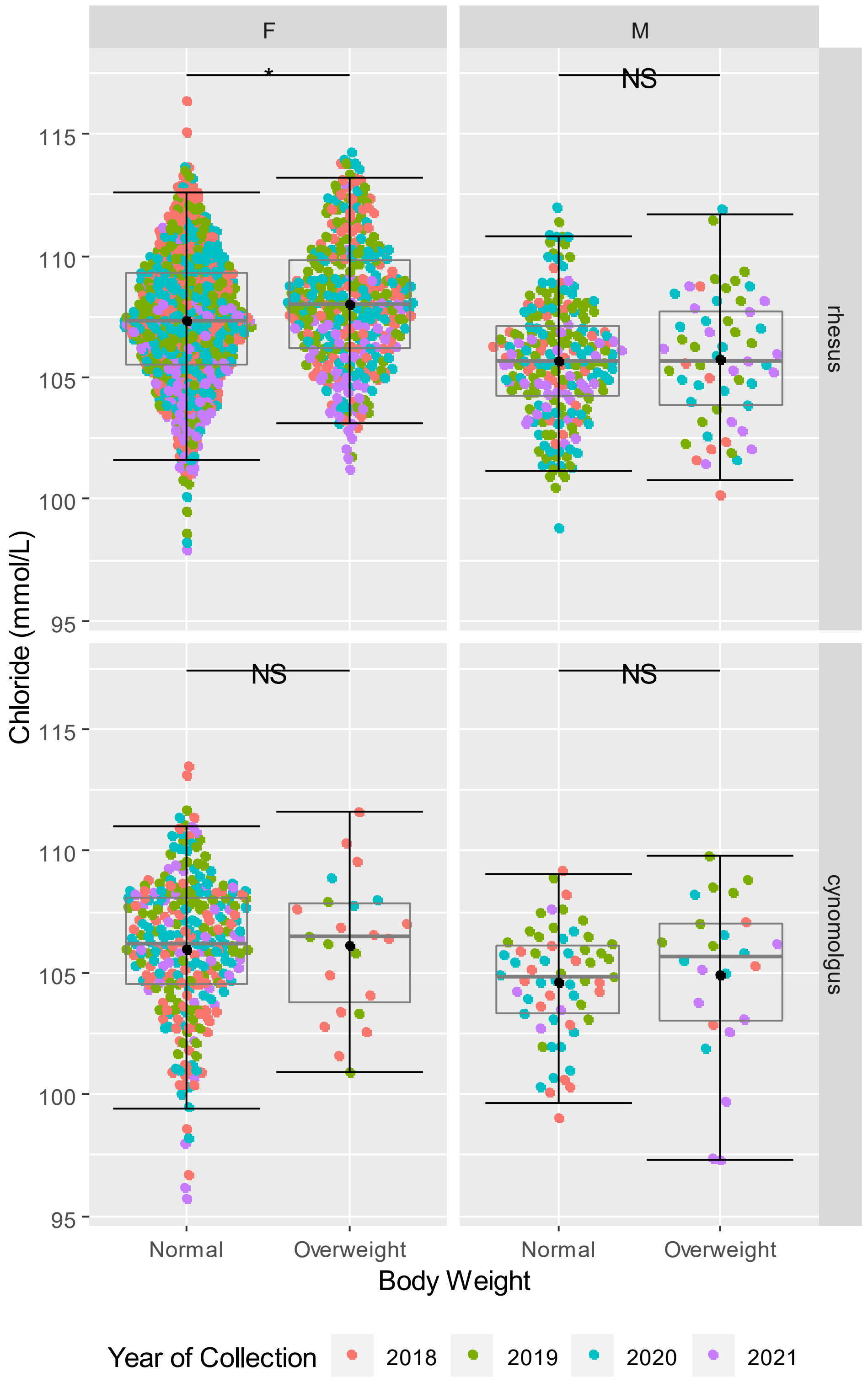

Figure S8 10. Chloride levels according to body weight in adult rhesus and cynomolgus macaques. Boxes indicate median and quartile ranges, black point indicates arithmetic mean and error bars indicate the normal range.

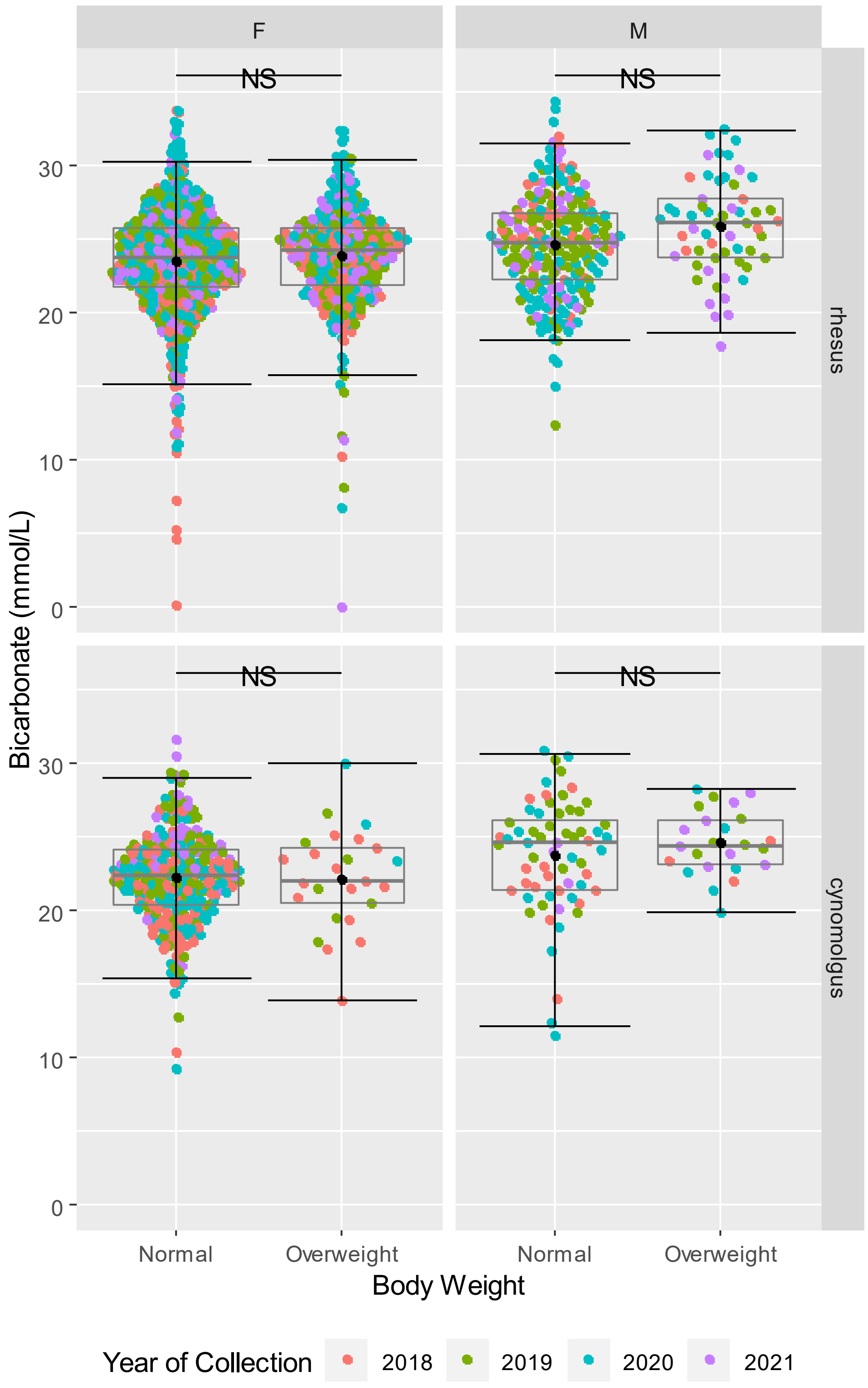

Figure S8 11. Bicarbonate levels according to body weight in adult rhesus and cynomolgus macaques. Boxes indicate median and quartile ranges, black point indicates arithmetic mean and error bars indicate the normal range.

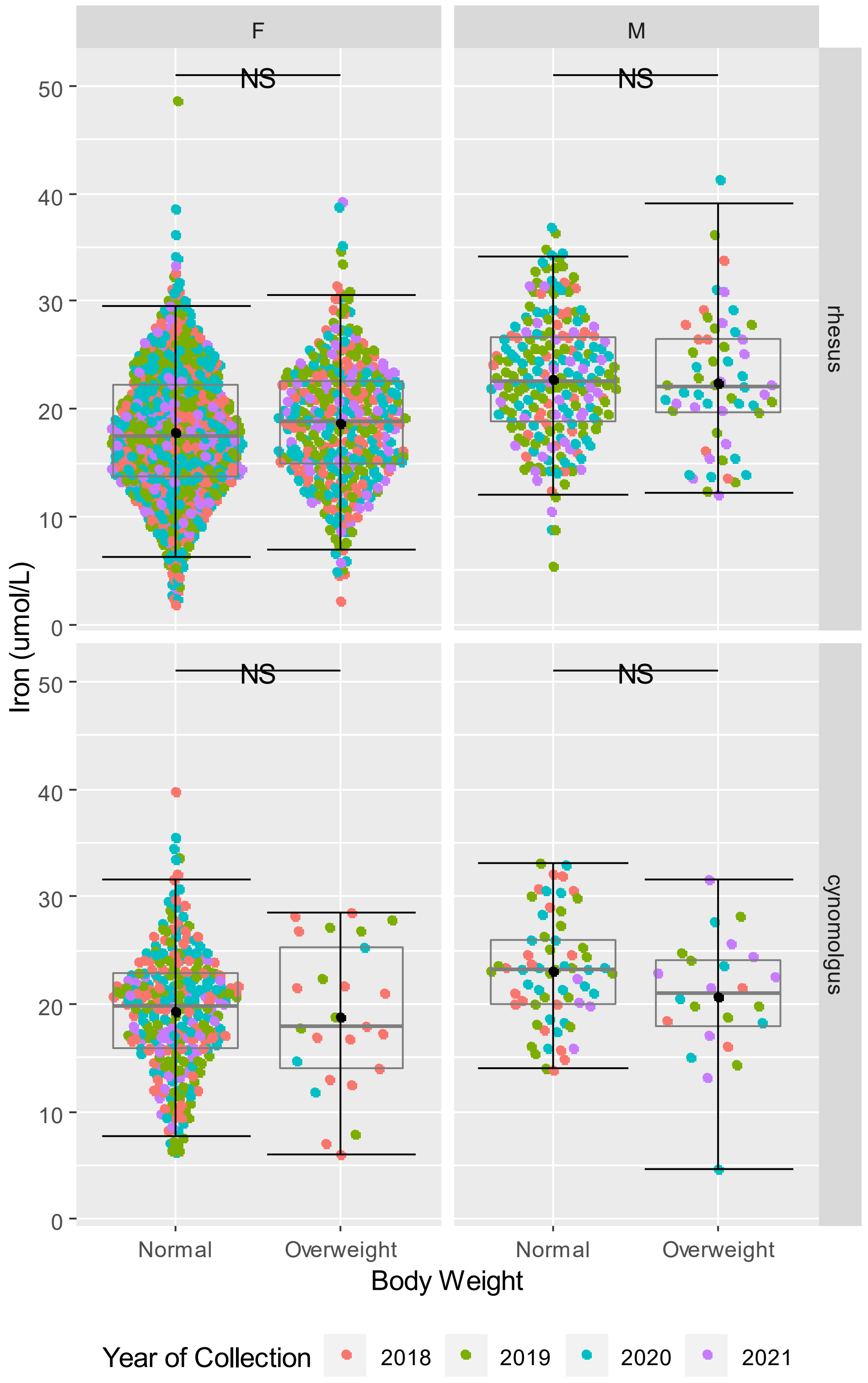

Figure S8 12. Iron levels according to body weight in adult rhesus and cynomolgus macaques. Boxes indicate median and quartile ranges, black point indicates arithmetic mean and error bars indicate the normal range.

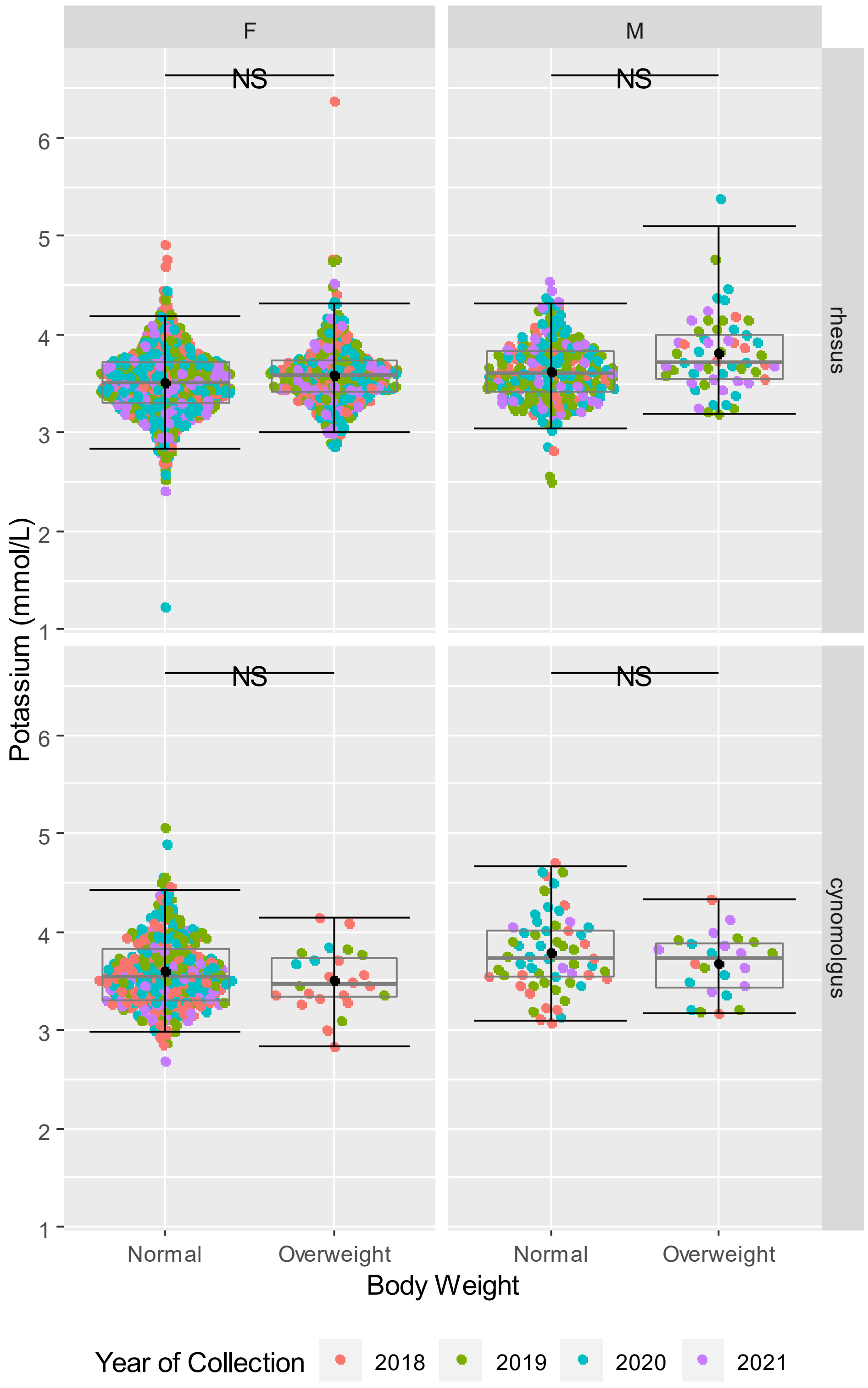

Figure S8 13. Potassium levels according to body weight in adult rhesus and cynomolgus macaques. Boxes indicate median and quartile ranges, black point indicates arithmetic mean and error bars indicate the normal range.

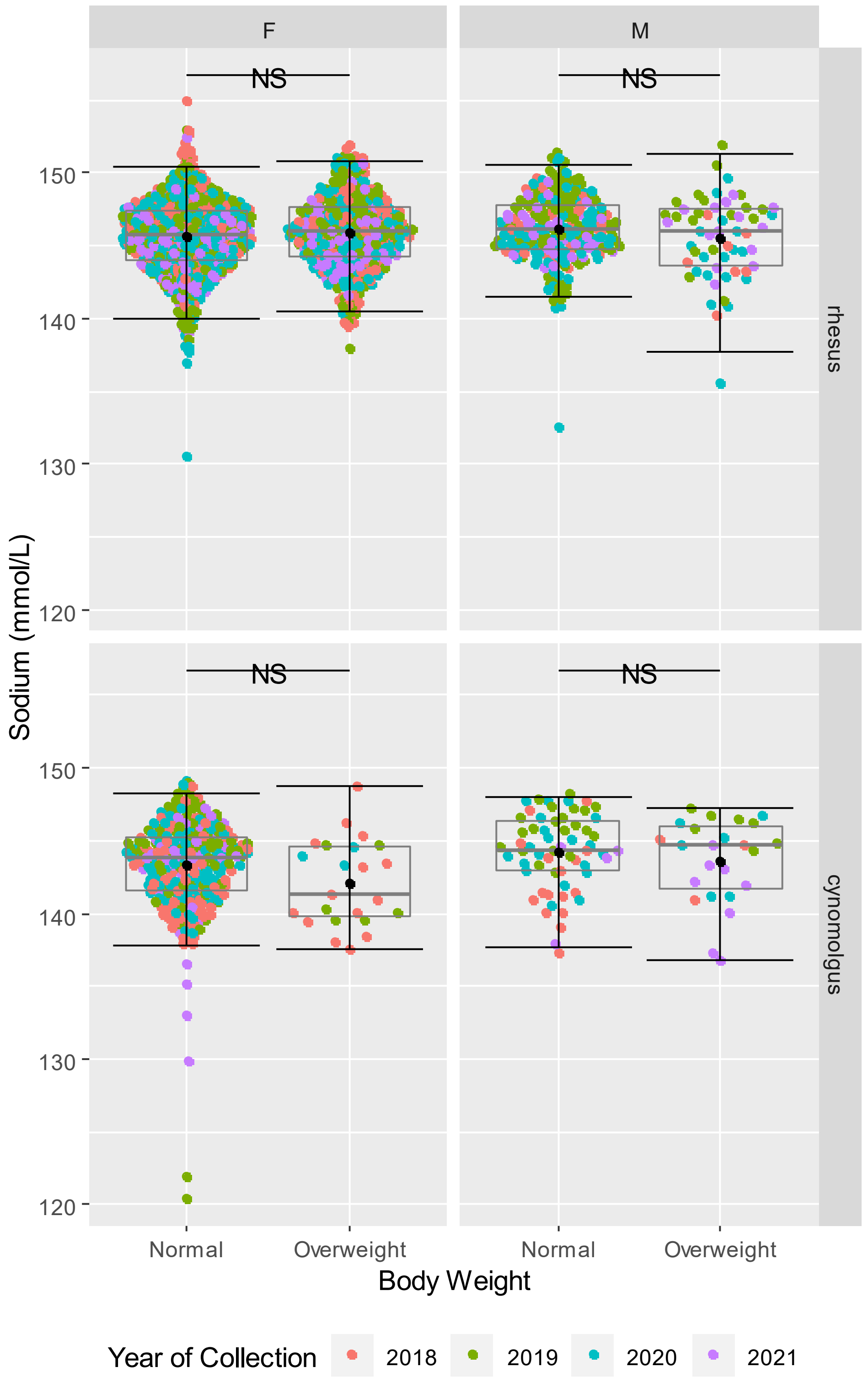

Figure S8 14. Sodium levels according to body weight in adult rhesus and cynomolgus macaques. Boxes indicate median and quartile ranges, black point indicates arithmetic mean and error bars indicate the normal range.

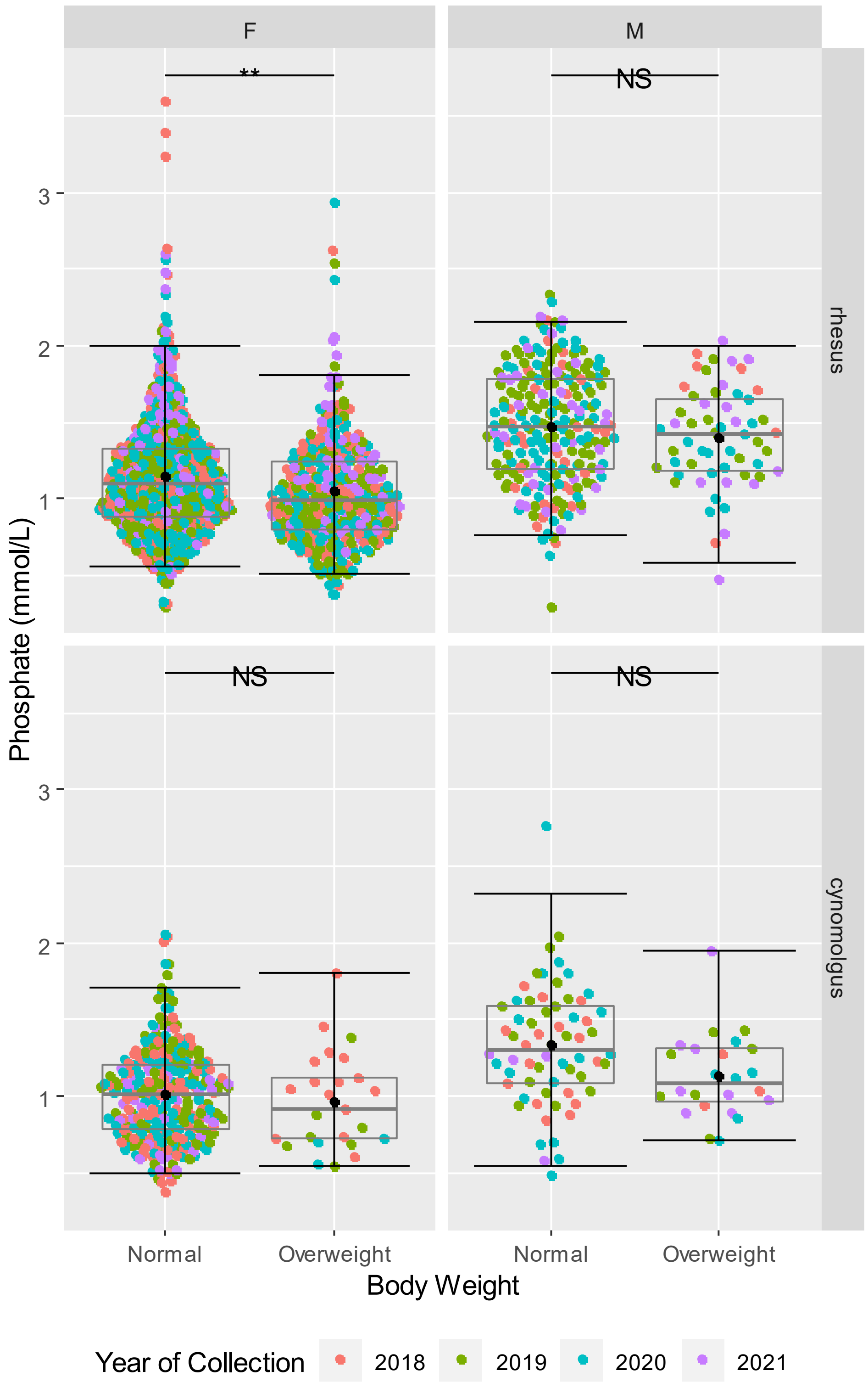

Figure S8 15. Phosphate levels according to body weight in adult rhesus and cynomolgus macaques. Boxes indicate median and quartile ranges, black point indicates arithmetic mean and error bars indicate the normal range.

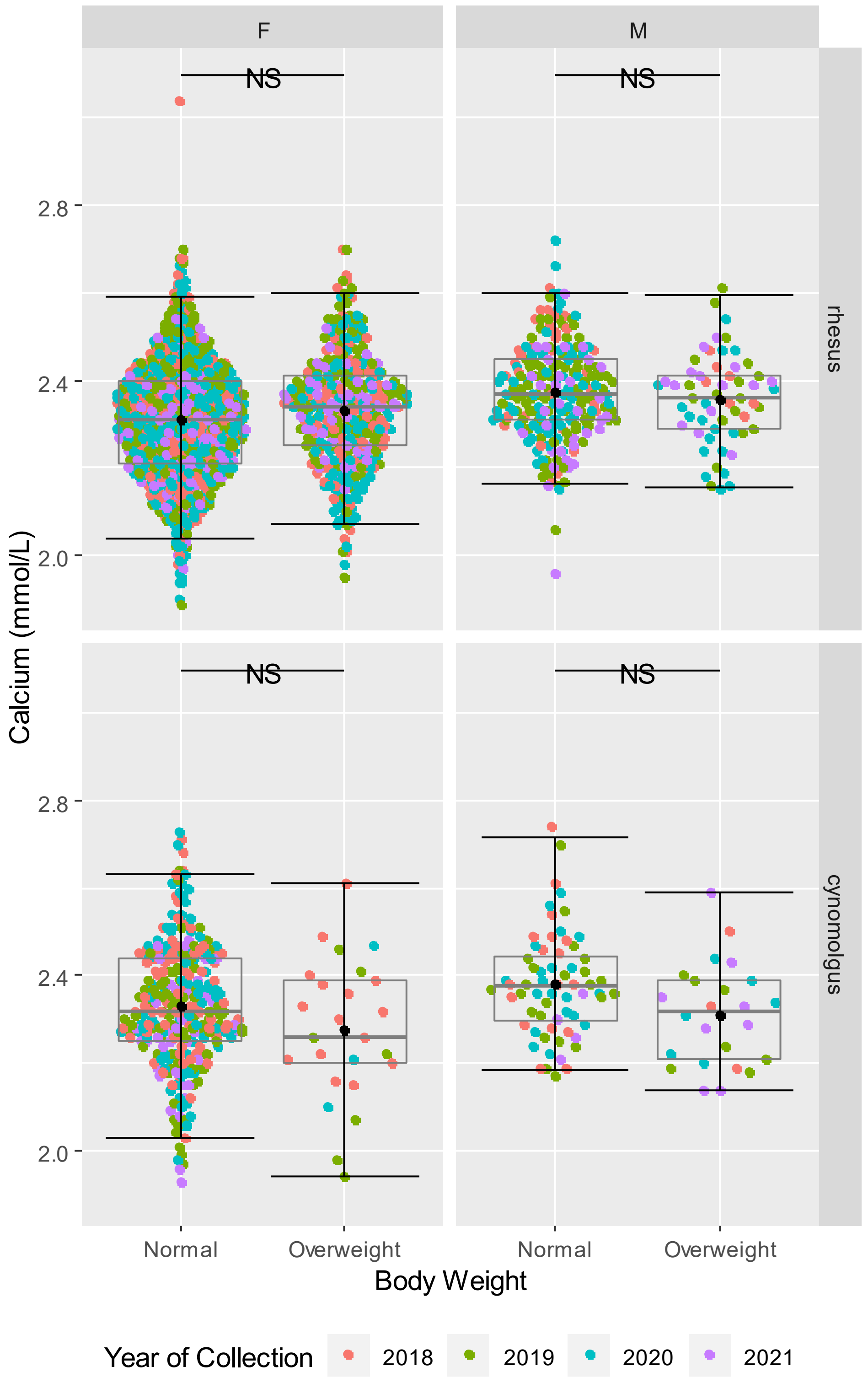

Figure S8 16. Calcium levels according to body weight in adult rhesus and cynomolgus macaques. Boxes indicate median and quartile ranges, black point indicates arithmetic mean and error bars indicate the normal range.

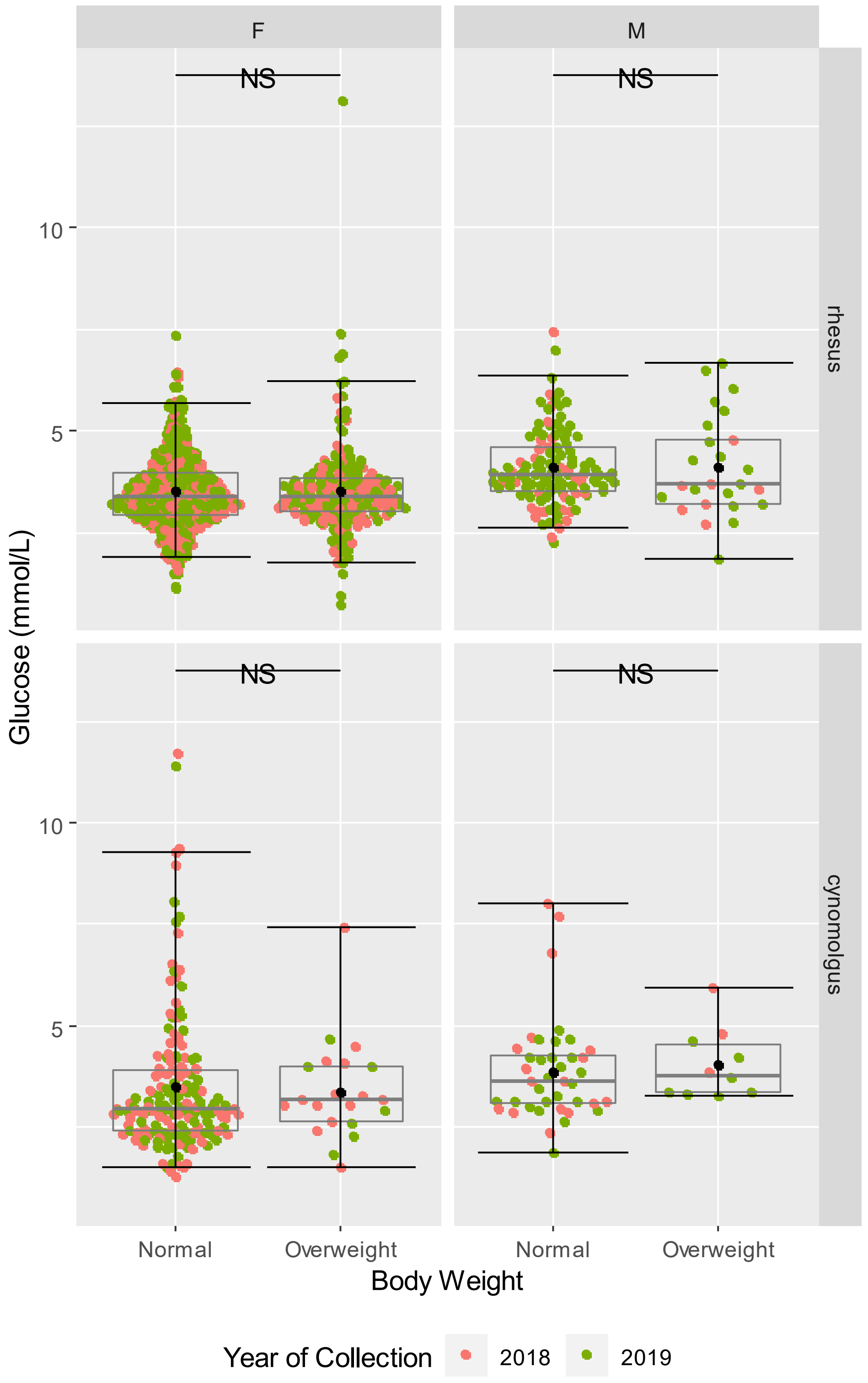

Figure S8 17A. Glucose levels in 2018-2019 according to body weight in adult rhesus and cynomolgus macaques. Boxes indicate median and quartile ranges, black point indicates arithmetic mean and error bars indicate the normal range.

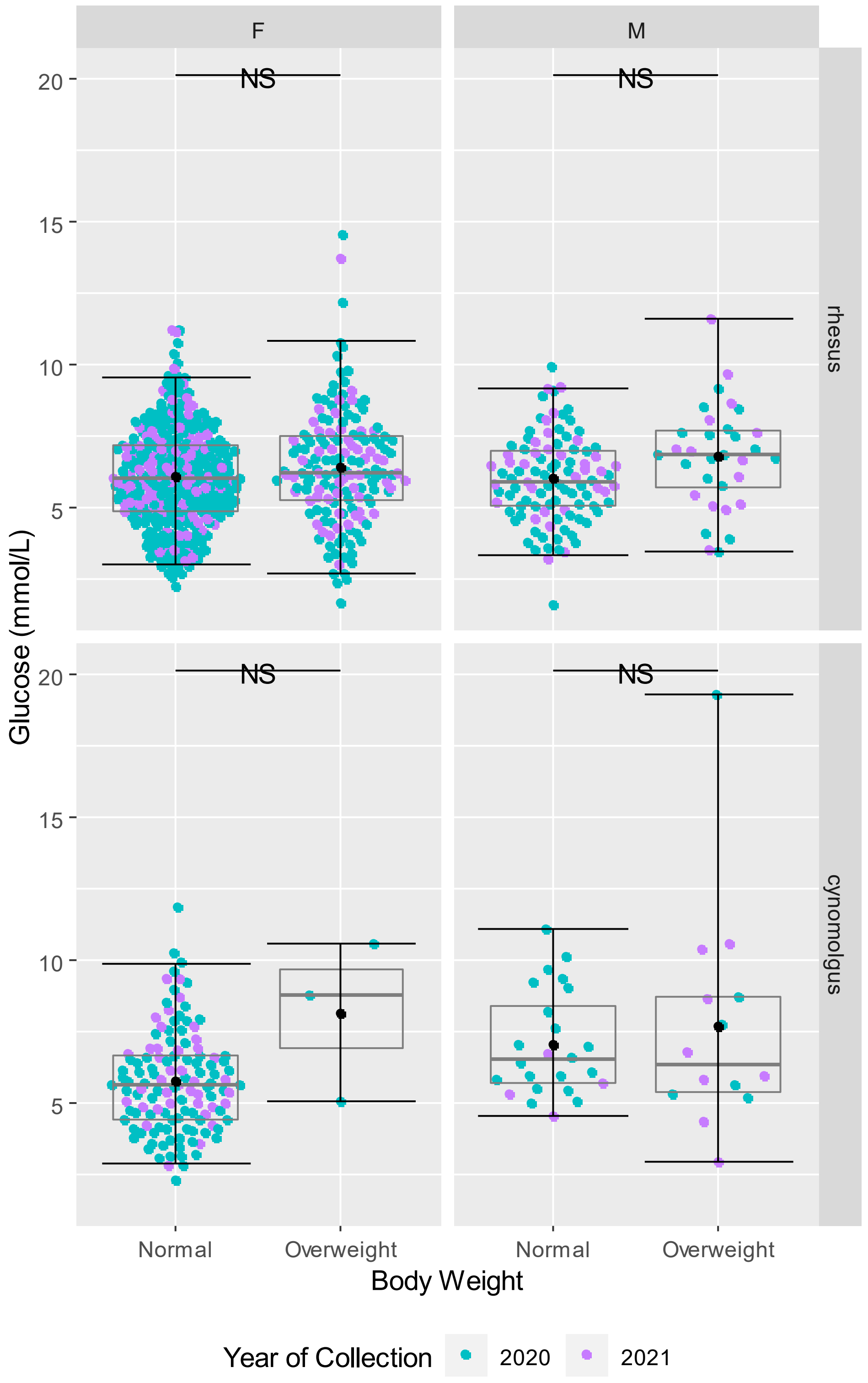

Figure S8 17B. Glucose levels in 2020-2021 according to body weight in adult rhesus and cynomolgus macaques. Boxes indicate median and quartile ranges, black point indicates arithmetic mean and error bars indicate the normal range.

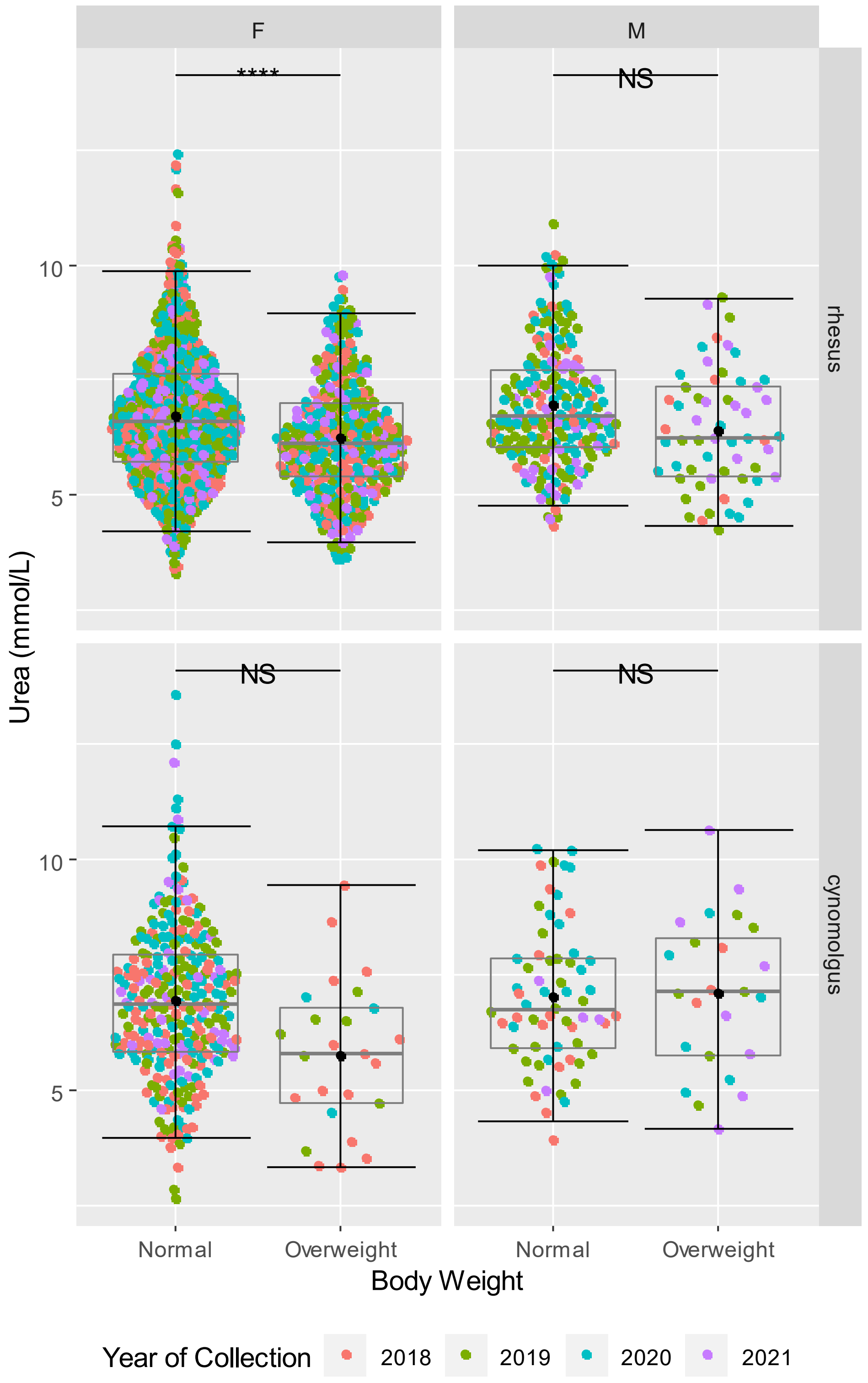

Figure S8 18. Urea levels according to body weight in adult rhesus and cynomolgus macaques. Boxes indicate median and quartile ranges, black point indicates arithmetic mean and error bars indicate the normal range.

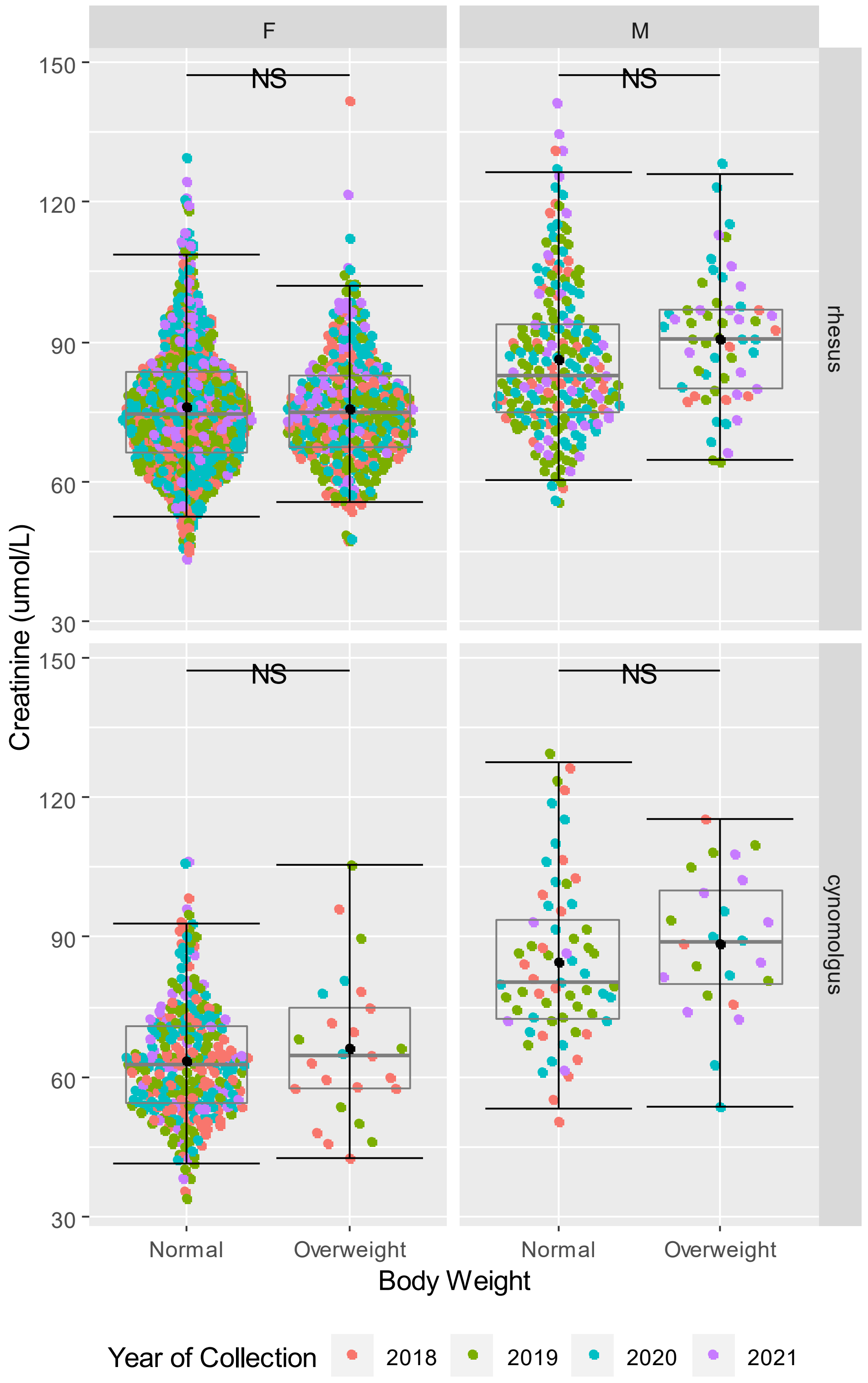

Figure S8 19. Creatinine levels according to body weight in adult rhesus and cynomolgus macaques. Boxes indicate median and quartile ranges, black point indicates arithmetic mean and error bars indicate the normal range.

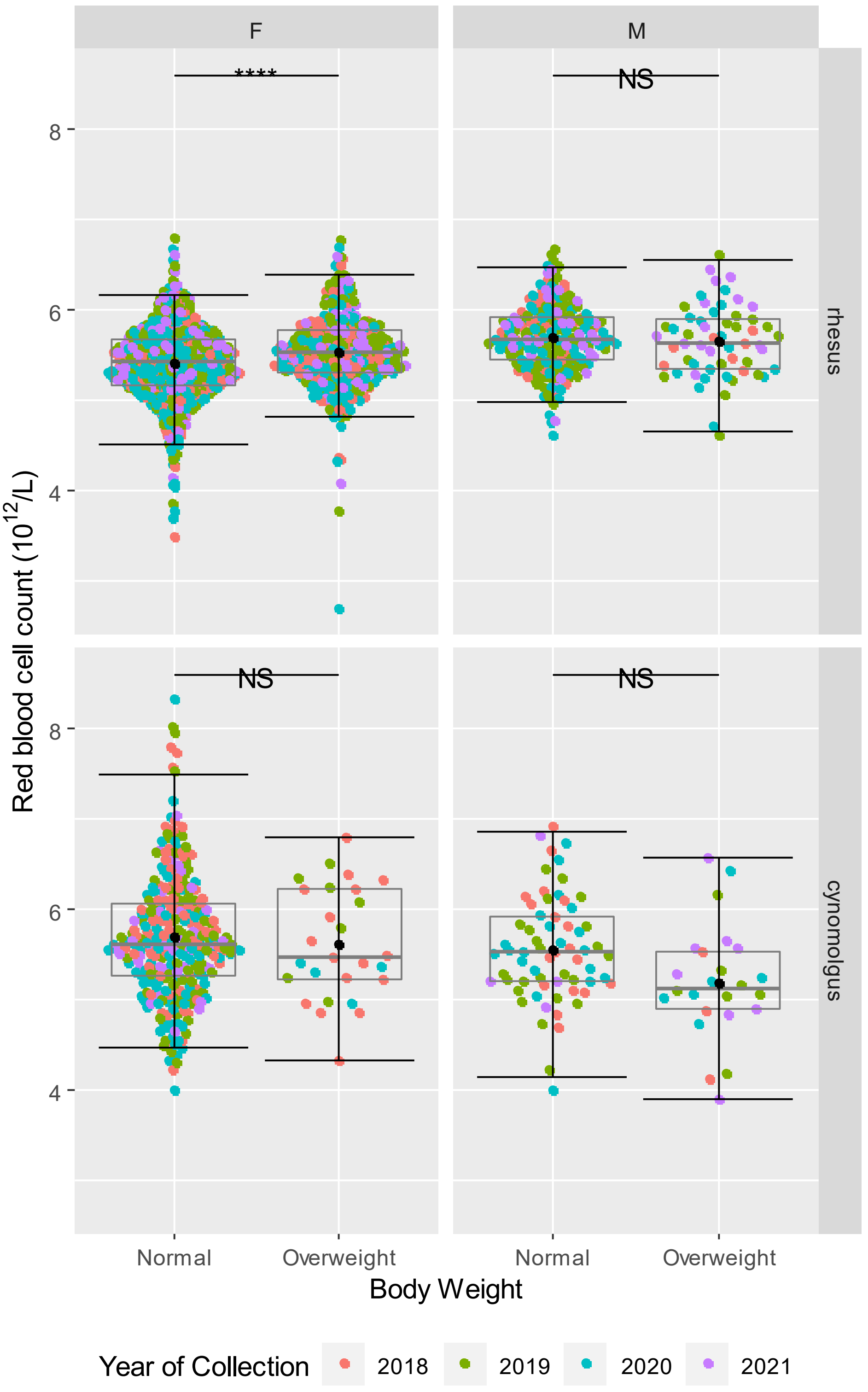

Figure S8 20. Red Blood Cell count according to body weight in adult rhesus and cynomolgus macaques. Boxes indicate median and quartile ranges, black point indicates arithmetic mean and error bars indicate the normal range.

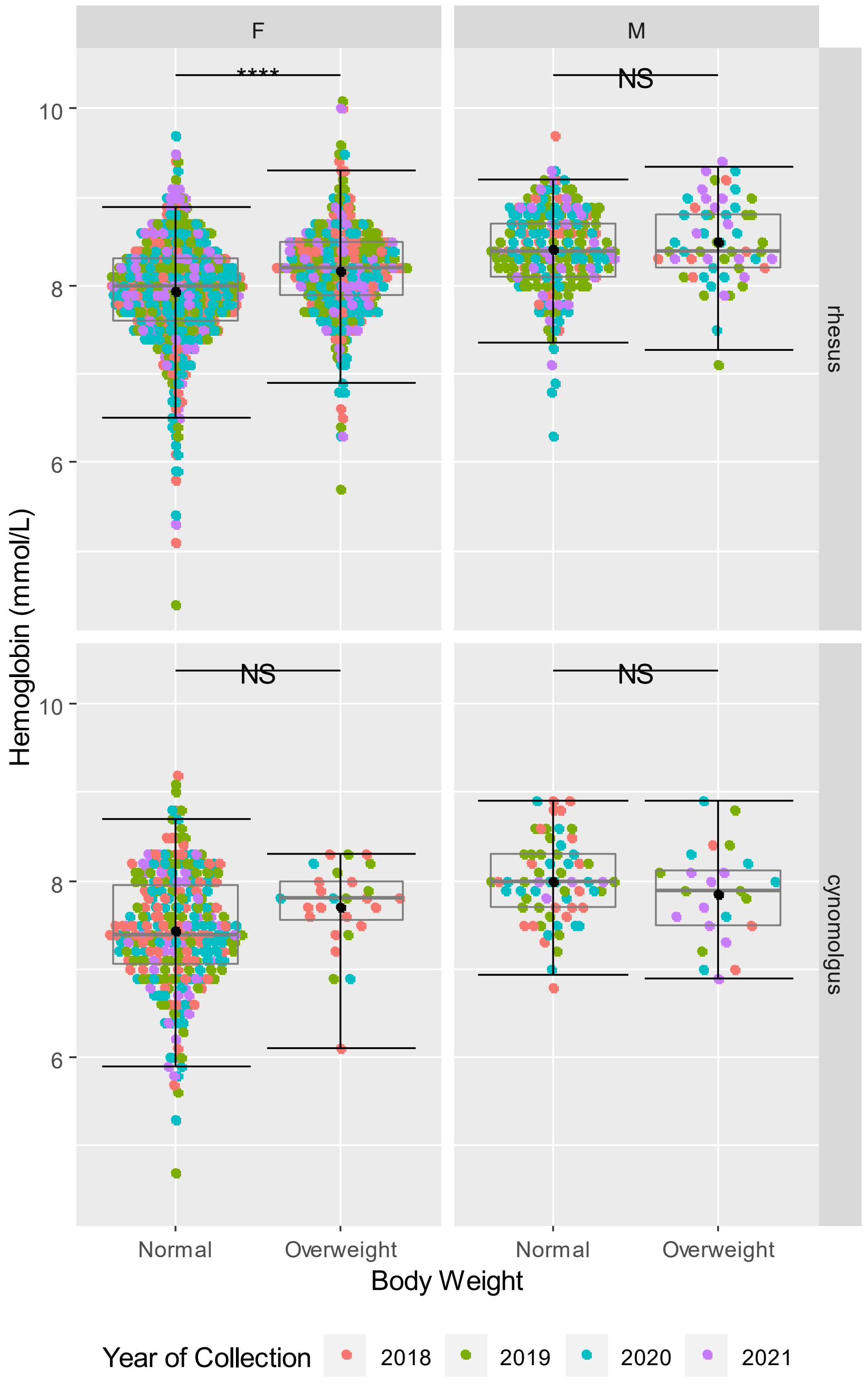

Figure S8 21. Hemoglobin levels according to body weight in adult rhesus and cynomolgus macaques. Boxes indicate median and quartile ranges, black point indicates arithmetic mean and error bars indicate the normal range.

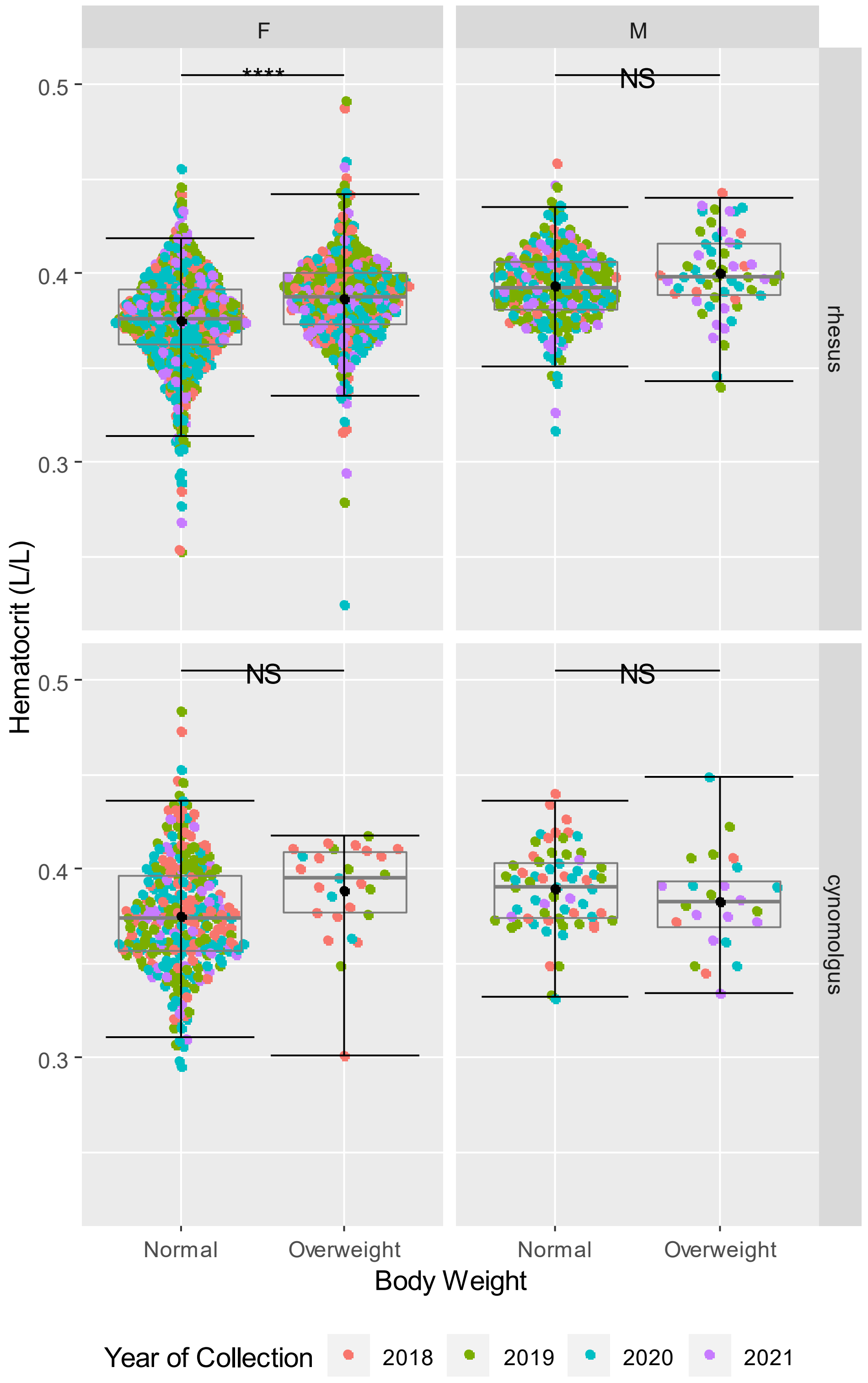

Figure S8 22. Hematocrit levels according to body weight in adult rhesus and cynomolgus macaques. Boxes indicate median and quartile ranges, black point indicates arithmetic mean and error bars indicate the normal range.

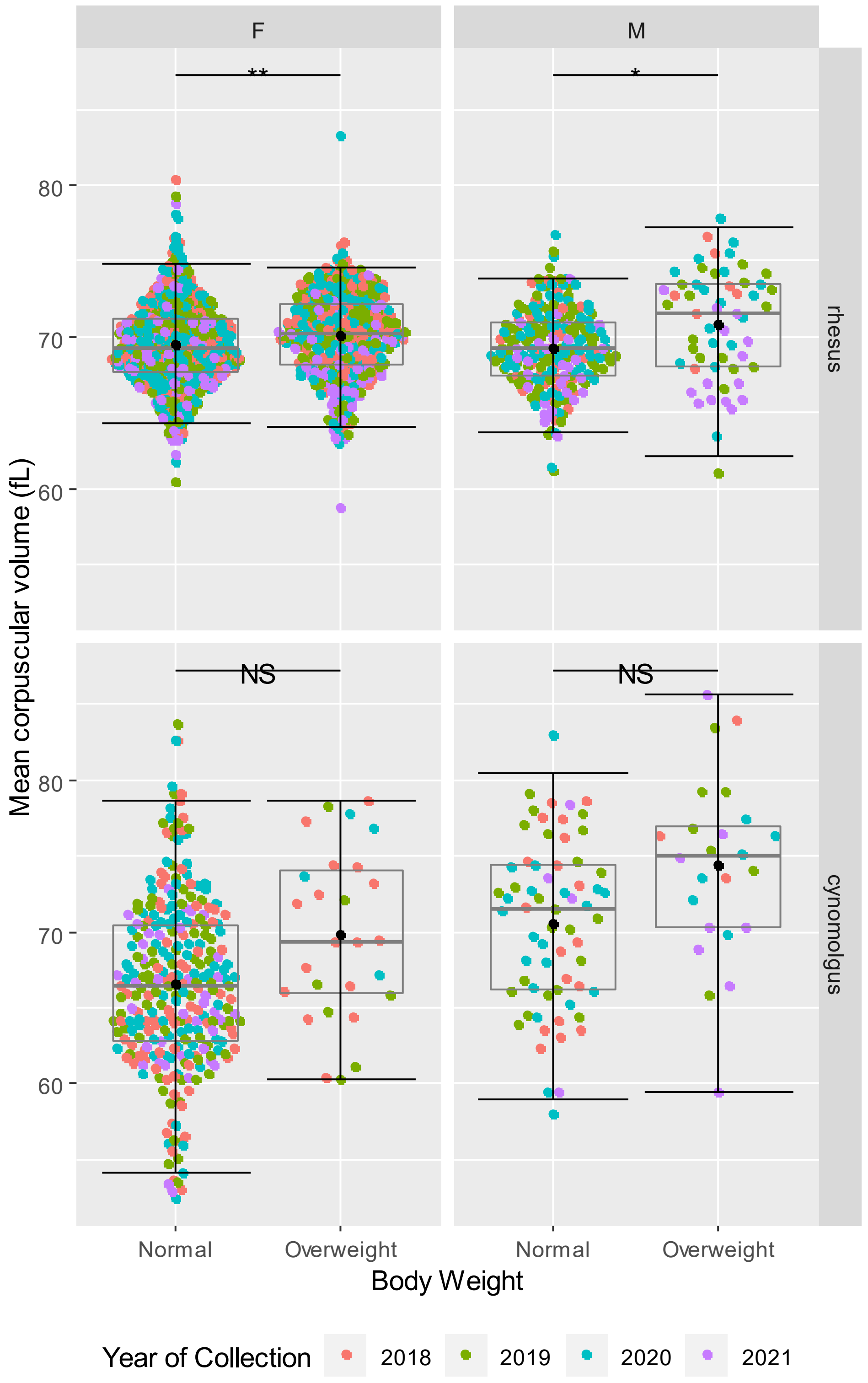

Figure S8 23. Mean Corpuscular Volume according to body weight in adult rhesus and cynomolgus macaques. Boxes indicate median and quartile ranges, black point indicates arithmetic mean and error bars indicate the normal range.

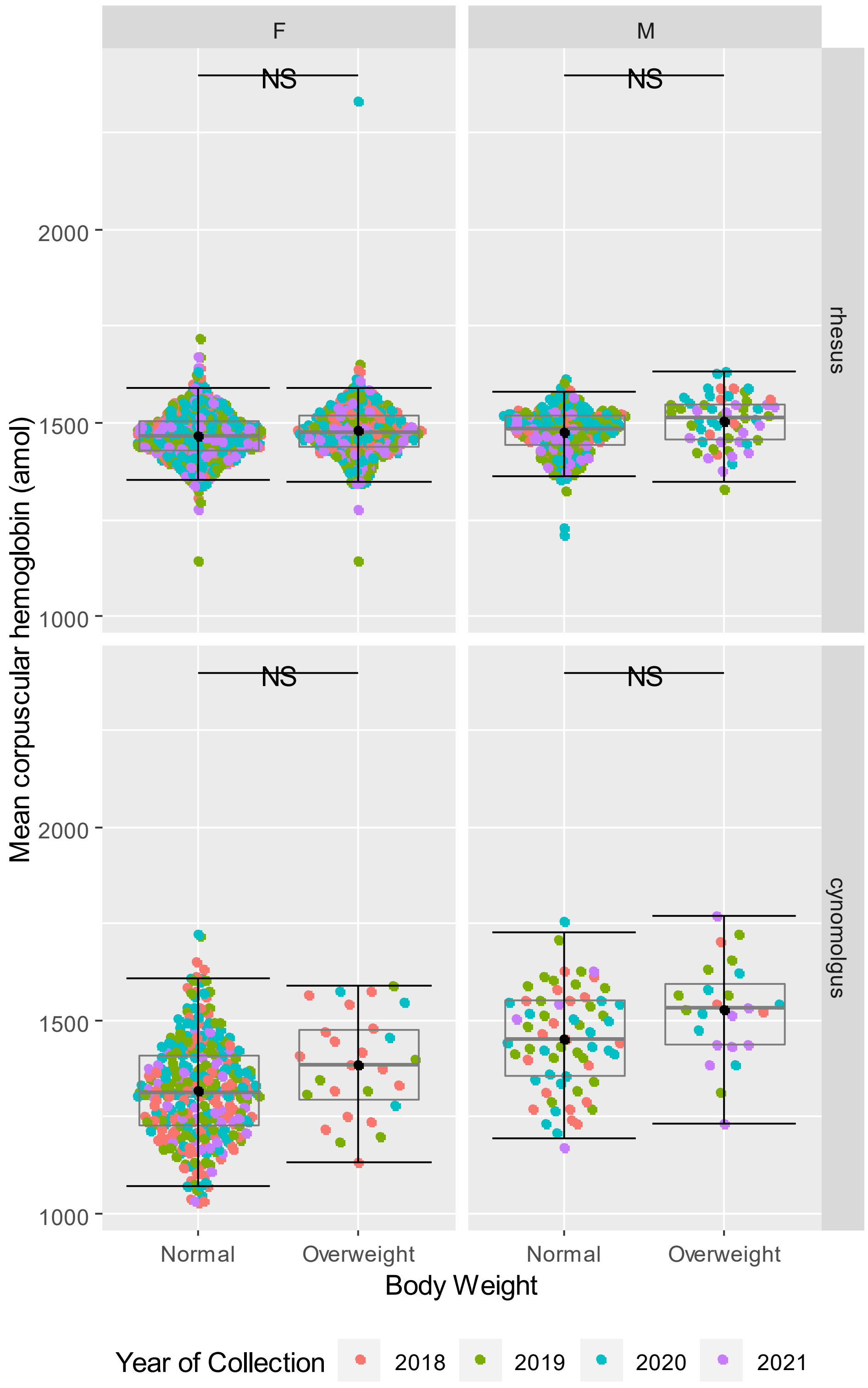

Figure S8 24. Mean Corpuscular Hemoglobin according to body weight in adult rhesus and cynomolgus macaques. Boxes indicate median and quartile ranges, black point indicates arithmetic mean and error bars indicate the normal range.

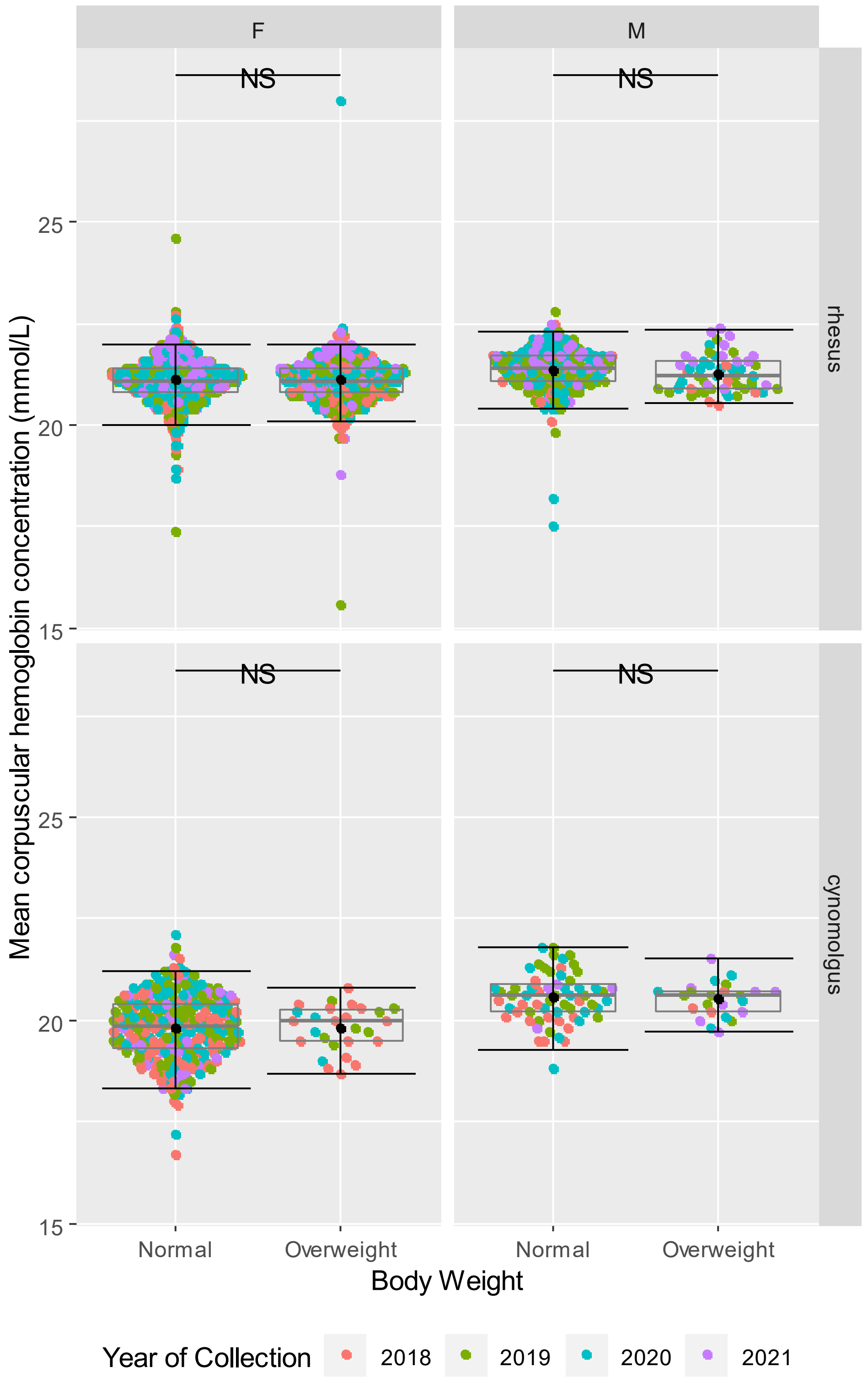

Figure S8 25. Mean Corpuscular Hemoglobin Concentration according to body weight in adult rhesus and cynomolgus macaques. Boxes indicate median and quartile ranges, black point indicates arithmetic mean and error bars indicate the normal range.

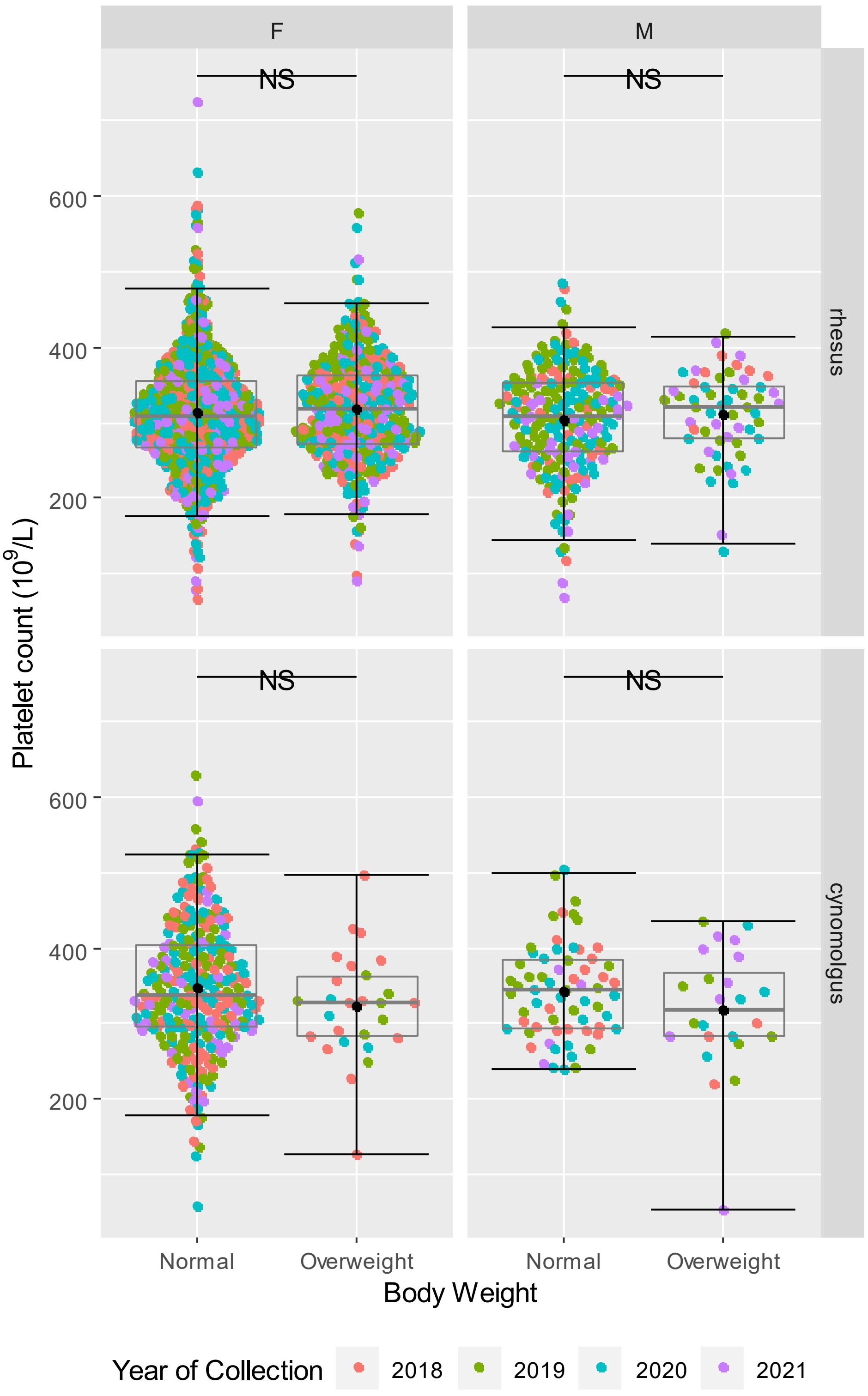

Figure S8 26. Platelet Count according to body weight in adult rhesus and cynomolgus macaques. Boxes indicate median and quartile ranges, black point indicates arithmetic mean and error bars indicate the normal range.

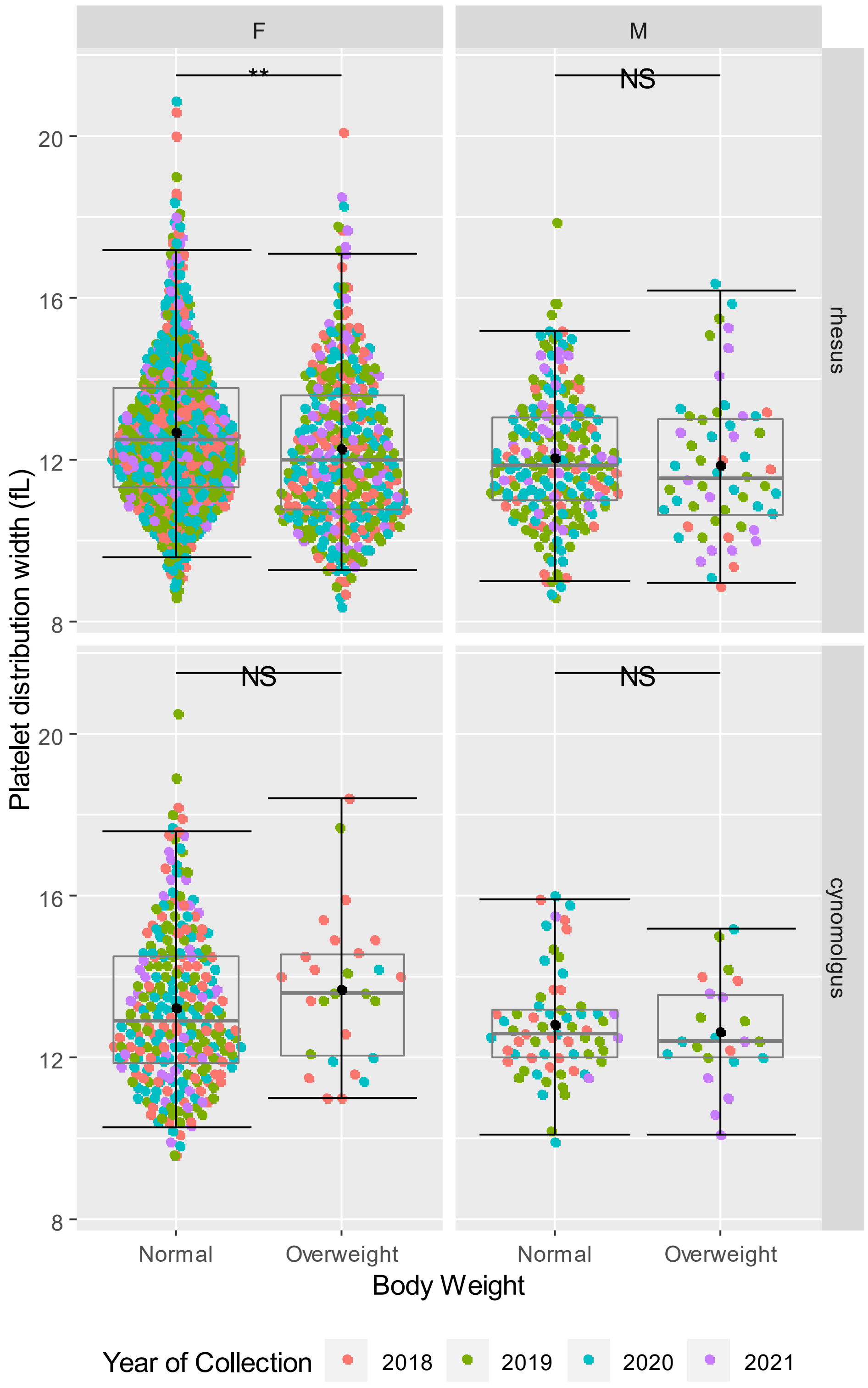

Figure S8 27. Platelet Distribution Width according to body weight in adult rhesus and cynomolgus macaques. Boxes indicate median and quartile ranges, black point indicates arithmetic mean and error bars indicate the normal range.

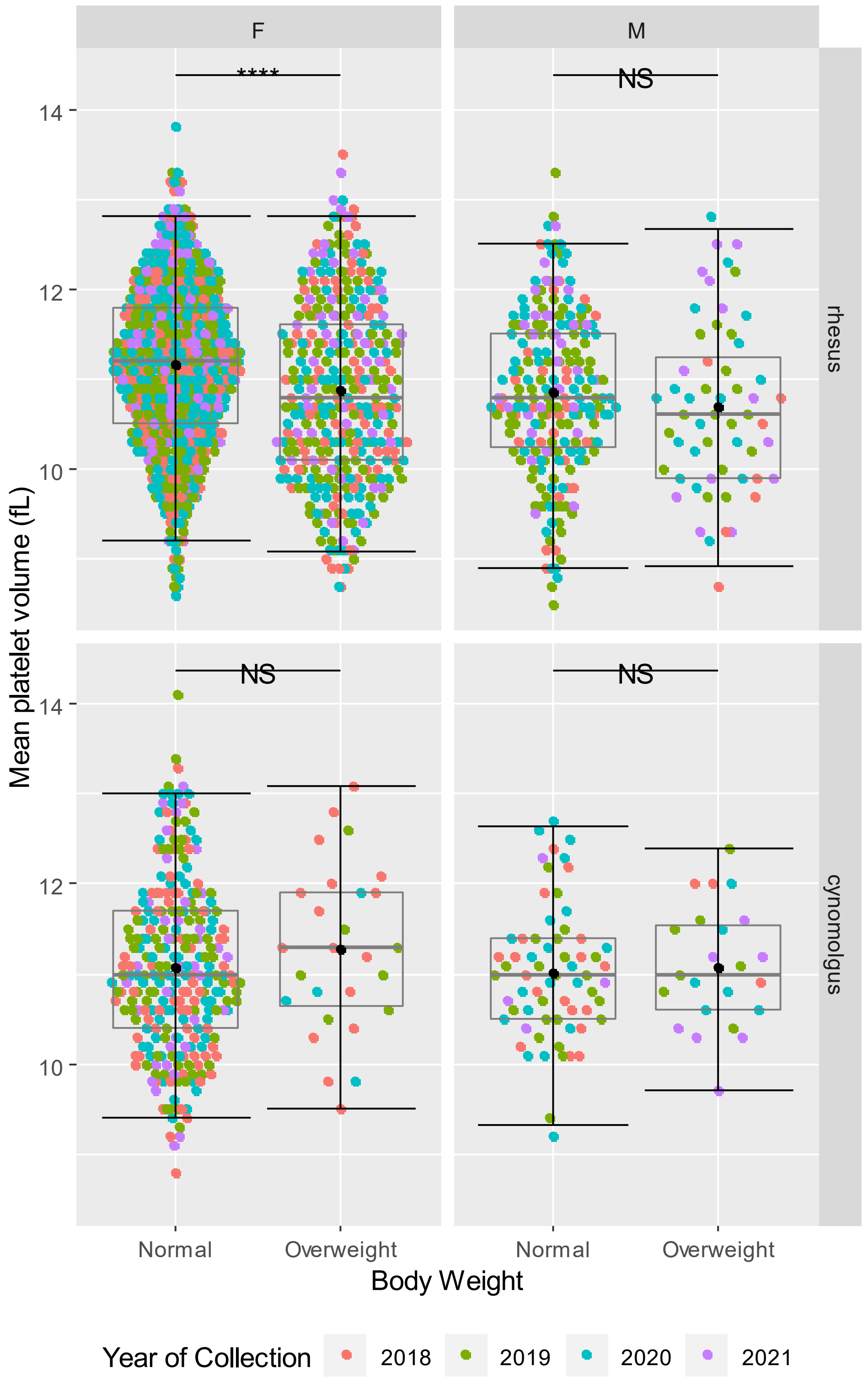

Figure S8 28. Mean Platelet Volume according to body weight in adult rhesus and cynomolgus macaques. Boxes indicate median and quartile ranges, black point indicates arithmetic mean and error bars indicate the normal range.

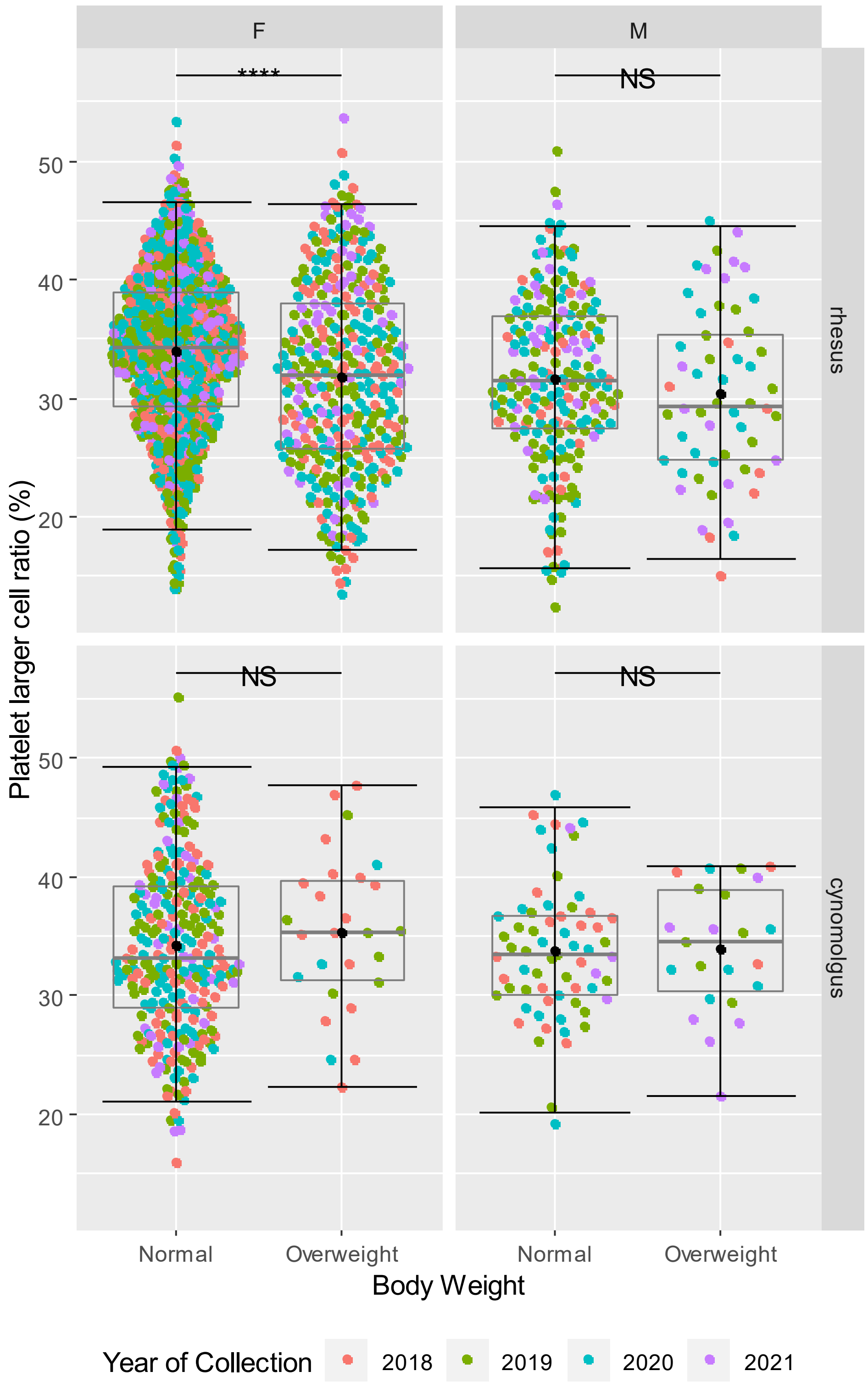

Figure S8 29. Platelet Larger Cell Ratio according to body weight in adult rhesus and cynomolgus macaques. Boxes indicate median and quartile ranges, black point indicates arithmetic mean and error bars indicate the normal range.

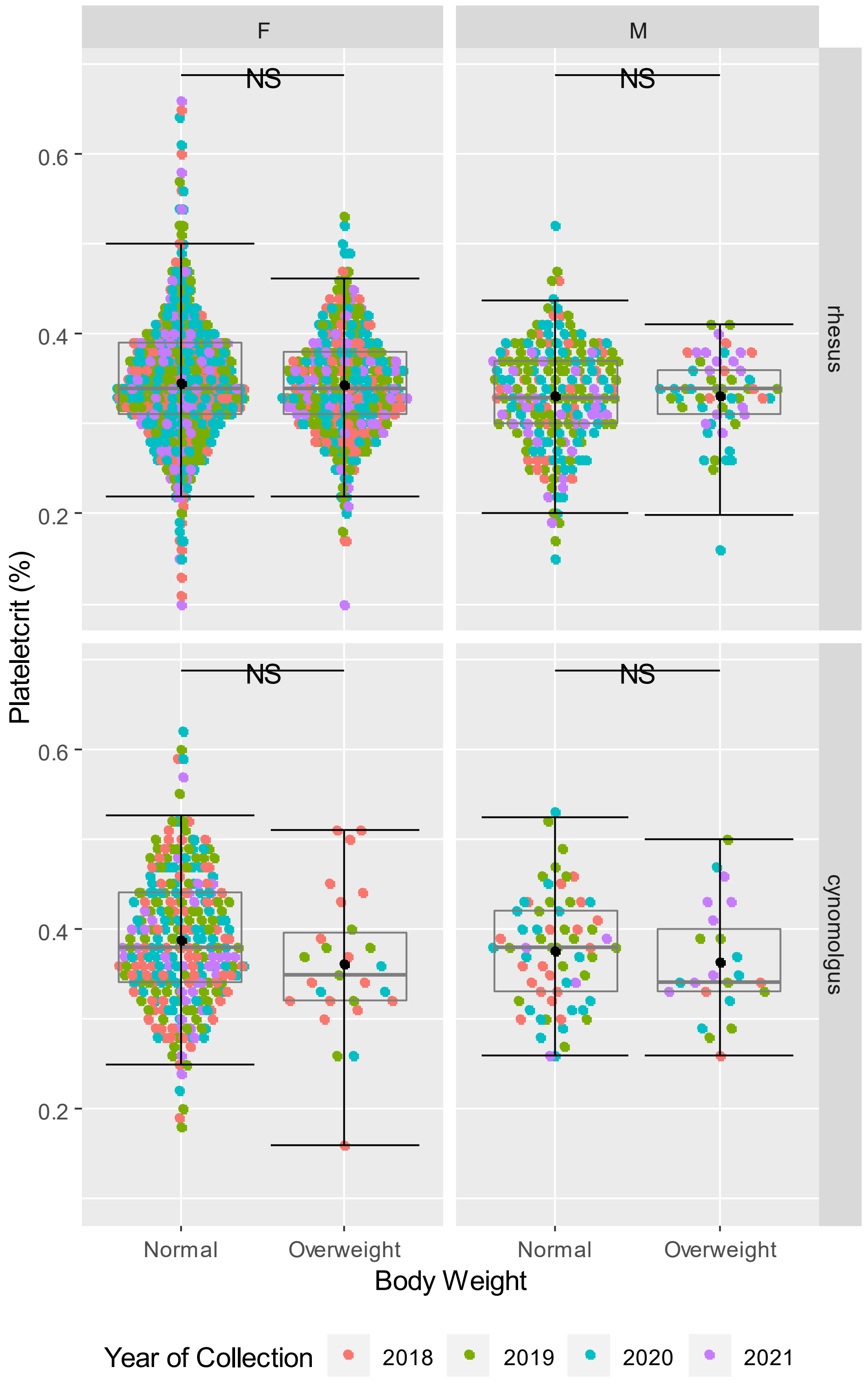

Figure S8 30. Plateletcrit according to body weight in adult rhesus and cynomolgus macaques. Boxes indicate median and quartile ranges, black point indicates arithmetic mean and error bars indicate the normal range.

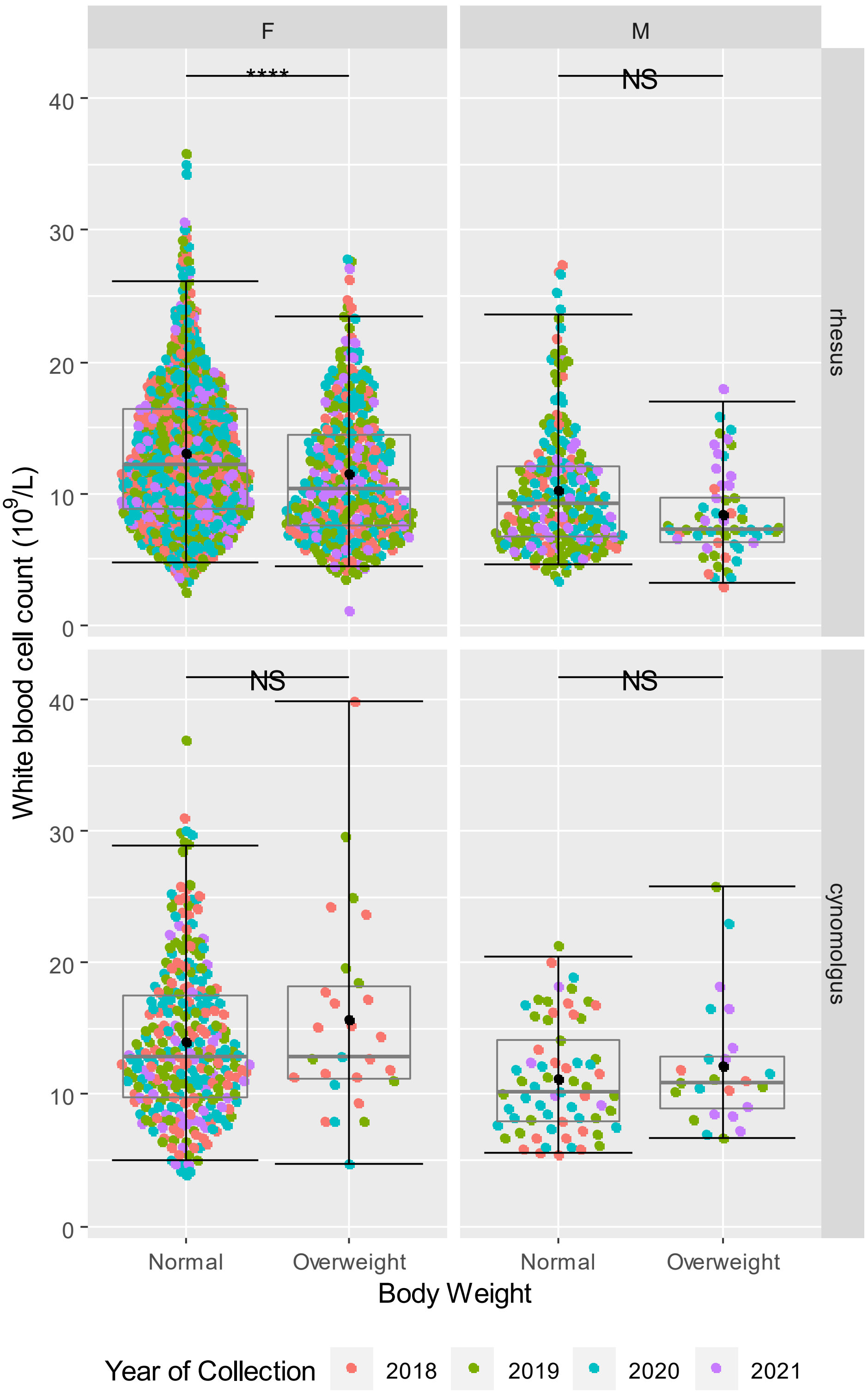

Figure S8 31. White Blood Cell Count according to body weight in adult rhesus and cynomolgus macaques. Boxes indicate median and quartile ranges, black point indicates arithmetic mean and error bars indicate the normal range.

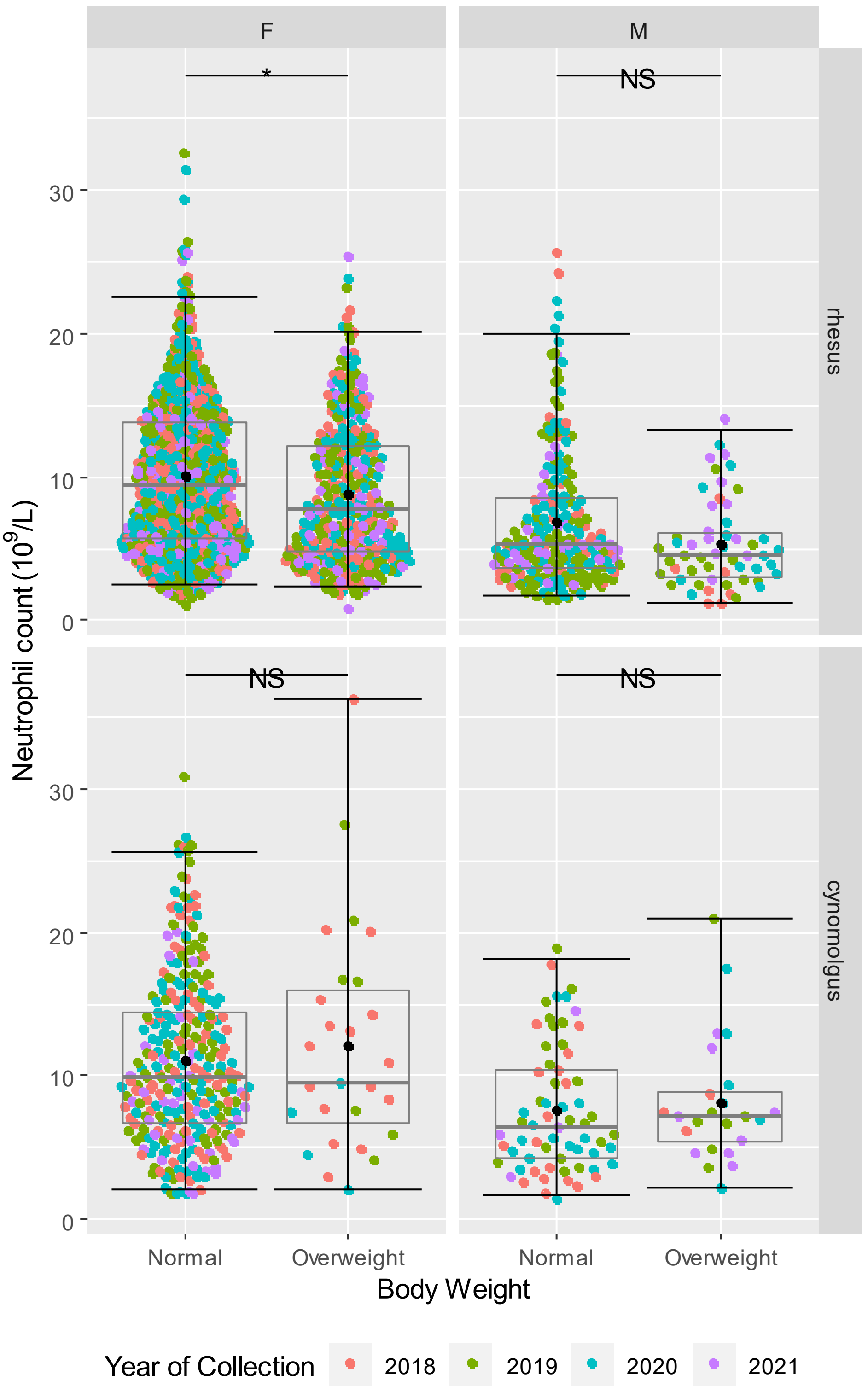

Figure S8 32. Neutrophil Count according to body weight in adult rhesus and cynomolgus macaques. Boxes indicate median and quartile ranges, black point indicates arithmetic mean and error bars indicate the normal range.

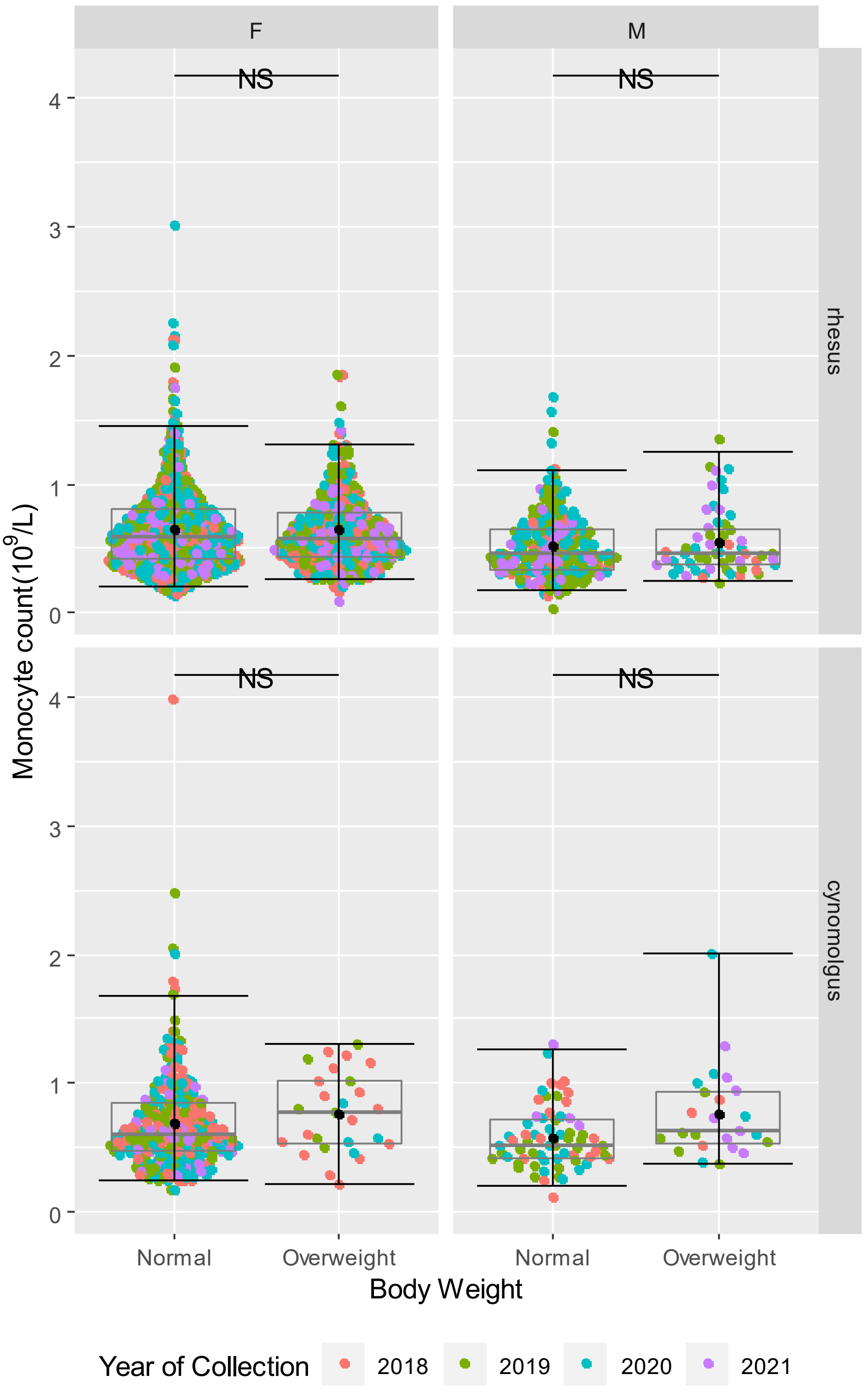

Figure S8 33. Monocyte Count according to body weight in adult rhesus and cynomolgus macaques. Boxes indicate median and quartile ranges, black point indicates arithmetic mean and error bars indicate the normal range.

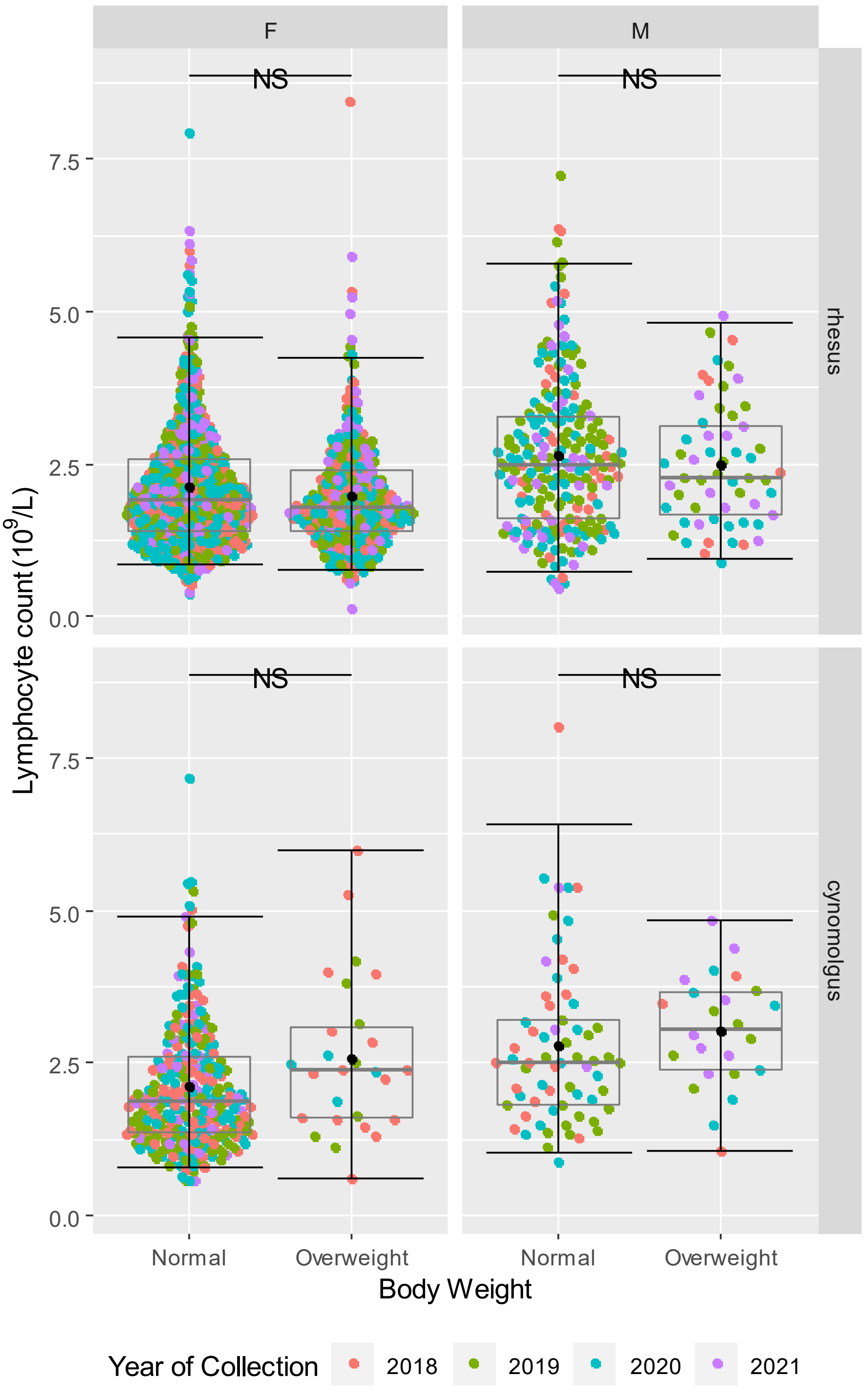

Figure S8 34. Lymphocyte Count according to body weight in adult rhesus and cynomolgus macaques. Boxes indicate median and quartile ranges, black point indicates arithmetic mean and error bars indicate the normal range.

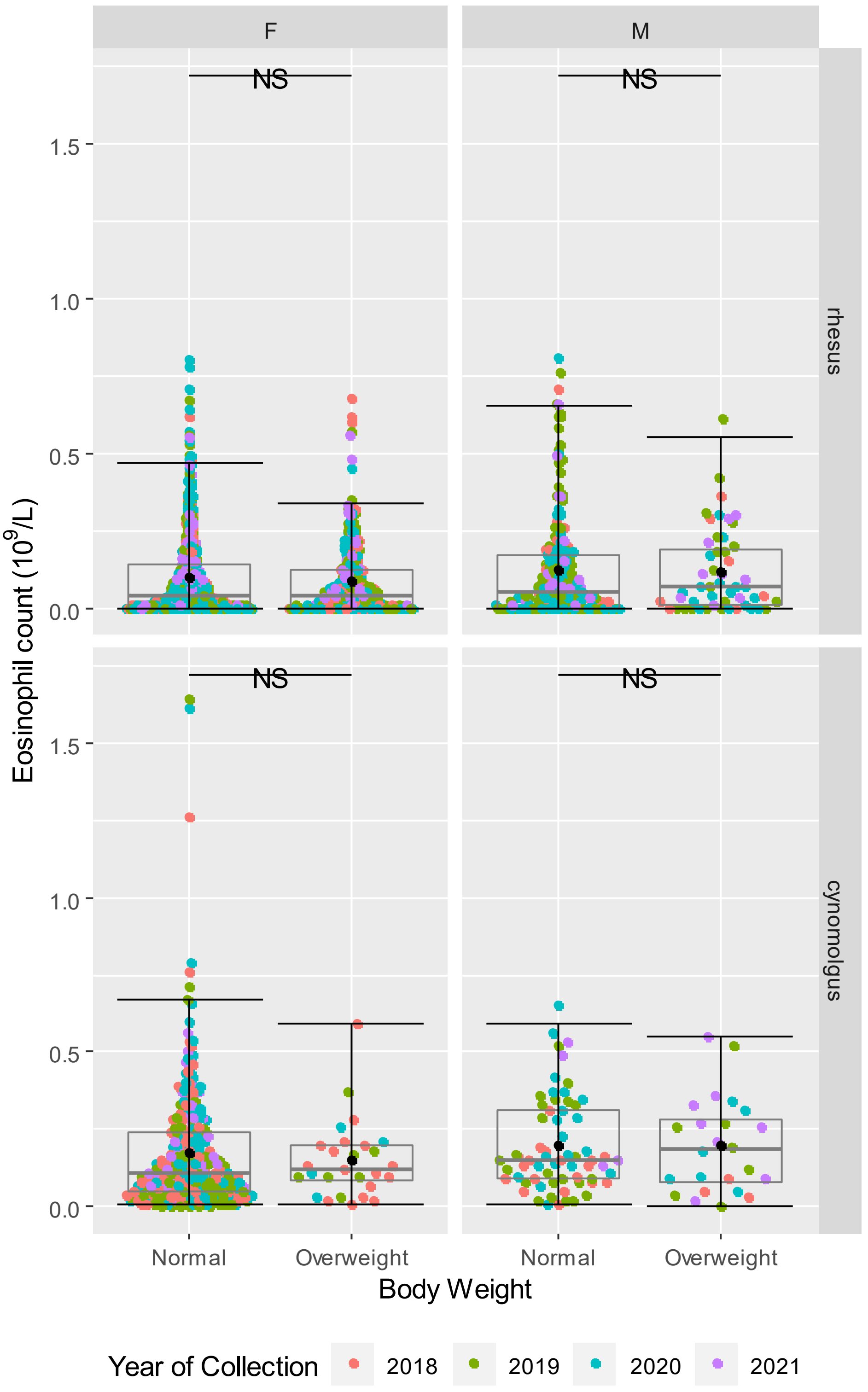

Figure S8 35. Eosinophil Count according to body weight in adult rhesus and cynomolgus macaques. Boxes indicate median and quartile ranges, black point indicates arithmetic mean and error bars indicate the normal range.

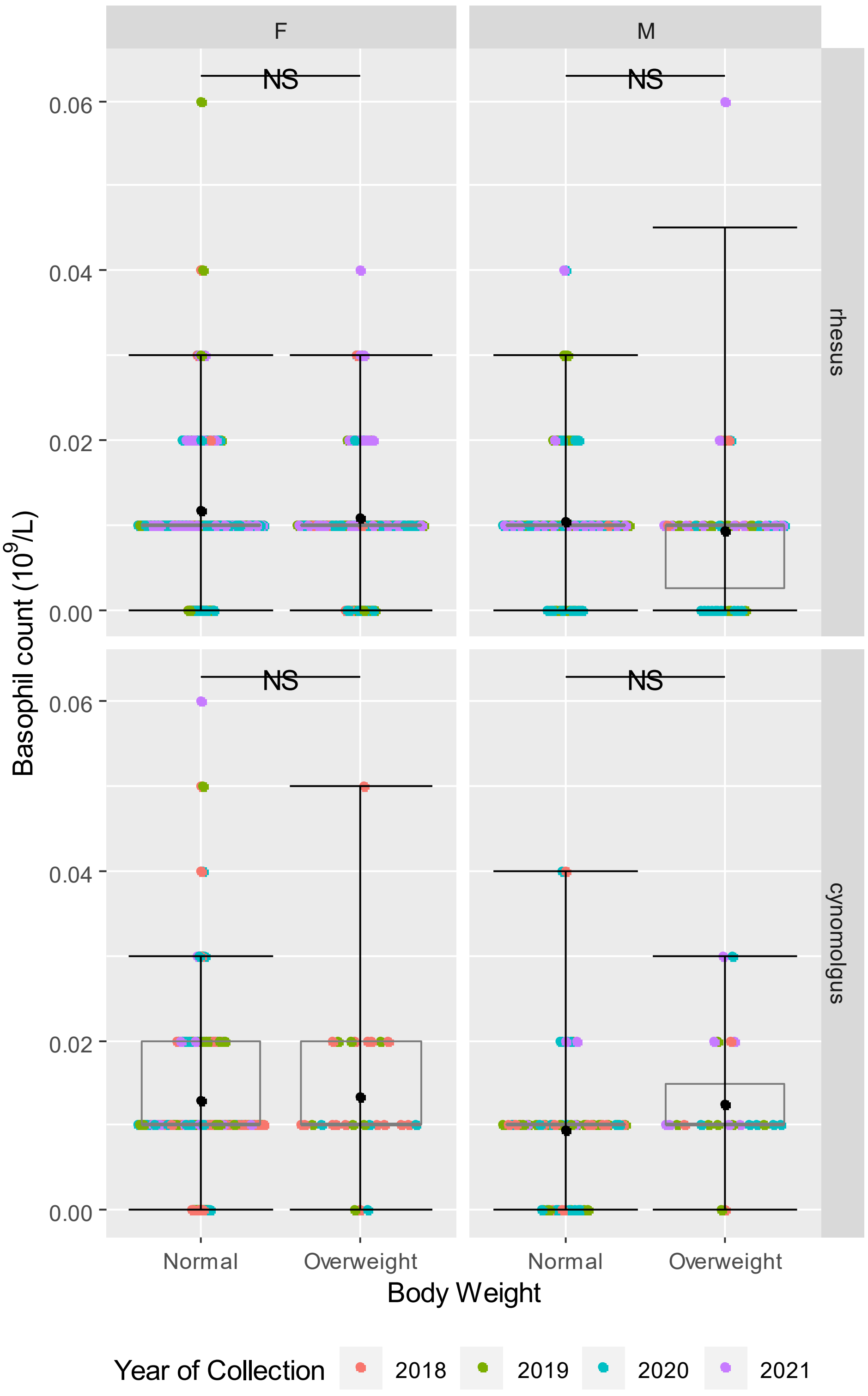

Figure S8 36. Basophil Count according to body weight in adult rhesus and cynomolgus macaques. Boxes indicate median and quartile ranges, black point indicates arithmetic mean and error bars indicate the normal range.
